# Towards near-term quantum simulation of materials

**DOI:** 10.1038/s41467-023-43479-6

**Published:** 2024-01-24

**Authors:** Laura Clinton, Toby Cubitt, Brian Flynn, Filippo Maria Gambetta, Joel Klassen, Ashley Montanaro, Stephen Piddock, Raul A. Santos, Evan Sheridan

**Affiliations:** Phasecraft Ltd., London, UK

**Keywords:** Computational methods, Quantum simulation, Electronic properties and materials

## Abstract

Determining the ground and excited state properties of materials is considered one of the most promising applications of quantum computers. On near-term hardware, the limiting constraint on such simulations is the requisite circuit depths and qubit numbers, which currently lie well beyond near-term capabilities. Here we develop a quantum algorithm which reduces the estimated cost of material simulations. For example, we obtain a circuit depth improvement by up to 6 orders of magnitude for a Trotter layer of time-dynamics simulation in the transition-metal oxide SrVO_3_ compared with the best previous quantum algorithms. We achieve this by introducing a collection of connected techniques, including highly localised and physically compact representations of materials Hamiltonians in the Wannier basis, a hybrid fermion-to-qubit mapping, and an efficient circuit compiler. Combined together, these methods leverage locality of materials Hamiltonians and result in a design that generates quantum circuits with depth independent of the system’s size. Although the requisite resources for the quantum simulation of materials are still beyond current hardware, our results show that realistic simulation of specific properties may be feasible without necessarily requiring fully scalable, fault-tolerant quantum computers, providing quantum algorithm design incorporates deeper understanding of the target materials and applications.

## Abstract (web summary)

The use of NISQ devices for useful quantum simulations of materials and chemistry is still mainly limited by the necessary circuit depth. Here, the authors propose to combine classically-generated effective Hamiltonians, hybrid fermion-to-qubit mapping and circuit optimisations to bring this requirement closer to experimental feasibility.

## Introduction

The ability to understand and design chemicals and materials is crucial for scientific, industrial, and commercial purposes^1^. This is evidenced by the central role of numerical simulation in guiding innovation in the multi-billion dollar chemical industry^2,3^. However, computational simulation methods on classical computers are limited by fundamentally inefficient descriptions of electron–electron interactions, hindering accuracy in the strong-coupling regime where many relevant technological applications are expected to appear^4^. A quantum computer (QC) can simulate these processes natively. This makes quantum simulation of materials and chemicals one of the most promising applications of quantum computing^5,6^. However in the near-term Noisy Intermediate-Scale Quantum (NISQ)^7^ devices are expected to be limited by low gate fidelities and small qubit numbers—severely constraining the range of deployable algorithms. Current estimates of circuit depth, given by the total number of layers of parallelizable quantum gates in a given quantum circuit, and qubit requirements of material and chemical quantum simulation lie well outside this regime (as we discuss in detail below). However with the race to demonstrate useful applications for near-term quantum computers begun in earnest, the question remains to what extent these estimates could be improved upon. In particular many naive cost estimates may not take full advantage of the features of the target application, such as computing materials’ local properties at equilibrium or simulating their out-of-equilibrium and excited state dynamics.

In this work we introduce several techniques that use general physical constraints of materials systems and are designed to work together to produce lower cost estimates for key quantum algorithms. The physics-based techniques are the careful selection of an active space consisting of maximally localised Wannier functions^8^ and the construction of a translational invariant local fermionic Hamiltonian for the modes in the active space, which consists of intra and intercell interactions/hopping. While active space methods are routinely employed for the atomistic simulations of materials^9,10^, as they reduce the dimension of the Hilbert space of the problem, incorporating them into a quantum simulation is not trivial, as an efficient fermion-to-qubit mapping is needed. Fermion-to-qubit mappings allow fermionic operators to be mapped into operations acting on the qubits of a quantum computer. Here, we leverage the structure of materials Hamiltonians found with active space and Wannier methods to introduce the following algorithmic techniques: a hybrid fermion-to-qubit mapping, designed to combine the compact encoding introduced in^11,12^ with a Jordan-Wigner mapping^13^, to efficiently describe fermion modes using the sparsity of the Hamiltonian and with a variable number of modes per site; a swap network optimization algorithm that specifically targets fermionic systems and has efficiency improvements over previous work^14^, in which fermionic modes encoded into adjacent qubits are swapped to facilitate the implementation of all the required interactions; and an optimized measurement protocol that aims to combine the efficiency of commuting measurements^15^ with the ease of implementation of qubitwise commuting measurements^16,17^. This is a key ingredient of variational quantum algorithms, in which the properties (usually the energy) of the trial quantum state must be computed accurately at each iteration of the algorithm as a sum of the expectation values of parts of the full system Hamiltonian^18^.

All these approaches combined together allowed us to reduce the resources (in particular, circuit depth and gate count) required to simulate realistic models of materials on a quantum computer by several orders of magnitude. This demonstrates that specific properties of materials may be obtained without requiring fully fault-tolerant quantum computers.

## Results

### Simulation algorithms

The two simulation algorithms we consider in this work are the variational quantum eigensolver (VQE) using a Hamiltonian variational ansatz^19^, and the time dynamics simulation (TDS) algorithm. VQE can be used to estimate properties of materials, such as equilibrium configurations, or correlation functions^20^. TDS on the other hand can be used to estimate response functions and spectral properties of materials^21^.

VQE employs a parametrized ansatz circuit to prepare an approximation to the ground state of a Hamiltonian *H* = ∑_*k*_*h*_*k*_ by varying the parameters to minimize the measured energy. The Hamiltonian variational ansatz is a sequence of circuit layers *l* of the form ${\prod }_{k}{e}^{i{t}_{lk}{h}_{k}}$, with *t*_*lk*_ variational parameters^19^. TDS simulates time evolution under the Hamiltonian of a given input state. It achieves this by breaking up the evolution into a sequence of circuits approximating short-time dynamics of duration *δt*, which in the simplest case each take the form ${\prod }_{k}{e}^{i\delta t{h}_{k}}$. Our approach to estimating the costs of these algorithms is to estimate the cost of implementing the subroutine circuit $U(\overrightarrow{a})={\prod }_{k}{e}^{i{a}_{k}{h}_{k}}$, since for both TDS and VQE the number of times one needs to execute this subroutine may vary. See Supplementary Note 4 for more details. We also consider the cost of state preparation for TDS and VQE (Supplementary Note 4B), as well as the cost of performing measurements (Supplementary Note 4D).

Ultimately our aim is to implement the subroutine $U(\overrightarrow{a})$ in the shortest possible circuit using as few qubits as possible while remaining faithful to the simulation goals of TDS and VQE. Our strategy is straightforward: use as small a Hilbert space as possible – reducing qubit count; minimize the number of terms required to faithfully represent *H* – reducing the number of entries in the product *U*; and choose a representation along with a compiling routine to minimize the cost of executing each individual step ${e}^{i{a}_{k}{h}_{k}}$. These three goals are achieved respectively by: identifying a good active space in the Bloch basis; identifying maximally localized Wannier functions^8^ on the active space and computing the Hamiltonian coefficients in this basis, truncating any small terms; employing a hybrid fermionic encoding tailored to the problem in conjunction with a dynamically optimized fermionic swap network to minimize the cost of executing each interaction and maximize the number of parallel executions. Each of these approaches – as it will be discussed below – involves the introduction of several technical insights, and furthermore all of these solutions are designed synergistically, operating in tandem to optimize all three goals. Before proceeding with further details, we briefly discuss some previous work, which has performed similar cost analyses.

#### Previous work

Qubit and gate resources required for Trotterized Hamiltonian simulation algorithms of fully local Hamiltonians have recently been investigated by Kanno et al.^22^. Here, effective Hamiltonians of several unit cells of materials have been constructed starting from a classical description that accounts for the important chemistry of the active space^10^. The resources to implement a single Trotter step are investigated on devices with nearest-neighbor connectivity in terms of CNOT and arbitrary single-qubit gates. They use a Jordan-Wigner (JW) transform to encode the fermionic modes, and fermionic swaps^23,24^ to deal with the large operator weight of the encoded Pauli operators. This leads to a scaling of the gate count that is $O({N}_{{{{{{{{\rm{cells}}}}}}}}}^{2})$ for a Hamiltonian defined in *N*_cells_ unit cells.

In comparison, our approach attains *O*(*N*_cells_) scaling of the number of gates, as the intercell interactions are implemented through ancillas in the compact encoding^11^. This incurs a qubit overhead proportional to the number of unit cells. Importantly, considering that the main problem of current QCs is the presence of gate errors, our approach allows us to achieve a layer depth for single Trotter step that is independent of the size of the system, in stark contrast with the $O({N}_{{{{{{{{\rm{cells}}}}}}}}}^{2/3})$ depth using JW (in a cubic system with nearest neighbour interactions)^22^. Delgado et al. recently gave a detailed resource analysis of quantum algorithms for determining properties of battery materials, such as equilibrium voltages and thermal stability^25^. They use a first quantisation approach with the plane wave basis and compute the cost of the quantum phase estimation algorithm. Considering one unit cell of the material Li_2_FeSiO_4_ with 156 electrons, these authors find a Toffoli gate cost of between 10^11^ and 10^15^ for quantum phase estimation, depending on the number of plane waves and level of accuracy required.

Counting the overall number of Toffoli or T gates is an appropriate approach to estimate complexity in the fault-tolerant regime, as this quantity directly determines the (very significant) overhead required for fault-tolerance. For near-term quantum computers, depending on the architecture, quantum circuit depth can be more appropriate, for several reasons. First, quantum computations are limited by decoherence, which sets an upper bound on the overall running time, as measured by circuit depth. Second, as errors can be seen as spreading out across a quantum circuit within a “lightcone”, lower-depth circuits lead to improved localisation of errors. Third, as the circuit depth determines the running time, a lower-depth circuit executes more quickly.

Several other works have produced quantum algorithmic resource costs tailored for the fault tolerant era in molecular systems^26–28^, the interacting electron gas (jellium)^29–32^, and for periodic systems^33^. While our focus is on identification of the active space for periodic materials, we remark that the idea of reducing the cost of quantum simulations for molecular systems has been proposed by Reiher et al.^34^. Molecular active space methods have also been explored in highly sophisticated implementations based on quantum information measures^35,36^ that have enabled automated protocols for the choice of active space^37,38^. Finally, we note that active space methods for periodic systems, described in the section below, have been recently employed in the context of quantum computation for the specific task of computing properties of spin defects in semiconductors using quantum embedding theory^39^.

### Identifying the active space

Material systems specifically have a number of features which—as we will demonstrate—may be leveraged to great effect. The most significant feature is the translational symmetry of the Hamiltonian. This symmetry allows for efficient approximate diagonalization in a band (Bloch) basis. In this basis it is easier to identify a subspace of the single-particle Hilbert space around the Fermi level wherein the dominant dynamics occur. By truncating to this subspace, the number of qubits required and the number of terms in the Hamiltonian may be reduced.

Specifically, the fermionic Hilbert space *F* is given by a tensor sum over *n*-particle Hilbert spaces, each consisting of *n*-fold antisymmetric products of single-particle Hilbert spaces *F*_1_. In order to reduce the size of *F* we truncate *F*_1_ to a subspace, called an *active space*, which ideally captures the dominant dynamics of our Hamiltonian *H* ^9,10^. As discussed in the “Active space and Wannier function" section of Methods, we identify this subspace using density functional theory (DFT)^40^. In DFT, *H* is approximated by an auxiliary Hamiltonian which replaces the fermion-fermion interactions with an effective external potential, turning the problem from a many-body problem to a single-particle problem, which may be solved efficiently. The eigenstates of this effective Hamiltonian are called Kohn-Sham (KS) states. In principle, the latter have no strict physical interpretation and are just a tool to obtain the ground state energy and density of the original Hamiltonian. However, one of the reasons behind the success of DFT is that, for many weakly correlated materials, KS states also provide a good description of the actual electronic structure and can therefore be employed to compute a number of additional physical properties. This picture breaks down for strongly correlated systems, in which KS states should only be used to calculate ground state energy and density^41^. Unfortunately, even the latter is a formidable task, due to inconsistencies in the approximate exchange correlational functional, and typically requires the introduction of ad hoc parameters beyond the standard DFT formulation^42^. In spite of that, the recent success of ab initio embedded approaches for describing materials and molecular properties, i.e., DFT+DMFT^43,44^ and DFT+DMET^45–49^, have demonstrated that KS states can be used as a good starting point for building low-energy effective models of correlated materials. These approaches then proceed to solve smaller, auxiliary problems within given active spaces that cannot be addressed with single-particle methods. Once solved, the results from the smaller active space are combined with the rest of the system. All such active space-based approaches have the potential to offer reliable corrections to the underlying single-particle description of materials but do not in general guarantee quantitative accuracy for all properties of interest, across varying time scales. Crucially, the regime of validity for active space approaches can be systematically improved on by assessing the impact of including more physical orbitals^50,51^, inclusion of screening^9,52,53^, as well as incorporating double-counting in an exact way^54^. As our approach is also based on determining an active space, it can be improved by using the same classical techniques mentioned above, to account for potential inaccuracies and resolve them.

**Fig. 1:**
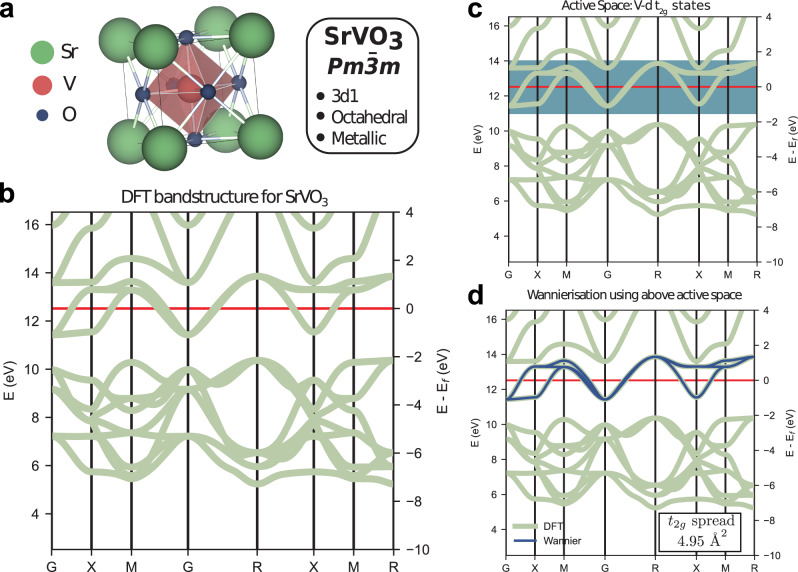
Active space of SrVO_3_. **a** Unit cell of the SrVO_3_ with spacegroup *Pm*$\overline{3}$*m*, 3d1 nominal electronic valence configuration, and octahedral coordination environment. **b** The ground state electronic bandstructure as predicted using DFT along the high symmetry path in the Brillouin zone. The red line indicates the position of the Fermi level. **c** An energy range (shaded blue) around the Fermi level is defined. **d** The bands around the Fermi level (dark blue) contained in the previous energy range are considered in the construction of the Hamiltonian.

Assuming that all the relevant degrees of freedom are included, these models provide a powerful tool to compute many low-energy properties of correlated systems. This approach is particularly suitable for materials with a few bands around the Fermi level, which retain a strong atomic character, such as the ones emerging from *d* and *f* localized orbitals. Crucially, this situation is common in many strongly correlated materials. In this case, the active space can be identified with the Hilbert space spanned by the KS states whose energy lie within a judiciously chosen energy range around the Fermi level, chosen to encapsulate the most relevant electronic degrees of freedom. This procedure is illustrated in Fig. 1. For more details about the choice of the active space and the approximations introduced, see the “Active space and Wannier functions" section of Methods and Supplementary Note 2F. Despite the reduced complexity of these effective models, their Hilbert space still grows exponentially with the systems’ size. Hence, only models with a limited number of degrees of freedom can be solved on a classical computer. This work shows that the resources required to solve larger and more general instances, which are beyond the capabilities of state-of-the-art classical solvers, on a quantum computer, can be reduced significantly via tailored quantum algorithms.

### Localized Wannier Representation

Having identified the single-particle active space, there remains an additional choice of which basis is best suited to representing *H* in second quantized form. In addition to the Bloch basis—which represents electrons in momentum space—crystalline solids possess a second natural basis, the Wannier basis, which represents electrons in real space. Here, we leverage maximally localized Wannier functions, which are single-particle wave functions localized in real space around the unit cell coordinates of the material^8,55^. In this basis, interactions can become highly localized, further reducing the number of dominant terms in the Hamiltonian. An added benefit of operating in the Wannier basis is that highly localized interactions lend themselves well to efficient representations on qubit devices. In particular, we find that for the materials we consider the dominant interactions of the Hamiltonian expressed in the Wannier basis can be confined to act between nearest and next-nearest neighbours. This has a twofold benefit. The first and most significant is that the number of Hamiltonian terms scales linearly with the size of the system *N*_cells_. This is a significant reduction in comparison to an expression in momentum space, where particles with significantly different momenta may strongly interact, leading to the number of quartic interactions growing as ${N}_{{{{{{{{\rm{cells}}}}}}}}}^{3}$. The second is that local interactions are better suited to representation on quantum devices, due to the challenges in mapping fermions on qubits and the overhead of implementing interactions between distant qubits in hardware. For more details on the Wannier representation see Methods and Supplementary Note 2C.

### Hybrid fermionic encoding and fermionic swap networks

Any simulation of a fermionic system on a qubit-based QC requires a mapping between the fermionic Hilbert space, and the multi-qubit Hilbert space of the QC—most conveniently given by a correspondence between fermionic and qubit operators^11,12,56–62^. In order to capture the exchange statistics of the fermionic algebra, interactions acting on a small number of modes in the fermionic system may be mapped to interactions acting on a large number of qubits on the QC. This is exemplified in the most commonly used mapping, the JW transform, which maps fermionic creation and annihilation operators to string-like qubit operators. Interactions acting on large numbers of qubits introduce large circuit overheads in quantum simulation, making them undesirable. For example, under the JW transform—which many naive methods use to estimate algorithmic costs—simulating the dynamics of one of the 2-mode hopping terms may require a quantum circuit whose depth grows with the size of the system^22^. However if the interactions are sparse such that each fermionic mode is involved in a relatively small number of interactions, then these problems may be avoided by a judicious choice of mapping^11,57,58^. By operating in the Wannier basis and localizing the interactions we can manifest this kind of sparsity.

In the Wannier basis each unit cell coordinate **R** may be thought of as a coarse-grained “site”, consisting of a number of densely interacting modes, which also interact densely with neighbouring sites, but sparsely with distant sites. In cases where there is a high degree of interaction between modes in the Hamiltonian, it is simply not possible to map all interactions to low-weight operators, regardless of the choice of mapping. Inevitably some interactions will be high-weight, in a similar fashion to the JW transform. Typically in this case, in lieu of low-weight representations, a JW transform is employed and a fermionic swap (fswap)^23^ protocol is applied wherein fermionic modes are dynamically re-ordered throughout the algorithm such that each interaction admits a low-weight representation at some point in the protocol. In the Wannier basis the mixture of dense short-range interactions with sparse long-range interactions suggests a hybrid approach wherein a sparse set of interactions between sites are represented with low-weight operators, and the modes within a site are represented by a JW style encoding, to which a custom-designed fswap protocol is applied. We introduce a family of encodings tailored to this approach which we call hybrid encodings—they are a hybridization of the JW transform on sites and the compact encodings introduced in refs. ^11,12^. We believe this is the first concrete example of the use of this kind of method, wherein two encodings are used jointly to leverage their respective strengths, and we expect this strategy to become commonplace in the future design of fermionic simulations. The fermionic encodings we consider may be understood as having a graph geometry, wherein modes are adjacent on the encoding when hopping terms between those modes admit a low-weight representation. For more details on the hybrid encoding, see the “Encoding" section of Methods and Supplementary Note 3.

Given the geometry of the encoding and the specific structure of the particular Hamiltonian under consideration, it is not suitable to use existing fswap protocols or devise a general-purpose fswap protocol. Instead we introduce an algorithm that searches for a custom fswap protocol that minimizes the number of fswap layers required to implement all terms in a given target Hamiltonian. Our algorithm targets interactions, which may occur on two, three, or four modes, each interaction having its own ideal adjacency conditions on the geometry of the encoding—for example, two-mode interactions are ideally adjacent, while four-mode interactions only require pairings of modes to be adjacent. The search routine employs a steepest descent heuristic, wherein a layer of fswaps is chosen from a pool of candidates by how much it reduces the distance of the remaining interactions from their ideal adjacency configuration. Success of this strategy depends on a carefully chosen notion of distance that leads to an efficient protocol. When used in conjunction with the custom hybrid encoding we find that the introduction of custom fswap protocols can have substantial impact on the total circuit depth. For example, the depth of implementing onsite and nearest neighbour terms for H_3_S is reduced from 7000 to 1214 (see Supplementary Table 10). Further details about the fswap protocol appear in Methods (section “Localized Wannier Representation") and Supplementary Note 5A.

### Compiler

In order to understand the potential gains in circuit depth that can be achieved from the strategy discussed above, we have built a compiler that incorporates these strategies along with additional optimizations – such as small-scale circuit optimizations where possible for better overall depth, and leveraging symmetries in the computation of basis wave functions for faster compile times. The compiler takes as input the atomic specification of the material, and produces an optimized circuit for VQE and TDS via the following automated steps: identifying an active space in the band basis; computing a maximally localized Wannier basis; computing the coefficients of the Hamiltonian in this basis using Monte-Carlo integration; specifying an appropriate encoding for the Hamiltonian; optionally decomposing terms into appropriate commuting groups for better Trotter error in TDS; finding an optimal fswap protocol, consisting of alternating layers of fswaps and interaction terms ${e}^{i{a}_{k}{h}_{k}}$; decomposing interaction terms and fswaps into circuit operations; costing and analyzing the resulting circuits. The compiler can handle a fully populated finite lattice or, given a material unit cell, it can compile a circuit that is infinitely tilable (see Supplementary Note 5B), allowing a circuit depth independent of the system’s size. We also introduce a routine for optimizing measurement protocols targeted at fermionic systems in order to reduce overheads in measuring energy for VQE. These measurements can be implemented in constant depth – much more efficiently than general commuting measurements – but provably require fewer rounds than the commonly used qubitwise commuting measurements^16,17^ (see Methods section “Measurement schemes" and Supplementary Note 4D). The compiler is also able to optionally prioritize minimizing Trotter error at the expense of circuit depth, and can produce an estimate of the Trotter error of a given circuit for a given target simulation time (see Supplementary Note 6C 2). A full-stack analysis of SrVO_3_ using our compiler is discussed in Supplementary Note 6C 1.

## Discussion

Selected results appear in Table 1, where we compare the circuit depth obtained by our methods with a standard, generic method that does not exploit the structure of the Hamiltonian (see Appendix A). To simplify our analysis, we assume that the hardware has all-to-all connectivity and that any two-qubit operation has circuit depth 1 while single-qubit gates have cost zero. The materials analysed here represent a selection of systems whose behaviours are dominated by distinct underlying mechanisms: they span a minimal but wide structural, chemical, and technological range. Strontium vanadate (SrVO_3_) is a strongly correlated material that serves as a benchmark for post-DFT methods^63^, gallium arsenide (GaAs) is a fairly well-understood material with many technological applications. Likewise, silicon (Si) is the cornerstone material used in modern electronics^64^ and is also important in many other applications, such as solar technologies^64^. Recently, hydrogen disulphide (H_3_S) has been found to host a high superconducting transition temperature at high pressures^65^. Finally, lithium copper oxide (Li_2_CuO_2_) is a material used in advanced lithium-ion battery technology^66^.

**Table 1:** Summary of resources needed to implement a single VQE layer that simulates the Hamiltonian of different materials, without accounting for initial state preparation

			Bands	Qubits	Gates	Depth
Material	Applications	Method				
GaAs	Semiconductors^82^, transistors^83^, solar cells^84^, spintronics^85^	This work	4	1120	4.1E+05	7.9+E03
		Baseline estimate	6	1500	3.0E+12	3.5E+09
H_3_S	Superconductors^65^	This work	7	1870	2.4E+06	3.7E+04
		Baseline estimate	6	1500	3.0E+12	3.5E+09
Li_2_CuO_2_	High-capacity battery cathode^66^	This work	11	1024	2.3E+05	8.4E+03
		Baseline estimate	14	1260	1.5E+12	2.1E+09
Si	Semiconductors^86^, solar cells^64^	This work	4	1120	4.5E+05	8.5E+03
		Baseline estimate	3	750	1.8E+11	4.3E+08
SrVO_3_	Solar cells^87^, batteries^88,89^	This work	3	180	7.5E+03	8.8E+02
		Baseline estimate	16	864	3.2E+11	6.7E+08

We considered a system consisting of a 3 × 3 × 3 supercell for SrVO_3_, 5 × 3 × 3 supercell for Li_2_CuO_2_ and a 5 × 5 × 5 supercell for all the other materials. *Baseline* estimates are based on standard methods available in the literature – namely employing the Jordan-Wigner transform in the Bloch basis – without considering the structure of the Hamiltonian. See Appendix A for details.

Since no prior work has been done for estimating the costs of these materials, we include in Table 1 estimates for the same materials in the Bloch basis employing the JW transform. These are all standard approaches used commonly in estimating simulation cost overheads of material and chemical systems. These estimates are based on bounds derived from term counting and qubit support, and include the potential for fswap protocols (see Appendix A for details). It is evident that our methods, which take advantage of the physics of the material system, yield substantial improvements over these standard methods.

The improvements in circuit depths demonstrated in this work bring material simulation applications out of the domain of impossibility for near-term quantum computers and into a regime where, at least for some materials, the costs are potentially within touching distance of the requirements of near-term devices provided improvements in hardware and algorithms continue at their current rate.

For example, IBM has reported two-qubit gate fidelities approaching 99.9%^67^. Error mitigation experiments employing virtual distillation by Google^68^ have demonstrated useful results at circuit fidelities of 10%. Naive estimates of gate requirements to achieve a target circuit fidelity of 10%, with 99.9% two-qubit gate fidelity yield a 2300 two-qubit gates budget. Furthermore, IBM has run accurate Hamiltonian simulation experiments involving approximately 3000 two-qubit gates^69^. Thus even given skepticism about currently reported gate fidelities, or required circuit fidelities, we may still accept that a naive two qubit gate count budget on the order of 2000-3000 is reasonable for a near-term application. SrVO_3_ has a two-qubit gate count of 7507 for the system size we consider. This suggests that to achieve a single circuit layer of the kind we consider for SrVO_3_ would require an approximate 3 fold two-qubit gate count reduction. A reduction of this type would make a single layer of HVA VQE potentially feasible for SrVO_3_ – with even a very small number of layers of VQE potentially being sufficient to observe qualitative features of the model^20^. However this still leaves standard first-order Trotter dynamics for long timescales out of reach, since many layers of Trotter are typically required. We are optimistic that the requisite gate counts for this kind of primitive can be reduced, that the algorithmic requirements of both TDS and HVA VQE can be further improved upon, and that other interesting materials may exist with better gate count requirements.

It is important to stress that our results do not constitute conclusive evidence that these applications will yield quantum advantage on near-term devices. First, although ab-initio embedding and active space-based methods have shown the potential to improve the description of certain properties of materials, a rigorous quantification of the trade-offs introduced by these approximations in the simulation of large materials’ properties and dynamics is an open problem and will require additional investigation. Further, the figures we obtained in this work give a sense of scale but do not take into account native availability of the required gates on hardware and do not constitute a concrete proposal for a quantum advantage experiment. Rather, our results demonstrate that material simulation on near-term devices is a promising direction to pursue in earnest, and that a simulation of this type may be feasible in the near future – whereas a naive estimate would suggest that such simulations are well within the fault-tolerant regime.

Simulating materials is a promising application for quantum computers. The progress reported here incorporates a number of complementary approaches across the full quantum materials simulation stack that, when combined together, reduce the quantum circuit depth requirements by orders of magnitude compared to naive baseline estimates. Crucially, the design process produces quantum circuit depths for Trotter and VQE layers which are independent of the material’s size by taking advantage of the locality of materials Hamiltonians. We expect that our proposed framework for materials simulation on quantum computers can be enhanced further by continuing to incorporate physically motivated structure into the choices of fermionic encodings, basis representations, and fswap network protocols. One consequence of this work is the identification of certain materials, from the small set we have considered, which are particularly well suited to quantum simulation due to the details of their physics—such as SrVO_3_. Beyond reducing circuit depths and improving error mitigation techniques, identifying the appropriate physical systems, which are best suited for simulation on NISQ devices is essential. The development of the tools described in this work can be used to allow the application of data-driven and high-throughput techniques to understand the classes of materials most amenable to quantum simulation. Our results show that considering seriously the structure of the physical problem at hand and incorporating these considerations into the design of quantum algorithms can accelerate progress towards quantum advantage.

## Methods

### Electronic Hamiltonian

The second quantized Hamiltonian of the electronic degrees of freedom for a material with no spin-orbit interactions or magnetic fields is

1 $$H=	 \mathop{\sum}\limits_{\sigma }\mathop{\sum}\limits_{{\lambda }_{1},{\lambda }_{2}}{t}_{{\lambda }_{1}{\lambda }_{2}}{c}_{{\lambda }_{1}, \sigma }^{{{{\dagger}}} }{c}_{{\lambda }_{2}, \sigma }\\ 	+\mathop{\sum}\limits_{\sigma,{\sigma }^{{\prime} }}\mathop{\sum}\limits_{{\lambda }_{1},{\lambda }_{2},{\lambda }_{3},{\lambda }_{4}}{V}_{{\lambda }_{1}{\lambda }_{2}{\lambda }_{3}{\lambda }_{4}}{c}_{{\lambda }_{1}, \sigma }^{{{{\dagger}}} }{c}_{{\lambda }_{2},{\sigma }^{{\prime} }}^{{{{\dagger}}} }{c}_{{\lambda }_{3},{\sigma }^{{\prime} }}{c}_{{\lambda }_{4}, \sigma }.$$

Here, *c*_*λ*,*σ*_ (${c}_{\lambda ,\sigma }^{{{{\dagger}}} }$) is the annihilation (creation) operator for an electron in the state (*λ*, *σ*), where *λ* represents the collection of all the particles’ quantum numbers but the spin. In terms of the latter, the electronic field operator is

2 $${\psi }_{\sigma }({{{{{{{\bf{r}}}}}}}})=\mathop{\sum}\limits_{\lambda }{\phi }_{\lambda, \sigma }({{{{{{{\bf{r}}}}}}}}){c}_{\lambda, \sigma },$$

where *ϕ*_*λ*,*σ*_(**r**) = *ϕ*_*λ*_(**r**)*χ*_*σ*_ is a spin-orbital state, with {*ϕ*_*λ*_(**r**)} a single-particle wavefunction basis set and *χ*_*σ*_ (*σ* ∈ {*↑*, *↓*}) a two-component spinor. In Eq. (1), we introduced the hopping matrix

3 $${t}_{{\lambda }_{1}{\lambda }_{2}}=\int\,d{{{{{{{\bf{r}}}}}}}}\,{\phi }_{{\lambda }_{1}}^{*}({{{{{{{\bf{r}}}}}}}})\left[-\frac{{\hslash }^{2}{\nabla }^{2}}{2m}+\tilde{U}({{{{{{{\bf{r}}}}}}}})\right]{\phi }_{{\lambda }_{2}}({{{{{{{\bf{r}}}}}}}}),$$

and the Coulomb tensor

4 $${V}_{{\lambda }_{1}{\lambda }_{2}{\lambda }_{3}{\lambda }_{4}}=	\frac{1}{2}\int \, d{{{{{{{\bf{r}}}}}}}} \int \,d{{{{{{{{\bf{r}}}}}}}}}^{{\prime} }\,{\phi }_{{\lambda }_{1}}^{*}({{{{{{{\bf{r}}}}}}}}){\phi }_{{\lambda }_{2}}^{*}({{{{{{{{\bf{r}}}}}}}}}^{{\prime} })\\ 	 \times W({{{{{{{\bf{r}}}}}}}}, {{{{{{{{\bf{r}}}}}}}}}^{{\prime} }){\phi }_{{\lambda }_{3}}({{{{{{{{\bf{r}}}}}}}}}^{{\prime} }){\phi }_{{\lambda }_{4}}({{{{{{{\bf{r}}}}}}}}).$$

Here, $\tilde{U}({{{{{{{\bf{r}}}}}}}})$ represents the periodic external potential generated by the ions in the lattice and is given by

5 $$\tilde{U}({{{{{{{\bf{r}}}}}}}})=\frac{{q}_{e}}{4\pi {\epsilon }_{0}}\mathop{\sum}\limits_{I}\frac{{Z}_{I}}{| {{{{{{{\bf{r}}}}}}}}-{{{{{{{{\bf{r}}}}}}}}}_{I}| },$$

where *Z*_*I*_ is the charge of the *I* − th ion and **r**_*I*_ is its position. The constants *ℏ*, *m*, *q*_*e*_ and *ϵ*_0_, are Planck’s constant, the electron mass, electron charge, and the vacuum permittivity of space, respectively. In the Coulomb tensor, $W({{{{{{{\bf{r}}}}}}}},{{{{{{{{\bf{r}}}}}}}}}^{{\prime} })=1/(4\pi {\epsilon }_{0}| {{{{{{{\bf{r}}}}}}}}-{{{{{{{{\bf{r}}}}}}}}}^{{\prime} }| )$ denotes the repulsive Coulomb interaction between electrons.

As we will discuss in the Methods section “Active space and Wannier functions", in this work we focus on the electronic degrees of freedom corresponding to the electronic bands {*ϕ*_*λ*_(**r**)} contained in a selected region around the Fermi level (the so-called active region). In this case, both the single-particle potential $\tilde{U}({{{{{{{\bf{r}}}}}}}})$ and the Coulomb interaction $W({{{{{{{\bf{r}}}}}}}},{{{{{{{{\bf{r}}}}}}}}}^{{\prime} })$ shall be modified to take into account the effects of the electrons outside the active region on the active ones. In the case of the Coulomb repulsion, this results in an effective screening of the interaction between electrons in the active space. Many sophisticated approaches have been developed to compute the exact form of $W({{{{{{{\bf{r}}}}}}}},{{{{{{{{\bf{r}}}}}}}}}^{{\prime} })$^9,10^. The aim of this work is to design an efficient quantum algorithm to reduce the complexity of simulations of materials, and we will therefore consider the simplified case of an unscreened Coulomb potential $W({{{{{{{\bf{r}}}}}}}},{{{{{{{{\bf{r}}}}}}}}}^{{\prime} })=1/(4\pi {\epsilon }_{0}| {{{{{{{\bf{r}}}}}}}}-{{{{{{{{\bf{r}}}}}}}}}^{{\prime} }| )$ only. Note that, in general, this leads to more, stronger, and longer-range interactions and, therefore, our results represent an upper bound for the quantum circuit complexities for the simulation of realistic models.

### Active space and Wannier functions

Many low-energy properties of materials are determined by a limited number of electronic degrees of freedom around the Fermi level. To identify them, we start by computing the material’s band structure within the framework of density functional theory (DFT)^70,71^. The main concepts of DFT are reviewed in Supplementary Note 2F. DFT allows one to obtain estimates of many ground state properties of a fully interacting many-body system by solving a set of auxiliary single-particle problems^72^,

6 $$\left[\frac{{\hslash }^{2}}{2m}{\nabla }^{2}+{\tilde{U}}_{{{{{{{{\rm{eff}}}}}}}}}({{{{{{{\bf{r}}}}}}}})\right]{\phi }_{i}({{{{{{{\bf{r}}}}}}}})={\epsilon }_{i}{\phi }_{i}({{{{{{{\bf{r}}}}}}}}),$$

where the terms in square brackets in the left hand side term constitute the Kohn-Sham (KS) Hamiltonian, *H*^KS^, ${\tilde{U}}_{{{{{{{{\rm{eff}}}}}}}}}({{{{{{{\bf{r}}}}}}}})=\tilde{U}({{{{{{{\bf{r}}}}}}}})+{V}_{H}({{{{{{{\bf{r}}}}}}}})+{V}_{XC}({{{{{{{\bf{r}}}}}}}})$ is the effective KS potential, *ϵ*_*i*_ are the single-particle KS eigenvalues, and {*ϕ*_*i*_(**r**)} are the KS states. Crucially, in addition to the ionic potential $\tilde{U}({{{{{{{\bf{r}}}}}}}})$ of Eq. (5), ${\tilde{U}}_{{{{{{{{\rm{eff}}}}}}}}}({{{{{{{\bf{r}}}}}}}})$ contains two additional contributions, namely the Hartree energy *V*_*H*_(**r**) and the exchange-correlation potential *V*_*XC*_(**r**), which include the contribution of electron-electron interactions. In this work, DFT calculations have been performed with Quantum Espresso^73,74^ using pseudopotentials from the ONCVPSP library^75^.

KS states can be used as the starting point to build a more accurate low-energy models of strongly correlated materials, for which standard DFT usually provide unsatisfactory results^41,43^. Such a failure is usually due to the presence of electronic bands with strong *d* or *f* character in the neighbourhood of the Fermi level. In this case, modern embedding approaches, such as density matrix embedding theory (DMET)^45,49^ and dynamical mean field theory (DMFT)^43,44^, have shown that a more faithful description of these materials can be obtained from an effective model containing a limited number of electronic bands within an active space around the Fermi level. See Fig. 1 for the case of SrVO_3_. The critical aspect of this approach is to estimate how the electrons outside the active space affect the interactions of the electrons within the active space^10^. As anticipated in the “Electronic Hamiltonian" section of Methods, in this work we assume that electrons in the active space interact via an unscreened Coulomb interactions and we leave the use of more accurate potentials to future investigations. More details about the selection of the active space and a specific example are provided in Supplementary Notes 2F 2 and 6B, respectively.

Once the active space has been identified, the KS eigenstates within the active space are used to generate maximally localized Wannier functions (MLWFs), which will serve as the single-particle wavefunction basis set for the electronic Hamiltonian of Eq. (1). The *s* − th MLWF in the unit cell indexed by the lattice vector **R** is defined by

7 $${{{{{{{{\mathcal{W}}}}}}}}}_{s}^{{{{{{{{\bf{R}}}}}}}}}({{{{{{{\bf{r}}}}}}}})=\mathop{\sum}\limits_{{{{{{{{\bf{k}}}}}}}},n}{e}^{-i{{{{{{{\bf{k}}}}}}}}\cdot {{{{{{{\bf{R}}}}}}}}}{U}_{ns}({{{{{{{\bf{k}}}}}}}}){\phi }_{{{{{{{{\bf{k}}}}}}}},n}({{{{{{{\bf{r}}}}}}}}),$$

where *ϕ*_**k,***n*_(**r**) are the eigenstates of the KS Hamiltonian in reciprocal space, i.e., *H*^KS^(**k**)*ϕ*_**k,***n*_(**r**) = *ϵ*_**k,***n*_*ϕ*_**k,***n*_(**r**), and *U*_*ns*_(**k**) is a unitary matrix whose coefficients are obtained via an iterative routine minimizing the spread of the Wannier functions^1^. In our work, MLWF are generated from the DFT output using the Wannier90 code^76^. The full workflow is discussed in more details in Supplementary Note 2F. In the Wannier basis, the electonic Hamiltonian of Eq. (1) becomes

8 $$\begin{array}{rc}{H}^{W}&=\mathop{\sum}\limits_{\sigma }\mathop{\sum}\limits_{\begin{array}{c}m,n\\ {{{{{{{{\bf{R}}}}}}}}}_{1},{{{{{{{{\bf{R}}}}}}}}}_{2}\end{array}}T{({{{{{{{{\bf{R}}}}}}}}}_{1}-{{{{{{{{\bf{R}}}}}}}}}_{2})}_{mn}{w}_{{{{{{{{{\bf{R}}}}}}}}}_{1}, m,, \sigma }^{{{{\dagger}}} }{w}_{{{{{{{{{\bf{R}}}}}}}}}_{2},n,\sigma }\\ &+\mathop{\sum}\limits_{\sigma,{\sigma }^{{\prime} }}\mathop{\sum}\limits_{\begin{array}{c}s,l,m,n\\ {{{{{{{{\bf{R}}}}}}}}}_{1},{{{{{{{{\bf{R}}}}}}}}}_{2},{{{{{{{{\bf{R}}}}}}}}}_{3},{{{{{{{{\bf{R}}}}}}}}}_{4}\end{array}}{\tilde{V}}_{slmn}^{({{{{{{{{\bf{R}}}}}}}}}_{1}{{{{{{{{\bf{R}}}}}}}}}_{2},{{{{{{{{\bf{R}}}}}}}}}_{3},{{{{{{{{\bf{R}}}}}}}}}_{4})}\\ &\times {w}_{{{{{{{{{\bf{R}}}}}}}}}_{1},s,\sigma }^{{{{\dagger}}} }{w}_{{{{{{{{{\bf{R}}}}}}}}}_{2},l,{\sigma }^{{\prime} }}^{{{{\dagger}}} }{w}_{{{{{{{{{\bf{R}}}}}}}}}_{3},m,{\sigma }^{{\prime} }}{w}_{{{{{{{{{\bf{R}}}}}}}}}_{4},n,\sigma },\end{array}$$

with *w*_**R,***m*,*σ*_ (${w}_{{{{{{{{\bf{R}}}}}}}},m,\sigma }^{{{{\dagger}}} }$) destroying (creating) an electron in the state ${{{{{{{{\mathcal{W}}}}}}}}}_{m,\sigma }^{{{{{{{{\bf{R}}}}}}}}}({{{{{{{\bf{r}}}}}}}})={{{{{{{{\mathcal{W}}}}}}}}}_{m}^{{{{{{{{\bf{R}}}}}}}}}({{{{{{{\bf{r}}}}}}}}){\chi }_{\sigma }$.

### Encoding

The goal of an efficient encoding is to map each interaction in the fermionic Hamiltonian to low-weight operators acting on a multi-qubit Hilbert space. In the MLWF basis, the electronic Hamiltonian of Eq. (8) has a local structure we can leverage in the mapping of fermionic operators into qubit operators. In particular, the hopping matrix and Coulomb tensor coefficients involving modes with lattice sites **R**_1_ and **R**_2_ decay rapidly as a function of ∣**R**_1_ − **R**_2_∣. On the other hand, interactions between modes on the same site (i.e., with the same site index **R**) are usually dense and strong. In general, regardless of the choice of the mapping, the latter interactions cannot be mapped to low-weight operators. The best strategy is then to map all the modes sharing the same lattice site index **R** to qubits using the Jordan Wigner (JW) transform and use a fermionic swap (fswap) protocol to reduce the weight of the resulting qubit operators (see Methods section “Algorithms" and Supplementary Note 4)^23,77,78^. Within this framework, fermionic creation (${c}_{i}^{{{{\dagger}}} }$) and annihilation (${c}_{i}^{{{{\dagger}}} }$) operators are mapped to strings operators acting on qubits via

9 $${c}_{i}^{{{{\dagger}}} }\leftrightarrow \left(\mathop{\prod}\limits_{j < i}{Z}_{j}\right)\frac{({X}_{i}+i{Y}_{i})}{2}$$

10 $${c}_{i}\leftrightarrow \left(\mathop{\prod}\limits_{j < i}{Z}_{j}\right)\frac{({X}_{i}-i{Y}_{i})}{2}.$$

**Fig. 2:**
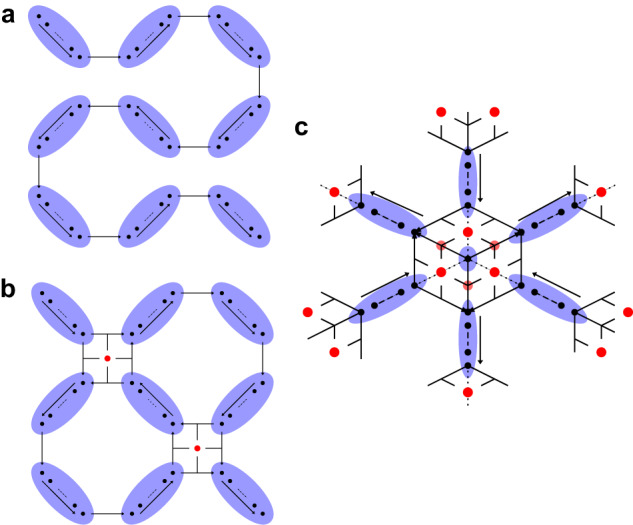
Comparison between encodings. (**a**) JW encoding, (**b**) hybrid encoding on a 2D lattice of sites, (**c**) 3D variant of the hybrid encoding. Sites (shaded ellipses) contain the modes/data qubits (black circles) with ancillary qubits (red) involved in intersite interactions. Pairs of adjacent modes in the linear ordering of modes/data qubits within each site, as well as those modes at the ends of the linear orderings between sites connected by black lines, have an efficient qubit representation. Interactions between modes, either within a site, or between neighbouring sites, are mediated by fermionic swap networks applied according to the graph topology, with fswaps applied on black edges. See Supplementary Note 3 for more details.

On the other hand, the sparse interactions between modes belonging to different lattice sites can be implemented more efficiently by mapping these modes via the compact encoding^11^. This suggests the use of a hybrid encoding in which all modes sharing the same site index **R** are associated with a collection of qubits laid out in a JW style string. In turn, each string is connected to nearest-neighbour string using the compact encoding design. See Fig. 2.

In this encoding, the recipe for mapping each fermionic term of the electronic Hamiltonian into a string of qubit operators is expressed in terms of “edge” and “vertex” operators, which are defined as

11 $${E}_{jk}\, \!\!:\!\!=-i{\gamma }_{j}{\gamma }_{k},\quad {V}_{j}\, \!\!:\!\!=-i{\gamma }_{j}{\bar{\gamma }}_{j},$$

respectively, with Majorana operators

12 $${\gamma }_{j}\, \!\!:\!\!={w}_{j}+{w}_{j}^{{{{\dagger}}} },\quad {\bar{\gamma }}_{j}\, \!\!:\!\!=({w}_{j}-{w}_{j}^{{{{\dagger}}} })/i,$$

where *j* (and *k*) is a multi-index over the site index **R**, mode index *m*, and spin index *σ*. The rules to map these operators into Pauli strings are shown in Supplementary Figures S12 and S14 for the 2D and 3D hybrid encodings, respectively. The qubit Hamiltonian can be obtained by first decomposing each term of the fermionic Hamiltonian in Eq. (8) into the operator basis of Majorana monomials,

13 $${H}_{M}\, \!\!:\!\!=\mathop{\sum}\limits_{b\in {\{0,1\}}^{2M}}{\alpha }_{b}\mathop{\prod}\limits_{j}{\gamma }_{j}^{{b}_{2j}}{\bar{\gamma }}_{j}^{{b}_{2j+1}},$$

with ∣*b*∣ ∈ {2, 4}, and by applying the encoding instructions to each monomial. Here, *M* is the total number of complex fermion modes and is given by *M* = *N*_modes/cell_*N*_cells_, with *N*_modes/cell_ and *N*_cells_ the number of modes per unit cells and unit cells in Eq. (8), respectively.

### Algorithms

The main goal of this work is to minimize the overall circuit depth for implementing one layer of both the Variational Quantum Eigensolver (VQE) and Time Dynamics Simulation (TDS) algorithms.

Given a Hamiltonian *H*_*M*_ = ∑_*k*_*h*_*k*_ and assuming that *H*_*M*_ = *H*_*A*_ + *H*_*B*_ and that an efficient quantum algorithm to prepare the ground state of *H*_*A*_ is known, the VQE algorithm allows to find an approximation to the ground state of *H*_*M*_ via a parameterized circuit ansatz consisting of a sequence of repeated layers. Within the Hamiltonian variational ansatz^19^ framework, the steps of a VQE simulations are the following:

1. Prepare the ground state of *H*_*A*_.
2. For each layer *l*, implement the operation
  $$\mathop{\prod}\limits_{k}{e}^{i{t}_{lk}{h}_{k}},$$
  for some parameters *t*_*lk*_, to produce a state $\left|\psi \right\rangle$.
3. Measure the energy of $\left|\psi \right\rangle$ with respect to *H*_*M*_.
4. Classically optimise over the parameters *t*_*lk*_ to find the ground state (or a good approximation).

On the other hand, the goal of the TDS approach is to find an approximation of the unitary ${e}^{-it{H}_{M}}$ for some time *t*. The standard method for executing this operation is by Trotterisation, wherein ${e}^{-it{H}_{M}}$ is approximated, for example, by a product of short time steps ${({\prod }_{k}{e}^{-i\delta t{h}_{k}})}^{L}$, with *δt* = *t*/*L*.

The algorithms above requires three type of operations: time-evolution step according to Majorana operators, fermionic swaps, and Givens rotations. Below we will discuss the bounds on the complexity associated with each of them. In doing that, we will assume all-to-all interactions between qubits, arbitrary 2-qubit gates having unit gate cost, and 1-qubit gates having zero gate cost. More details are provided in Supplementary Note 5C.

#### Time-evolution step

**Fig. 3:**
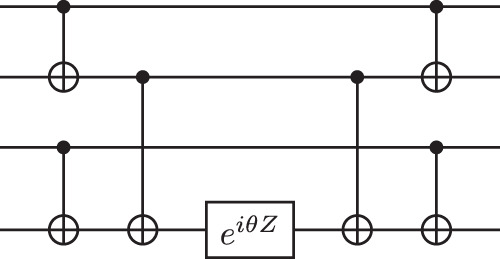
The quantum circuit implementing ${e}^{i\theta {Z}^{\otimes k}}$ in the case *k* = 4 in terms of CNOTs and single-qubit rotations. The three middle gates can be combined into one 2-qubit operation, given total cost (2-qubit gate depth) 3 in our model.

Whichever fermionic encoding is used, each term of the Hamiltonian will ultimately be represented on the quantum computer as a string of Pauli operators. As 1-qubit gates are modelled as free and all Pauli operators are equivalent up to unitary conjugation, implementing a term reduces to implementing the operation ${e}^{i\theta {Z}^{\otimes k}}$, acting on *k* ≥ 1 qubits, for arbitrary *θ*. This can be done in depth $2\lceil {\log }_{2}k\rceil -1$ via a circuit which uses a binary tree of CNOT operations to put the parity of the input state in the last qubit; performs a Z rotation on that qubit; and then performs the CNOT operations in reverse to uncompute the parity. We save depth 1 by combining the last two CNOTs and the Z rotation in one 2-qubit gate. See Fig. 3 for an example for the case *k* = 4.

#### Fermionic swaps

In the JW transform, fermionic swaps across adjacent modes are 2-qubit gates with cost 1. In the compact encoding, across most pairs of adjacent modes, the same holds. The exception is when a fermionic swap acts across different material sites, where an ancilla qubit is involved. In terms of the fermionic algebra, the fswap operator between modes *i* and *j* can be written as

14 $${{{{{{{{\rm{FSWAP}}}}}}}}}_{ij}=	 \exp \left(i\frac{\pi }{4}{V}_{i}\right)\exp \left(i\frac{\pi }{4}{V}_{j}\right)\\ 	 \times \exp \left(\frac{\pi }{4}{E}_{ij}{V}_{j}\right)\exp \left(\frac{\pi }{4}{V}_{i}{E}_{ij}\right).$$

The circuit depth may be computed by decomposing each of the terms in terms of Pauli matrices. However, for the 2D hybrid encoding, an efficient decomposition for weight 3 edge operators can be obtained, as explained in Supplementary Note 3B. This makes it possible to implement a fermionic swap by a circuit of 2-qubit depth 4. For the 3D encoding, in which the edge operators are weight 4, we will use the naive TDS decomposition.

#### Givens rotations

A Givens rotation is a unitary operation which mixes pairs of fermionic operators in the following way^79^:

15 $$\left(\begin{array}{c}{{{{{{{{\mathcal{G}}}}}}}}}_{ij}(\theta,\phi ){c}_{i}^{{{{\dagger}}} }{{{{{{{{\mathcal{G}}}}}}}}}_{ij}^{{{{\dagger}}} }(\theta,\phi )\\ {{{{{{{{\mathcal{G}}}}}}}}}_{ij}(\theta,\phi ){c}_{j}^{{{{\dagger}}} }{{{{{{{{\mathcal{G}}}}}}}}}_{ij}^{{{{\dagger}}} }(\theta,\phi )\end{array}\right)=\left(\begin{array}{cc}\cos (\theta )&-{e}^{i\phi }\sin (\theta )\\ \sin (\theta )&{e}^{i\phi }\cos (\theta )\end{array}\right)\left(\begin{array}{c}{c}_{i}^{{{{\dagger}}} }\\ {c}_{j}^{{{{\dagger}}} }\end{array}\right).$$

The cost to implement a Givens rotation can again most easily determined by writing it in terms of fermionic edge and vertex operators, which may then be translated into qubit operations using the chosen mapping:

16 $${{{{{{{{\mathcal{G}}}}}}}}}_{ij}(\theta,\phi )=\exp \left(-i\frac{\phi }{2}{V}_{j}\right)\times \exp \left(i\frac{\theta }{2}[{E}_{ij}-{V}_{i}{V}_{j}{E}_{ij}]\right),$$

In the JW transform, when acting on adjacent modes this operator corresponds to a 2-qubit operation. In the compact encoding, it is usually a 2-qubit operation, except when acting across sites. As shown in Supplementary Note 4A, in this case a Givens rotation corresponds to a 3-qubit operation in the 2D encoding, which can be implemented in 2-qubit gate depth 4, and to a 4-qubit operator in the 3D case, which can be implemented in 2-qubit gate depth 6.

### State preparation

A requisite of the VQE algorithm is preparing the ground state of *H*_*A*_, which is a part of the original material’s Hamiltonian, *H*_*M*_ = *H*_*A*_ + *H*_*B*_. The procedure to prepare a Fock state and a fermionic Gaussian state within the hybrid compact enconding and their cost are discussed in details in Supplementary Note 4B. Counting only two-qubit gates,a Fock state can be prepared with a circuit with 2-qubit gate depth 12 in the 2D case and estimated 50 in the 3D case. On the other hand, a fermionic Gaussian state requires a circuit whose overall depth is at most 2 $*$ 4⌈(*M* − 1)/2⌉ + ⌊(*M* − 1)/2⌋ ≈ 4.5*M* for the 2D hybrid encoding and 2 $*$ 6⌈(*M* − 1)/2⌉ + ⌊(*M* − 1)/2⌋ ≈ 6.5*M* for the 3D hybrid encoding, with *M* the total number of modes in the material’s Hamiltonian of Eq. (13).

### Time-evolution protocol

A common step in both the VQE and TDS algorithms is to find an efficient implementation of the time evolution according to each term *h*_*k*_ of a material’s Hamiltonian, *H*_*M*_. We assume the ordering of the product to be arbitrary. This is always the case for VQE and for first-order Trotterization. To implement this step in the most efficient way we devise a generalized protocol based on fswap networks^23^ consisting of alternating layers of the following form:

1. Fswap gates across modes that are adjacent with respect to the graph of the fermionic encoding that we are using. For example, in the JW transform, fswaps across qubits of the form (*i*, *i* + 1) would be allowed.
2. Time-evolution by all terms that are efficiently implementable given the current permutation of the graph of the fermionic encoding. Here, we consider a term to be efficiently implementable if there is a split of the modes on which it acts into pairs such that all pairs are adjacent within the encoding graph. For the fermionic encodings we use, such terms correspond to low-weight Pauli operators.

The sequence of fswap gates implemented in the first step is determined with the help of a greedy protocol. At each layer, we look at the set ${{{{{{{\mathcal{T}}}}}}}}$ of interactions *t* which have not yet been implemented, and define a distance function which measures the difficulty of implementing these interactions. Here we focus on *ℓ*_*p*_ distance functions of the form

17 $$D={\left(\mathop{\sum}\limits_{t\in {{{{{{{\mathcal{T}}}}}}}}}d{(t)}^{p}\right)}^{1/p},$$

where *p* > 0 and *d*(*t*) is the “distance” of a term *t*. This is defined as the minimum, over all splits of the modes into pairs, of the distance within the encoding graph of those modes. Empirically, we obtain good and consistent results by setting *p* = 0.5. We consider swapping each possible adjacent pair of modes in the interaction graph, and compute *D* for each choice. If there exists a pair of modes which reduces *D* upon being swapped, we then fswap the pair and mark it as used. We repeat this process until all modes have been used, or there is no choice of modes to fswap that reduces *D*.

After the fswaps layer, the sequence in which all the efficiently implementable terms of *H*_*M*_ for the current encoding graph are implemented is determined by the solution of a graph colouring problem. The vertices of the graph are terms that should be implemented in the current layer and two vertices are connected if they can be implemented simultaneously. Here we take the simple view that two terms can be implemented simultaneously if they act on disjoint sets of qubits. Then the minimal number of colours required to colour this graph such that no two adjacent vertices have the same colour is the same as the minimal number of sublayers required to implement all the terms. To efficiently determine an upper bound on the minimal number of layers we use a greedy colouring algorithm with the DSATUR heuristic as implemented in the NetworkX package^80^.

As an additional optimisation, we implement as the first step of our protocol the fswap network of Kivlichan et al.^23^. This allows us to implement all quadratic terms using *M* layers of fswaps, for a system with *M* modes. During this process, we can also implement some other terms, if they happen to become efficiently available. As we expect the overall complexity to be significantly greater than *M*, this is a lower-order cost that can reduce the number of terms used substantially.

Our protocol is discussed in more detail in Supplementary Notes 4C and 5.

### Measurement schemes

We consider three different measurement strategies, focusing in particular on the measurement of energy in VQE circuits. We assume that the operators to be measured are expressed in the JW transform. This also allows us to handle the case of the hybrid encoding with nearest-neighbour interactions between sites, though terms acting on next-nearest neighbours and beyond do not necessarily have this property. Then, a quadratic fermion term corresponds to Pauli strings of the form *AZZ* … *ZB*, where *A*, *B* ∈ {*X*, *Y*}, and the quartic case is either a product of two such strings on disjoint sets of qubits, or the product of a quadratic string and a *Z* operator elsewhere.

To minimize the number of measurement rounds, we consider three strategies to decompose a set of Majorana operators into groups that can be measured simultaneously:

- (QWC) Measuring qubitwise commuting terms simultaneously. These are Pauli terms which commute when restricted to individual qubits. This family of measurement strategies is easy to implement by measuring each qubit in the correct X/Y/Z basis, so requires only additional single-qubit gates^16,17^.
- (NC) Measuring a family of non-crossing terms simultaneously. A pair of distinct quadratic Majorana operators acting on modes *i*≤*j* and *k*≤*l* is non-crossing if either:
  1. *j* < *k*, or *l* < *i*, or *i* < *k*≤*l* < *j*, or *k* < *i*≤*j* < *l*;
  2. or *i* = *k*, *j* = *l*, and the endpoints of the two operators are picked from the set {*XX*, *YY*}, or the set {*XY*, *YX*}.
  A set *T* of Majorana operators is non-crossing if there exists a set *S* of non-crossing quadratic Majorana operators such that all operators in *T* are equal to a product of terms from *S*. Operators in such a set can be measured simultaneously with a simple protocol, discussed in detail in Supplementary Note 4D.
- (COM) Measuring a family of commuting operators simultaneously. This is the approach resulting in the fewest measurement rounds we considered. However, the quantum circuits required to simultaneously diagonalise a set of measurement operators may be relatively difficult to implement (requiring depth Θ(*M*)).

In all the three cases above, the decomposition of Majorana operators into groups can be found by exploiting a mapping to a graph colouring problem. Analytical lower and upper bounds for the various strategies are summarised in Table 2, and derived in Supplementary Note 4D.

**Table 2:** Summary of the lower and upper bounds on the number of measurement rounds required

Strategy	Lower bound	Upper bound
QWC	$\frac{{M}^{4}}{16}$	$\frac{{M}^{4}}{3}$
NC	$\frac{2{M}^{2}}{3}$	$\frac{7{M}^{2}}{3}$
COM	$\frac{2{M}^{2}}{3}$	$\frac{5{M}^{2}}{3}$

### Supplementary information

Supplementary Information

Peer Review File

## Data Availability

Data supporting the figures and tables in this manuscript, including data for the Hamiltonians, fermionic encodings and quantum circuits presented, is available at^81^.

## Code availability

DFT data have been obtained using Quantum Espresso^73,74^ using pseudopotentials from the ONCVPSP library^75^. Maximally localized Wannier functions have been generated via the Wannier90 code^76^.
