# Supplementary material for: Towards near-term quantum simulation of materials
Source: Nat Commun. 2024 Jan 24;15:211. doi: 10.1038/s41467-023-43479-6 (PMC10808561; doi:10.1038/s41467-023-43479-6)
Supplement: Supplementary file 1 — Supplementary Information [file 41467_2023_43479_MOESM1_ESM.pdf]

Supplementary Information
Towards near-term quantum simulation of materials

Laura Clinton, Toby Cubitt, Brian Flynn, Filippo Maria Gambetta, Joel Klassen,*
Ashley Montanaro, Raul A. Santos,† Stephen Piddock,‡ and Evan Sheridan
Phasecraft Ltd.
(Dated: November 7, 2023)

Contents

1. Design strategy 3

2. Effective description of the Hamiltonian 4
   A. General characteristics of fermion Hamiltonians 6
      1. Structure of two and four fermion integrals 6
      2. Cauchy-Schwarz inequality for the Coulomb tensor 6
   B. Momentum-space single-particle bases 7
      1. Plane wave basis 8
      2. Bloch wave basis 9
   C. Real-space single-particle basis: Wannier functions 10
      1. Material real-space motif 12
   D. General constraints and symmetry properties of the fermion integrals 13
      1. General constraints and symmetry properties of the fermion integrals for general real single-particle wavefunctions 14
      2. Inversion symmetry 15
      3. Time-reversal symmetry 16
   E. Crystal symmetries 17
   F. Using DFT to choose degrees of freedom 20
      1. Kohn-Sham eigenstates in the plane-wave basis 21
      2. Truncation into an active space 23
   G. Summary 25

3. Qubit representation 26
   A. Bloch basis mapping 27
   B. Wannier basis mapping 27
      1. Stabilizers 29
      2. 3D Layout 29

4. VQE and TDS algorithms 30
   A. Gate decompositions of operations 33
   B. State preparation 35
      1. Fock states 35
      2. Fermionic Gaussian states 36
   C. Time-evolution according to terms in materials Hamiltonians 37
   D. Measurements 38
      1. Previous work 40
      2. Analytical lower bounds 41

---

* joel@phasecraft.io
† raul@phasecraft.io
‡ Currently: Royal Holloway, University of London

3. Analytical upper bounds 41
4. Quartic terms that act on three modes 43
E. Summary

5. Circuit compiler design 44
A. Fswap network implementation details 45
1. Chain fswap network 45
2. Distance minimising fswap network 46
3. Composite fswap network 47
B. Compiling unit cells of translationally invariant Hamiltonians 48
1. Compiling beyond-nearest-neighbour terms 49
C. Circuit costing 50

6. Results 50
A. Circuit analysis of a simple example 51
1. One unit cell 51
2. Fermionic swap networks 53
3. Many unit cells 54
B. Full-stack analysis: Strontium vanadate 54
1. Computation of hopping matrix and Coulomb tensor coefficients 57
C. Circuit analysis 63
1. Strontium vanadate 63
2. Trotter error 64
3. Further materials 66
4. Hardware requirements 68
5. Measurement rounds 69

7. Outlook 69

A. Baseline for qubit requirements and gate depth of materials 71

B. Exponentially localized Wannier functions 73

C. Optimality of the Jordan-Wigner transform and fswap networks 75

D. Hamiltonian coefficients pipeline 76
1. Full Hamiltonian of an electronic system on a lattice 76
2. Motif Hamiltonian 78
a. Hopping matrix coefficients 79
b. Coulomb tensor coefficients 80
c. Single-index HM and CT coefficients and Majorana Hamiltonian 83

E. Materials analysis 83
1. Material properties 83
2. Circuit depth 83
3. Measurement layers 84

Supplementary Notes 1 – Design strategy

The noisy intermediate-scale quantum (NISQ) era is characterised by quantum computers (QCs) operating without fault tolerance, so the depth of implementable quantum circuits is fixed by the error level present in the available device. Therefore the construction of compact circuits for simulation is crucial, as it can enable meaningful results (i.e., circuits where the accumulated error can be mitigated), as opposed to the random noise otherwise likely. Such constructions rely on two critical components: the physical instance being simulated, and an efficient decomposition of the physical information into layers of quantum gates. Our design strategy tackles these aspects in tandem.

The first step is to identify the relevant degrees of freedom (DoF) of the phenomena under investigation. This is not a sharp (or even well defined) procedure, but instead depends on the nature of the question being asked. For a given material, for example, studying electric transport at low temperatures involves different physical processes than the melting behaviour at high temperature. At a high level, this approach consists of choosing an *active space*, commonly discussed in chemistry and materials science [1]. This active space can be seen as a distillation of the relevant DoF at a certain energy scale. Once the relevant DoF in the active space have been identified, their dynamics are constructed: these dynamics are governed by an *effective Hamiltonian* which describes their interactions.

Once this effective Hamiltonian has been obtained, a map between the physical and the logical DoF is required. Abstractly, this procedure maps interactions between the original DoF to qubit operations. In particular for fermions, the interplay between the structure of the Hamiltonian interactions and the fermionic encoding plays an important role in the ability to create compact circuits. At the end of this step a collection of Pauli operators is derived, comprising the qubit Hamiltonian H_Q. Following this, the protocol implementing all the terms in the qubit Hamiltonian is computed. Here, the general approach that we use has the same structure as Trotterization of the evolution operator $\exp(iH_Q t)$. The structure of this step is indicative of the cost of finding a ground state via a Variational Quantum Eigensolver (VQE) approach (in particular under the Hamiltonian variational ansatz [2]), or Time Dynamics Simulation (TDS). This produces the circuit for a single Trotter step (or a single layer of VQE). Finally, we determine the measurement protocol that produces the minimum measurement overhead. Clearly, the decisions at each stage will have an effect on final cost of implementing all the qubit operators present in H_Q through a quantum circuit. Hence we adopt a multi-tiered strategy for minimizing the cost of the quantum circuit, that we describe below in the context of materials simulations.

For the physics-based construction of the Hamiltonian of a material we adopt the Born-Oppenheimer approximation [3–5], and concentrate on the quantum description of the electron DoF, including the nuclei as a classical background potential. While this approach is general enough to be used in chemistry and materials science, we note that including the quantum mechanical DoF of the nuclei is also possible within this framework. The existence of the periodic ionic potential is a distinctive feature of materials, which sets them apart from molecules. We use density functional theory (DFT) for a low level exploration of the active space of materials, defined as an energy window around the Fermi level. Using this window containing the relevant DoF, we construct an effective Hamiltonian by classically computing its matrix elements.[1]

We study two natural single-particle bases for the electrons: the Bloch basis, and the Wannier basis [4, 5]. The bands kept in the active space become modes in the unit cell, and the size of the material determines the number of unit cells. Due to the locality in real space achieved by the Wannier basis, a bespoke selection of bands allows us to construct a local Hamiltonian in real space, where the Coulomb interactions are localised, and the hopping range of electrons between unit cells does not scale with the system size. This local Hamiltonian defines a *motif* that can be used to tile a system of any size without increasing the depth, i.e. the number of layers, each containing many quantum gates.

---

[1] The problem of properly computing these matrix elements is ambiguous, for at least two reasons. An unavoidable problem that appears once an active space is used is that the electrons outside the active space renormalise the interactions that the electrons in the active space feel with the nucleus. To fully characterise that renormalisation, the solution of the many-body interacting problem has to be found, which is what we are trying to do in the first place. The second reason is that any realisation of DFT is an approximation in itself, as the exchange correlation functional is unknown. Both problems are known in the community, and are handled in a plethora of different ways see, e.g., [6–9].

To leverage the locality of the obtained fermion Hamiltonians, we introduce a novel fermionic encoding that uses the local structure of Coulomb interactions and hopping terms by hybridizing two existing encodings: the Jordan Wigner (JW) transform within a unit cell (where the majority of the electron-electron interactions are present), and the compact encoding [10] between unit cells, where fewer interactions have to be considered, following from the locality of the fermion Hamiltonian. This comes at the cost of introducing further ancillary qubits. In order to deal with the existence of large weight operators along the JW line, we introduce an algorithm based on the use of fermionic swap operations (fswaps) [11]. These operations can bring operators closer together along the JW line and minimise their weight, by relabelling the fermionic modes. This construction can be expanded to span structures other than single unit cells, depending on the connectivity graph of the Hamiltonian in question.

We invoke a cost model where all-to-all qubit interactions are available, arbitrary 2-qubit gates have cost 1 each, and 1-qubit gates are free (i.e. negligible). We perform an in-depth analysis of the cost of implementing the most general terms allowed by symmetry, and the cost of performing fswaps to bring modes into an adjacent ordering within the JW string. Finally, we analyse the cost of executing a full VQE layer of the Hamiltonian $H_Q = \sum_k H_k$ given by $\prod_k e^{i\theta_k H_k}$, where $\{ \theta_k \}$ is a set of variational parameters. Here H_Q is obtained from a fermion representation in the Wannier bases, with different fermionic encodings. Additionally, we calculate the classical measurement overhead, i.e., we determine how many times a given circuit must be repeated to estimate an observable of interest, according to a series of measurement strategies.

We develop a tool to perform the necessary decomposition into layers of simultaneously implementable terms from a given Hamiltonian. This allows us to study different materials and model Hamiltonians, to understand their cost complexity, and to find a full decomposition into quantum circuits. The summary of the design strategy to optimise over circuit cost is shown in Supplementary Figure 1.

A self-contained exposition of the physics behind the construction of Hamiltonians is presented in Supplementary Note 2. The role of symmetries, Wannier and Bloch functions, and efficient techniques to construct the matrix elements are discussed there.

In Supplementary Note 3 we introduce a hybrid fermionic encoding, and discuss its use in the context of materials simulation, where it represents an efficient fermion-to-qubit mapping. In Supplementary Note 4 we first concentrate on the quantum algorithm (VQE) itself and then discuss the decomposition of operators in terms of gates, initial state preparation, time evolution according to the material's Hamiltonian, and measurement protocols. Combining these ideas, in Supplementary Note 5 we discuss the design of our circuit compiler, which we go on to use in Supplementary Note 6 to analyse the cost of running a single layer of VQE or a single Trotter step for TDS, in examples of increasing complexity.

Supplementary Notes 2 – Effective description of the Hamiltonian

The full simulation of a physical system comprises infinitely many DoF, which makes it infeasible. This has never been a problem in domains where the relevant energy scale of the problem is restricted to a finite range. In this situation, the DoF at that scale are the ones that mostly contribute to the physical phenomena in question. For everyday applications, where most of the processes are controlled by the behaviour of the electron DoF in atoms, the Hamiltonian[2]

$$H = \sum_\sigma \int d\mathbf{r} \left[\frac{\hbar^2}{2m} |\nabla \hat{\psi}_\sigma(\mathbf{r})|^2 + \tilde{U}(\mathbf{r}) \hat{\psi}^\dagger_\sigma(\mathbf{r}) \hat{\psi}_\sigma(\mathbf{r}) \right] + \frac{1}{2} \sum_{\sigma,\sigma'} \int d\mathbf{r} \int d\mathbf{r}' \hat{\psi}^\dagger_\sigma(\mathbf{r}) \hat{\psi}^\dagger_{\sigma'}(\mathbf{r}') V(|\mathbf{r}-\mathbf{r}'|) \hat{\psi}_{\sigma'}(\mathbf{r}') \hat{\psi}_\sigma(\mathbf{r}) \quad (1)$$

[2] The full Hamiltonian includes the lattice ions. The mass of the ions is much larger than the mass of the electrons, so a good approximation is to consider the ions frozen. The lattice of frozen ions then acts as an external potential on the electrons. This approach, known as the Born-Oppenheimer approximation [3], has found success outside typical everyday experimental phenomena, from the prediction of the optimal structural configuration of the rare earth hydrides used in high pressure room temperature superconductors [12] to understanding the role of Li-ion migration in conventional batteries [13].

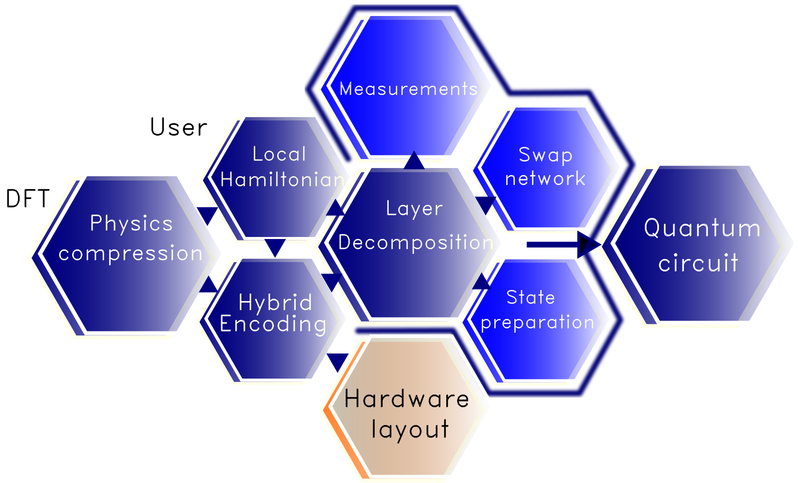

Supplementary Figure 1. Summary of the strategy developed in this work to minimise circuit depth in the simulation of materials. Starting from a low level calculation based on DFT, we perform a compression of the physical information into relevant degrees of freedom. The locality of the interactions and the hardware layout determine the structure of the hybrid encoding. In order to minimise the circuit depth, the layer decomposition module determines the appropriate fswap networks, state preparation layers, and a measurement protocol. These elements constitute the quantum circuit that implements a layer of either VQE or TDS.

describes all the possible non-relativistic physical systems in the absence of external magnetic fields. Here, $\hat{\psi}^\dagger_\sigma(\mathbf{r})$ $(\hat{\psi}_\sigma(\mathbf{r}))$ is an operator that creates (destroys) an electron at position $\mathbf{r}$ of spin σ. For the sake of notational simplicity, in what follows we will omit the hat when denoting operators. In Eq. (1), $V(|\mathbf{r}-\mathbf{r}'|)$ is the distance-dependent repulsive potential between electrons. To derive explicit formulas, in what follows we will consider the screened Coulomb potential $V(|\mathbf{r}-\mathbf{r}'|) = q_e(4\pi\epsilon_0)^{-1}e^{-\mu|\mathbf{r}-\mathbf{r}'|}/|\mathbf{r}-\mathbf{r}'|$, with μ being the inverse screening length, but our results hold for any positive definite, central, and spin-independent potential. The constants $\hbar$, m, q_e and ϵ_0, are Planck's constant, the electron mass, electron charge, and the vacuum permittivity of space respectively.

The abundant phenomena we observe in nature day-to-day is due, in part, to the structure of the potential $\tilde{U}(\mathbf{r})$, which characterises the Coulomb potential produced by the positively charged nucleus of the atoms in the system.

In materials, the external potential created by the ions in the lattice heavily influences the electrons. Assuming a block of material is invariant under lattice translations $\mathbf{R}_n$, the external potential satisfies $\tilde{U}(\mathbf{r}+\mathbf{R}_n) = \tilde{U}(\mathbf{r})$. A usual way of parameterising it is

$$\tilde{U}(\mathbf{r}) = \frac{q_e}{4\pi\epsilon_0}\sum_I \frac{Z_I}{|\mathbf{r}-\mathbf{r}_I|}, \tag{2}$$

where Z_I is the charge of the ions and $\mathbf{r}_I$ is their position.

Starting from this scenario, in this section we discuss how the reduction of the Hamiltonian in Eq. (1) (which from now on we assume to represent a block of material, and thus lattice periodic) is performed, leading to a Hamiltonian over finitely many degrees of freedom and with an interaction structure that makes it amenable to simulation using shorter quantum circuits. As quantum simulation brings different communities together, we present a self contained discussion, revising familiar concepts to condensed matter physicists and materials scientists, but which may be not completely familiar to other communities.

A. General characteristics of fermion Hamiltonians

1. *Structure of two and four fermion integrals*

In this section we examine the general properties of the two- and four-fermion integrals occurring in the Hamiltonian of Eq. (1). We first expand the electron operator $\psi_\sigma(\mathbf{r})$ in a basis of single-particle wavefunctions $\{\,\phi_\lambda(\mathbf{r})\,\}$ as

$$\psi_\sigma(\mathbf{r}) = \sum_\lambda \phi_\lambda(\mathbf{r}) c_{\lambda,\sigma}, \tag{3}$$

where λ represents the collection of all the particles' quantum numbers but the spin[3], and $c_{\lambda,\sigma}$ ($c^\dagger_{\lambda,\sigma}$) is the annihilation (creation) operator for a fermion in the state (λ,σ). In terms of the latter, Eq. (1) becomes

$$H = \sum_\sigma \sum_{\lambda_1,\lambda_2} t_{\lambda_1\lambda_2} c^\dagger_{\lambda_1,\sigma} c_{\lambda_2,\sigma} + \sum_{\sigma,\sigma'} \sum_{\lambda_1,\lambda_2,\lambda_3,\lambda_4} V_{\lambda_1\lambda_2\lambda_3\lambda_4} c^\dagger_{\lambda_1,\sigma} c^\dagger_{\lambda_2,\sigma'} c_{\lambda_3,\sigma'} c_{\lambda_4,\sigma}. \tag{4}$$

Here, the *hopping matrix* is defined as

$$t_{\lambda_1\lambda_2} = \int d\mathbf{r}\ \phi^*_{\lambda_1}(\mathbf{r}) \left[-\frac{\hbar^2\nabla^2}{2m} + \tilde{U}(\mathbf{r}) \right] \phi_{\lambda_2}(\mathbf{r}), \tag{5}$$

while the *Coulomb tensor* is

$$V_{\lambda_1\lambda_2\lambda_3\lambda_4} = \frac{1}{2} \int d\mathbf{r} \int d\mathbf{r}'\ \phi^*_{\lambda_1}(\mathbf{r}) \phi^*_{\lambda_2}(\mathbf{r}') V(|\mathbf{r}-\mathbf{r}'|) \phi_{\lambda_3}(\mathbf{r}') \phi_{\lambda_4}(\mathbf{r}). \tag{6}$$

In particular, both the hopping matrix and the Coulomb tensor are Hermitian, i.e., $t_{\lambda_1\lambda_2} = t^*_{\lambda_2\lambda_1}$ and $V_{\lambda_1\lambda_2\lambda_3\lambda_4} = V^*_{\lambda_4\lambda_3\lambda_2\lambda_1}$, where a^* denotes the complex conjugate of a. From Eq. (6) it immediately follows that the Coulomb tensor obeys the index-swap symmetry $V_{\lambda_1\lambda_2\lambda_3\lambda_4} = V_{\lambda_2\lambda_1\lambda_4\lambda_3}$.

2. *Cauchy-Schwarz inequality for the Coulomb tensor*

Exploiting the fact that $V(|\mathbf{r}-\mathbf{r}'|)$ is a real positive definite function, one can rewrite Eq. (6) in terms of an inner product. The latter can be defined in two possible ways. The first one is

$$V_{\lambda_1\lambda_2\lambda_3\lambda_4} \equiv \langle \rho_{\lambda_1\lambda_2}, \rho_{\lambda_4\lambda_3} \rangle_1 = \frac{1}{2} \int d\mathbf{r} \int d\mathbf{r}'\ V(|\mathbf{r}-\mathbf{r}'|) \rho^*_{\lambda_1\lambda_2}(\mathbf{r},\mathbf{r}') \rho_{\lambda_4\lambda_3}(\mathbf{r},\mathbf{r}'), \tag{7}$$

where $\rho_{\lambda_i\lambda_j}(\mathbf{r},\mathbf{r}') \equiv \phi_{\lambda_i}(\mathbf{r})\phi_{\lambda_j}(\mathbf{r}')$. Hence, the following inequality between the elements of the Coulomb tensor follows from the Cauchy-Schwarz inequality applied to Eq. (7)

$$|V_{\lambda_1\lambda_2\lambda_3\lambda_4}|^2 \leq |V_{\lambda_1\lambda_2\lambda_2\lambda_1} V_{\lambda_4\lambda_3\lambda_3\lambda_4}|. \tag{8}$$

On the other hand, another well-defined inner product can be introduced as

$$V_{\lambda_1\lambda_2\lambda_3\lambda_4} \equiv \langle \rho'_{\lambda_1\lambda_4}, \rho'_{\lambda_2\lambda_3} \rangle_2 = \frac{1}{2} \int d\mathbf{r} \int d\mathbf{r}'\ V(|\mathbf{r}-\mathbf{r}'|) \rho'^*_{\lambda_1\lambda_4}(\mathbf{r}) \rho'_{\lambda_3\lambda_2}(\mathbf{r}'), \tag{9}$$

where $\rho'_{\lambda_i\lambda_j}(\mathbf{r}) \equiv \phi_{\lambda_i}(\mathbf{r})\phi^*_{\lambda_j}(\mathbf{r})$. Similarly to the previous case, the Cauchy-Schwarz inequality associated with this inner product implies the following relation between the Coulomb tensor elements

$$|V_{\lambda_1\lambda_2\lambda_3\lambda_4}|^2 \leq |V_{\lambda_1\lambda_4\lambda_1\lambda_4} V_{\lambda_3\lambda_2\lambda_3\lambda_2}|. \tag{10}$$

Eq. (8) and Eq. (10) can be exploited to obtain bounds on the Coulomb tensor coefficients, allowing one to truncate the elements smaller than a given threshold without having to directly compute them. This is usually very useful in reducing the classical computation needed to determine a quantum Hamiltonian.

---

[3] In systems with strong spin-orbit coupling, a more general single-particle spinor wavefunction $\phi_{\lambda,\sigma}(\mathbf{r})$ is possible. We do not consider this case here.

B. Momentum-space single-particle bases

In this and the following sections we will introduce some of the most common single-particle bases to study condensed matter systems. As we will be discussing different bases for the same Hamiltonian, to avoid confusion, especially when these Hamiltonians are mapped into qubit operators, we will explicitly add a superscript to a Hamiltonian in a particular basis, each of which will be defined below. We will have:

- H^P: Hamiltonian Eq. (1) in the plane wave single-particle electron basis. The second quantized creation (annihilation) operators of momentum k and spin σ in this context are denoted by $c^\dagger_{k,\sigma}$ ($c_{k,\sigma}$). Choosing a lattice of discrete translations, the total momentum k can always be decomposed in the lattice momentum $\mathbf{k}$ and reciprocal lattice vector $\mathbf{G}$ as $k = \mathbf{k} + \mathbf{G}$.
- H^B: Hamiltonian Eq. (1) in the Bloch-wave single-particle electron basis. The creation (annihilation) operators are $f^\dagger_{\mathbf{k},n,\sigma}$ ($f_{\mathbf{k},n,\sigma}$), with $\mathbf{k}$ the lattice momentum, n the band index and σ the spin.
- H^W: Hamiltonian Eq. (1) in the Wannier single-particle electron basis. The creation (annihilation) operators of band n and spin σ are $w^\dagger_{\mathbf{R},n,\sigma}$ ($w_{\mathbf{R},n,\sigma}$), where $\mathbf{R}$ is the lattice vector.

All single-particle basis operators (called generically A_j) satisfy the equal-time anti-commutation relations $\{A_i, A^\dagger_j\} = \delta_{ij}$.

We begin with momentum-space bases, which fully exploit the translational invariance of crystalline solids. For more details see e.g., Refs. [5, 14]. In a material, atoms are arranged in a periodic structure (see Supplementary Figure 2) which is spanned by the lattice vectors $\mathbf{R}_a$, $a = 1, 2, 3$. The lattice points correspond to

$$\mathbf{R} = n_1\mathbf{R}_1 + n_2\mathbf{R}_2 + n_3\mathbf{R}_3, \tag{11}$$

where $n_a \in \mathbb{Z}$. The lattice vector $\mathbf{R}_a$ has length R_a. Translations $T_\mathbf{R}$ along these lattice vectors leave the Hamiltonian H invariant (assuming periodic boundary conditions). Consequently, we can block-diagonalize the Hamiltonian, and each block will correspond to a different eigenvalue of the translation operator. The Bloch Theorem allows us to find the simultaneous eigenfunctions of $T_\mathbf{R}$ and H [5, 14].

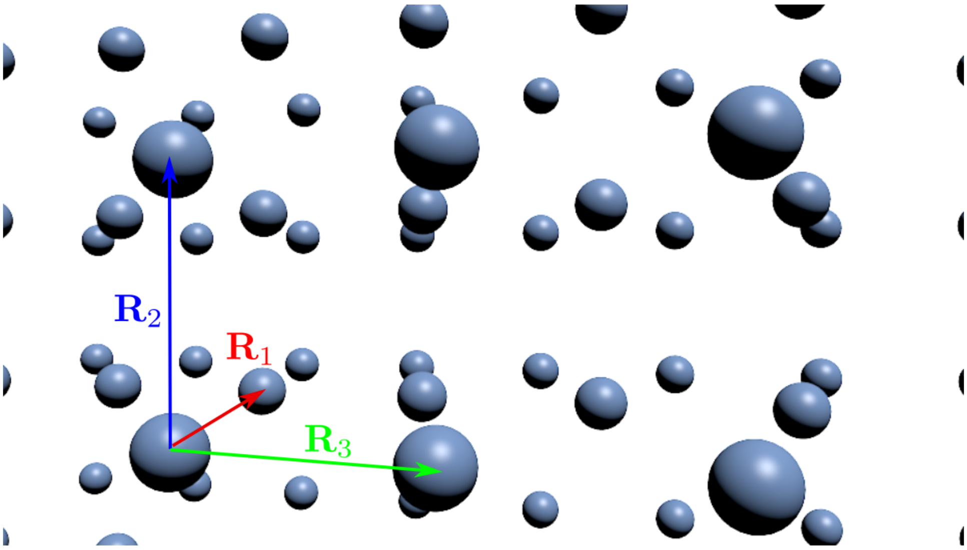

Supplementary Figure 2. Simple Orthorhombic Bravais lattice, with its lattice vectors.

The translation operator $T_\mathbf{R}$ forms an Abelian group, satisfying $T_\mathbf{R}T_{\mathbf{R}'} = T_{\mathbf{R}+\mathbf{R}'}$, with $T_\mathbf{0} = 1$. As T should be represented by a unitary operator, in its diagonal basis it acts on the single-particle

wavefunctions as

$$T_{\mathbf{R}}\phi(\mathbf{r}) \equiv \phi(\mathbf{r}+\mathbf{R}) = e^{i\mathbf{k}\cdot\mathbf{R}}\phi(\mathbf{r}), \tag{12}$$

where the vector $\mathbf{k}$ is called the crystal momentum[4]. In a periodic system with linear size $L_a = N_a R_a$ in each lattice vector direction, the periodic boundary conditions (Born-von Karman boundary conditions) $\psi(\mathbf{r}+N_a\mathbf{R}_a) = \psi(\mathbf{r})$ imply the quantization of the crystal momentum as

$$\mathbf{k} = \frac{n_1}{N_1}\mathbf{b}_1 + \frac{n_2}{N_2}\mathbf{b}_2 + \frac{n_3}{N_3}\mathbf{b}_3, \tag{13}$$

where the reciprocal lattice vectors $\mathbf{b}_j$ satisfy $\mathbf{b}_i \cdot \mathbf{R}_j = 2\pi\delta_{ij}$ and $n_a \in [0, N_a - 1]$. The eigenstates of the translation operator $T_{\mathbf{R}}$ can then be labelled by the triplet n_1, n_2, n_3, corresponding to a total of $N = N_1N_2N_3$ states. The total volume of the crystal is $V_c = N\Omega$, with $\Omega = |\mathbf{R}_1 \cdot (\mathbf{R}_2 \times \mathbf{R}_3)|$ the volume of the unit cell. The relation between direct and reciprocal lattice is shown in Supplementary Figure 3.

Using Eq. (12), we can define

$$\phi_{\mathbf{k}}(\mathbf{r}) = e^{i\mathbf{k}\cdot\mathbf{r}}[e^{-i\mathbf{k}\cdot\mathbf{r}}\phi_{\mathbf{k}}(\mathbf{r})] = e^{i\mathbf{k}\cdot\mathbf{r}}u_{\mathbf{k}}(\mathbf{r}), \tag{14}$$

with $u_{\mathbf{k}}(\mathbf{r}+\mathbf{R}) = u_{\mathbf{k}}(\mathbf{r})$ a lattice periodic function. The single electron wavefunction $\phi_{\mathbf{k}}(\mathbf{r}) = e^{i\mathbf{k}\cdot\mathbf{r}}u_{\mathbf{k}}(\mathbf{r})$ is called a Bloch wave [4, 5]. Since $u_{\mathbf{k}}(\mathbf{r})$ is a lattice periodic function, it may be useful to expand it in Fourier series as

$$u_{\mathbf{k}}(\mathbf{r}) = \sum_{\mathbf{G}} u_{\mathbf{k},\mathbf{G}} e^{i\mathbf{G}\cdot\mathbf{r}}, \tag{15}$$

where $\mathbf{G}$ is a reciprocal lattice vector $\mathbf{G} = m_1\mathbf{b}_1 + m_2\mathbf{b}_2 + m_3\mathbf{b}_3$, with $m_i \in \mathbb{Z}$, and to write the Bloch wave as

$$\phi_{\mathbf{k}}(\mathbf{r}) = \sum_{\mathbf{G}} u_{\mathbf{k},\mathbf{G}} e^{i(\mathbf{k}+\mathbf{G})\cdot\mathbf{r}}. \tag{16}$$

1. *Plane wave basis*

A particularly simple choice for the functions $u_{\mathbf{k}}(\mathbf{r})$ is $u_{\mathbf{k}}(\mathbf{r}) = N^{-1}\sum_R \delta(\mathbf{r}-\mathbf{R})$, with $\delta(\mathbf{R})$ the Dirac delta function. This choice implies that all the Fourier coefficients $u_{\mathbf{k},\mathbf{G}}$ in Eq. (16) are set to 1 and, therefore, it corresponds to expanding the Bloch wave $\phi_{\mathbf{k}}(\mathbf{r})$ in the plane wave basis $\{\, e^{i(\mathbf{k}+\mathbf{G})\cdot\mathbf{r}} \,\}$. An advantage of this basis is that plane waves for different momenta are orthogonal. The electron operator takes the form

$$\psi_\sigma(\mathbf{r}) = \frac{1}{\sqrt{V_c}} \sum_{\mathbf{k},\mathbf{G}} e^{i(\mathbf{k}+\mathbf{G})\cdot\mathbf{r}} c_{\mathbf{k}+\mathbf{G},\sigma}, \tag{17}$$

where $c_{\mathbf{k}+\mathbf{G},\sigma}$ ($c^\dagger_{\mathbf{k}+\mathbf{G},\sigma}$) is the annihilation (creation) operator of an electron with momentum $\mathbf{k}+\mathbf{G}$ and spin σ. In the plane wave basis, the Hamiltonian of Eq. (4) becomes

$$H^P = \sum_{\mathbf{k},\mathbf{G},\mathbf{G}',\sigma} h_{\mathbf{k},\mathbf{G}-\mathbf{G}'} c^\dagger_{\mathbf{k}+\mathbf{G},\sigma} c_{\mathbf{k}+\mathbf{G}',\sigma} + \sum_{\substack{\mathbf{k},\mathbf{k}',p \\ \mathbf{G},\mathbf{G}',\sigma,\sigma'}} V_p c^\dagger_{\mathbf{k}+\mathbf{G}+p,\sigma} c^\dagger_{\mathbf{k}'+\mathbf{G}'-p,\sigma'} c_{\mathbf{k}'+\mathbf{G}',\sigma'} c_{\mathbf{k}+\mathbf{G},\sigma}, \tag{18}$$

where $h_{\mathbf{k},\mathbf{G}-\mathbf{G}'} = \left[\frac{|\hbar(\mathbf{k}+\mathbf{G})|^2}{2m}\delta_{\mathbf{G},\mathbf{G}'} + U_{\mathbf{G}-\mathbf{G}'}\right]$, $V_p = 1/(2V_c)\int d\mathbf{r} e^{-ip\cdot\mathbf{r}} V(|\mathbf{r}|)$, and $p = \mathbf{p}+\mathbf{P}$ is the total momentum, with $\mathbf{p} = \mathbf{k}+\mathbf{k}'$ and $\mathbf{P} = \mathbf{G}+\mathbf{G}'$. $U_{\mathbf{G}}$ is the Fourier component of the external lattice potential at reciprocal lattice vector $\mathbf{G}$

$$\tilde{U}(\mathbf{r}) = \sum_{\mathbf{G}} e^{i\mathbf{G}\cdot\mathbf{r}} U_{\mathbf{G}} \quad \rightarrow \quad U_{\mathbf{G}} = \frac{1}{\Omega}\int_{uc} d\mathbf{r} e^{-i\mathbf{G}\cdot\mathbf{r}} \tilde{U}(\mathbf{r}), \tag{19}$$

[4] Note that the crystal momentum does **not** coincide with the momentum of the particle. The latter can be obtained from its group velocity according to $\mathbf{v}_n(\mathbf{k}) = \frac{1}{\hbar}\nabla_{\mathbf{k}} E_n(\mathbf{k})$, where $E_n(\mathbf{k})$ is the energy of $n-$th band.

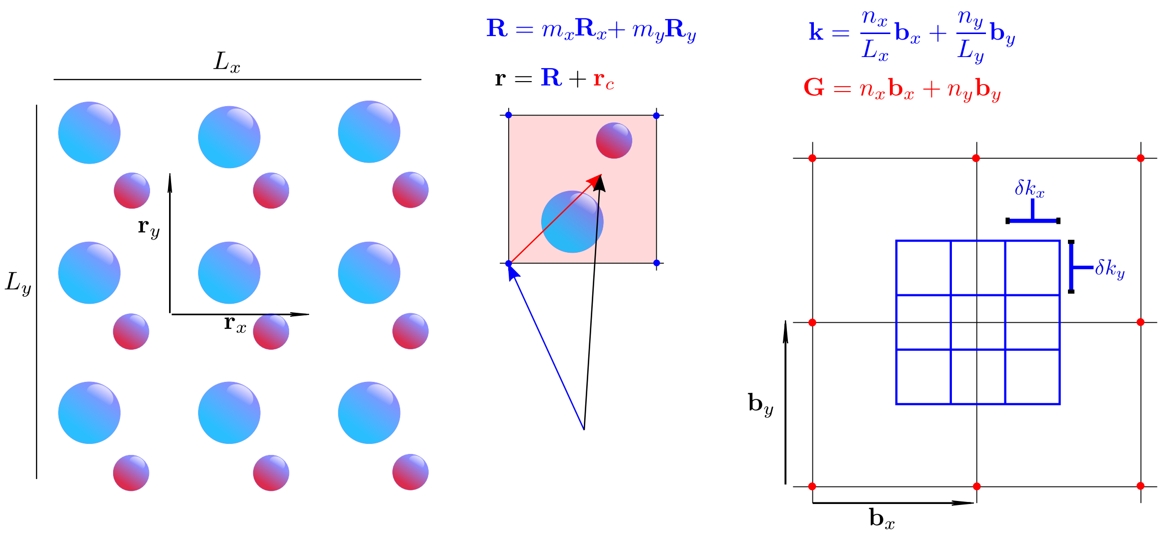

Supplementary Figure 3. Direct and reciprocal lattice of a 2D square Bravais lattice. Left: Square Bravais lattice with two atoms per unit cell. The lattice vectors are $\mathbf{r}_x$ and $\mathbf{r}_y$ and the size of the system is L_x and L_y. Middle: Every position $\mathbf{r}$ in the material can be decomposed in the position of the cell $\mathbf{R}$ and a position inside the cell $\mathbf{r}_c$. Right: The reciprocal lattice (black grid) is unbounded. The finer k-mesh (blue lattice) represents the different lattice momentum states $\mathbf{k}$. The system size determines the number of k points and does not affect the reciprocal lattice.

where the integral is over the unit cell. Using Eq. (2), we find

$$U_{\mathbf{G}} = \frac{q_e}{\epsilon_0 \Omega} \sum_a Z_a \frac{e^{i\mathbf{r}_a \cdot \mathbf{G}}}{|\mathbf{G}|^2}, \quad \text{for } \mathbf{G} \neq 0 \quad (U_0 = 0), \tag{20}$$

where the sum runs over the positions $\mathbf{r}_a$ of the atoms in the unit cell (see Supplementary Figure 3).

2. *Bloch wave basis*

Going back to the Hamiltonian of Eq. (18), in the non-interacting limit we see that the lattice momentum $\mathbf{k}$ enters as a parameter,

$$H_0 = \sum_{\mathbf{k},\mathbf{G},\mathbf{G}',\sigma} \left[\frac{|\hbar(\mathbf{k}+\mathbf{G})|^2}{2m} \delta_{\mathbf{G},\mathbf{G}'} + U_{\mathbf{G}-\mathbf{G}'} \right] c^{\dagger}_{\mathbf{k}+\mathbf{G},\sigma} c_{\mathbf{k}+\mathbf{G}',\sigma}. \tag{21}$$

This implies that we can decompose the Hamiltonian in different crystal momentum blocks as $H = \sum_{\mathbf{k}} H_0(\mathbf{k})$ and solve an independent Schrödinger equation for each of them,

$$H_0(\mathbf{k})|\Psi_n(\mathbf{k})\rangle = \epsilon_n(\mathbf{k})|\Psi_n(\mathbf{k})\rangle, \tag{22}$$

with $|\Psi_n(\mathbf{k})\rangle$ being a two-component spinor state. It is useful to define a particular zone of $\mathbf{k}$ values called the Brillouin zone, which corresponds to the Wigner-Seltz cell construction in reciprocal space, i.e., the locus of points $\mathbf{k}$ in the reciprocal space which is closer to $\mathbf{G} = 0$. The eigenvalues $\epsilon_n(\mathbf{k})$ define the energy bands of the system.

The expansion of the non-interacting Hamiltonian in the basis defined by the momentum block eigenstates of Eq. (22), can be obtained by diagonalising $h_{\mathbf{k},\mathbf{G}-\mathbf{G}'}$ in Eq. (18),

$$H_0(\mathbf{k}) \equiv \sum_{\mathbf{G},\mathbf{G}',\sigma} h_{\mathbf{k},\mathbf{G}-\mathbf{G}'} c^{\dagger}_{\mathbf{k},\mathbf{G},\sigma} c_{\mathbf{k},\mathbf{G}',\sigma} = \sum_{n,\sigma} \epsilon_n(\mathbf{k}) f^{\dagger}_{\mathbf{k},n,\sigma} f_{\mathbf{k},n,\sigma}, \tag{23}$$

where $f_{\mathbf{k},n,\sigma} = \sum_{\mathbf{G}} S_{n,\mathbf{G}}(\mathbf{k},\sigma) c_{\mathbf{k}+\mathbf{G},\sigma}$ ($f^\dagger_{\mathbf{k},n,\sigma}$) is the band fermion annihilation (creation) operator. Here, $S_{n,\mathbf{G}}(\mathbf{k},\sigma)$ is the unitary matrix that diagonalises h, i.e., $h_{\mathbf{k},\mathbf{G}-\mathbf{G}'} = \sum_n (S^\dagger)_{\mathbf{G},n}(\mathbf{k},\sigma)\epsilon_n(\mathbf{k}) S_{n,\mathbf{G}'}(\mathbf{k},\sigma)$. The index n here denotes the band and takes the same number of values $|n|$ as the reciprocal lattice vectors $\mathbf{G}$, i.e., $|n| = 8G^3_{\max}$ in $D = 3$ dimensions.

For a system with $\nu_{\rm el}$ electrons per unit cell (i.e., corresponding to a total of $\nu_{\rm el} \times N$ electrons), the system will have $\nu_{\rm el}$ occupied bands, as each band can accommodate N states, which is the number of different lattice momentum values in the Brillouin zone (note that $\nu_{\rm el}$ can be a rational number, in which case there are $\lfloor \nu_{\rm el} \rfloor$ fully occupied bands and the last band $\lceil \nu_{\rm el} \rceil$ is partially occupied).

In real space, the non-interacting Hamiltonian corresponding to each momentum block is $H_0(\mathbf{k}) = \frac{\hbar^2}{2m}(\mathbf{k} - i\nabla)^2 + \tilde{U}(\mathbf{r})$. The spin components of its eigenstates coincide with the periodic functions $u_{\mathbf{k},n,\sigma}(\mathbf{r})$ introduced in Eq. (14), i.e.,

$$H_0(\mathbf{k}) u_{\mathbf{k},n,\sigma}(\mathbf{r}) = \epsilon_n(\mathbf{k}) u_{\mathbf{k},n,\sigma}(\mathbf{r}), \tag{24}$$

with the boundary condition $u_{\mathbf{k}+\mathbf{G},n,\sigma}(\mathbf{r}) = e^{-i\mathbf{G}\cdot\mathbf{r}} u_{\mathbf{k},n,\sigma}(\mathbf{r})$. Note that the functions $u_{\mathbf{k},n,\sigma}(\mathbf{r})$ are defined within the unit cell via $u_{\mathbf{k},n,\sigma}(\mathbf{r}) = u_{\mathbf{k},n,\sigma}(\mathbf{r}_c + \mathbf{R}) = u_{\mathbf{k},n,\sigma}(\mathbf{r}_c)$, where $\mathbf{R}$ is a lattice vector and $\mathbf{r}_c$ is a vector with domain in the unit cell. By expanding $u_{\mathbf{k},n,\sigma}(\mathbf{r})$ in Eq. (24) in Fourier series one can verify that

$$u_{\mathbf{k},n,\sigma}(\mathbf{r}) = \sum_{\mathbf{G}} e^{i\mathbf{G}\cdot\mathbf{r}} S_{n,\mathbf{G}}(\mathbf{k},\sigma). \tag{25}$$

In the language of Supplementary Note 2 A, what we have done so far corresponds to expanding the electron operator on a Bloch wave basis (also called band fermion basis) $\{\phi_{\mathbf{k},n,\sigma}(\mathbf{r})\}$, with $\phi_{\mathbf{k},n,\sigma}(\mathbf{r}) = e^{i\mathbf{k}\cdot\mathbf{r}} u_{\mathbf{k},n,\sigma}(\mathbf{r})/\sqrt{V_c}$. In this basis, the full Hamiltonian is

$$H^B = \sum_{\mathbf{k},n,\sigma} \epsilon_n(\mathbf{k}) f^\dagger_{\mathbf{k},n,\sigma} f_{\mathbf{k},n,\sigma} + \sum_{\sigma,\sigma'} \sum_{\substack{n_1,n_2,n_3,n_4\\ \mathbf{k},\mathbf{q},\mathbf{k}'}} V^{(\mathbf{k},\mathbf{k}',\mathbf{q})}_{n_1n_2n_3n_4} f^\dagger_{\mathbf{k}+\mathbf{q},n_1,\sigma} f^\dagger_{\mathbf{k}'-\mathbf{q},n_2,\sigma'} f_{\mathbf{k}',n_3,\sigma'} f_{\mathbf{k},n_4,\sigma}, \tag{26}$$

with Coulomb tensor coefficients

$$V^{(\mathbf{k},\mathbf{k}',\mathbf{q})}_{n_1n_2n_3n_4} = \sum_{\mathbf{G},\mathbf{K},\mathbf{G}'} V_{\mathbf{q}+\mathbf{K}} S_{n_1,\mathbf{G}+\mathbf{K}}(\mathbf{k}+\mathbf{q},\sigma) S_{n_2,\mathbf{G}'-\mathbf{K}}(\mathbf{k}'-\mathbf{q},\sigma') S^*_{n_3,\mathbf{G}'}(\mathbf{k}',\sigma') S^*_{n_4,\mathbf{G}}(\mathbf{k},\sigma). \tag{27}$$

and $V_{\mathbf{q}+\mathbf{K}}$ defined after Eq. (18).

C. Real-space single-particle basis: Wannier functions

In Eq. (26), the quadratic part of the Hamiltonian is diagonal, but the electron-electron interaction is highly non-local. On the other hand, in a real-space coordinate basis (such as the one obtained by discretising the position operator $\mathbf{r}$ on a real-space grid), the electron-electron interaction is diagonal but non-local, while the kinetic term is not diagonal. To reduce the number of the relevant coefficients entering the Hamiltonian, one strategy is to look for a representation where both the hopping matrix and Coulomb tensor are not diagonal with respect to the single-particle basis, but as local (in real space) as possible. One convenient way to achieve this goal is to consider Wannier functions as the single-particle basis. The fermion annihilation operators associated with the latter are defined in [15] as

$$w_{\mathbf{R},n,\sigma} = \sum_{\mathbf{k},m} e^{i\mathbf{k}\cdot\mathbf{R}} U^*_{mn}(\mathbf{k}) f_{\mathbf{k},m,\sigma} \to f_{\mathbf{k},m,\sigma} = \frac{1}{N}\sum_{\mathbf{R},n} e^{-i\mathbf{k}\cdot\mathbf{R}} U_{mn}(\mathbf{k}) w_{\mathbf{R},n,\sigma}, \tag{28}$$

where $U_{mn}(\mathbf{k})$ is a unitary transformation representing the gauge freedom in the definition of the Bloch waves. In this basis, the Hamiltonian of Eq. (4) becomes

$$H^W = \sum_{\sigma} \sum_{\substack{m,n \\ \mathbf{R}_1,\mathbf{R}_2}} T(\mathbf{R}_1 - \mathbf{R}_2)_{mn} w^{\dagger}_{\mathbf{R}_1,m,\sigma} w_{\mathbf{R}_2,n,\sigma}$$
$$+ \sum_{\sigma,\sigma'} \sum_{\substack{s,l,m,n \\ \mathbf{R}_1,\mathbf{R}_2,\mathbf{R}_3,\mathbf{R}_4}} \tilde{V}^{(\mathbf{R}_1\mathbf{R}_2,\mathbf{R}_3,\mathbf{R}_4)}_{slmn} w^{\dagger}_{\mathbf{R}_1,s,\sigma} w^{\dagger}_{\mathbf{R}_2,l,\sigma'} w_{\mathbf{R}_3,m,\sigma'} w_{\mathbf{R}_4,n,\sigma}, \tag{29}$$

with the matrix elements

$$T(\mathbf{R})_{mn} = \frac{1}{N^2} \sum_{\mathbf{k}} e^{i\mathbf{k}\cdot\mathbf{R}} [U(\mathbf{k})\epsilon(\mathbf{k})U^{\dagger}(\mathbf{k})]_{nm}, \tag{30}$$

$$\tilde{V}^{(\mathbf{R}_1,\mathbf{R}_2,\mathbf{R}_3,\mathbf{R}_4)}_{slmn} = \frac{1}{N^4} \sum_{\substack{n_1n_2n_3n_4 \\ \mathbf{k},\mathbf{q},\mathbf{k}'}} V^{(\mathbf{k},\mathbf{k}',\mathbf{q})}_{n_1n_2n_3n_4} U^{*}_{n_1s}(\mathbf{k}+\mathbf{q}) U^{*}_{n_2l}(\mathbf{k}-\mathbf{q}) U_{n_3m}(\mathbf{k}') U_{n_4n}(\mathbf{k})$$
$$\times e^{i\mathbf{k}\cdot(\mathbf{R}_1-\mathbf{R}_4)} e^{i\mathbf{q}\cdot(\mathbf{R}_1-\mathbf{R}_2)} e^{i\mathbf{k}'\cdot(\mathbf{R}_2-\mathbf{R}_3)}. \tag{31}$$

The Wannier functions corresponding to the operators in Eq. (28) are

$$\mathcal{W}^{\mathbf{R}}_{s,\sigma}(\mathbf{r}) = \mathcal{W}^{\mathbf{0}}_{s,\sigma}(\mathbf{r}-\mathbf{R}) = \sum_{\mathbf{k},n} e^{-i\mathbf{k}\cdot\mathbf{R}} U_{ns}(\mathbf{k}) u_{\mathbf{k},n,\sigma}(\mathbf{r}) e^{i\mathbf{k}\cdot\mathbf{r}}. \tag{32}$$

In terms of the latter, the matrix elements of the hopping matrix and the Coulomb tensor can expressed as

$$T(\mathbf{R}_1 - \mathbf{R}_2)_{mn} = \int d\mathbf{r} \mathcal{W}^{\mathbf{R}_1 *}_{m,\sigma}(\mathbf{r}) \left[-\frac{\hbar^2\nabla^2}{2m} + \tilde{U}(\mathbf{r}) \right] \mathcal{W}^{\mathbf{R}_2}_{n,\sigma}(\mathbf{r}), \tag{33a}$$

$$\tilde{V}^{(\mathbf{R}_1,\mathbf{R}_2,\mathbf{R}_3,\mathbf{R}_4)}_{slmn} = \frac{1}{2} \int d\mathbf{r} \int d\mathbf{r}' \mathcal{W}^{\mathbf{R}_1 *}_{s,\sigma}(\mathbf{r}) \mathcal{W}^{\mathbf{R}_2 *}_{l,\sigma'}(\mathbf{r}') V(|\mathbf{r}-\mathbf{r}'|) \mathcal{W}^{\mathbf{R}_3}_{m,\sigma'}(\mathbf{r}') \mathcal{W}^{\mathbf{R}_4}_{n,\sigma}(\mathbf{r}). \tag{33b}$$

The discrete translational invariance of the lattice allows us to rewrite the coefficients above as

$$T(\mathbf{R})_{mn} = \int d\mathbf{r} \mathcal{W}^{\mathbf{R}}_{m,\sigma}(\mathbf{r}) \left[-\frac{\hbar^2\nabla^2}{2m} + \tilde{U}(\mathbf{r}) \right] \mathcal{W}^{\mathbf{0}}_{n,\sigma}(\mathbf{r}), \tag{34a}$$

$$\tilde{V}^{(\mathbf{0},\mathbf{R}_2,\mathbf{R}_3,\mathbf{R}_4)}_{slmn} = \frac{1}{2} \int d\mathbf{r} \int d\mathbf{r}' \mathcal{W}^{\mathbf{0}}_{s,\sigma}(\mathbf{r}) \mathcal{W}^{\mathbf{R}_2}_{l,\sigma'}(\mathbf{r}') V(|\mathbf{r}-\mathbf{r}'|) \mathcal{W}^{\mathbf{R}_3}_{m,\sigma'}(\mathbf{r}') \mathcal{W}^{\mathbf{R}_4}_{n,\sigma}(\mathbf{r}), \tag{34b}$$

with $\mathbf{R} = \mathbf{R}_1 - \mathbf{R}_2$.

Since the Coulomb tensor coefficients $\tilde{V}^{(\mathbf{0},\mathbf{R}_2,\mathbf{R}_3,\mathbf{R}_4)}_{slmn}$ involves integrals over the real space, if the Wannier functions $\mathcal{W}^{\mathbf{R}}_{s,\sigma}(\mathbf{r})$ are localized around $\mathbf{R}$, then the coefficients will decay fast for distant cells in the lattice. From the definition of the Wannier functions we have $\mathcal{W}^{\mathbf{0}}_{s,\sigma}(\mathbf{r}) \equiv \sum_{\mathbf{k}} v_{\mathbf{k},s,\sigma}(\mathbf{r}) e^{i\mathbf{k}\cdot\mathbf{r}}$, where $v_{\mathbf{k},s,\sigma}(\mathbf{r}) = \sum_n U_{ns}(\mathbf{k}) u_{\mathbf{k},n,\sigma}(\mathbf{r})$ are quasi-Bloch functions. This relation tells us that the quasi-Bloch functions and the Wannier functions are related by a Fourier transform. As discussed in Appendix B, we can then use the analytical form of the quasi-Bloch functions $v_{\mathbf{k},s,\sigma}(\mathbf{r})$ as a function of the crystal momentum $\mathbf{k}$ to show that maximally localized Wannier functions (MLWFs) can be obtained if the following conditions are satisfied [15–18]:

1. The system has a vanishing Chern number. This condition is automatically satisfied in systems with time-reversal symmetry, as in this case where the Chern number is zero. Note that systems without time-reversal symmetry can still have a vanishing Chern number.

2. An energy gap exists between the bands in the active space (see Supplementary Note 2 F 2 below) and the rest. Note that a system satisfying this condition does not necessarily represent an insulator, as the Fermi energy can lie within an active space which is separated from the rest of the bands.

In this case, both the non-interacting and the electron-electron interaction terms of the Hamiltonian in the Wannier basis of Eq. (29) contain only local terms. Moreover, MLWFs are always real [19]. This fact, combined with the hermiticity of the hopping matrix $T(\mathbf{R})_{mn} = T(-\mathbf{R})_{nm}$ (see Eq. (36) below), implies

$$T(\mathbf{R})_{mn} = T(-\mathbf{R})_{nm}. \tag{35}$$

This identity restricts the type of quadratic terms that can appear in the Hamiltonian. As we will discuss in Supplementary Note 2 D below, symmetry properties of the physical system further constrain the form of the Hamiltonian coefficients.

1. *Material real-space motif*

The key advantage of using a localised single-particle basis is that the magnitude of the hopping matrix and Coulomb tensor coefficients of Eq. (34) decreases quickly as a function of the distance between the unit cells involved in the integrals. A natural approximation is thus to consider only those coefficients involving cells which are reciprocal nearest neighbours of order n, with the latter depending on the material and on the degree of accuracy required for the Hamiltonian coefficients. In particular, we say that two unit cells A and B, identified by the lattice vectors $\mathbf{R}_A$ and $\mathbf{R}_B$, are nearest neighbors of order n if $|\mathbf{R}_A - \mathbf{R}_B| = d_n$, with $|...|$ denoting the Euclidean norm. Here, $0 = d_0 < d_1 < d_2 < ... < d_n$ is a ascending sequence of distances corresponding to nearest neighbors of order 0, 1, 2, and n, respectively. Within this approximation, we do not need to evaluate the hopping matrix and the Coulomb tensor coefficients over the whole material lattice but we can rather focus on a minimal set of cells which includes a central cell and its nearest neighbors up to order n. All the other coefficients can then be obtained by exploiting the discrete translational invariance of the lattice. For this reason, we will name such a minimal set of unit cells a *motif* of order n. As an example, in Supplementary Figure 4 we show the motifs of order $n = 1$ and $n = 2$ for silicon. Note that the motif is formed by the material unit cells and not by its individual atoms. We denote by $\mathcal{N}^n_{\mathcal{G}'}$ the set of all the unit cells of a material lattice $\mathcal{G}$ which are nearest neighbors of order $\leq n$ with respect to all the unit cells of a sublattice $\mathcal{G}' \subseteq \mathcal{G}$, i.e., $\mathcal{N}^n_{\mathcal{G}'} \equiv \{\, A \in \mathcal{G} \mid |\mathbf{R}_A - \mathbf{R}_B| \leq d_n, \forall B \in \mathcal{G}' \,\}$. The motif of order n centered on the unit cell O coincides with $\mathcal{N}^n_O$ and contains $N_{\text{cells/motif}} = \dim(\mathcal{N}^n_O)$ cells of the material lattice. Its Hamiltonian can be obtained from Eq. (29) by restricting the various summations over the lattice vectors only to those $\mathbf{R}_i$ corresponding to the unit cells forming the motif. Moreover, thanks to the discrete translational invariance of the lattice, only those coefficients involving the central cell of the motif at least once should be included in the motif Hamiltonian. See Appendix D 2 for more details on the explicit form of the latter.

As we will see in Supplementary Note 5 B, the presence of a motif can be exploited to implement highly parallelisable quantum circuits whose depth is independent from the total number of motifs forming the lattice of the simulated material. This fact leverages the capabilities of a hybrid fermion to qubit encoding [10, 20], which will be discussed in Supplementary Note 3. In view of this step, we notice that the sites of an arbitrary lattice can be labeled by the triplets of integers (n_1, n_2, n_3) introduced in Eq. (11). Therefore, any lattice can be visualized on the Cartesian grid defined by those triplets. This fact establishes a natural mapping between a (real-space) motif of order n and a *Cartesian motif* consisting of $N_C = L_x \times L_y \times L_z$ sites, with $L_i = \max_{(n_1,n_2,n_3)\in\mathcal{N}^n_O}(n_i) - \min_{(n_1,n_2,n_3)\in\mathcal{N}^n_O}(n_i) + 1$

Since, in general, $N_C \neq N_{\text{cells/motif}}$, the Cartesian motif contains $N_D = N_C - N_{\text{cells/motif}}$ additional cells. The latter do not enter the motif Hamiltonian but should be taken into account anyway during the encoding stage described in Supplementary Note 3. In Supplementary Figure 5, we show the Cartesian motifs corresponding to the motifs of order $n = 1$ and $n = 2$ for silicon. Here, grey spheres denote the additional N_D sites forming the Cartesian motif. Note that distances between unit cells ought to be computed in real space and that, as a result of the mapping, two units cells which are (real-space) nearest neighbours of order n may be nearest neighbours of order $n' \neq n$ on the Cartesian grid.

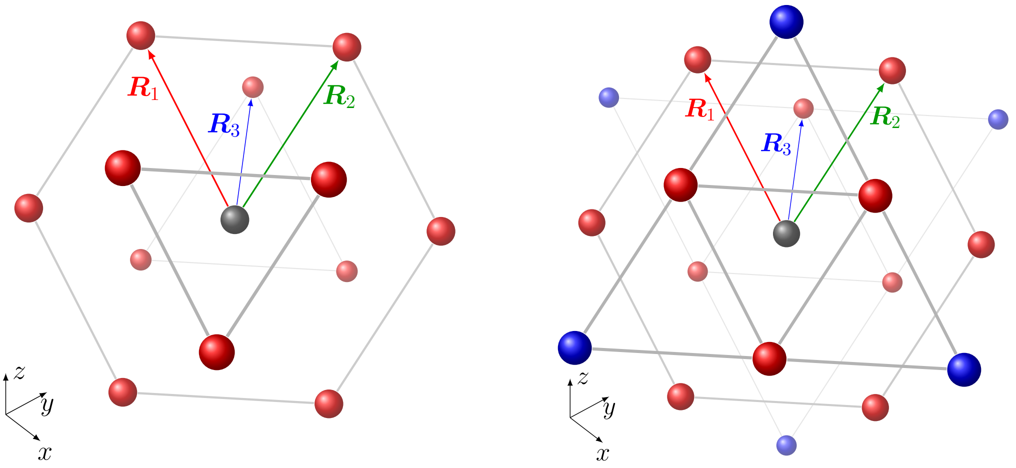

Supplementary Figure 4. Motif of order (left) $n = 1$ and (right) $n = 2$ for silicon. The motif of order 1 contains the central cell (black) and its 12 nearest neighbours (red), while the motif of order 2 includes also the 6 next-nearest neighbours (blue). The gray links connect the nearest and next-nearest neighbouring cells belonging to the same (111) crystallographic plane, with thinner lines denoting planes with larger distance with respect to the reader along the direction perpendicular to the page. $\mathbf{R}_1$, $\mathbf{R}_2$, $\mathbf{R}_3$ denote the silicon lattice vectors.

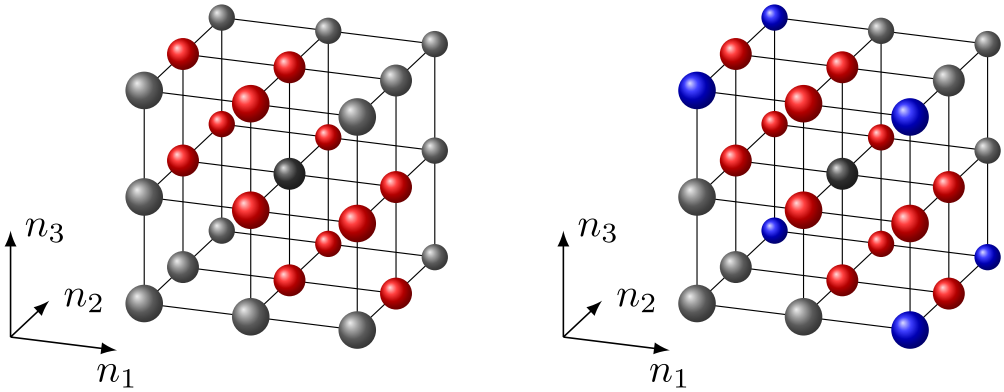

Supplementary Figure 5. Cartesian motifs corresponding to the motif of order (left) $n = 1$ and (right) $n = 2$ for silicon. As in Supplementary Figure 4, the central unit cell is in black, and real-space nearest neighbour and next-nearest neighbour cells are shown in red and blue, respectively. Grey spheres represent the additional N_D sites of the Cartesian motif which do not correspond to any cell of the motifs shown in Supplementary Figure 4. Note that, in general, nearest neighbour cells of order n in the motif can be nearest neighbours of order $n' \neq n$ in the Cartesian motif.

D. General constraints and symmetry properties of the fermion integrals

The single-particle wavefunctions determine the symmetry properties of the second-quantized Hamiltonian. For generic real space wavefunctions, the symmetry properties are discussed below. After that, we discuss in this section two important generic symmetries in the Bloch and Wannier basis respectively, inversion (Supplementary Note 2 D 2) and time reversal symmetry (Supplementary Note 2 D 3).

1. General constraints and symmetry properties of the fermion integrals for general real single-particle wavefunctions

The hopping matrix and Coulomb tensor of a general many-body system satisfy the following identities (see Eq. (5), Eq. (6)):

$$t_{\lambda_1\lambda_2} = t^*_{\lambda_2\lambda_1} \quad \text{(hermiticity)}, \tag{36}$$
$$V_{\lambda_1\lambda_2\lambda_3\lambda_4} = V_{\lambda_2\lambda_1\lambda_4\lambda_3} \quad \text{(swap symmetry)}, \tag{37}$$
$$V_{\lambda_1\lambda_2\lambda_3\lambda_4} = V^*_{\lambda_4\lambda_3\lambda_2\lambda_1} \quad \text{(hermiticity)}, \tag{38}$$
$$V_{\lambda_1\lambda_2\lambda_3\lambda_4} = V^*_{\lambda_3\lambda_4\lambda_1\lambda_2} \quad \text{(hermiticity + swap)}. \tag{39}$$

For single-particle bases with real wavefunctions $\{ \phi_\lambda(\mathbf{r}) \}$ (see Supplementary Note 2 C) the above relations simplify to

$$t_{\lambda_1\lambda_2} = t_{\lambda_2\lambda_1}, \tag{40}$$
$$\begin{aligned} V_{\lambda_1\lambda_2\lambda_3\lambda_4} &= V_{\lambda_4\lambda_3\lambda_2\lambda_1} = V_{\lambda_2\lambda_1\lambda_4\lambda_3} = V_{\lambda_4\lambda_2\lambda_3\lambda_1} = V_{\lambda_1\lambda_3\lambda_2\lambda_4} \\ &= V_{\lambda_3\lambda_4\lambda_1\lambda_2} = V_{\lambda_3\lambda_1\lambda_4\lambda_2} = V_{\lambda_2\lambda_4\lambda_1\lambda_3}. \end{aligned} \tag{41}$$

In particular, the latter set of equivalences has been obtained by combining the hermiticity and swap symmetry of the Coulomb tensor with the additional symmetry $V_{\lambda_1\lambda_2\lambda_3\lambda_4} = V_{\lambda_4\lambda_2\lambda_3\lambda_1} = V_{\lambda_1\lambda_3\lambda_2\lambda_4}$ arising from Eq. (6) for real wavefunctions and it can be exploited to significantly reduce the number of independent Coulomb tensor coefficients one has to directly compute.

After the single-particle basis has been picked and thus the second quantized Hamiltonian is fixed, it will have a form like Eq. (4). To pass into a qubit Hamiltonian, as we will discuss in Supplementary Note 3, it is useful to group together the single-particle quantum numbers and the spin in a single label $\xi_i = (\lambda_i, \sigma_i)$. Introducing the spin-dependent hopping matrix $\mathcal{T}_{\xi_1\xi_2}$ and Coulomb tensor $\mathcal{V}_{\xi_1\xi_2\xi_3\xi_4}$, we can re-write Eq. (4) as

$$H = \sum_{\xi_1,\xi_2} \mathcal{T}_{\xi_1\xi_2} c^\dagger_{\xi_1} c_{\xi_2} + \sum_{\xi_1,\xi_2,\xi_3,\xi_4} \mathcal{V}_{\xi_1\xi_2\xi_3\xi_4} c^\dagger_{\xi_1} c^\dagger_{\xi_2} c_{\xi_3} c_{\xi_4}. \tag{42}$$

Here the *spinful* hopping matrix is (with $\xi_i = (\lambda_i, \sigma_i)$)

$$\mathcal{T}_{\xi_1\xi_2} = \begin{cases} t_{\lambda_1\lambda_2} & \text{if } \sigma_1 = \sigma_2 \\ 0 & \text{otherwise,} \end{cases} \tag{43}$$

while the *spinful* Coulomb tensor is

$$\mathcal{V}_{\xi_1\xi_2\xi_3\xi_4} = \begin{cases} V^s_{\lambda_1\lambda_2\lambda_3\lambda_4} & \text{for } \sigma_1 = \sigma_2 = \sigma_3 = \sigma_4, \\ \frac{1}{2} V_{\lambda_1\lambda_2\lambda_3\lambda_4} & \text{if } \sigma_1 = \sigma_4 \text{ and } \sigma_2 = \sigma_3 \ (\sigma_1 \neq \sigma_2), \\ -\frac{1}{2} V_{\lambda_2\lambda_1\lambda_3\lambda_4} & \text{if } \sigma_1 = \sigma_3 \text{ and } \sigma_2 = \sigma_4 \ (\sigma_1 \neq \sigma_2) \\ 0 & \text{otherwise,} \end{cases} \tag{44}$$

with $V^s_{\lambda_1\lambda_2\lambda_3\lambda_4} = (V_{\lambda_1\lambda_2\lambda_3\lambda_4} - V_{\lambda_2\lambda_1\lambda_3\lambda_4})/2$.

The spinful Coulomb tensor $\mathcal{V}_{\xi_1\xi_2\xi_3\xi_4}$ is hermitian, $\mathcal{V}_{\xi_1\xi_2\xi_3\xi_4} = \mathcal{V}^*_{\xi_4\xi_3\xi_2\xi_1}$, and antisymmetric under exchange of the first or last pair of indices

$$\mathcal{V}_{\xi_1\xi_2\xi_3\xi_4} = -\mathcal{V}_{\xi_2\xi_1\xi_3\xi_4} = -\mathcal{V}_{\xi_1\xi_2\xi_4\xi_3} = \mathcal{V}_{\xi_2\xi_1\xi_4\xi_3}. \tag{45}$$

Note that if $\sigma_1 = \sigma_4$ and $\sigma_2 = \sigma_3$, $\mathcal{V}_{\xi_1\xi_2\xi_3\xi_4}$ obeys the same identities in Eq. (41) as $V_{\lambda_1\lambda_2\lambda_3\lambda_4}$. In this latter case, by exploiting Eq. (41) and Eq. (45), one can show that $\mathcal{V}_{\xi_1\xi_2\xi_3\xi_4}$ also satisfies the following Jacobi identity:

$$\mathcal{V}_{\xi_1\xi_2\xi_3\xi_4} + \mathcal{V}_{\xi_1\xi_3\xi_4\xi_2} + \mathcal{V}_{\xi_1\xi_4\xi_2\xi_3} = 0 \quad \text{if } \sigma_1 = \sigma_4 \text{ and } \sigma_2 = \sigma_3. \tag{46}$$

2. Inversion symmetry

Inversion symmetry $\mathcal{I}$ transforms space and momentum variables according to $\mathbf{r} \to -\mathbf{r}$ and $\mathbf{k} \to -\mathbf{k}$, respectively. In a crystal with inversion symmetry the external potential satisfies $\tilde{U}(\mathbf{r}) = \tilde{U}(-\mathbf{r})$.

a. Bloch basis. Under inversion, a Bloch wavefunction $\phi_{\mathbf{k},n,\sigma}(\mathbf{r})$ transforms as

$$\phi_{\mathbf{k},n,\sigma}(\mathbf{r}) \to \mathcal{I}\phi_{\mathbf{k},n,\sigma}(\mathbf{r}) = \phi_{\mathbf{k},n,\sigma}(-\mathbf{r}) = \phi_{-\mathbf{k},n,\sigma}(\mathbf{r}). \tag{47}$$

Recalling that in the Bloch basis the hopping matrix is $h_{mn}(\mathbf{k},\mathbf{k}') = \epsilon_m(\mathbf{k})\delta_{\mathbf{k},\mathbf{k}'}\delta_{mn}$, with

$$\epsilon_m(\mathbf{k}) = \int d\mathbf{r}\phi^*_{\mathbf{k},m,\sigma}(\mathbf{r}) \left[-\frac{\hbar^2\nabla^2}{2m} + \tilde{U}(\mathbf{r})\right] \phi_{\mathbf{k},m,\sigma}(\mathbf{r}), \tag{48}$$

in a crystal with inversion symmetry one finds

$$\epsilon_m(\mathbf{k}) = \epsilon_m(-\mathbf{k}). \tag{49}$$

Since the electron-electron interaction potential $V(|\mathbf{r}-\mathbf{r}'|)$ is always invariant under inversion, one also obtains

$$V^{(\mathbf{k},\mathbf{k}',\mathbf{q})}_{n_1n_2n_3n_4} = V^{(-\mathbf{k},-\mathbf{k}',-\mathbf{q})}_{n_1n_2n_3n_4}. \tag{50}$$

b. Wannier basis. Under inversion with respect to $\mathbf{R} = \mathbf{0}$, a Wannier function $\mathcal{W}^{\mathbf{R}}_{m,\sigma}(\mathbf{r})$ transforms as

$$\mathcal{W}^{\mathbf{R}}_{m,\sigma}(\mathbf{r}) \to \mathcal{I}\mathcal{W}^{\mathbf{R}}_{m,\sigma}(\mathbf{r}) = \mathcal{W}^{-\mathbf{R}}_{m,\sigma}(-\mathbf{r}). \tag{51}$$

First, we note that if the unitary transformation entering the definition of the Wannier basis in Eq. (28) is trivial, i.e., $U_{mn}(\mathbf{k}) = \delta_{mn}$, Eq. (30) reduces to

$$T(\mathbf{R})_{mn} = \frac{1}{N^2}\sum_{\mathbf{k}} e^{i\mathbf{k}\cdot\mathbf{R}}\epsilon_n(\mathbf{k})\delta_{mn}. \tag{52}$$

If the crystal has inversion symmetry, from Eq. (49) we have $\epsilon_n(\mathbf{k}) = \epsilon_n(-\mathbf{k})$ and, therefore,

$$T(\mathbf{R})_{mn} = T(-\mathbf{R})_{mn} = T(\mathbf{R})_{nm}, \quad \forall m,n, \tag{53}$$

where in the last step we used Eq. (35). Unfortunately, for MLWFs the unitary matrix $U_{mn}(\mathbf{k})$ is usually more complicated and the relation above does not hold in general.

A more general identity can be obtained if the Wannier functions transform under inversion as $\mathcal{I}\mathcal{W}^{\mathbf{R}}_{m,\sigma}(\mathbf{r}) = \mathcal{W}^{-\mathbf{R}}_{m,\sigma}(-\mathbf{r}) = \sum_{m'} P^\pi_{mm'}\mathcal{W}^{-\mathbf{R}}_{m',\sigma}(\mathbf{r})$, $\forall m,\mathbf{R}$, with P^π the generalized permutation matrix corresponding to a permutation π acting on the orbital indices, with $P^\pi_{mm'} = \eta_m = \pm 1$ for $m' = \pi(m)$ and $P^\pi_{mm'} = 0$ otherwise. In simpler terms, the latter condition implies that under inversion a given Wannier function with orbital index m and centred at $\mathbf{R}$ transforms into another Wannier function with orbital index m' (which can be different from m) centred at $-\mathbf{R}$. Since usually Wannier functions retain the main features of the corresponding atomic orbitals, this a quite common situation. For Wannier functions centred at the centre of the unit cell one usually has $P^\pi_{mm'} = \pm\delta_{mm'}$. For instance, the action of the inversion symmetry on a p_x-type orbital centred at $\mathbf{R} = \mathbf{0}$ is $\mathcal{I}p^{\mathbf{0}}_x = -p^{\mathbf{0}}_x$. In this case, in a crystal with inversion symmetry, one obtains

$$T(\mathbf{R})_{mn} = \sum_{m',n'} P^\pi_{mm'}P^\pi_{nn'}T(\mathbf{R})_{m'n'} = \eta_m\eta_n T(\mathbf{R})_{\pi(m)\pi(n)}, \tag{54}$$

from which one can see that

$$T(\mathbf{R})_{mn} = 0 \quad \text{if } (\pi(m),\pi(n)) = (m,n) \text{ and } \eta_m\eta_n = -1. \tag{55}$$

The identity above can be generalized by noting that for $\mathbf{R} = \mathbf{0}$ we have $T(\mathbf{0})_{mn} = T(\mathbf{0})_{nm}$ and hence the two orbital configurations (m,n) and (n,m) are equivalent in the computation of the

hopping matrix. We can then introduce the set of the orbital configurations equivalent to (m,n) as $[(m,n)]^{\mathbf{0}} = \{\,(m,n),(n,m)\,\}$ and $[(m,n)]^{\mathbf{R}} = \{\,(m,n)\,\}$ for $\mathbf{R} \neq 0$. Using this fact, we finally obtain

$$T(\mathbf{R})_{mn} = 0 \quad \text{if } (\pi(m),\pi(n)) \in [(m,n)]^{\mathbf{R}} \text{ and } \eta_m\eta_n = -1. \tag{56}$$

On the other hand, the electron-electron interaction potential $V(|\mathbf{r}-\mathbf{r}'|)$ is always invariant under inversion. In cases with Wannier functions transforming as $\mathcal{I}\mathcal{W}^{\mathbf{R}}_{m,\sigma}(\mathbf{r}) = \mathcal{W}^{-\mathbf{R}}_{m,\sigma}(-\mathbf{r}) = \sum_{m'} P^{\pi}_{mm'}\mathcal{W}^{-\mathbf{R}}_{m',\sigma}(\mathbf{r})$ under inversion, one obtains

$$\tilde{V}^{(\mathbf{R}_1,\mathbf{R}_2,\mathbf{R}_3,\mathbf{R}_4)}_{s,l,m,n} = 0 \quad \text{if } (\pi(s),\pi(l),\pi(m),\pi(n)) \in [(s,l,m,n)]^{\mathbf{R}_1\mathbf{R}_2\mathbf{R}_3\mathbf{R}_4} \text{ and } \eta_s\eta_l\eta_m\eta_n = -1. \tag{57}$$

Here, $[(s,l,m,n)]^{\mathbf{R}_1\mathbf{R}_2\mathbf{R}_3\mathbf{R}_4}$ is the set of equivalent orbital configurations according to Eq. (41). See Appendix D 2 b for details. The equation above is particularly useful since it allows one to determine a number of coefficients of the Coulomb tensor which are identically zero without having to compute them explicitly.

A general discussion on discrete crystal symmetries and the structure of matrix elements is presented in Supplementary Note 2 E.

3. Time-reversal symmetry

Under time-reversal symmetry $\mathcal{T}$ momentum and spin variables transform according to $\mathbf{k} \to -\mathbf{k}$ and $\mathbf{s} \to -\mathbf{s}$, with $\mathbf{s} = \boldsymbol{\sigma}/2$ and $\boldsymbol{\sigma} = (\sigma_x,\sigma_y,\sigma_z)$. Time-reversal symmetry is represented by an anti-unitary operator. One common choice is $\mathcal{T} = e^{-i\pi\sigma_y}K$, with K denoting the complex conjugation operator and the unitary operator $e^{-i\pi\sigma_y}$ performing a $\pi-$rotation of the spin around the $y-$axis. If the crystal possesses time-reversal symmetry, the external potential $\tilde{U}(\mathbf{r})$ is spin-independent and the electronic bands are doubly degenerate. This is always the case for a non-relativistic system (e.g., with no spin-orbit coupling) and in the absence of an external magnetic field. Since both these assumptions have been made in Supplementary Note 2, the general Hamiltonians of Eq. (1) and Eq. (4) already take into account the consequences of time-reversal invariance. To identify the latter in an explicit way, in this section we will examine a generalization of the Hamiltonians considered in Supplementary Note 2 with a spin-dependent external potential $\tilde{U}_{\sigma_1\sigma_2}(\mathbf{r})$. In this case, the quadratic part of Eq. (4) should be modified as follows,

$$H_0 = \sum_{\sigma_1,\sigma_2}\sum_{\lambda_1,\lambda_2} t^{\sigma_1,\sigma_2}_{\lambda_1\lambda_2} c^{\dagger}_{\lambda_1,\sigma_1} c_{\lambda_2,\sigma_2}, \tag{58}$$

with

$$t^{\sigma_1,\sigma_2}_{\lambda_1\lambda_2} = \int d\mathbf{r}\ \phi^*_{\lambda_1,\sigma_1}(\mathbf{r}) \left[-\frac{\hbar^2\nabla^2}{2m} + \tilde{U}_{\sigma_1\sigma_2}(\mathbf{r})\right] \phi_{\lambda_2,\sigma_2}(\mathbf{r}). \tag{59}$$

a. Bloch basis. Under time-reversal a Bloch wavefunction $\phi_{\mathbf{k},n,\sigma}(\mathbf{r})$ transforms as

$$\phi_{\mathbf{k},n,\uparrow}(\mathbf{r}) \to \phi_{-\mathbf{k},n,\downarrow}(\mathbf{r}) = \phi_{\mathbf{k},n,\downarrow}(-\mathbf{r}) \quad \text{and} \quad \phi_{\mathbf{k},n,\downarrow}(\mathbf{r}) \to -\phi_{-\mathbf{k},n,\uparrow}(\mathbf{r}) = -\phi_{\mathbf{k},n,\uparrow}(-\mathbf{r}). \tag{60}$$

If the external potential $\tilde{U}(\mathbf{r})$ is spin-independent, the hopping matrix coefficients $h^{\sigma,\sigma'}_{mn}(\mathbf{k},\mathbf{k}') = \epsilon_m(\mathbf{k})\delta_{\mathbf{k},\mathbf{k}'}\delta_{m,n}\delta_{\sigma,\sigma'}$ satisfy the identity

$$\epsilon_m(\mathbf{k}) = \epsilon_m(-\mathbf{k}), \tag{61}$$

while for the Coulomb tensor coefficients one finds

$$V^{(\mathbf{k},\mathbf{k}',\mathbf{q})}_{n_1n_2n_3n_4} = V^{(-\mathbf{k},-\mathbf{k}',-\mathbf{q})}_{n_1n_2n_3n_4}. \tag{62}$$

Note that the above equations are the same as the ones we obtained for a system with inversion symmetry.

On the other hand, in the presence of a spin-dependent but time-reversal invariant external potential $\tilde{U}_{\sigma_1\sigma_2}(\mathbf{r}) = \tilde{U}_{\bar{\sigma}_1\bar{\sigma}_2}(\mathbf{r})$ (where $\bar{\sigma}_i = -\sigma_i$), one finds $h^{\sigma,\sigma'}_{mn}(\mathbf{k},\mathbf{k}') = \epsilon^{\sigma,\sigma'}_m(\mathbf{k})\delta_{\mathbf{k},\mathbf{k}'}\delta_{m,n}$, with $\epsilon^{\sigma,\sigma'}_m(\mathbf{k}) \in \mathbb{R}$ and

$$\epsilon^{\uparrow,\uparrow}_m(\mathbf{k}) = \epsilon^{\downarrow,\downarrow}_m(-\mathbf{k}) \quad \text{and} \quad \epsilon^{\uparrow,\downarrow}_m(\mathbf{k}) = -\epsilon^{\downarrow,\uparrow}_m(-\mathbf{k}) = -\epsilon^{\uparrow,\downarrow}_m(-\mathbf{k}). \tag{63}$$

If, in addition, the crystal also possesses inversion symmetry, the above equation becomes

$$\epsilon^{\uparrow,\uparrow}_m(\mathbf{k}) = \epsilon^{\downarrow,\downarrow}_m(\mathbf{k}) \quad \text{and} \quad \epsilon^{\uparrow,\downarrow}_m(\mathbf{k}) = -\epsilon^{\uparrow,\downarrow}_m(\mathbf{k}) = 0. \tag{64}$$

b. Wannier basis. Under time-reversal a Wannier function $\mathcal{W}^{\mathbf{R}}_{n,\sigma}(\mathbf{r})$ transforms as

$$\mathcal{W}^{\mathbf{R}}_{n,\uparrow}(\mathbf{r}) \to \mathcal{W}^{\mathbf{R}}_{n,\downarrow}(\mathbf{r}) \quad \text{and} \quad \mathcal{W}^{\mathbf{R}}_{n,\downarrow}(\mathbf{r}) \to -\mathcal{W}^{\mathbf{R}}_{n,\uparrow}(\mathbf{r}). \tag{65}$$

If the external potential $\tilde{U}(\mathbf{r})$ is spin-independent, time-reversal invariance does not lead to any further relation for the hopping matrix and Coulomb tensor coefficients.

On the other hand, in the presence of a spin-dependent and time-reversal invariant external potential $\tilde{U}_{\sigma_1\sigma_2}(\mathbf{r}) = \tilde{U}_{\bar{\sigma}_1\bar{\sigma}_2}(\mathbf{r})$, one obtains

$$T(\mathbf{R})^{\uparrow,\uparrow}_{mn} = T(\mathbf{R})^{\downarrow,\downarrow}_{mn} \quad \text{and} \quad T(\mathbf{R})^{\uparrow,\downarrow}_{mn} = -T(\mathbf{R})^{\downarrow,\uparrow}_{mn}, \tag{66}$$

where we have used the reality of the matrices $T(\mathbf{R})$, that follows from the reality of the Wannier functions. Finally, from the last identity and the hermiticity of the Hamiltonian it follows that $T(\mathbf{0})^{\uparrow,\downarrow}_{mm} = 0$.

E. Crystal symmetries

A Hamiltonian H invariant under a symmetry group G (e.g., crystal symmetries, inversion, discrete rotations, etc), satisfies particular *selection rules* that determine the type of operators allowed in the Hamiltonian. In this section we discuss the general strategy to determine those rules for a general discrete group. Leveraging those constraints allows us to reduce the number of classical computations (computations of fermion integrals) and, more importantly, reduces the number of gates needed to implement the Hamiltonian by constraining the allowed operators in H.

Let us consider the electron-electron interaction term in Eq. (4). The single-particle wavefunctions used to span the Hilbert space form a basis that can be chosen to transform under a definite representation of the symmetry of the Hamiltonian. In this case, a symmetry operation $g \in G$, with representation $D^{(\mu)}_{\lambda_i\lambda_i'}(g)$ (which can always be chosen to be some irreducible representation μ), satisfies

$$\sum_{\lambda_1',\lambda_2',\lambda_3',\lambda_4'} D^{\dagger(\mu_1)}_{\lambda_1\lambda_1'}(g) D^{\dagger(\mu_2)}_{\lambda_2\lambda_2'}(g) D^{(\mu_3)}_{\lambda_3\lambda_3'}(g) D^{(\mu_4)}_{\lambda_4\lambda_4'}(g) V_{\lambda_1'\lambda_2'\lambda_3'\lambda_4'} = V_{\lambda_1\lambda_2\lambda_3\lambda_4}. \tag{67}$$

For simplicity we assume that the symmetries discussed here are unitary.

The equation above means in particular that the tensor product of different representations involved in a particular element of the interaction tensor should contain the trivial representation, that does not transform under the symmetry. In general, the tensor product of representations can be decomposed into the direct sum of irreducible representations (irreps) as [21]

$$D^{(\mu_1\times\mu_2)}(g) = \bigoplus_{\nu} c_\nu(\mu_1,\mu_2) D^{(\nu)}(g), \tag{68}$$

where $c_\nu(\mu_1,\mu_2)$ is the number of times that the irrep ν appears in the tensor product of irreps μ_1 and μ_2. Using this equation repeatedly and taking the trace, i.e., $\chi^{(\mu)}(g) = \mathrm{Tr}(D^{(\mu)}(g))$ with $\chi^{(\mu)}$ the character of $D^{(\mu)}$, we find the relation between the characters $\chi^{*(\mu_1\times\mu_2)}(g)\chi^{(\mu_3\times\mu_4)}(g) = \sum_{\nu,\nu',\rho} c_\nu(\mu_1,\mu_2)c_{\nu'}(\mu_3,\mu_4)\chi^{*(\nu)}(g)\chi^{(\nu')}(g)$. In this product the number of times that the trivial representation e appears can be computed using the orthogonality relation of the characters $c_e(\nu,\nu') = \frac{1}{|G|}\sum_g \chi^{*(\nu)}(g)\chi^{(\nu')}(g) = \delta_{\nu\nu'}$, where $|G|$ is the dimension of G [21].

For a given matrix element of a Hamiltonian to have a chance of being invariant under the symmetry, the representations involved in that matrix element should be such that their tensor product contains a copy of the trivial representation. This means that the tensor product of the irreps corresponding to non-zero coefficients of the Coulomb tensor can be decomposed as $D^{\dagger(\mu_1\times\mu_2)}(g)D^{(\mu_3\times\mu_4)}(g) = \bigoplus_\nu c_\nu(\mu_1,\mu_2)c_\nu(\mu_3,\mu_4)D^{(e)}(g)$.

Starting from the definition of the Coulomb tensor

$$V^{\mu_1\mu_2\mu_3\mu_4}_{\lambda_1\lambda_2\lambda_3\lambda_4} = \int d\mathbf{r}d\mathbf{r}'\phi^{*(\mu_1)}_{\lambda_1}(\mathbf{r})\phi^{*(\mu_2)}_{\lambda_2}(\mathbf{r}')V(|\mathbf{r}-\mathbf{r}'|)\phi^{(\mu_3)}_{\lambda_3}(\mathbf{r}')\phi^{(\mu_4)}_{\lambda_4}(\mathbf{r}), \tag{69}$$

where with a slight abuse of notation we use the label and the representation under which the single-particle wavefunction $\phi^{(\mu_i)}_{\lambda_i}(\mathbf{r})$ transforms as indices for the tensor, we can decompose it into irreps to find

$$V^{\mu_1\mu_2\mu_3\mu_4}_{\lambda_1\lambda_2\lambda_3\lambda_4} = \sum_\nu\sum_{s,s'} C^{*\mu_1\mu_2,\nu}_{\lambda_1\lambda_2,s}C^{\mu_3\mu_4,\nu}_{\lambda_3\lambda_4,s'}\int d\mathbf{r}d\mathbf{r}'\Psi^{*(\nu)}_s(\mathbf{r},\mathbf{r}')V(|\mathbf{r}-\mathbf{r}'|)\Psi^{(\nu)}_{s'}(\mathbf{r}',\mathbf{r}), \tag{70}$$

with $\{\Psi^{(\nu)}_{s'}\}$ the set of the basis functions of $D^{(\nu)}(g)$. The three-leg tensor $C^{\mu_1\mu_2,\nu}_{\lambda_1\lambda_2,s}$ (a Clebsch-Gordan coefficient of the group G) transforms the tensor product of basis in the μ_1 and μ_2 irreps into a new basis transforming in the ν irrep. They can be computed via the relation [21]

$$\frac{1}{|G|}\sum_g D^{(\mu_1)}_{\lambda_1\lambda_2}(g)D^{(\mu_2)}_{\lambda_3\lambda_4}(g)D^{(\nu)}_{ss'}(g) = \left(\frac{1}{\sqrt{n_\nu}}C^{\mu_1\mu_2,\nu}_{\lambda_1\lambda_3,s}\right)\left(\frac{1}{\sqrt{n_\nu}}C^{\mu_1\mu_2,\nu}_{\lambda_2\lambda_4,s'}\right)^*, \tag{71}$$

where n_ν is the dimension of the irrep ν. Fixing $\lambda_1=\lambda_2, \lambda_3=\lambda_4$, and $s=s'$, we have

$$\frac{1}{|G|}\sum_g D^{(\mu_1)}_{\lambda_1\lambda_1}(g)D^{(\mu_2)}_{\lambda_3\lambda_3}(g)D^{(\nu)}_{ss}(g) = \left|\frac{1}{\sqrt{n_\nu}}C^{\mu_1\mu_2,\nu}_{\lambda_1\lambda_3,s}\right|^2. \tag{72}$$

The equation above allows us to look for the set of labels where the Clebsch-Gordan coefficient does not vanish. Together with Eq. (70), this determines the selection rules, and can be used to find the set of allowed labels (i.e., the ones corresponding to non-vanishing terms) in the Coulomb tensor.

a. Example: $\mathbb{Z}_m$ group. The different irreps of the cyclic group are all one dimensional and they are parameterised by $D^{(\mu)}(g^n) = e^{i\frac{2\pi}{m}\mu n}$, with $\mu = 0,\ldots,m-1$ and $n=0,\ldots,m-1$. Eq. (72) becomes

$$\frac{1}{m}\sum_{n=0}^{m-1} e^{i\frac{2\pi}{m}(\mu_1+\mu_2+\nu)n} = \delta_{\mu_1+\mu_2+\nu,0} = |C^{\mu_1\mu_2,\nu}|^2, \tag{73}$$

from where we find the selection rule $\mu_1+\mu_2+\mu_3+\mu_4 = 0$. Let us apply this to one of the many symmetries of Silicon. The lattice vectors (in Å) are $a=(-2.7,0,2.7), b=(0,2.7,2.7)$ and $c=(-2.7,2.7,0)$. Starting with four valence WFs, each one aligned with one of the four axis of a Silicon tetrahedron (see Supplementary Figure 6),

$$\begin{aligned} &W^{Si}_1 : a+b-3c \to \text{axis } 1, \quad W^{Si}_2 : -3a+b+c \to \text{axis } 2,\\ &W^{Si}_3 : a-3b+c \to \text{axis } 3, \quad W^{Si}_4 : a+b+c \to \text{axis } 4, \end{aligned} \tag{74}$$

a $\mathbb{Z}_3$ rotation around each of these axes is a symmetry of the crystal (we consider only these symmetries for simplicity of exposition, recalling that the full space group of Silicon is m3m). We call S_j the operator associated with a rotation by $2\pi/3$ around an axis j. In Supplementary Figure 6 the transformation generated by S_4 is shown. These transformations permute the WFs in the basis $[W^{Si}_1, W^{Si}_2, W^{Si}_3, W^{Si}_4]^T$. Focusing on S_4, we can find a basis where the action of the symmetry is diagonal, i.e., $S_4\tilde{W}_aS_4^\dagger = \omega^a\tilde{W}_a$, with $\omega = e^{\frac{2\pi i}{3}}$. This basis consists of $\tilde{W}_4 = W^{Si}_4$ and

$$\begin{bmatrix}\tilde{W}_0\\ \tilde{W}_1\\ \tilde{W}_2\end{bmatrix} = \frac{1}{\sqrt{3}}\begin{bmatrix}1 & 1 & 1\\ 1 & \omega & \omega^2\\ 1 & \omega^2 & \omega\end{bmatrix}\begin{bmatrix}W^{Si}_1\\ W^{Si}_2\\ W^{Si}_3\end{bmatrix}. \tag{75}$$

The selection rule of Eq. (73) applies now for the symmetry $\mathbb{Z}_3$ and is nothing more than the condition that the overall phase that the Coulomb term in the $\tilde{W}$ basis acquires under S_4 is zero, fixing the product of functions in Eq. (69) to have the form $\tilde{W}_a\tilde{W}_b\tilde{W}_c\tilde{W}_d$ with $a+b+c+d = 0$ mod 3.

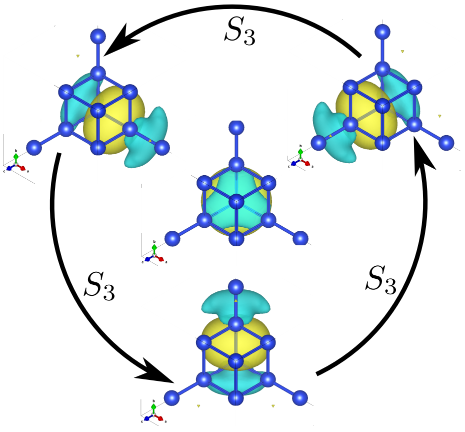

Supplementary Figure 6. Four valence Wannier functions of Silicon. Each of these is aligned with respect to the axes 1 through 4 of Eq. (74). A $\mathbb{Z}_3$ rotation around one of the axis maps the Wannier functions into themselves. The transformation matrix together with the invariance of the Hamiltonian determines the selection rules.

b. Example: Octahedral metal centre. As a further example of how symmetry could help in reducing the number of Coulomb tensor coefficients one has to compute, we now consider a cluster consisting of a transition metal atom (e.g., Manganese (Mn)) surrounded by six Oxygen (O) atoms. This structure is typical of many transition metal oxides and perovskites.

In our analysis, we employ renormalized Hydrogen-like atomic orbitals. The main reason for that is that they are simpler than Wannier functions and they feature the same symmetry properties. They are defined as

$$\psi_{nlm}(Z_{\text{eff}};\mathbf{r}) = R_{nl}(Z_{\text{eff}};r)X_l^m(\mathbf{r}), \tag{76}$$

with n, l, m the standard principal, angular, and magnetic quantum numbers, Z_{eff} the effective nuclear charge [22], $X_l^m(\mathbf{r})$ cubic spherical harmonics, and

$$R_{nl}(Z_{\text{eff}};r) = \sqrt{\left(\frac{Z_{\text{eff}}}{n}\right)^3 \frac{(n-l-1)!}{2n(n+l)!}}\, e^{-\frac{Z_{\text{eff}}r}{2n}} \left(\frac{Z_{\text{eff}}r}{n}\right)^l L_{n-l-1}^{2l+1}\left(\frac{Z_{\text{eff}}r}{n}\right) \tag{77}$$

the radial wavefunction. Here, lengths are measured in units of $a_0/2$, with a_0 the Born radius, and $L_{n-l+1}^{2l+1}(z)$ is the generalized Laguerre polynomial of degree $n-l+1$.

In what follows we will assume that the largest contribution to the Coulomb tensor is due to the 5 d orbitals centred at the transitional metal, $d_{xy}, d_{xz}, d_{xz}, d_{x^2-y^2}, d_{z^2}$, whose corresponding wavefunctions are denoted as $\mathcal{W}_a(\mathbf{r})$ with $a = 1, ..., 5$, respectively. We are interested in calculating the Coulomb tensor coefficients in the central unit cell

$$V_{abcd} = \int d\mathbf{r}d\mathbf{r}'\mathcal{W}_a(\mathbf{r})\mathcal{W}_b(\mathbf{r}')V(|\mathbf{r}-\mathbf{r}'|)\mathcal{W}_c(\mathbf{r})\mathcal{W}_d(\mathbf{r}'). \tag{78}$$

In doing that, symmetry properties can be exploited to determine a priori which elements of V are vanishing. Here, we take into account the reflection symmetry along the x, y, and z axes, and $\pi/2$ rotations around the $z-$axis. The corresponding operators are denoted by $\mathcal{I}_x$, $\mathcal{I}_y$, $\mathcal{I}_z$, and $\mathcal{I}_R$, respectively. Their action on the orbitals $W_a(\mathbf{r})$ is $\mathcal{I}_\mu\mathcal{W}_a(\mathbf{r}) = \sum_{a'} P_{aa'}^{\pi_\mu}\mathcal{W}_{a'}(\mathbf{r})$, with $\mu = \{x, y, z, R\}$ and P^{π_μ} a generalized permutation matrix corresponding to the permutation of the wavefunction

No symmetry	Inversion	Inversion + Rotation
325	157	129

Supplementary Table 1. Number of independent Coulomb tensor coefficients to be computed for a transition metal cluster with 5 d orbitals without taking into account any symmetry, by exploiting the inversion symmetry only, and by considering both the inversion and rotational symmetries, respectively. Note that the table reports the minimal number of coefficients required to determine the whole Coulomb tensor via the general relations given in Eq. (41), i.e., the number of the independent coefficients.

indices π_μ, with $P^{\pi_\mu}_{aa'} = \eta^\mu_a = \pm 1$ if $a' = \pi_\mu(a)$ and $P^{\pi_\mu}_{aa'} = 0$ otherwise. Exploiting the fact that $V(|\mathbf{r} - \mathbf{r}'|)$ is invariant under the operations associated with $\mathcal{I}_\mu$, we can identify which elements of the Coulomb tensor are zero. In particular, $V_{abcd} = 0$ if $(\pi_\mu(a), \pi_\mu(b), \pi_\mu(c), \pi_\mu(d)) \in [(i,j,k,l)]$ and $\eta^\mu_a \eta^\mu_b \eta^\mu_c \eta^\mu_d = -1$. Here, $[(i,j,k,l)]$ is the set of all the configurations equivalent to (a,b,c,d) according to Eq. (41). See Appendix D 2 b for additional details about the definition of $[(i,j,k,l)]$.

Supplementary Table 1 shows that symmetries may allow us to reduce significantly the number of Coulomb tensor coefficients we need to compute. For the particular example we have illustrated in this section, the number of the required coefficients passes from 325 (original case with no symmetry exploited), to 157 if inversion symmetry is taken into account and to 129 if both inversion and $\pi/2$ rotational symmetry around the $z-$axis are considered, respectively.

F. Using DFT to choose degrees of freedom

As we discussed in previous sections, in order to obtain the Bloch and Wannier functions in an actual material it is necessary to obtain the eigenstates of the electronic gas moving in the ionic potential $\tilde{U}(\mathbf{r})$, which is completely determined by just knowing the position of the ions. This requires solving a system of N Schrodinger equations for each possible value of the wavevector $\mathbf{k}$ (see Eq. (24)), a task which is classically unfeasible for most common materials. However, this procedure is generally unnecessarily complex as there are many electrons that are strongly bound to the ions (core electrons) which, if the processes involved are not very energetic, will not participate in the chemistry of the system. Therefore, the properties of a material can be effectively determined by studying the motion of the outermost electrons in a modified ionic potential (pseudo-potential) which combines the original ionic potential with the screening effects of the core electrons. An efficient way of dealing with this approach, which has been developed to maturity in the last century, is density functional theory (DFT) [23, 24]. In this section, we discuss how to obtain Bloch and Wannier functions within the framework of DFT and how to select the relevant degrees of freedom for the description of the system.

DFT is a highly efficient, accurate and flexible method for simulating atomic systems. It has enjoyed decades of success for simulating the ground state properties of numerous quantum systems at the atomic scale across the entire periodic table for translationally invariant systems [25, 26].

In this work, we use DFT to generate single-particle Kohn-Sham states (described below) which then are used to select an active space of bands where the relevant processes occur. Once this active space has been chosen, we generate Maximally localised Wannier functions (MLWFs) for the construction of second quantised many-body Hamiltonians.

DFT formally states that there exists an exact mapping between the external potential of a many-body system and its ground state density $n_0(\mathbf{r})$, whereby the nondegenerate ground-state wavefunction is a unique functional of the ground-state density, i.e.,

$$\Psi_0(\mathbf{r_1}, \mathbf{r}_2, \ldots, \mathbf{r}_N) = \Psi_0[n_0(\mathbf{r})]. \tag{79}$$

An important ground-state property is its energy, and Hohenberg and Kohn additionally proved that it is minimal if the electron density is the ground-state electron density [27],

$$E[n_0(\mathbf{r})] \leq E[n(\mathbf{r})], \tag{80}$$

where $n(\mathbf{r})$ is the density of the system. Kohn and Sham subsquently showed how to map the fully

interacting many-body problem onto a single-particle problem [28], i.e.,

$$\left[\frac{\hbar^2}{2m}\nabla^2 + \tilde{U}_{\text{eff}}(\mathbf{r})\right]\phi_i(\mathbf{r}) = \epsilon_i\phi_i(\mathbf{r}), \tag{81}$$

where $\tilde{U}_{\text{eff}}(\mathbf{r})$ is the effective Kohn-Sham potential, ϵ_i are the single-particle Kohn-Sham eigenvalues and, $\{\,\phi_i(\mathbf{r})\,\}$ are the Kohn-Sham states. The latter are fictitious orbitals (i.e., they are not formally related to any physical electron state) such that they must obey the following constraint

$$n_0(\mathbf{r}) = \sum_{i=1}^{\text{occ}} |\phi_i(\mathbf{r})|^2, \tag{82}$$

where $n_0(\mathbf{r})$ is the ground state density and the sum runs over all occupied electron states. This constraint fixes the electron occupation in the system.

While DFT is in principle an *ab initio* method, i.e., it requires only the lattice structure of the system, practically it necessitates the choice of *(i)* a basis for the fictitious single-particle Kohn-Sham states $\{\phi_i(\mathbf{r})\}$ and *(ii)* a parameterized Kohn-Sham exchange-correlation functional. In this work, for the DFT exploration we exclusively consider plane-wave basis sets, as implemented in Quantum Espresso [29, 30], which are subsequently transformed into real-space MLWFs using Wannier90 [31]. We note that it is also possible to solve the Kohn-Sham equation exclusively in the real space basis, using the so-called "all-electron" methods [32]. We now summarise some of the main features of employing a plane-wave basis code and truncation of the Hilbert space using MLWFs.

1. *Kohn-Sham eigenstates in the plane-wave basis*

Bloch's theorem in periodic systems can be applied to the solution of the full Hamiltonian of any system that can be written in the plane-wave basis. Similarly, so can the Kohn-Sham equations. In the plane-wave basis-set the Hilbert space is truncated by considering only a finite number of reciprocal lattice vectors $\mathbf{G}$. This is motivated by the fact that the kinetic energy is an unbounded operator that depends on the length of the reciprocal lattice vector, so a maximum energy sets a maximum value for the norm of $\mathbf{G}$ as $\frac{\hbar}{2m}|\mathbf{G}_{\text{max}}|^2 = E_{\text{max}}$. After diagonalization of the single-particle Hamiltonian, only a finite number of bands below and above the Fermi energy are retained. Moreover, as a consequence of Bloch's theorem, there is a natural partition of the degrees of freedom using the reciprocal lattice vectors (see Supplementary Figure 7). While the size of the system under study defines the number of inequivalent $\mathbf{k}$ points as seen in Eq. (13), the number of different reciprocal lattice vectors is unbounded.

The following cutoff,

$$\frac{\hbar^2}{2m}|\mathbf{G}|^2 \leq E_{\text{cut}}, \tag{83}$$

defines the total number of plane-waves used in the DFT calculation. Fewer plane waves can be used by replacing the core level Kohn-Sham states by an effective potential, famously known as the pseudopotential [24]. It is this crucial observation, i.e., that the core electrons do not participate in low energy, chemically relevant, excitations like their valence counterparts, that enables the success of the plane-wave method. Otherwise, the valence electron wavefunctions require impractically large number of Fourier components to remain orthogonal to all states within the core region, which are chemically inert. Thus, "freezing" the core states into an overall effective potential lifts this constraint, and allows the valence electron wavefunctions to be efficiently represented with far fewer Fourier components, without any nodes inside the core regions. Practically, this means that smaller matrices have to be diagonalized when solving Eq. (81), due to the reduced basis size. Pseudopotentials for atoms corresponding to the ions in the lattice are constructed by solving for all eigenvalues of their atomic wavefunction, fitting them to pseudo-wavefunctions and generating the corresponding atomic pseudopotential by solving all-electron atomic calculations for a single atom and then inverting the corresponding radial Schrodinger equation to find the effective pseudpotential. The constructed pseudopotential must reproduce the atomic properties of the element, i.e., the

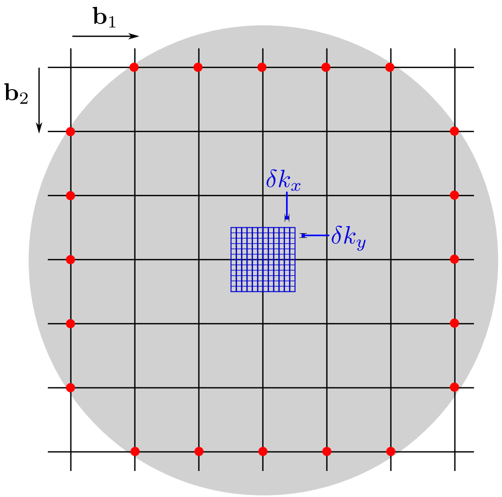

Supplementary Figure 7. Reciprocal lattice of a 2D square Bravais lattice. The black grid corresponds to the reciprocal lattice $\mathbf{G} = n_1\mathbf{b}_1 + n_2\mathbf{b}_2$, and it is unbounded ($n_a \in \mathbb{Z}$). The blue lattice represents the different lattice momentum states $\mathbf{k}$. Increasing the system size leads to a finer blue lattice, without affecting the black lattice. The red dots represent the maximum value of $\mathbf{G}$ inside a cut-off region, which is depicted by the grey circle.

scattering properties of the ionic potential, and agree with its true wavefunction outside of a cut-off radius away from the core. Moreover, it should be transferrable to a variety of chemical enviroments. For the materials used in this work, we use pseudopotentials from the pregenerated ONCVPSP library [33].

As a result, the actual value for the kinetic energy cut-off E_{cut} is usually chosen such that it corresponds to the minimum energy where the convergence of the total ground state energy does not change with respect to a specific tolerance, typically 1 meV per atom. In a material like silicon, a value used in DFT calculations [34] is $E_{\text{cut}} \sim 200$ eV. Using Eq. (83) this translates into $|\mathbf{G}_{\text{max}}| = \frac{2\pi}{a} N_{\text{max}}$ with $N_{\text{max}} \sim 5$. Depending on the pseudopotential used and the material under consideration, the total ground-state energy converges for larger cut-off energies. A cut off energy of 800 eV implies a doubling of the value of N_{max} from 5 to 10. From the perspective of the Kohn-Sham approach to DFT, in reciprocal space, the Kohn-Sham equations in second quantization are given by (see Eq. (24))

$$H^{\text{KS}}(\mathbf{k})\phi_{\mathbf{k},n}(\mathbf{r}) = \epsilon_{\mathbf{k},n}\phi_{\mathbf{k},n}(\mathbf{r}), \tag{84}$$

with

$$H^{\text{KS}}(\mathbf{k}) = \sum_{\mathbf{G},\mathbf{G}'} \left[\frac{\hbar^2|\mathbf{k}+\mathbf{G}|^2}{2m} \delta_{\mathbf{G},\mathbf{G}'} + U^{\text{eff}}_{\mathbf{G}-\mathbf{G}'} \right] f^\dagger_{\mathbf{k}+\mathbf{G}} f_{\mathbf{k}+\mathbf{G}'}, \tag{85}$$

where the effective potential $U^{\text{eff}}_{\mathbf{G}-\mathbf{G}'}$ also includes the contribution due to the exchange-correlation functional.

Additionally, before the optimal truncation of the active space occurs, the force on the ions created by the electronic charge of the electrons must be minimised. So far, in the spirit of the Born-Oppenheimer approximation, we have neglected the dynamics of the ions [3]. In particular, their positions $\{\mathbf{R}_I\}$ enter the Kohn-Sham equations of Eq. (81) as parameters, while their motion occurs on potential energy surfaces which are determined by the eigenvalues $\epsilon_i(\{\mathbf{R}_I\})$ of the electronic problem. At equilibrium, denoting by $\epsilon_0(\{\mathbf{R}_I\})$ the ground-state energy of the electronic system, the minimization of the force acting on ion I requires that

$$F_I = \frac{\partial \epsilon_0(\{\mathbf{R}_I\})}{\partial \mathbf{R}_I} < \delta \quad \forall I, \tag{86}$$

i.e., the ions' equilibrium positions are obtained from the minimization of $\epsilon_0(\{\mathbf{R}\})$, which is a function of $3N$ variables, and δ is the threshold value on the force all ions must satisfy. In the upper part of Supplementary Figure 8 we illustrate the typical workflow of a self-consistent DFT calculation, from calculating the external potential until self consistency is achieved in the electronic density and geometry.

2. *Truncation into an active space*

As stated in the main text, DFT breaks down for strongly correlated materials and, in many cases, even fails to provide a correct qualitative description [35]. Nevertheless, the resulting Kohn-Sham states have been successfully employed to build low-energy effective models of such materials [36]. The starting point is to notice that many materials' electronic and magnetic properties are determined by a few bands around the Fermi level. Therefore, an accurate description of such properties can obtained via an effective model, in which the dimension of the Hilbert space is reduced by truncating the Kohn-Sham eigenstates into a minimal representation within an active space of chemical interest. This is of particular relevance in such cases where the relevant bands around the Fermi level describe strongly correlated electrons, such as the ones occupying d or f orbitals. Here, an active space spanned by a limited number of the corresponding Kohn-Sham states can be selected, with the rest of the bands acting as an environmental mean field. This is the idea at the centre of the so-called quantum embedding theories, of which density matrix embedding theory (DMET) [37, 38] and dynamical mean field theory (DMFT) [36, 39] are two of the most prominent examples. Within these theories, particular care is required to decouple the active region from the environment in order to properly take into account the effect of the electrons occupying the inactive bands on the dynamics of the electrons in the active ones [40–42]. This mainly results in a screening of the Coloumb potential between electrons in the active region. A number of perturbative many-body approaches, such as constrained random phase approximation (cRPA) [40], have been developed to calculate the resulting screened Coulomb potential. In many cases, the decoupling between the active and inactive regions gives rise to non-negligible dynamical correlations, i.e., the screened potential for the electrons in the active subspace is in general frequency dependent. To lowest order, this dependence can be taken into account via a renormalization of the static hopping matrix and Coulomb tensor of the effective Hamiltonian [40–42]. These topics are at forefront of the research in material modelling and no general approach has proved to be sufficiently general and reliable. In this work, our focus is more on obtaining general estimates of the resources required to simulate materials on a quantum computer rather than giving an accurate description of each material. Therefore, we will neglect the effect of screening and we will employ a bare Coulomb interaction instead. This will result in denser hopping matrix and Coulomb tensor (see Supplementary Note 6 B 1 below), and therefore our estimates will provide an upper bound for the resources needed to simulate more realistic models of materials. In what follows, we will consider an active space consisting of the states around the Fermi level of a given material, as illustrated in Supplementary Figure 9. Fixing the number of bands below the last occupied band (including the latter) to be $n_<$ and the number of bands above it to be $n_>$, the dimension of the reduced Hilbert space $\mathcal{H}_{\text{red}}$ for a three dimensional material scales as

$$\text{Dim}(\mathcal{H}_{\text{red}}) \sim O(e^{(n_> + n_<)N_1 N_2 N_3 \mathcal{F}(x)}), \tag{87}$$

with $x = \frac{n_< - 1 + \nu_{\text{el}} - \lfloor \nu_{\text{el}} \rfloor}{n_> + n_<}$ and $\mathcal{F}(x) = -x \ln x - (1-x)\ln(1-x)$.

In addition to knowing the dimension of the Hilbert space, the orbital character of the individual quantum states is required for the chemical interpretation of the possible physical processes that can occur between these states. Once the relevant Kohn-Sham orbitals have been selected, this represents the basis of the fermion operator in the active space. In contrast, to generate Wannier functions, further classical computation has to be performed.

A crucial step to generating MLWFs is to provide an initial set of sensible projectors that reflect the orbital character of the Kohn-Sham plane-wave eigenstates. To achieve this, a local projection operator $\hat{\mathbf{P}}_i^{\mathcal{N}}$ is used, which projects onto the subspace $\mathcal{N} = \{\alpha, l, m\}$, where α is the principal quantum number, l is the azimuthal quantum number and m the magnetic spin quantum number

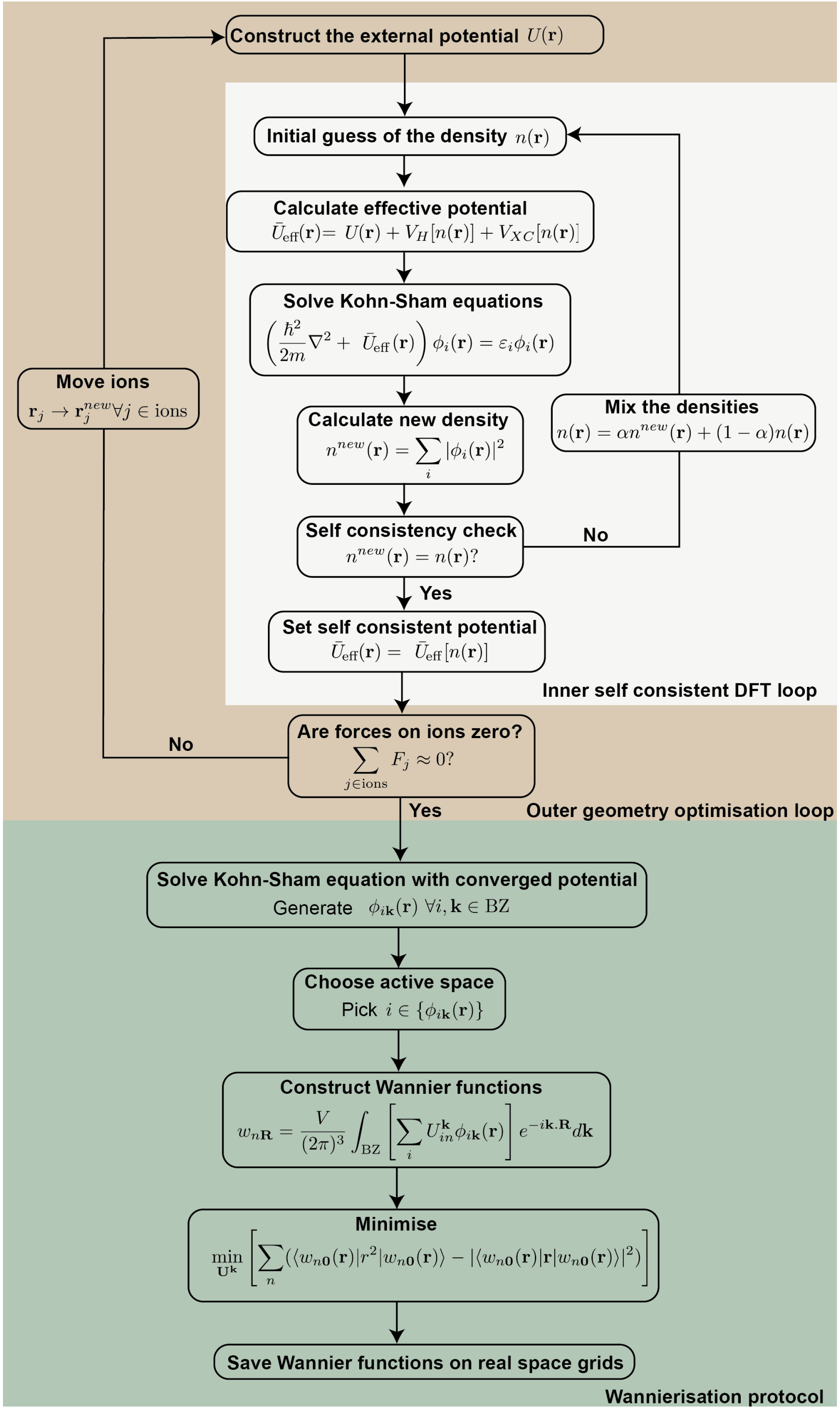

Supplementary Figure 8. Workflow (in yellow) of a typical Density Functional Theory calculation highlighting the inner electronic self-consistency loop and outer structural optimisation loop. We supplement this procedure with a Wannierisation protocol (in green) illustrating the steps required to transform from a plane-wave basis set to a maximally localised one.

centered at ion I [5]. Assuming a paramagnetic spin system, the local projection is given by,

$$\hat{\mathbf{P}}_I^{\mathcal{N}} = |Y_I^{\mathcal{N}}\rangle\langle Y_I^{\mathcal{N}}|, \tag{88}$$

where $Y_I^{\mathcal{N}}$ is the spherical harmonic centred at the centre of ion I and in the subspace $\mathcal{N}$. Therefore, for the Kohn-Sham eigenpair $(\epsilon_{\mathbf{k},n}, \phi_{\mathbf{k},n})$, the projected weight $p_{\mathbf{k},n}^{\mathcal{N}}$ is defined as,

$$p_{\mathbf{k},n}^{\mathcal{N}} = \langle \phi_{\mathbf{k},n}|\hat{\mathbf{P}}^{\mathcal{N}}|\phi_{\mathbf{k},n}\rangle = |\langle \phi_{\mathbf{k},n}|Y_I^{\mathcal{N}}\rangle|^2. \tag{89}$$

Subsequently, each Kohn-Sham eigenpair generates a set of weighted projections $\{(\epsilon_{\mathbf{k},n}, \phi_{\mathbf{k},n}, p_{\mathbf{k},n}^{\mathcal{N}})\}$ at each ion site I for the chosen subspace $\mathcal{N}$. Typically, for a given subspace, this weight is overlaid at each point in the band-structure as a colour gradient, and highlights the orbital character of all the Kohn-Sham eigenpairs in the chosen subspace. For example, $\mathcal{N} = \{2, 2, 0\}$ determines the $3d_{z^2}$ subspace, given by the spherical harmonic $Y_I^{2,2,0}$ centred at ion I. In Quantum Espresso, the axes of the spherical harmonics are orientated so that they aligns with the Cartesian axes.

Finally, MLWFs are then generated with the Wannier90 code [31]. The lower half of Supplementary Figure 8 summarises the protocol for producing MLWFs after the DFT calculation has been run, which takes as input the Kohn-Sham eigenstates and outputs Wannier functions on a real space grid.

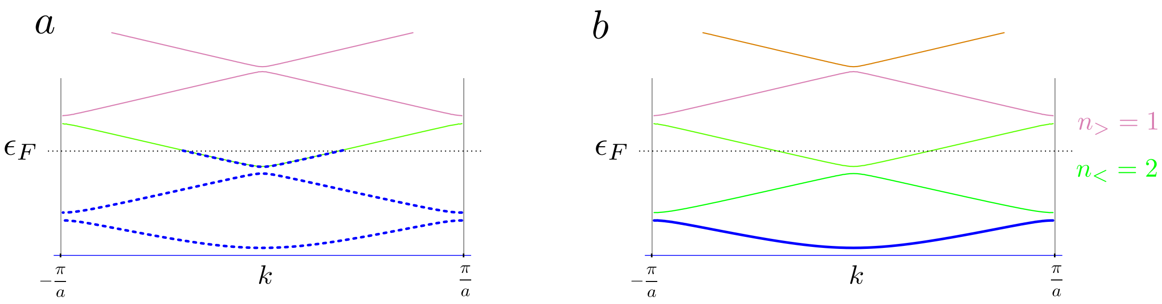

Supplementary Figure 9. **(a)** The number of electrons in the system defines the Fermi energy ϵ_F. In the non-interacting picture, the fermions fill the lowest single-particle energy levels, shown here as bands in the Brillouin zone, for a one dimensional system. The blue dots represent those energy levels occupied by fermions. In the figure, the lowest two bands are fully occupied, while the third band is partially occupied. **(b)** Instead of considering all the bands, we can define an active space, where the electrons will reorganize due to interaction effects. This active space is represented here by the states in the shaded area, around the Fermi level ϵ_F. In this example, the number of bands below (or crossing) the Fermi level is $n_< = 2$, while the number of bands above the Fermi level is $n_> = 1$.

So far we have discussed the construction of effective Hamiltonians starting from a material and reducing it to generate a Hamiltonian with the same general characteristics as the starting system (with the same symmetries, and of the same size in terms of unit cells). Another approach is to appeal to effective descriptions of physical systems, where a portion of the system is considered in a different footing than the rest. If one subsystem is small compared with the other, it is possible to replace the larger portion by an effective description in terms of a bath. This procedure is actually exact in the limit of lattices with infinite connectivity [43]. At the end of this procedure, a Hamiltonian that looks formally like Eq. (4) is obtained. The tools that we develop in the sections below work equally well for these systems.

G. Summary

Electrons in solids can behave in completely unexpected ways, depending on the ion composition and the interactions between electrons. Using a classically cheap zeroth order description based on

[5] These are the quantum numbers associated with the eigenstates of the angular momentum operator, and here are used as a basis for interpretability of the Kohn-Sham orbitals in therms of atomic or molecular orbitals

DFT, it is possible to isolate the relevant degrees of freedom that participate in a given phenomenon. From this description, we can construct a distilled Hamiltonian that contains the most important interactions and hopping terms within modes in the active space. We have also shown that a further compression is possible due to the structure of materials, where the thermodynamic number of degrees of freedom is encapsulated in a separation between bath and impurity modes in embedded approaches. Both strategies ultimately generate a Hamiltonian consisting of a restricted set of modes. This effective Hamiltonian is constrained by the symmetries of the system, and the same symmetries can be used to construct the Hamiltonian, reducing the classical cost of computation, but also limiting the possible interaction terms, thus reducing the overall complexity of the quantum circuits that implement the interactions. The relevant physics of the system is encoded in this compression.

In the following sections, we discuss in detail how to create a quantum circuit that implements the different terms of an effective Hamiltonian, with the goal of performing VQE or TDS. To achieve that, it is crucial to have an efficient way of representing fermionic degrees of freedom in terms of qubits.

Supplementary Notes 3 – Qubit representation

In order to represent a fermionic system on a QC, a mapping must be specified between the fermionic Hilbert space, and the multi-qubit Hilbert space of the QC. Such a mapping is most conveniently specified by a correspondence between fermionic operators and qubit operators. There are many design schemes available for such mappings [10, 20, 44–50], with significant room for variation in the details of their implementation. The most commonly used mapping is the JW transform, which maps fermionic creation ($c_i^\dagger$) and annihilation (c_i) operators to string-like qubit operators:

$$c_i^\dagger \leftrightarrow \left(\prod_{j<i} Z_j\right) \frac{(X_i + iY_i)}{2}\ ,\ c_i \leftrightarrow \left(\prod_{j<i} Z_j\right) \frac{(X_i - iY_i)}{2}. \tag{90}$$

The choice of mapping can have important consequences for the circuit depth and qubit requirements of TDS and VQE. Furthermore the way in which the mapping choice influences these costs will depend strongly on the structure of the given Hamiltonian, as well as the available hardware connectivity. Thus it is not obvious what the correct choice of mapping should be in general.

It is generally best to use a mapping which specifically maps the interactions (understood as both electron-electron interactions and hopping terms) present in the Hamiltonian to low-weight operators (i.e., operators that act non-trivially in just a small subset of the qubits, without scaling with the size of the system). The JW transform is not well equipped to do this in general. An example of a mapping which is better suited to this, and that we will make use of in this work is the Compact Encoding [10].

Unfortunately, in cases where there is a high degree of interaction between modes in the Hamiltonian, it is simply not possible to map all interactions to low-weight operators, regardless of the choice of mapping. In lieu of low-weight representations, a fswap network protocol may be employed, wherein fermionic modes are dynamically re-ordered throughout the algorithm, such that each interaction admits a low-weight representation at some point in the protocol. Such an fswap network amortizes the cost of performing high-weight interactions, at the expense of having to actively re-order the fermionic modes in the mapping. This amortization can be very powerful. Indeed, in the case where we want to implement all-to-all quadratic interactions it can be shown – under weak algorithmic assumptions – that fswap network methods in conjunction with the JW mapping can yield essentially optimal circuit depths (see Appendix C). More details about the fswap network protocol will be discussed in Supplementary Note 4 C and Supplementary Note 5 A.

To date, fswap networks of this kind have been employed exclusively in conjunction with the JW transform [11, 51, 52]. However, in principle, they may be used in conjunction with any fermion-to-qubit mapping, as the act of reordering fermionic modes admits a representation purely in terms of the fermionic algebra. Furthermore, in the case where a subset of modes have a high degree of

interactivity, fswap network protocols may be applied to this subset in isolation. This allows us to leverage the optimality of the fswap network protocol for all-to-all interactions, restricted to this subset where it is relevant. This suggests that a hybrid strategy may be ideal, wherein clusters of highly interacting modes are handled by an fswap network protocol, while any sparse connectivity is handled by a specific choice of mapping.

With this in mind, and for the purposes of comparison, we focus our attention on two basis choices for the material Hamiltonian: the Bloch basis Hamiltonian (Eq. (26)) and the Wannier basis Hamiltonian (Eq. (29)).

A. Bloch basis mapping

The Bloch basis Hamiltonian is given by (see Eq. (26)):

$$H^B = \sum_{\mathbf{k},n,\sigma} \epsilon_n(\mathbf{k}) f^\dagger_{\mathbf{k},n,\sigma} f_{\mathbf{k},n,\sigma} + \sum_{\sigma,\sigma'} \sum_{\substack{n_1,n_2,n_3,n_4 \\ \mathbf{k},\mathbf{q},\mathbf{k}'}} V^{(\mathbf{k},\mathbf{k}',\mathbf{q})}_{n_1 n_2 n_3 n_4} f^\dagger_{\mathbf{k}+\mathbf{q},n_1,\sigma} f^\dagger_{\mathbf{k}'-\mathbf{q},n_2,\sigma'} f_{\mathbf{k}',n_3,\sigma'} f_{\mathbf{k},n_4,\sigma}. \tag{91}$$

The quartic interactions in the Bloch basis Hamiltonian have no specific local structure – there are effectively interactions between every mode. This suggests that the best choice of mapping is the JW transform, in conjunction with an fswap network protocol.

B. Wannier basis mapping

The Wannier basis Hamiltonian (see Eq. (29)) is given by:

$$\begin{aligned} H^W = &\sum_{\sigma} \sum_{\substack{m,n \\ \mathbf{R}_1,\mathbf{R}_2}} T(\mathbf{R}_1 - \mathbf{R}_2)_{mn} w^\dagger_{\mathbf{R}_1,m,\sigma} w_{\mathbf{R}_2,n,\sigma} \\ &+ \sum_{\sigma,\sigma'} \sum_{\substack{s,l,m,n \\ \mathbf{R}_1,\mathbf{R}_2,\mathbf{R}_3,\mathbf{R}_4}} \tilde{V}^{(\mathbf{R}_1 \mathbf{R}_2,\mathbf{R}_3,\mathbf{R}_4)}_{slmn} w^\dagger_{\mathbf{R}_1,s,\sigma} w^\dagger_{\mathbf{R}_2,l,\sigma'} w_{\mathbf{R}_3,m,\sigma'} w_{\mathbf{R}_4,n,\sigma}. \end{aligned} \tag{92}$$

In contrast to the Bloch basis, the Wannier basis Hamiltonian has some local structure: interactions between modes indexed by $\mathbf{R}_1$ and $\mathbf{R}_2$ are suppressed as $|\mathbf{R}_1 - \mathbf{R}_2|$ increases. However, in the case where $|\mathbf{R}_1 - \mathbf{R}_2|$ is small, for example nearest neighbour or on-site, interactions are strong, and in general all-to-all with respect to the orbital index. In this case we make use of a hybrid strategy. First, as discussed in Supplementary Note 2 C 1, we label each site index $\mathbf{R}$ by the corresponding triplet of integers (n_1, n_2, n_3) (defined in Eq. (11)) on a Cartesian grid. Then, we map the system to an expanded compact encoding illustrated in Supplementary Figure 10 for a 2D material. In this mapping, all modes which share a common site index $\mathbf{R}$ are associated with a collection of qubits laid out in a JW style string, and each string is connected to nearest-neighbouring strings using the compact encoding design. Hybridizing encodings in this way is in general non-trivial, and so this construction, which allows for a variable number of modes per site, constitutes a genuinely new fermionic encoding specifically tailored to this task. This encoding is most concisely expressed in terms of "edge" (E_{ij}) and "vertex" (V_i) operators, which are defined as:

$$E_{jk} := -i\gamma_j\gamma_k, \quad V_j := -i\gamma_j\bar{\gamma}_j, \tag{93}$$

with Majorana operators

$$\gamma_j := w_j + w_j^\dagger, \quad \bar{\gamma}_j := (w_j - w_j^\dagger)/i, \tag{94}$$

where j (and k) is a multi-index over the site index $\mathbf{R}$, mode index m, and spin index σ. The edge and vertex operators are hermitian, they anti-commute when they share an index, and commute otherwise. Furthermore, the edge operators satisfy an important composition relation:

$$E_{ik} = iE_{ij}E_{jk}. \tag{95}$$

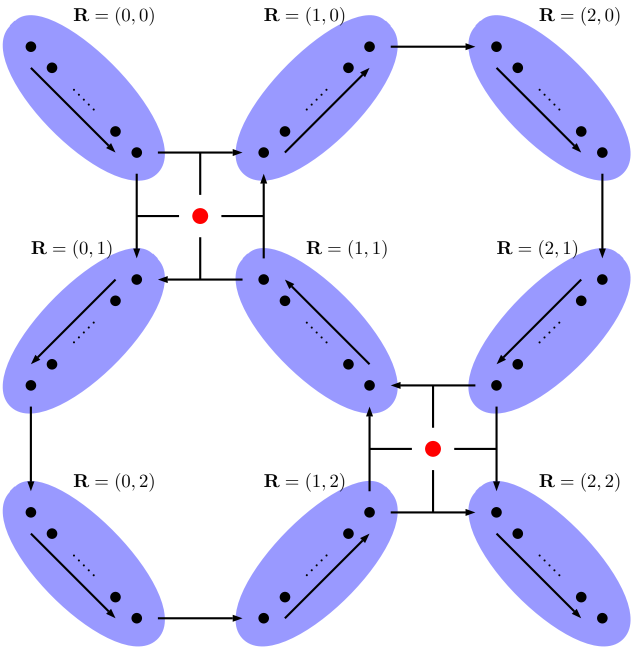

Supplementary Figure 10. Schematic for the hybrid fermion to qubit mapping for a 2D material. Black dots correspond to both fermionic modes and their corresponding data qubit. Red dots correspond to ancillary face qubits operating in the same fashion as in the square compact encoding [10]. Blue ellipses surround all fermionic modes assigned to a given site R. These modes are arranged in a line, with all interactions within the line taking the same form as in a JW transform with an identical linear ordering. Interactions between modes at the ends of neighbouring lines take the form of interactions between neighbouring modes in the compact encoding. Physically, each blue ellipse contains modes associated with the orbitals kept in a unit cell.

The edge and vertex operators can be combined to synthesize any fermionic terms with an even number of creation and/or annihilation operators, i.e., all observables that preserve parity superselection – a fundamental requirement of any realistic Hamiltonian. For reference we include these decompositions for quadratic terms:

$$w_i^\dagger w_i = (1 - V_i)/2,\ \ w_i^\dagger w_j = \frac{i}{4}(1 - V_i)(1 + V_j)E_{ij}, \tag{96}$$

$$w_i w_j = \frac{i}{4}(1 + V_i)(1 + V_j)E_{ij},\ \ w_i^\dagger w_j^\dagger = \frac{i}{4}(1 - V_i)(1 - V_j)E_{ij}, \tag{97}$$

$$w_i^\dagger w_j + w_j^\dagger w_i = \frac{-i}{2}\left(E_{ij}V_j + V_i E_{ij}\right). \tag{98}$$

Quartic terms may be constructed from quadratic terms; however, in this case, there is a freedom in the choice of decomposition into edge operators thanks to Eq. (95). This freedom may be used to choose a decomposition with the smallest qubit representation. Given that the fermionic encoding ultimately maps products of Majorana operators (Majorana monomials) to Pauli operators, it is most convenient to first decompose the fermionic Hamiltonian into the operator basis of Majorana monomials

$$H_M := \sum_{b \in \{0,1\}^{2M}} \alpha_b \prod_j \gamma_j^{b_{2j}} \bar{\gamma}_j^{b_{2j+1}}\ ,\ |b| \in \{2, 4\}, \tag{99}$$

before proceeding with mapping it to a qubit Hamiltonian by applying the encoding to each Majorana monomial. Here, M is the total number of complex fermion modes and is given by $M = N_{\text{modes/cell}} N_{\text{cells}}$, with $N_{\text{modes/cell}}$ and N_{cells} the number of modes per unit cells and unit cells in Eq. (92), respectively.

The precise details of how the hybrid mapping specifies the edge and vertex operators are given in Supplementary Figure 11. In the mapping, only certain edge operators are specified. Any other edge operators must be constructed using the composition relation in Eq. (95). Thus interactions between modes distant from one another on the graph geometry will decompose into products of edge operators, yielding string-like Pauli representations similar to JW strings. It is here where the fswap network protocol plays an important role.

This representation takes advantage of both the benefits of fswap network protocols in the context of all-to-all connectivity, which can be naturally applied to these JW style strings, and the benefits of the local structure manifest between sites, through the compact encoding. The efficacy of this approach will depend strongly on how localized the Hamiltonian interactions turn out to be for the particular material.

1. Stabilizers

Unlike the JW transform, the hybrid encoding represents fermionic states in a subspace of the multi-qubit Hilbert space. This subspace is best described as the code space of a stabilizer code [53]. The generators of the stabilizer code are given by the ordered product of loops of edge operators around the octagonal faces in Supplementary Figure 10, i.e., the faces with no ancillary qubit. An instance of one these generators is illustrated in Supplementary Figure 12.

The fact that the fermionic system lives in a code space means that state preparation incurs an additional overhead compared to the JW transform. This is discussed further in Supplementary Note 4 B. However one benefit these stabilizers do supply is error detection. We will not discuss that in detail in this paper. For some examples of this, see [10].

2. 3D Layout

For the simulation of the 3D bulk of materials there are two approaches one may take.

One approach is to collapse the 3D lattice of sites into a 2D lattice that matches the QC layout, with each site containing $N_{\mathrm{modes/cell}} * L_i$ modes, as illustrated in Supplementary Figure 13, where L_i is the side length you collapse. If the original 3D Hamiltonian has a nearest neighbour interaction structure on the Cartesian motif, then the collapsed 2D counterpart will also have a nearest neighbour structure. The downside of this approach is that it increases the depth of the fswap network protocol, since the number of modes on an individual site has increased. Additionally, it does not leverage the full sparsity of the Hamiltonian. The upside is that the qubits on superconducting devices are typically confined to a planar layout, and so this approach lends itself well to such devices.

The second approach is to employ a 3D generalization of the 2D compact encoding, as described in [20] to construct a 3D generalization of the hybrid encoding – illustrated in Supplementary Figure 14. The basic principle remains the same, however in this case the edge operators connecting the different sites are weight 4 instead of weight 3. The details of this construction are given in Supplementary Figure 15.

After constructing the map between fermion and qubit operators, we have all the preliminary ingredients to study the complexity of performing a quantum algorithm. In the next section we discuss the implementation of VQE and TDS algorithms.

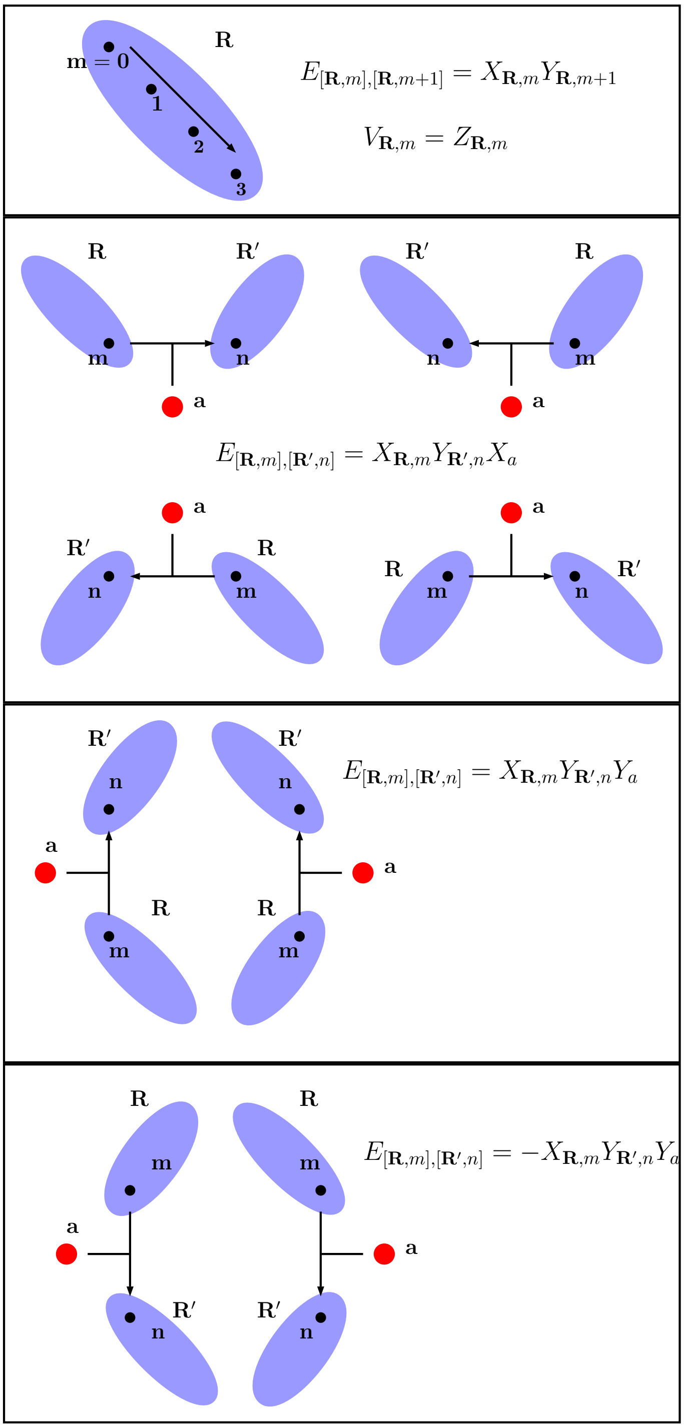

Supplementary Figure 11. Form of the edge (E_{ij}) and vertex (V_i) operators associated with the diagram in Supplementary Figure 10.

Supplementary Notes 4 – VQE and TDS algorithms

In this section we will find bounds on the complexity of implementing the VQE [54, 55] and TDS algorithms for materials' Hamiltonians. We begin by reviewing these algorithms before describing

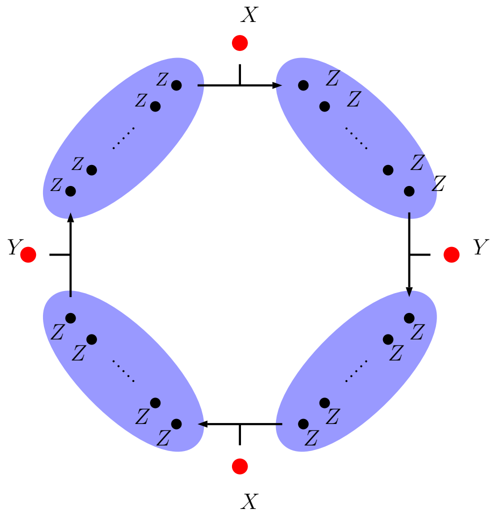

Supplementary Figure 12. Stabilizer of the hybrid encoding.

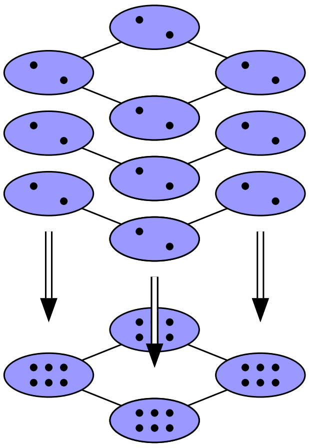

Supplementary Figure 13. Collapsing a 3D model to a 2D model

the details of how we implement and cost them for the systems of interest to us. We will use a simple model where all-to-all qubit interactions are allowed, arbitrary 2-qubit gates are cost 1 each, and 1-qubit gates are free. Our goal is to minimise the overall circuit depth.

Note that one could also aim to minimise the total gate count. Which metric is most appropriate will depend on the hardware platform being used and whether one is considering a near-term or fault-tolerant model. Assuming that the hardware platform allows gates to be implemented in parallel, minimising the quantum circuit depth can reduce the effect of decoherence as well as the wall-clock running time. Further, by a light-cone argument, quantum circuits with low depth experience less spreading out of local errors. In any case, our low-depth circuits are also efficient in terms of gate count.

VQE is a method which aims to produce the ground state of a quantum Hamiltonian, H, by optimising over trial quantum circuits picked from some family ("ansatz"), based upon the advance knowledge that such ansatz states should be able to represent the ground state effectively, and/or may be efficiently implementable on quantum hardware [54, 55]. Circuits from the ansatz have parameters which are optimised using a classical optimisation routine. This routine aims to minimise the energy with respect to H of the state produced by the quantum circuit. By the variational principle, if the ground state can be represented within the ansatz and is unique, minimising the energy output from parameterised circuit will lead to the circuit which prepares ground state. In

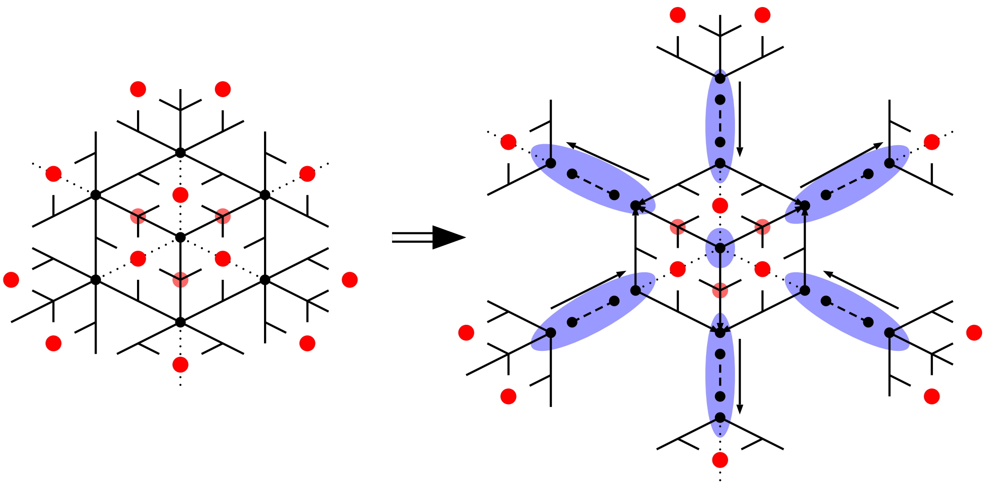

Supplementary Figure 14. (left) 3D compact encoding converted to (right) 3D hybrid encoding. Black dots correspond to both fermionic modes and their corresponding data qubits, and red dots correspond to ancillary qubits. Red dots are positioned on the faces of the cubes.

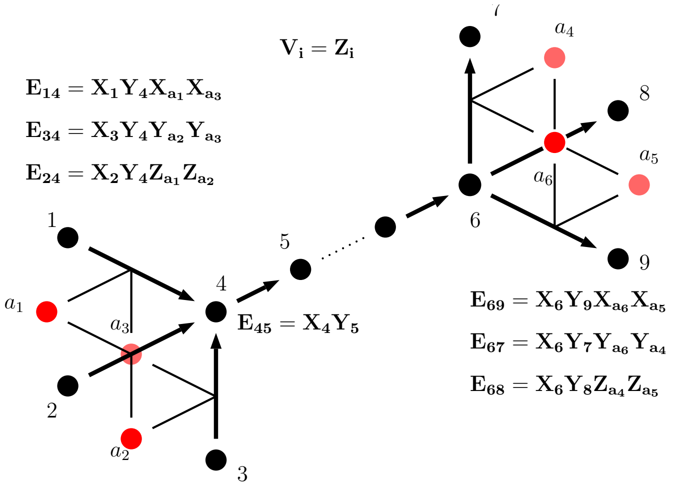

Supplementary Figure 15. The edge and vertex operators of the 3D hybrid encoding.

practice, the optimisation routine may find a local minimum rather than the global minimum. Once an approximation to the ground state has been produced, measurements can be performed to determine properties of interest.

Here we will use the Hamiltonian variational ansatz [2] within the VQE framework. This ansatz may be used to find the ground state of a Hamiltonian $H_M = \sum_k h_k$, where the terms h_k correspond to the nonzero Majorana monomials in Eq. (99). We assume that we can write $H_M = H_A + H_B$, where we have an efficient quantum circuit for preparing the ground state of H_A, and that the time-evolution operations e^{ith_k} can be implemented efficiently for all k and arbitrary times t (for example, h_k may act non-trivially on only a small number of qubits). Then we perform the following steps:

1. Prepare the ground state of H_A.

2. For each layer l, implement the operation

$$\prod_k e^{it_{lk}h_k},$$

for some parameters t_{lk}, to produce a state $|\psi\rangle$. Note that we will allow the ordering of the product to be arbitrary below, which can allow for more efficient algorithms.

3. Measure the energy of $|\psi\rangle$ with respect to H_M.
4. Optimise over the parameters t_{lk} to find the ground state (or a good approximation).

Here we will compute the complexity of step 1, and of one layer of step 2. We will also determine the number of measurements required to measure the energy of the ground state. To gain a full understanding of the complexity of VQE, it is also necessary to understand how many layers are required, and how efficient the optimisation process is, which we will not consider here; see [51, 56] for detailed numerical analyses of these points in the case of the Fermi-Hubbard model.

By contrast to VQE, the TDS approach corresponds to approximately implementing the unitary operation e^{-itH_M} for some t. The standard method for executing this operation is by Trotterisation, wherein for example e^{-itH_M} is approximated by a product of short time steps $(\prod_k e^{-i\delta t h_k})^L$, with $\delta t = t/L$. Simulating time-dynamics of a quantum system is theoretically more straightforward than finding a ground state (Bounded-error Quantum Polynomial time (BQP)-complete in the worst case, rather than Quantum Merlin Arthur (QMA)-complete [57]), yet may be more challenging in practice for near-term quantum computers, as the approximation may demand that the number of Trotter steps L be large. If an algorithm based on Trotterisation is used, TDS is very similar to implementing step 2 of the VQE algorithm. However, note that we have assumed in VQE that the time-evolution steps for each h_k can occur in arbitrary order, whereas for some more sophisticated Trotterisation methods we may want to fix a particular order for TDS. For the purposes of this analysis we assume the Trotterisation does not require a particular order on the sequence of terms – which is true in the case of first order Trotterisation. The only caveat is that we group together commuting terms in order to be able to compute improved upper bounds on the Trotter error. As in VQE, our analysis is for a single short time step. The circuit depth would then need to be multiplied by the desired number of time steps.

We now discuss how each of the above steps is implemented. The algorithm is based on a set of basic operations, and we begin by calculating their quantum circuit complexity.

A. Gate decompositions of operations

There are three types of operations used in our algorithm: time-evolution according to Majorana operators, fermionic swaps, and Givens rotations.

Time-evolution. Whichever fermionic encoding is used, each term of the Hamiltonian will ultimately be represented on the quantum computer as a string of Pauli operators. As 1-qubit gates are free and all Pauli operators are equivalent up to unitary conjugation, implementing a term reduces to implementing the operation $e^{i\theta Z^{\otimes k}}$, acting on $k \geq 1$ qubits, for arbitrary θ. This can be done in depth $2\lceil \log_2 k \rceil - 1$ via a circuit which uses a binary tree of CNOT operations to put the parity of the input state in the last qubit; performs a Z rotation on that qubit; and then performs the CNOT operations in reverse to uncompute the parity. We save depth 1 by combining the last two CNOTs and the Z rotation in one 2-qubit gate. See Supplementary Figure 16 for an example for the case $k = 4$.

Note that we may have multiple commuting terms acting on the same qubits, which can lead to efficiency savings by implementing time evolution according to these simultaneously. However, we do not consider this in our calculations.

Fermionic swaps. In the JW transform, fermionic swaps across adjacent modes are 2-qubit gates with cost 1. In the compact encoding, across most pairs of adjacent modes, the same holds. The exception is fermionic swaps across different material sites, where an ancilla qubit is involved. Thus it is most convenient to express the fermionic swap operator in terms of the fermionic algebra as

$$\mathrm{FSWAP}_{ij} = \exp\left(i\frac{\pi}{4}V_i\right)\exp\left(i\frac{\pi}{4}V_j\right)\exp\left(\frac{\pi}{4}E_{ij}V_j\right)\exp\left(\frac{\pi}{4}V_iE_{ij}\right), \tag{100}$$

The circuit depth may be computed by decomposing each of the terms in terms of Pauli matrices. However, here we can get an efficient decomposition for weight 3 edge operators by combining

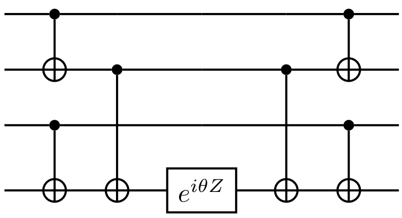

Supplementary Figure 16. The quantum circuit for implementing $e^{i\theta Z^{\otimes k}}$ in the case $k = 4$ in terms of CNOTs and single qubit rotations. The three middle gates can be combined into one 2-qubit operation, given total cost (2-qubit gate depth) 3 in our model.

operators as follows. The first two terms are single-qubit unitaries and hence free in our model. Written in terms of Pauli operators, the remaining terms are of the form

$$\exp\left(\pm i\frac{\pi}{4}(X_1X_2 + Y_1Y_2)P_A\right), \tag{101}$$

where P_A is a single qubit Pauli operator. We can diagonalise P_A on the ancilla with a local unitary, and can diagonalise $X_1X_2 + Y_1Y_2$ with a 2-qubit gate G, which turns out to be a Hadamard gate acting only on the odd parity subspace. We get

$$G(X_1X_2 + Y_1Y_2)G^\dagger = I - \frac{1}{2}(Z_1 + Z_2). \tag{102}$$

Therefore, we can implement the fermionic swap by a circuit of 2-qubit depth 4 (first apply $G_{12}^\dagger$ to switch to the correct basis for these qubits; then a gate across qubit 1 and the ancilla; then across qubit 2 and the ancilla; and then G_{12}). However, for the 3D encoding the edge operators are weight 4, and so we will not be using this decomposition in those cases, but rather the naive TDS decomposition.

Givens rotations. A Givens rotation is a unitary operation which mixes pairs of fermionic operators in the following way [58]:

$$\begin{pmatrix} \mathcal{G}_{ij}(\theta,\phi)c_i^\dagger \mathcal{G}_{ij}^\dagger(\theta,\phi) \\ \mathcal{G}_{ij}(\theta,\phi)c_j^\dagger \mathcal{G}_{ij}^\dagger(\theta,\phi) \end{pmatrix} = \begin{pmatrix} \cos(\theta) & -e^{i\phi}\sin(\theta) \\ \sin(\theta) & e^{i\phi}\cos(\theta) \end{pmatrix} \begin{pmatrix} c_i^\dagger \\ c_j^\dagger \end{pmatrix}. \tag{103}$$

Givens rotations are again most easily defined in terms of fermionic edge and vertex operators, which may then be translated into qubit operations using the chosen mapping:

$$\mathcal{G}_{ij}(\theta,\phi) = \exp\left(-i\frac{\phi}{2}V_j\right)\exp\left(i\frac{\theta}{2}[E_{ij} - V_iV_jE_{ij}]\right), \tag{104}$$

In the JW transform, when acting on adjacent modes this is a 2-qubit operation. In the compact encoding, it is usually a 2-qubit operation, except when acting across sites, when it is an operation of the form

$$e^{-i\frac{\phi}{2}Z_j}e^{i\frac{\theta}{2}(X_iY_j - Y_iX_j)P_A},$$

in 2D, and

$$e^{-i\frac{\phi}{2}Z_j}e^{i\frac{\theta}{2}(X_iY_j - Y_iX_j)P_AP_B},$$

in 3D, where P_A and P_B are single qubit Paulis on face qubits, specified by the orientation of the edge. By a similar argument to the fermionic swap operation (also see Appendix A of [51]), the 3-qubit operation can be implemented in 2-qubit gate depth 4. The 4-qubit operator can be implemented in 2-qubit gate depth 6.

As pointed out in the discussion at the beginning of this section, when using the Hamiltonian variational ansatz within the VQE framework usually one splits the Hamiltonian as $H_M = H_A + H_B$ and needs to prepare the ground state of H_A. Below we discuss how this can be done in the hybrid encoding.

B. State preparation

1. Fock states

The preparation of Fock states under the JW transform is straightforward, with each Fock state corresponding to a computational basis state, i.e.,

$$|b_0, b_1, ..., b_m\rangle \to \otimes_i |b_i\rangle \quad b_i \in \{0, 1\}.$$

However in the case of the hybrid compact encoding, the Fock state is encoded in a stabilizer code space, which necessitates a non-trivial state preparation procedure.

In the case of the 2D hybrid encoding, each stabilizer can be decomposed into a product of a classical parity check (product of Z operators) on the strings of data qubits $S_D = ZZZ...ZZ$, coupled to a four qubit operator $S_F = XYXY$ acting on the four face qubits (red ancillary qubits in Supplementary Figure 10). The set of stabilizers on the face qubits are identical to the stabilizers of the surface code, up to some local Pauli basis transformations [59, 60]. Thus if we wish to prepare a state where $S_D = 1$ for all data qubit stabilizers, such as the vacuum Fock state $|0\rangle$, then the algorithmic overhead of this state preparation is at most that of preparing a surface code state.

A given surface code state can be prepared either via a unitary circuit, or by measuring and correcting stabilizers. In the case of unitary preparation, a circuit of depth of $2\mathcal{L}$ is required, where $\mathcal{L}$ is the max side length of the surface code [61]. In the case of the hybrid encoding $\mathcal{L}$ depends only on the number of sites $\{N_1, N_2, N_3\}$, and not on the mode number. More specifically, $\mathcal{L} = \max(N_1, N_2)/2$ – assuming N_3 is collapsed into a single site, as described in Supplementary Note 3 B. Similar considerations hold in the 3D encoding, however the underlying code generated by the support of the face qubits is not as well studied. We defer comment on unitary state preparation in this fashion to a later date when we can present a more comprehensive understanding of this underlying code.

If one has access to intermediate measurements and classically conditioned circuit operations, then it may be more convenient to use a measure-and-correct scheme of preparation. In the case of preparing a surface code state the measure-and-correct scheme works by measuring stabilizers and performing depth-1 Pauli corrections conditioned on the outcomes of the measurements. These corrections appear as Pauli strings between pairs of syndromes [60]. However, in the case of preparing a Fock state, one does not need to prepare a surface code state, and may instead change the convention of the logical fermionic operations depending on the syndrome measurement. This gauge fixing saves having to actively perform the correction. An additional feature of encoding a fermionic Fock state is that the surface code stabilizers S_F can be measured without introducing any additional ancillary qubits. Instead, one may employ one of the data qubits and measure in the Z basis. After correcting or gauging out the syndromes, the Fock state prepared will depend on the syndrome produced, with a fermion occupying each data qubit which yielded -1 when measured in the Z basis. Finally we note that in the case of preparing a Fock state, the data qubits need not be measured in the stabilizer, since any stabilizer acts on these qubits with a Z operator. Thus the cost of coherently measuring the stabilizer is related only to its support on the ancillary face qubits. The reasoning described here applies in both the 2D and 3D encoding. In the 2D case the stabilizer on the face qubits is weight 4, while in the 3D case the stabilizer is weight 8.

Given an encoding of a vacuum Fock state $|0\rangle$, any other Fock state can be reached by applying a layer of single qubit Pauli operators (and vice-versa), in an analogous fashion to how any computational basis state can be reached from any other by a layer of single qubit X operators. To see this, we note that a product of Majoranas of the first kind (γ_i) applied to a vacuum state yields a Fock state (up to appropriate sign from normal ordering)

$$\prod_i \gamma_i |0\rangle = \prod_i w_i^\dagger |0\rangle ,$$

and any Fock state can be expressed this way. In the hybrid compact encoding, any product of Majoranas corresponds to a product of Pauli operators, which can be applied as a single layer of single qubit unitaries, each unitary being a single qubit Pauli operator.

The only caveat to the above discussion is that in some cases the hybrid compact encoding only represents even or odd parity Fock states. For example in the 2D encoding this happens when the number of non-trivial stabilizers is one more than the number of ancillary qubits, ie when the number of face qubits is less than half the number of faces. In this case the parity operator $\prod_i V_i$ is in the set of stabilizers and odd products of Majoranas do not admit a representation. However one may freely fix a convention for the parity of the encoding by changing sign conventions for specific edge operators. So one may prepare any Fock state by fixing the correct edge operator sign convention, thus fixing the parity sector, and then proceeding as described above.

Thus, using the methods outlined above, the minimal cost of preparing a Fock state is the circuit depth of coherently measuring the syndromes of the face qubit support of the stabilizers, followed by a depth-1 single qubit unitary operation – which executes any requisite corrections and performs the transformation from the vacuum Fock state to any other Fock state. With access to arbitrary two qubit operations a stabilizer of weight w can be measured coherently in depth $2\lceil\log_2(w)\rceil - 1$. The generating stabilizers in the 2D case can be measured in a sequence of 4 simultaneously measurable layers. This can be seen by noting that each face qubit has support on 4 generators. In the 3D case it is more challenging to determine the ideal decomposition since not every cycle of edges around a face need to be a generating stabilizer. However, the number of distinct face cycles that act on an individual face qubit is 10, so we may multiply the circuit depth by this number to get an estimate on the number of layers. Counting only two-qubit gates, the depth of Fock state preparation is thus 12 in the 2D case and estimated 50 in the 3D case.

2. *Fermionic Gaussian states*

Another commonly prepared class of input fermionic states are the fermionic Gaussian states, which include Slater determinants. These states appear as the ground states of non-interacting fermionic Hamiltonians (those containing only quadratic terms) and also as ansatzes for ground states of interacting fermionic Hamiltonians in classical methods. As such they can often serve as good input states for quantum algorithms such as VQE.

The best known methods for preparing Gaussian states are designed for JW representations of fermionic systems and involve sequences of Givens rotations $\mathcal{G}_{ij}(\theta, \phi)$ and Boguliubov transformations $\mathcal{B}$ [58]:

$$\mathcal{B} := \gamma_F \prod_{i \neq L} V_i, \tag{105}$$

$$\mathcal{B} c_L \mathcal{B}^\dagger = c_L^\dagger, \tag{106}$$

$$\mathcal{B} c_i \mathcal{B}^\dagger = c_i, \quad \text{for } i \neq L, \tag{107}$$

where here L is the index of the last mode in the choice of JW ordering. As discussed earlier, there can be cases where the hybrid compact encoding does not admit a representation of single Majoranas, in which case Bogoliubov transformations of this kind are not possible to implement, however one may always choose a parity sector and then restrict to the class of transformations that preserve parity.

For the JW transform, preparing Gaussian states requires circuit depth at most $M - 1$ using an algorithm of Jiang et al. [58] – where the number of modes $M = N_{\text{cells}} N_{\text{modes/cell}}$. In the case of the 2D and 3D hybrid compact encodings the scaling of the depth of the circuit for preparing a Gaussian state can not be much worse than that of the JW transform, because one can always overlay a JW ordering on the full hybrid compact encoding and perform the Givens rotations in accordance with the best known methods for the JW transform. However, in this case the hybrid encoding introduces some overhead due to the higher Pauli weight of some of the edge operators – namely those with support on face qubits – and reduced opportunities for parallelization – namely when attempting to perform Givens rotations on two pairs of modes whose edge operators have support on a common qubit. The algorithm of Jiang et al. acts on (at most) all consecutive modes at each step in an even-odd pattern. If we act on an even number of modes in total (as will always be the case if we consider spin) then at every other step, we only have 2-qubit gates. By Supplementary Note 4 A, the depths of the steps involving 3-qubit and 4-qubit gates are 4 and 6, respectively. To account for

overlapping action on face qubits we have to stagger the Givens rotations on those edges. Therefore, the overall depth is at most $2 * 4\lceil (M-1)/2 \rceil + \lfloor (M-1)/2 \rfloor \approx 4.5M$ for the 2D hybrid encoding and $2 * 6\lceil (M-1)/2 \rceil + \lfloor (M-1)/2 \rfloor \approx 6.5M$ for the 3D hybrid encoding.

C. Time-evolution according to terms in materials Hamiltonians

For both the VQE and TDS algorithms, we need to implement time-evolution according to the quadratic and the quartic parts of the materials' Hamiltonians, H^B or H^W. To do this efficiently, we will use a protocol based on fswap networks [11]. These networks use layers of fswap gates to rearrange the fermionic ordering and to enable terms to be implemented efficiently – for example, by moving modes to be adjacent in the JW transform. Here we apply fswap networks to more general fermionic encodings than the JW transform, and the notion of efficiency we will use is to apply operations across modes which are adjacent in terms of the encoding graph. That is, we will swap modes (using fswap operations on adjacent modes) with the intent of bringing modes across which we wish to perform some operation closer together.

Theoretical constructions of fswap networks are known which implement all quadratic [11] or quartic [9, 62] terms efficiently, in the sense that they reduce the quantum circuit depth by a factor scaling like the number of modes. That is, all quadratic terms on M modes can be implemented in quantum circuit depth $O(M)$, and all quartic terms can be implemented in quantum circuit depth $O(M^3)$. However, here we will want to apply fswap networks to specific materials' Hamiltonians that do not include all terms. We are therefore led to an algorithmic approach to produce an efficient fswap network protocol for a given Hamiltonian. We consider protocols that alternate between layers of the following form:

1. Fswap gates across modes that are adjacent with respect to the graph of the fermionic encoding that we are using. For example, in the JW transform, fswaps across qubits of the form $(i, i+1)$ would be allowed.

2. Time-evolution by all terms that are efficiently implementable given the current permutation of the graph of the fermionic encoding[6]. We consider a term to be efficiently implementable if there is a split of the modes on which it acts into pairs such that all pairs are adjacent within the encoding graph. For the fermionic encodings we use, such terms correspond to low-weight Pauli operators.

The aim is then to find a protocol using a small number of layers (corresponding to a good choice of positions for fswap gates), as well as implementing the interactions within each layer efficiently. We can achieve both of these using computational techniques, which we summarise here, with a more detailed description in Supplementary Note 5.

To find a good sequence of fswap gates, we use a greedy protocol. At each layer, we look at the set $\mathcal{T}$ of interactions t which have not yet been implemented, and define a distance function which measures the difficulty of implementing these interactions. Here we focus on ℓ_p distance functions of the form

$$D = \left(\sum_{t \in \mathcal{T}} d(t)^p \right)^{1/p}, \tag{108}$$

where $p > 0$ and $d(t)$ is the "distance" of a term t. This is defined as the minimum, over all splits of the modes into pairs, of the distance within the encoding graph of those modes. This is closely related to the number of fswaps required to bring these modes together. Empirically, we found that choosing $p = 0.5$ seems to produce good results. We believe that this is because choosing $p < 1$ puts greater weight on bringing terms with low distance closer together.

---

[6] Note that following this structure gives a "greedy" protocol where we implement all terms that are available at each step. One could also use a more incremental strategy where only some of these terms are implemented before the next layer of fswaps.

We consider swapping each possible adjacent pair of modes in the interaction graph, and compute D for each choice. If there exists a pair of modes which reduces D upon being swapped, we then fswap the pair and mark it as used. We repeat this process until all modes have been used, or there is no choice of modes to fswap that reduces D. A potential issue with this approach is that even in the first step, there may be no choice of pairs to fswap that reduces D. We can handle this by adding a fallback step where an fswap operation is chosen that at least reduces $d(t)$ for some term $t \in \mathcal{T}$.

To implement all terms efficiently in step 2 above, we express this problem in terms of graph colouring. We define a graph whose vertices are terms that should be implemented in the current layer, and where two vertices are connected if the corresponding terms can be implemented simultaneously. Here we take the simple view that two terms can be implemented simultaneously if they act on disjoint sets of qubits. Then the minimal number of colours required to colour this graph such that no two adjacent vertices have the same colour is the same as the minimal number of sublayers required to implement all the terms. As graph colouring is an NP-complete problem, we do not expect to be able to find the exact minimal number of sublayers for large numbers of terms and many qubits. However, we can use graph colouring heuristics to find an upper bound on the minimal number of layers efficiently. Here we use a greedy colouring algorithm with the DSATUR heuristic implemented in the NetworkX package [63].

As an additional optimisation, as the first step of our protocol we implement the fswap network of Kivlichan et al. [11], which enables all quadratic terms to be implemented using M layers of fswaps, for a system with M modes. During this process, we can also implement some other terms, if they happen to become efficiently available. As we expect the overall complexity to be significantly greater than M, this is a lower-order cost that can reduce the number of terms used substantially.

Finally, we need to decompose the operations we apply in terms of elementary quantum gates. For this we use the costing procedure described in Section 4 A.

We remark that recent work by Lao and Browne [64], on quantum circuit compilation for efficiently simulating time-dynamics of 2-local qubit Hamiltonians, follows a similar strategy of decomposing the overall quantum simulation in terms of alternating layers of swaps and implementation of time-evolution operations, implemented in an arbitrary order. Their work also aims to find “good” swaps that minimise a distance measure. As here we are instead simulating fermionic Hamiltonians, this leads to a different (though conceptually related) notion of distance. In particular, in our setting we need to handle quartic terms, whereas the 2-local terms in Lao and Browne’s work correspond to quadratic terms. Another point of difference is that these authors use a distance measure corresponding to reducing the distance of the “closest” term, rather than our distance measure that aims to track distance more globally, and hence to bring many terms closer together at once; also, here we introduce the use of a Steiner tree to reduce the size of the graph being considered, and use the “chain” swap network of [11] as a subroutine. See Supplementary Note 5 A 3 below for details.

D. Measurements

The final step of a variational quantum algorithm is to measure the energy of the quantum state produced, with the overarching goal of minimising this energy. One may also wish to measure some other operator to extract a physical property of the state. In this section we discuss how measurement can be achieved efficiently, in the rather general setting where one wishes to measure an arbitrary set of quadratic or quartic terms, expressed as Majorana operators.

We will focus on operators expressed in the JW transform. This also allows us to handle the case of the hybrid encoding with nearest-neighbour interactions between sites, because we can split terms into 4 groups (in 2D) or 6 groups (in 3D) that are only connected to nearest neighbours and locally look like Majorana operators in the Jordan-Wigner transform, with the exception of an ancilla qubit, which is always measured in the same basis. However, terms acting on next-nearest neighbours and beyond do not necessarily have this property.

Then a quadratic fermion term corresponds to Pauli strings of the form $AZZ \dots ZB$, where $A, B \in \{X, Y\}$, and the quartic case is either a product of two such strings on disjoint sets of qubits, or the product of a quadratic string and a Z operator elsewhere. In the two-string case, we can

assume that we only need to measure terms containing an even number of X's (or Y's), since our Hamiltonian has time reversal symmetry.

A naive measurement strategy would measure each term in sequence, using a number of measurements equal to the number of terms in the Hamiltonian, which can be at worst $\Theta(M^4)$ measurements for a quartic Hamiltonian on M modes. Our goal here will be to do better by measuring multiple terms at a time. Minimising the number of rounds may not always give the strategy requiring the minimal number of measurements to achieve a certain level of accuracy, as this also depends on the variance of each measurement [65]; however, this approach is a reasonable starting point. We also look for the measurements to be implemented using straightforward (ideally constant-depth) quantum circuits. To this end, we consider three types of measurement strategies:

- **(QWC)** Measuring qubitwise commuting terms simultaneously. These are Pauli terms which commute when restricted to individual qubits. This family of measurement strategies is easy to implement by measuring each qubit in the correct X/Y/Z basis, so requires only additional single-qubit gates. In the literature, this concept is sometimes called measuring in a tensor product basis (TPB) [66, 67].

- **(NC)** Measuring a family of *non-crossing* terms simultaneously. We say that a pair of distinct quadratic Majorana operators acting on modes $i \leq j$ and $k \leq l$ is non-crossing if either:

  1. $j < k$, or $l < i$, or $i < k \leq l < j$, or $k < i \leq j < l$;
  2. or $i = k$, $j = l$, and the endpoints of the two operators are picked from the set $\{XX, YY\}$, or the set $\{XY, YX\}$.

  A set T of Majorana operators is non-crossing if there exists a set S of non-crossing quadratic Majorana operators such that all operators in T are equal to a product of terms from S. A set of Majorana operators can be measured simultaneously in a simple way if they are non-crossing. This is because XX, YY, and ZZ commute and hence can be measured simultaneously (by a simple local transformation, corresponding to transforming to the Bell basis); the same is true for the set $\{XY, YX, ZZ\}$. So if all endpoints of all operators are either both contained within the Z-string of another Majorana operator, or avoid that operator completely, we can measure the terms simultaneously.

  An NC measurement protocol on M modes gives rise to a non-crossing matching on the complete graph with M vertices (see Supplementary Figure 17 below for some examples), where we apply a 2-qubit unitary to the endpoints of each edge in the matching, and then measure in the computational basis. Such a protocol allows us to measure all the corresponding quadratic terms (and hence their products) simultaneously.

  Explicitly, the protocol is as follows: to measure a set S of non-crossing quadratic Majorana operators, for each operator $O \in S$, measure the pair of qubits at the endpoints of O either in the basis with respect to which $\{XX, YY, ZZ\}$ are diagonal, or the basis in which $\{XY, YX, ZZ\}$ are diagonal, depending on the endpoints. If O has just one endpoint, measure it in the Z basis. Any remaining qubits are measured in the Z basis.

  This is a well-defined protocol, because by the non-crossing constraint, each qubit can be the endpoint of at most two distinct operators, which completely overlap and are jointly measurable. To show that it allows all of the operators in S to be measured, observe that the endpoints of each operator are measured in the correct basis, and the qubits between each endpoint are all measured in a basis that allows $ZZ \ldots Z$ to be measured. This is because each qubit is either measured in the Z basis directly, if it is not the endpoint of a quadratic operator; or is one of a pair of qubits measured in an entangled basis allowing ZZ to be measured (by the non-crossing constraint).

- **(COM)** Measuring a family of commuting operators simultaneously. Note that non-crossing operators are always also commuting, but the converse is not true. This is the most general approach we will consider, so it will require correspondingly fewer measurement rounds. However, the quantum circuits required to simultaneously diagonalise a set of measurement operators may be relatively difficult to implement (requiring depth $\Theta(M)$).

Strategy	Lower bound	Upper bound
**QWC**	$\frac{M^4}{16}$	$\frac{M^4}{3}$
**NC**	$\frac{2M^2}{3}$	$\frac{7M^2}{3}$
**COM**	$\frac{2M^2}{3}$	$\frac{5M^2}{3}$

Supplementary Table 2. Summary of the lower and upper bounds on the number of measurement rounds required.

Finding an efficient measurement procedure based on one of the above strategies corresponds to decomposing a set of Majorana operators into groups, such that each group only contains operators which are qubitwise commuting, non-crossing, or commuting (respectively). The number of groups corresponds to the number of measurement settings. Since in the commuting and qubitwise commuting cases a group of operators can be simultaneously measured if and only if all pairwise combinations can be, this is then a graph colouring problem and can be solved computationally for any given set of operators. The non-crossing case is similar if we first choose a decomposition of each term into a particular product of quadratic terms. Each vertex corresponds to a term, and two vertices are connected by an edge when they are incompatible with respect to one of the above strategies (i.e., act incompatibly on the same qubit, cross, or anticommute). Compatible terms can be measured in the same round. The number of colours required then corresponds to the number of measurement rounds. Often in the literature the complement graph (where edges correspond to compatible operators) is considered in which case the graph colouring problem is equivalent to the problem of finding a minimum clique cover, as discussed for example in [67].

We remark that, as mentioned above, when using the hybrid encoding it does not seem straightforward to apply the non-crossing condition for next-nearest-neighbour terms. These can be handled separately and measured using a QWC strategy.

1. *Previous work*

Each of the above families of measurement strategies has been studied previously.

Qubitwise commuting strategies were used in [66], and were studied using algorithms for solving graph colouring and minimum clique cover in [67]. We are not aware of any lower bounds in the literature for strategies of this form.

Commuting strategies have been studied as a graph colouring problem [68] and as a minimum clique cover problem [69, 70]. But the strongest results are in [71], where $\Theta(M^2)$ bounds are shown. We will discuss these bounds in detail and adapt the upper bound to the non-crossing setting.

Non-crossing measurements have been studied in different contexts (and with different terminology). Cade et al. used the concept of non-crossing measurements for the special case of the Fermi-Hubbard model [51]. This enabled energies to be measured using only 5 computational basis measurements. Hamamura and Imamichi [72] considered measuring general sets of Pauli operators given the ability to measure pairs of qubits in an entangled basis such as the Bell basis. They give an algorithm based on a greedy approach for finding pairs of qubits that are suitable for applying this method, and carried out numerical tests and experiments on quantum hardware to validate their method.

By restricting to measuring Majorana operators, here we obtain the advantage that the non-crossing condition defines joint measurability, via a simple (constant-depth) measurement strategy, without needing to fix the measurement in advance. This allows the problem of finding an efficient non-crossing strategy to be efficiently reduced to graph colouring, for which any desired approximate or exact algorithm can be used. This is because the inclusion of any term in a group uniquely specifies how the measurement of that term should be performed. By contrast, using more general Hamiltonians or measurement strategies, there does not seem to be such a unique specification and one seems to need to resort to a strategy such as trying each measurement operator in turn.

Next we will obtain analytical upper and lower bounds for each type of strategy in the case where arbitrary quartic terms are allowed in the Hamiltonian. These bounds highlight the differences between the strategies and show their worst-case behaviour. The bounds are presented in Table 2.

2. *Analytical lower bounds*

(QWC) We produce a set of $\Omega(M^4)$ quartic terms, each pair of which are not qubitwise commuting. Assume for simplicity that M is a multiple of 4.

We consider the set of quartic interactions acting on modes (i,j,k,l) such that

$$1 \le i \le M/4, \qquad M/4+1 \le j < k \le 3M/4, \qquad 3M/4+1 \le l \le M.$$

There are $\frac{M}{4}\binom{M/2}{2}\frac{M}{4} = M^4/2^7 - O(M^3)$ quadruples of this form, and so there are at least $8 \times M^4/2^7 = M^4/16$ corresponding quartic interactions.

We claim that any pair of interactions P_1, P_2 from this set are not qubitwise commuting. Consider a pair of interactions P_1, P_2 from this set acting on (i_1, j_1, k_1, l_1) and (i_2, j_2, k_2, l_2) respectively. If $(i_1, j_1, k_1, l_1) = (i_2, j_2, k_2, l_2)$, then they must act differently at one of i_1, j_1, k_1, l_1 in order to be distinct. So instead consider the case where $(i_1, j_1, k_1, l_1) \neq (i_2, j_2, k_2, l_2)$. Suppose $i_1 < i_2$, then at qubit i_2, P_1 acts as Z but P_2 acts as X or Y. The same argument applies if $k_1 < k_2$. And similarly, if $j_1 < j_2$ (or $l_1 < l_2$), then at qubit j_1 (l_1), P_1 acts as X or Y, but P_2 acts as Z.

(COM) In [71], it is shown that a maximal set of commuting quartic terms is of size at most $\binom{M}{2}$ (in the large M limit). Since we wish to measure $8\binom{M}{4}$ terms in total, any strategy will require at least

$$8\binom{M}{4}\Big/\binom{M}{2} = \frac{2(M-3)(M-2)}{3} = \frac{2M^2}{3} - O(M),$$

measurement rounds. This result also bounds the NC strategy as all non-crossing operators commute.

3. *Analytical upper bounds*

(QWC) There is a trivial upper bound of $8\binom{M}{4} = \frac{M^4}{3} + O(M^3)$ from the strategy of measuring each term in turn.

We can do marginally better by splitting $\{1,\ldots,M\}$ in half and doing the measurements that act only on $\{1,\ldots,M/2\}$ in parallel with the ones that act only on $\{M/2+1,\ldots,M\}$. There are $8\binom{M/2}{4} \approx \frac{1}{16}\frac{M^4}{3}$ terms that act only on $\{1,\ldots,M/2\}$, so the total number of measurements is $\frac{15}{16}\frac{M^4}{3}$.

We can iterate this procedure, by first measuring the $\frac{14}{16}$ fraction of the terms that act on both sides of $M/2$. Then, of the $\frac{1}{16}$ fraction of terms that act within the first half, do the $\frac{14}{16}$ fraction of terms acting across $M/4$ and so on. This gives a total number of measurements that asymptotically approaches:

$$\left[\frac{14}{16} + \frac{1}{16}\left(\frac{14}{16} + \frac{1}{16}\ldots\right)\right]\frac{M^4}{3} = \frac{14}{16}\frac{1}{1-\frac{1}{16}}\frac{M^4}{3} = \frac{14}{15}\frac{M^4}{3}.$$

(COM) An upper bound is provided in [71] for measuring all quartic interactions, including those that do not respect time reversal symmetry. Here we describe how the same method can be used to measure all the interactions we are interested in. Then we will show how to adapt this method into a NC strategy.

The abstract combinatorial problem that needs to be solved is the following. We say a list of disjoint pairs of a set is a *matching*. We need to construct a list of matchings of $\{1,\ldots,M\}$ such that each quadruple $\{a,b,c,d\}$ appears in a matching as the union of two pairs (i.e., there is a matching that contains either $\{a,b\},\{c,d\}$ or $\{a,c\},\{b,d\}$ or $\{a,d\},\{b,c\}$). We say that a set of matchings with this property *covers all quadruples*. Let $mq(M)$ denote the minimal number of matchings required to cover all quadruples in the set $\{1,\ldots,M\}$. To get all quartic fermionic terms as in [71], we can use this list of matchings as follows. Two quadratic Majorana terms $\gamma_i\gamma_j$ and $\gamma_k\gamma_l$ commute if and only if $i,j,k,l \in \{1,\ldots 2M\}$ are all distinct. A collection of commuting quadratic Majorana terms can be labelled by matchings of $\{1,\ldots,2M\}$. A list of matchings that covers all quadruples therefore corresponds to a list of measurement settings that measures all

quartic Majorana operators. The total number of measurements required is $mq(2M)$. If we are interested only in terms that respect time-reversal symmetry, we can instead use a list of matchings of $\{1,\dots,M\}$ that covers all quadruples. For each matching we do two measurements: (i) for each pair in the matching we measure the corresponding pair of qubits in the $\{XX,YY,ZZ\}$ basis, (ii) for each pair in the matching we measure the corresponding pair of qubits in the $\{XY,YX,ZZ\}$ basis. This results in a total of $2mq(M)$ measurements. Each matching contains $\lfloor M/2\rfloor$ pairs and so $\binom{\lfloor M/2\rfloor}{2}$ quadruples are covered by each matching. Since there are $\binom{M}{4}$ quadruples in total, we have a lower bound of $mq(M)\geq\binom{M}{4}/\binom{\lfloor M/2\rfloor}{2}\approx M^2/3$. It is shown in [71] that, when M is a power of 2, $mq(M)\leq 5M^2/6$. This therefore corresponds to an upper bound of

$$2mq(M)\leq\frac{5M^2}{3}.$$

Before moving on to discuss non-crossing strategies, we will first discuss the algorithm of [71] in more detail so that we can later adapt it into a non-crossing strategy. There are two important subroutines for creating matchings that contain certain pairs (which can be thought of as methods for measuring quadratic interactions). The first is a list of matchings to get all pairs of a set $\{1,\dots,M\}$ which is equivalent to finding an edge colouring of the complete graph. Baranyai's theorem [73] says that this takes $M-1$ colours if M is even and M colours if M is odd. The second subroutine is a list of matchings to get all pairs of the form $\{x,y\}$ where $x\in\{1,\dots,M/2\}, y\in\{M/2+1,\dots,M\}$. This can be done in $M/2$ matchings and corresponds to an edge colouring of the complete bipartite graph.

We now describe the algorithm when M is a power of 2. Split the set $\{1,\dots,M\}$ into blocks of size 2^n labelled by $B^n_m=\{m2^n<i\leq(m+1)2^n\}$. For each $n\in\{1,\dots,\log_2(M)\}$ do the following:

- Divide the set B^n_m in half (using the same notation, B^n_m splits into $B^n_m=B^{n-1}_{2m}\cup B^{n-1}_{2m+1}$). Generate a list of $2^{n-1}-1$ matchings of B^{n-1}_{2m} to get all pairs in B^{n-1}_{2m}. Extend each of these matchings with each of the $2^{n-1}-1$ matchings of B^{n-1}_{2m+1} that covers all pairs in B^{n-1}_{2m+1}. This gives a total of $<4^{n-1}$ matchings that covers all quadruples in B^n_m that consist of a pair in B^{n-1}_{2m} and a pair in B^{n-1}_{2m+1}. Do this in parallel for all m.

For each $n\in\{1,\dots,\log_2(M)-1\}$ do the following:

- Generate a list of $M2^{-n}-1$ matchings of $\{1,\dots,M2^{-n}\}$ that covers all pairs. For each of these matchings $\{\{a_1,b_1\},\{a_2,b_2\},\dots\}$, pair up $B^n_{a_i}$ and $B^n_{b_i}$.
  - Split B^n_a and B^n_b into half again and for $c,d\in\{0,1\}$ do the following: Generate a list of 2^{n-1} matchings that gets all pairs with one element of B^{n-1}_{2a+c} and one element of B^{n-1}_{2b+d}. Extend each of these matchings to a matching on $B^n_a\cup B^n_b$ in $2^{n-1}-1$ ways so that each pair in B^{n-1}_{2a+1-c} and each pair in B^{n-1}_{2b+1-d} is covered.

    This gives a total of $\leq 4\times 4^{n-1}$ matchings that cover all quadruples of the form (w,x,y,z) with $w\in B^n_a$, $x,y,z\in B^n_b$ and at least one element in both B^{n-1}_{2b} and B^{n-1}_{2b+1} (or similarly for $a\leftrightarrow b$).

The total number of matchings is therefore

$$\sum_{n=1}^{\log_2 M}4^{n-1}+\sum_{n=1}^{\log_2 M-1}M2^{-n}\times 4\times 4^{n-1}\leq\frac{5M^2}{6},$$

as claimed.

(NC) We want to adapt the method of the previous section so that all matchings are non-crossing. We first describe how to get all pairs in $\{1,\dots,M\}$ using M matchings. The jth matching pairs up $(i,j-i \mod M)$ for all i. Note that if $j-i \mod M=0$, the $i-$th mode is not paired. These matchings can be visualized as parallel lines on circles as shown in Supplementary Figure 17.

We can use this method in all the places in the previous algorithm where we need to generate all pairs within a subset B^{n-1}_{2m}. This takes 2^{n-1} non-crossing matchings, compared to $2^{n-1}-1$

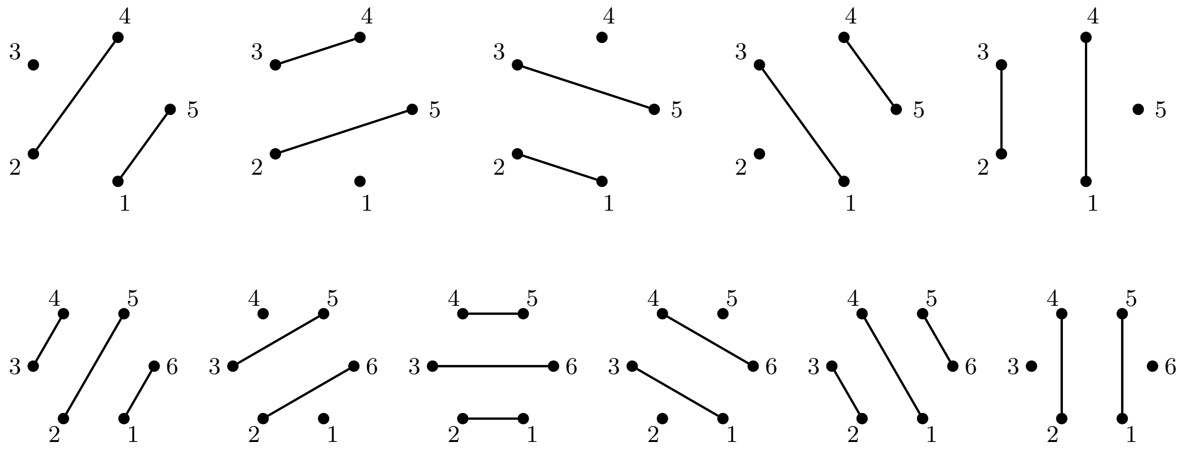

Supplementary Figure 17. M matchings that contain all pairs for (top) $M = 5$ and (bottom) $M = 6$. Each matching is a set of parallel lines on the circle.

matchings previously, making minimal difference to the total. We can also use this method when pairing up blocks B_a^n and B_b^n.

However, we do not have an alternative non-crossing strategy to get all pairs with one element in B_{2a+c}^{n-1} and one element in B_{2b+d}^{n-1}, which we were able to do with $2^{n-1} - 1$ matchings. Instead, we can just use our non-crossing strategy to get all pairs in $B_{2a+c}^{n-1} \cup B_{2b+d}^{n-1}$ using 2^n matchings.

If we do this, the total number of non-crossing matchings is

$$\sum_{n=1}^{\log_2 M} 4^{n-1} + \sum_{n=1}^{\log_2 M-1} M2^{-n} \times 4 \times 2^{n-1} \times 2^n \leq \frac{M^2}{3} + M^2 = \frac{7M^2}{6},$$

giving an upper bound of $7M^2/3$ non-crossing measurements.

4. Quartic terms that act on three modes

The strategy discussed in the previous section allows all quartic fermionic terms to be measured. However, we can get a more efficient protocol if we only want to measure terms that act non-trivially on only 3 modes. Physically, this corresponds to correlated hopping of the form $c_i^\dagger c_j n_k$ that may appear in the Hamiltonian. After the JW transformation, these are of the form

$$X_i \left(\prod_{i<l<k} Z_l \right) Y_j Z_k \quad \text{or} \quad Y_i \left(\prod_{i<l<k} Z_l \right) X_j Z_k,$$

where $i < k$ and $l \notin \{i, k\}$.

There are $2\binom{M}{2}(M-2) = M^3 - O(M)$ terms of this form, and we can measure all of them in $2M \log(M)$ non-crossing measurements as follows.

Assume for simplicity that M is a power of 2, $M = 2^m$. We can construct M non-crossing matchings such that all pairs occur in at least one matching, as shown in Supplementary Figure 17 and discussed in the upper bound for non-crossing strategies to measure all quartic terms in Supplementary Note 4 D 3. For each of these matchings, we choose $2\log_2(M/2)$ measurement settings, where for each measurement setting we measure each pair in the matching in either the $\{XY, YX, ZZ\}$ basis or the $\{ZI, IZ\}$ basis. We want to do this such that for any two pairs in a matching, say $\{a, b\}$ and $\{c, d\}$, there is a measurement setting such that $\{a, b\}$ is measured in the $\{XY, YX, ZZ\}$ basis and $\{c, d\}$ is measured in the $\{ZI, IZ\}$ basis (and vice versa). This can be done with a binary partitioning scheme. Explicitly, label each pair in the matching with a binary string $x \in \{0, 1\}^{m-1}$. For $j \in \{1, \ldots, m-1\}$, we have two measurement settings: one where the pair with label x is measured

in the $\{XY, YX, ZZ\}$ basis if $x_j = 0$ and in the $\{ZI, IZ\}$ basis if $x_j = 1$; and another where we do the opposite – i.e. we measure the pair labeled by x in the $\{XY, YX, ZZ\}$ basis if $x_j = 1$ and in the $\{ZI, IZ\}$ basis if $x_j = 0$. If we have two pairs with labels x and y, then if x and y are distinct, they must differ in at least one bit, and so there is a measurement setting where they are measured in different bases.

E. Summary

In this section, we have discussed all the algorithms needed to efficiently simulate a material Hamiltonian in a quantum computer. Based on these ideas, we have built a compiler that is able to perform the different decompositions of terms into layers of quantum gates, thus allowing us to explore the circuit depth associated with different materials. In the next section we explain the structure and design of this compiler.

Supplementary Notes 5 – Circuit compiler design

Here we outline the structure of the compiling algorithm which we use to compute the depth of the circuit resulting from applying either TDS or VQE to a given material. The compiler computes the circuit depth of a Trotter step in TDS, or an ansatz layer in VQE, both of which have similar structure. The methods in the compiler can be separated in two main steps.

Precompilation step: The precompiling step prepares the requisite data for the circuit compiler. It takes as input a fermionic Hamiltonian, a specification of which modes are associated with which sites, and a specification of the spatial layout of the sites (e.g., Hamiltonian constructed in Supplementary Note 2). Depending on user specification, the compiler builds the appropriately sized 3D or 2D hybrid encoding, and assigns modes in the encoding so that all modes on the same site are grouped together on the same JW chain in the hybrid encoding, or if one is representing a 3D system on a 2D encoding, it collapses one axis down into a single site before assigning modes. See discussion in Supplementary Note 3.

Compiling step: In the compiling step the terms in the Hamiltonian are organized into a sequence of groupings via a series of decomposition subroutines. Starting with a single grouping, every decomposition subroutine decomposes each grouping into smaller groupings. Next, the terms are translated into Pauli operators and undergo a final decomposition. Finally the circuit cost for each grouping is totalled.

The decomposition subroutines are

- **Common sites**: Groups together terms which act on exactly the same set of sites.
- **Mutually commuting terms**: Groups together mutually commuting terms, optimizing for the smallest number of groups using a pre-supplied graph colouring algorithm.
- **Fswap network**: Finds an optimized sequence of fermionic swaps on the modes of the encoding. Groups together any terms with sufficiently low-weight Pauli representation at every step of the fswap sequence. Optionally all modes are put back in their original location at the end of each sequence of fswaps. See subsection 5 A for more details.
- **Disjoint qubits**: Groups together terms which act on different sets of qubits, optimizing for the smallest number of groups using a pre-supplied graph colouring algorithm.

The choice of ordering and inclusion of particular decomposition routines will depend on a number of considerations:

- The decomposition into common sites is primarily useful when compiling the unit cell of a translationally invariant system (see subsection 5 B), and may be excluded otherwise.
- The inclusion of a decomposition into mutually commuting terms is important for evaluating the Trotter error of a particular TDS circuit and may be excluded when considering VQE.

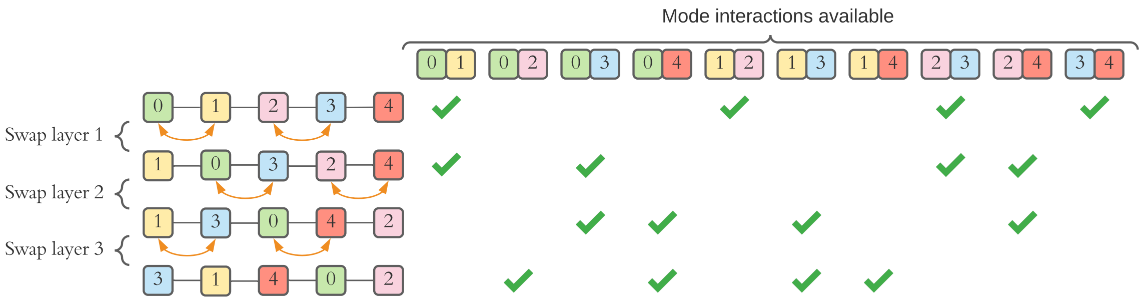

Supplementary Figure 18. Fswap network example for a five-mode linear system, where we require all-to-all interactions, i.e., that each mode should be adjacent to all other modes at least once. The chain on the left represent the configuration of the system, and the table on the right indicates whether a given interaction (between a pair of modes) is available at that configuration. Green ticks indicate that the pair is available on the present configuration; by the end of the third layer, all required interactions have been implementable at least once. Fswap layers on the mode configuration (left) are ordered: the first layer performs fswaps on even-indexed edges, i.e., the 0th and 2nd of the chain; the second layer performs fswaps on odd-indexed ($1^{\text{st}}, 3^{\text{rd}}$ edges), in a repeating pattern until either (i) all required interactions have been implemented or (ii) the chain has completely reversed after m fswap layers.

Depending on the importance of minimizing Trotter error, it may appear either before or after the fswap network routine.

- Generally it is useful to include the fswap network routine, but in some cases the density of long range terms may be sufficiently small that the fswap network does not improve circuit depths, so it may be worth checking the compilation with and without the fswap network.

A. Fswap network implementation details

With respect to circuit depth, when implementing an interaction between two modes, it is preferable that they are adjacent within the fermionic encoding, as outlined in Supplementary Note 4 C. By swapping modes through the graph of the encoding, we can ensure that modes which share an interaction will become adjacent at some point, in almost all cases. In the case where the modes are arranged locally on a graph as opposed to a chain, this is not guaranteed, but can be obtained in practice, as we will describe in Supplementary Note 5 A 2. Fswap operations can be performed efficiently on modes which are adjacent, i.e., which share an edge in the encoding (see Supplementary Note 4 C), and fswaps can be performed in parallel provided no mode is involved in more than one fswap. We therefore design an fswap network, i.e. a series of fswap layers interspersed with layers of interactions which are facilitated by the intermediate mode configurations.

In particular, we seek the fswap network that enables implementation of all terms using the smallest number of fswap layers. After each fswap layer we implement all allowed terms (those on adjacent modes) before proceeding to the next fswap layer. This does not necessarily give the lowest quantum circit depth overall, but is a reasonable heuristic. The compiler is equipped with two routines for determining the fswap layers required to facilitate interactions, which we now introduce.

1. *Chain fswap network*

The chain fswap network, as described in [11], is applicable in the case where all relevant modes are arranged linearly, i.e., in a JW string of length m modes (equivalently, m data qubits). Fswap layers iteratively perform fswaps on even-indexed edges, followed by odd-indexed edges. This mechanism ensures that every mode-pair is achieved within m fswap layers.

For example, the fswap network in Supplementary Figure 18 aims to implement a set of all-to-all interactions among five modes, where each mode interacts with every other mode in the system. This can be described as the set of layers in Supplementary Table 3, which must then be translated into circuit gates.

Layer type	Modes to interact/fswap
Interaction	$(0,1),(1,2),(2,3),(3,4)$
Fswap	$(0,1),(2,3)$
Interaction	$(0,3),(2,4)$
Fswap	$(0,3),(2,4)$
Interaction	$(0,4),(1,3)$
Fswap	$(1,3),(0,4)$
Interaction	$(0,2),(1,4)$

Supplementary Table 3. Circuit layers corresponding to the fswap network in Supplementary Figure 18.

2. *Distance minimising fswap network*

For a more general *mode graph* where, for instance, the modes are not arranged linearly, we do not have such a straightforward strategy. Moreover, the chain fswap network does not guarantee that quartic terms will all be facilitated: quartic terms consist of four Majorana indices, and therefore require that two mode-pairs are adjacent simultaneously, even if the two pairs are distant from each other on the encoding graph. The chain fswap network ensures that every pair will be adjacent, but not that every set of two pairs will be simultaneously adjacent, as required. Instead, given a set of required interactions, we devise an fswap network which seeks to find the optimal set of fswaps at each fswap layer, in order to facilitate as many interactions as possible on the subsequent interaction layer. That is, the distance of a mode graph G with respect to the set of interaction terms required, $\mathcal{T}$, can be evaluated as

$$D(G) = \left(\sum_{t \in \mathcal{T}} d(t|G)^p \right)^{1/p}, \tag{109}$$

where $d(t|G)$ is the cost of a single term t evaluated with respect to the graph G, and $p > 0$ is a hyperparameter described in Eq. (108) of Supplementary Note 4 C. The cost of each term is evaluated as the path length between the modes involved in the term,

$$d(t|G) = G.\texttt{path\_length}(t). \tag{110}$$

Then, a proposed fswap s will result in a modified graph $G(s)$, yielding

$$d(t|G,s) = G(s).\texttt{path\_length}(t). \tag{111}$$

The edge list of G – labelled $\mathcal{E}$ – defines the set of permitted fswaps for G; we can evaluate the cost of each available fswap independently, giving $\{D(G(s))\}_{s\in\mathcal{E}}$. The edge which results in the lowest cost, s_1, is added to the present fswap layer, $\mathcal{S} = \{s_1\}$. However, we can perform numerous fswaps in the same layer, provided no mode is involved in more than one swap operation. We therefore evaluate the effect of swapping the remaining available edges, i.e. compute the set $\{D(G(s))\}_{s\in\mathcal{E}\setminus s_1}$, and add the best fswap from this set to $\mathcal{S}$. This process is repeated until there are no remaining available fswaps, or it is no longer advantageous to include more fswaps, i.e., the cost of the proposed graph would increase from any available fswap. Supplementary Figure 19 shows the calculation of $d(t|G,s)$ for each $s \in \mathcal{E}$ for a small graph which requires a single interaction $\mathcal{T} = \{(0,4)\}$. In this example the fswap network is seeking an interaction between modes $(0,4)$, and finds that a single fswap layer $\mathcal{S} = \{(0,1),(2,4)\}$ facilitates the sole required interaction.

This distance minimising fswap network mechanism can be varied by defining an alternative total distance function, Eq. (109), or internal distance function, Eq. (110). Furthermore, in realistic use

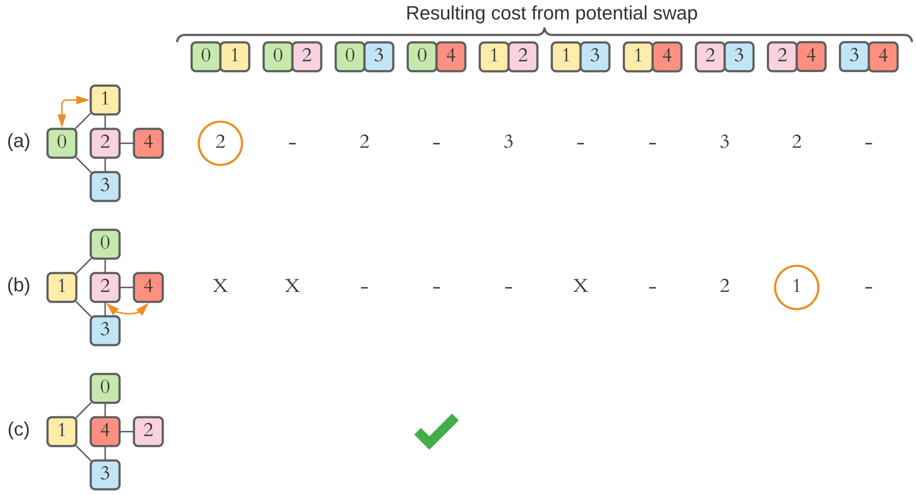

Supplementary Figure 19. Distance minimising fswap network: determining the set of fswaps to apply in order to facilitate an interaction between modes $(0, 4)$ on a small mode graph. Mode graphs (left) represent modes as squares, while lines between modes indicate an edge, i.e., a connection in the fermionic encoding. In the initial configuration **(a)**, the shortest distance between modes $(0, 4)$ is 3, e.g., the path via edges $\{(0, 1), (1, 2), (2, 4)\}$. Iteratively, (i) available fswaps $s \in \mathcal{E}$ are evaluated, i.e., $d(t|G, s)$ is computed $\forall s \in \mathcal{E}$, reported as a path length in the table of the graphic; (ii) one of the the fswaps which yield the lowest cost within the round (circled, orange) is added to the fswap layer $\mathcal{S}$. Dashes indicate that such an edge is not present in the graph. **(b)**, The available edges are again evaluated and the best option is identified as the swap between modes (2,4); `X`s indicate that one of the modes in the proposed fswap has already been used and is therefore unavailable. **(c)**, $G(\mathcal{S})$, the mode graph after the fswap layer has been applied, showing the interaction $t = (0, 4)$ is available (green tick).

cases (see, e.g., Supplementary Note 6 A), the distance function will be based on Majorana indices, of which two reside on each mode. Such terms are implementable if both Majoranas are on the same mode, or their corresponding modes are adjacent. Such considerations can be built into the design of $d(t|G)$. In particular, the swap network can be designed to handle quartic interaction terms by specifying an internal distance function which is minimised when the graph permits the implementation of the quartic term.

3. *Composite fswap network*

The method described in Supplementary Note 5 A 2 ought to produce a shorter fswap network overall, and is applicable for all mode graphs including chains. However, it is much more expensive to compute than the chain swap network, owing to the requirement to evaluate the cost of all remaining $t \in \mathcal{T}$ for each $s \in \mathcal{E}$, on potentially large graphs G. It is therefore preferable to rely on the chain fswap network of Supplementary Note 5 A 1 when possible, which guarantees that all quadratic (and some quartic) interactions will be implementable within m fswap layers (for a chain of length m modes). The cases in which the chain method do not suffice are:

- the mode graph of the system is not linear;
- some quartic interactions are not made implementable, i.e., when multiple edges are required to implement a single term, the chain method does not guarantee they will occur concurrently.

In practice then, we compose an fswap network from one or both of the above methods, depending on the required interactions and graph structure. Also note that the fswap networks we wish to construct will likely exist in a larger mode graph than they require. For instance, the entire system under study contains M modes across several sites, but we are interested in simulating only a subset of $m < M$ modes, such as the interactions contained on single physical site. In these cases, it is sensible first to reduce to the minimal graph which supports the m modes we require to implement

the interactions in $\mathcal{T}$. This can be done by finding the Steiner tree of the mode graph containing all the modes involved in any $t \in \mathcal{T}$: this can include some modes which are *not* directly involved in any interaction, but which reside in between modes of interest. We can compute the fswap network only upon this subgraph, but in practice the swaps will be applied in the space of the full graph.

We employ the following strategy for a given mode graph G and interactions $\mathcal{T}$.

1. Isolate the modes $\mathcal{M}$ which are involved in *any* interaction in $\mathcal{T}$:
   (a) construct a Steiner tree consisting only of $\mathcal{M}$, i.e., a subgraph of G;
   (b) if the Steiner tree has fewer modes than G, it will require fewer fswaps to traverse, so replace G with the Steiner tree.
2. If G is semi-Eulerian, i.e., there is a path containing each edge once (equivalently, a chain encompassing all modes):
   (a) form a new graph from the Euler path edges of G, and replace G;
   (b) run the chain fswap network on G for $\mathcal{T}$, resulting in the fswap network S_1;
   (c) throughout S_1, the terms $\mathcal{T}_1 \subseteq \mathcal{T}$ were implementable
      i. if the initial $\mathcal{T}$ contains only quadratic terms, $\mathcal{T}_1 = \mathcal{T}$, and the fswap network composition can terminate;
      ii. in general, some terms remain, so replace $\mathcal{T}$ with $\mathcal{T} \setminus \mathcal{T}_1$.
3. Again, attempt to reduce G to a Steiner tree based on the required interactions $\mathcal{T}$.
4. Pass $G, \mathcal{T}$ to the distance-minimising fswap network to produce S_2.
5. Combine the generated fswap networks into a single fswap network, $S \leftarrow \{S_1, S_2\}$.
6. Restore the initial mode graph G
   (a) After all fswap and interaction layers of S are implemented, reverse all fswaps in S so that the final configuration matches the initial configuration. This is necessary to facilitate the circuit tiling described next in Supplementary Note 5 B.

B. Compiling unit cells of translationally invariant Hamiltonians

Although the compiler is capable of handling a fully populated multi-site material Hamiltonian, it is usually more efficient to perform the compilation on a unit cell and its neighbours. This introduces a few subtleties which are worth explaining.

The premise behind performing the compilation on a unit cell is that a judicious choice of groupings of terms in $\mathcal{T}$ onto common sites will yield a circuit depth that reflects the circuit depth of the fully populated system. This holds when each grouping of terms onto common sites can be uniformly tiled onto the full system in a parallelizable fashion, so that the circuit depth of each grouping is the circuit depth of the full tiling. This is illustrated in Supplementary Figure 20 and Supplementary Figure 21 for nearest neighbour terms.

Because the fermionic encoding does not have exactly the same translational symmetry as the material, some care needs to be taken in how the groupings are ultimately tiled, and how the modes are ordered in the encoding. In particular, the choice of tiling needs to ensure that two parallelized groupings are not using the same ancillary face qubits at the same time. Furthermore, the modes in the encoding of the unit cell need to be ordered in such a way that their relative positions within a grouping are consistent throughout the tiling of that grouping.

In the case of next nearest neighbour terms, one can always consider a larger cell which includes more sites, however again care should be taken in the precompiling stage to remove any terms that would be double counted under tiling, to ensure that mode ordering is consistent, and to ensure the terms are grouped in a way that can be uniformly tiled. Currently the compiler is not equipped to handle this case, and so instead any terms that are beyond nearest neighbour are separated out and used to populate a complete lattice, described in Supplementary Note 5 B 1. The remaining compiler routines are then applied to this remaining collection of terms. Unfortunately this yields sub-optimal circuit depths and can be quite computationally taxing. Further improvements on the

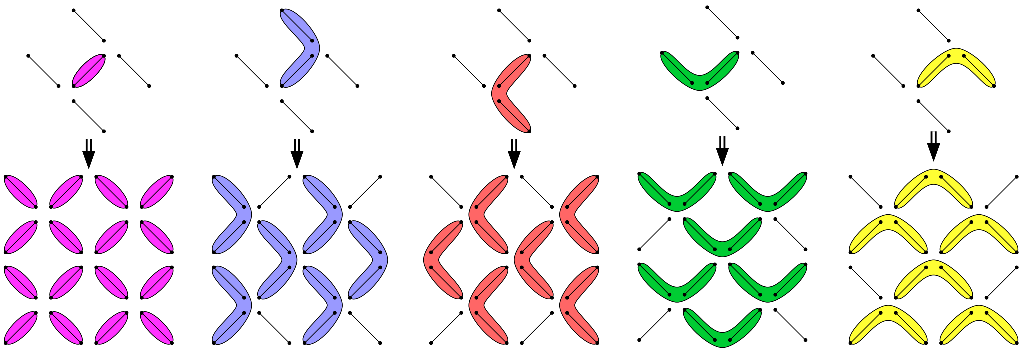

Supplementary Figure 20. An illustration of how the common site decomposition on a unit cell translates to the full system for nearest neighbour interactions on the 2D hybrid encoding.

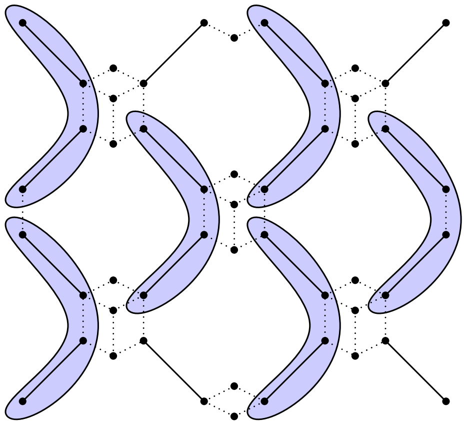

Supplementary Figure 21. An illustration of how one of the possible common site decomposition on a unit cell translates to the full system for nearest neighbour interactions on the 3D hybrid encoding. Here the tiling is done in the orthographic plane.

logic regarding tiling are currently in development and we expect circuit depths on terms beyond nearest neighbour to significantly improve after these changes.

1. *Compiling beyond-nearest-neighbour terms*

As detailed in Supplementary Note 5 B, terms beyond next nearest neighbour, denoted NNN+, must be included explicitly in the set of terms used to cost the overall circuit corresponding to the lattice $\mathcal{L}$. That is, the subset of terms $\mathcal{T}_{\text{NNN+}} \in \mathcal{T}$ must be repeated throughout $\mathcal{L}$ wherever they are compatible, i.e. wherever they do not exceed the boundaries of $\mathcal{L}$. Starting from a term $t \in \mathcal{T}_{\text{NNN+}}$, which by construction involves the central site $s_0 \in \mathcal{L}$, we attempt to tile t upon $\mathcal{L}$ by the following steps:

- Determine the structure of t, i.e., the sites and orbitals involved in the interaction, and their relative positions.
- For each site $s \in \mathcal{L}$, construct a new term t' retaining the same structure of t by translating all the sites of t by the vector $\mathbf{v} = s - s_0$.
- if t' extends beyond the lattice, i.e., it involves a site $s' \notin \mathcal{L}$, it is discarded. In other words, the Hamiltonian is truncated to terms completely contained in $\mathcal{L}$. For instance let us consider the following case:
  - $\mathcal{L}$ is a $3 \times 3 \times 3$ lattice, i.e., it consists of the points (n_1, n_2, n_3) of a Cartesian grid with

$n_i \in \{0, 1, 2\}$.

- t involves the central site $s_0 = (1,1,1)$, and the central-north site at $s_N = (1,1,2)$, such that t contains the relative vector of t is $\mathbf{v}_N = s_N - s_0 = (0,0,1)$.
- Then, tiling t to a new site, say $s' = (2,2,2)$, would involve the site $s' + \mathbf{v} = (2,2,3)$, but $s' \notin \mathcal{L}$.
- Therefore the new term is not included in $\mathcal{T}_{\text{NNN+}}$.

- If t' is valid within $\mathcal{L}$, then add it to $\mathcal{T}_{\text{NNN+}}$.

Following this tiling procedure, $\mathcal{T}_{\text{NNN+}}$ now has many more terms and dominates the onsite/nearest-neighbour terms. In dealing with $\mathcal{T}_{\text{NNN+}}$, we do not group terms in advance of costing, since the advantage of this step is to arrange subsets of terms which facilitate the parallelism outlined in Supplementary Note 5 B which is not available for terms in $\mathcal{T}_{\text{NNN+}}$. Moreover, the interactions in this set are large-range by definition, i.e., they extend across at least three sites of $\mathcal{L}$, necessitating either high-cost circuit terms or many fswaps in order to achieve low-cost terms. Since we do not have groupings, here we opt to omit the swap network stage of the compiler since it would have to act on the entire space of $\mathcal{L}$ which is unlikely to outperform the direct cost of the terms, although it is an open question whether a swap routine could improve the depth here. For these reasons, the depth associated with $\mathcal{T}_{\text{NNN+}}$ is expected to dominate the circuit for simulating the Hamiltonian, despite not dominating the total number of terms. We are currently working to improve the compiler to handle NNN+ terms, by incorporating logic which is able to generically understand the non-trivial relationship between the translational symmetry of the physical system and the translational symmetry of the given encoding. This will allow for the grouping and tiling of beyond-nearest-neighbour terms in the same fashion as onsite and nearest-neighbour terms – which is expected to yield significant reduction in circuit depths for the unitary execution of NNN+ terms.

C. Circuit costing

After applying the decomposition routines, each grouping is given a circuit cost according to the terms it contains. The depth cost of an individual grouping is given by the largest circuit depth of an individual term in the group, which may depend on the gate set available in hardware. Assuming access to arbitrary 2-qubit rotations yields a depth of $2\lceil \log_2(w) \rceil - 1$, where w is the weight of the Pauli term (see Supplementary Note 4 A). Summing this cost over all groups gives the total depth. In order to account for the fact that 2-qubit gates are given cost 1, we modify the disjoint qubit decomposition routine to allow any terms acting on exactly the same two qubits to be kept together in the same grouping. This way the coster treats them as though they are performed in parallel.

The fermionic swap network will introduce a number of fswap gates. These can be handled in exactly the same fashion as the time evolution operations coming from the Hamiltonian. See Supplementary Note 4 A for details.

Supplementary Notes 6 – Results

In this section we present several results about the circuit costs of different materials using the methods discussed in this paper. To familiarise the reader with our approach, in Supplementary Note 6 A we walk through a system simple enough that all the components can be understood easily. This system consists of a 2D lattice of unit cells with $N_{\text{orbitals/cell}} = 2$ orbitals per unit cell. We include this discussion as an aid to grasp the algorithmic concepts developed in this work. This simple system allows one to detach the analysis of the circuit complexity from the physics, but it does not answer satisfactorily how to generate a Hamiltonian instance related to a particular material. In Supplementary Note 6 B we describe in full detail the whole construction developed in this work, from a DFT analysis all the way down to the circuit decomposition, for the strongly correlated material strontium vanadate ($SrVO_3$). The results for different materials and assumptions are shown in Supplementary Note 6 C 3.

A. Circuit analysis of a simple example

Here we discuss a simple model of immobile impurity levels coupled to mobile electrons (the bath) in 2D and show the results of applying our circuit compiler to it. The unit cell contains one impurity level and one bath mode, and the bath modes can hop to neighboring sites (see Supplementary Figure 22). We first concentrate on a single unit cell, where we discuss in detail the procedure of mapping a fermionic model into a Pauli Hamiltonian, and the use of the JW string and fswap networks. In Supplementary Note 6 A 3 we discuss tiling this unit cell across the lattice, and the advantages of the hybrid encoding. Each disconnected unit cell is easily diagonalisable on a classical computer.

1. *One unit cell*

A system with a single unit cell consists of two electrons, one being the impurity electron of spin σ (created and annihilated by $d_\sigma^\dagger$ and d_σ, respectively) and the other being the bath electron of spin σ (created and annihilated by $c_\sigma^\dagger$ and c_σ, respectively). This corresponds to the simple Hamiltonian in complex fermion form

$$H_{\text{cell}} = \sum_{\sigma=\uparrow,\downarrow} (\epsilon_\sigma c_\sigma^\dagger c_\sigma + \Delta(c_\sigma^\dagger d_\sigma + d_\sigma^\dagger c_\sigma) + \epsilon_\sigma^d d_\sigma^\dagger d_\sigma) + U d_\uparrow^\dagger d_\downarrow^\dagger d_\downarrow d_\uparrow, \tag{112}$$

where ϵ_σ, ϵ_σ^d, Δ, and U are real parameters. This system can be represented by 4 modes. Relabelling $(d_\uparrow, d_\downarrow, c_\uparrow, c_\downarrow) = (a_0, a_1, a_2, a_3)$ and introducing the Majorana fermion representation $a_j = (\gamma_{2j} + i\gamma_{2j+1})/2$ the Hamiltonian in Eq. (112) (up to an overall constant) becomes

$$H_{\text{cell}} = \sum_{j=0}^{3} i\frac{\epsilon_j}{2}\gamma_{2j}\gamma_{2j+1} + \sum_{j=0,1} i\frac{\Delta}{2}(\gamma_{2j}\gamma_{2j+5} - \gamma_{2j+1}\gamma_{2j+4}) - \frac{U}{4}\gamma_0\gamma_1\gamma_2\gamma_3, \tag{113}$$

with parameters $(\epsilon_0, \epsilon_1, \epsilon_2, \epsilon_3) = (\epsilon_\uparrow^d + \frac{U}{2}, \epsilon_\downarrow^d + \frac{U}{2}, \epsilon_\uparrow, \epsilon_\downarrow)$. The particular connectivity structure in this example maps straightforwardly to the fermionic encoding given by a single JW line. As we will see in more complex situations, this encoding can be hybridized with the compact encoding to generate a more efficient mapping, i.e., one that reduces the operator weight in term of the Pauli operators. In what follows, we will use the JW string depicted in Supplementary Figure 22[7].

In this system we have two type of fundamental interactions: $\gamma_{2m}\gamma_{2l+1}$ or $\gamma_{2m+1}\gamma_{2l}$ (with $m \leq l$). The quartic interaction is a product of these fundamental ones. Interaction terms like $\gamma_{2i}\gamma_{2j}$ or $\gamma_{2i-1}\gamma_{2j+1}$ are not present because the original Hamiltonian is Hermitian and invariant under complex conjugation $\mathcal{K}$ (i.e., $\mathcal{K}H\mathcal{K}^{-1} = H$, with $\mathcal{K}i\mathcal{K}^{-}1 = -i$, where i is the imaginary number). This antiunitary symmetry acts like usual time-reversal symmetry on spinless fermions. We find

$$\mathcal{K}a_j\mathcal{K}^{-1} = a_j \rightarrow \mathcal{K}\gamma_{2j}\mathcal{K}^{-1} = \gamma_{2j}, \quad \mathcal{K}\gamma_{2j+1}\mathcal{K}^{-1} = -\gamma_{2j+1}. \tag{114}$$

Hermitian quadratic operators have the form $i\gamma_a\gamma_b$. For these terms to be invariant under $\mathcal{K}$, they have to contain an odd number of Majoranas with odd index, according to Eq. (114). This shows the role of symmetry in restricting the type of operators present in the Hamiltonian. Interactions are uniquely specified by the structure of the Majorana monomial, and can be mapped directly to the Pauli algebra under the JW encoding. There are three possibilities: $i\gamma_{2m}\gamma_{2m+1}$ is mapped into Z_m, $-i\gamma_{2m}\gamma_{2l+1}$ to $Y_m S_{ml} Y_l$, and $-i\gamma_{2m-1}\gamma_{2l}$ to $-X_m S_{ml} X_l$ (with $m \leq l$). Here, $S_{ij} \equiv \prod_{i<k<j} Z_k$ is a string of Pauli Zs between the mode $i+1$ and the mode $j-1$, For further details see Supplementary Note 3.

---

[7] The reader may note that for the particular connectivity graph of this problem, a better JW ordering would be produced by relabelling $2 \rightarrow 0$ and $0 \rightarrow 2$, as there all the interactions would be between nearest neighbors. We intentionally choose the ordering shown above, as it motivates the introduction of larger weight operators, which are needed in generic situations where the connectivity graph does not consist of a simple line.

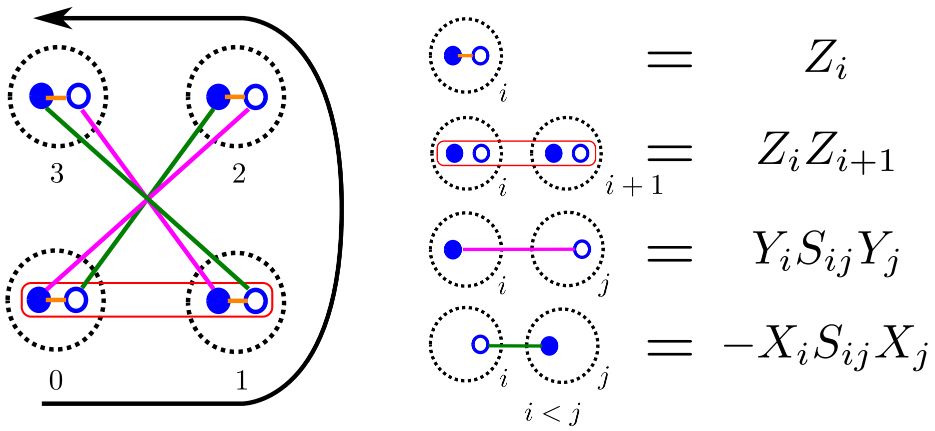

Supplementary Figure 22. (Left) Connectivity graph of a single unit cell. Each mode is represented by dashed circles. Filled (open) blue circles inside represent the even (odd) Majoranas at that site. Quadratic interactions are depicted as lines between Majorana modes. The quartic interaction is represented by the red rectangle containing four modes. The black arrow denotes the JW ordering. (Right) Four different types of interactions in the Hamiltonian and their map into Pauli monomials. S_{ij} is a string of Pauli Z between i and j (see main text for more details).

The Hamiltonian H_{cell} in the Pauli representation is

$$H_{\text{cell}} = \sum_{j=0}^{3} \frac{\epsilon_j}{2} Z_j - \sum_{j=0,1} \frac{\Delta}{2}(Y_j S_{j,j+2} Y_{j+2} + X_j S_{j,j+2} X_{j+2}) + \frac{U}{4} Z_0 Z_1. \tag{115}$$

For quantum algorithms like VQE or TDS, we need to create a unitary of the form $U = e^{i\theta H_{\text{cell}}}$, where θ is some parameter. In a quantum computer, the accessible operations form a fixed subset of gates, from which any possible unitary on the whole system can be approximated. Usually these consists of 1-qubit and 2-qubit gates. Using the Suzuki-Trotter formula, we can approximate the unitary U by a series of simpler unitaries that can be implemented in the quantum computer. This decomposition has to be done in a way that minimizes the depth of the circuit constructed.

We wish to know the circuit depth of the unitary generated by the Pauli string for a given interaction. Our circuit depth will reflect the number of layers of 2-qubit gates required to run a given algorithm for the specified Hamiltonian. If only a single qubit is involved, i.e., the weight is $w = 1$, we only require 1-qubit gates, which we assume access to at no cost. In general, for Pauli strings with weight w, we saw in Supplementary Note 4 A that such a circuit can be performed with *cost*

$$c(w) = 2\lceil \log_2 w \rceil - 1 \tag{116}$$

sublayers of 2-qubit gates. However, we cannot concurrently perform operations which involve the same qubits: that is, we must separate the interactions into lists, each of which contains terms with disjoint support. We therefore separate the terms in Eq. (115) into a number of *layers*, where each layer can consist of several terms that can be implemented simultaneously. The cost of any layer is the depth, d, of the most expensive term in that layer. The overall depth is therefore

$$d = \sum_{l \in \text{layers}} \max_{w} \{c(w)\}_l. \tag{117}$$

We break down the set of interactions in Eq. (115) into layers and show the resulting circuit in Supplementary Figure 23. The overall circuit depth for implementing all the terms of this Hamiltonian once is therefore $d = 13$ rounds of 2-qubit gates.

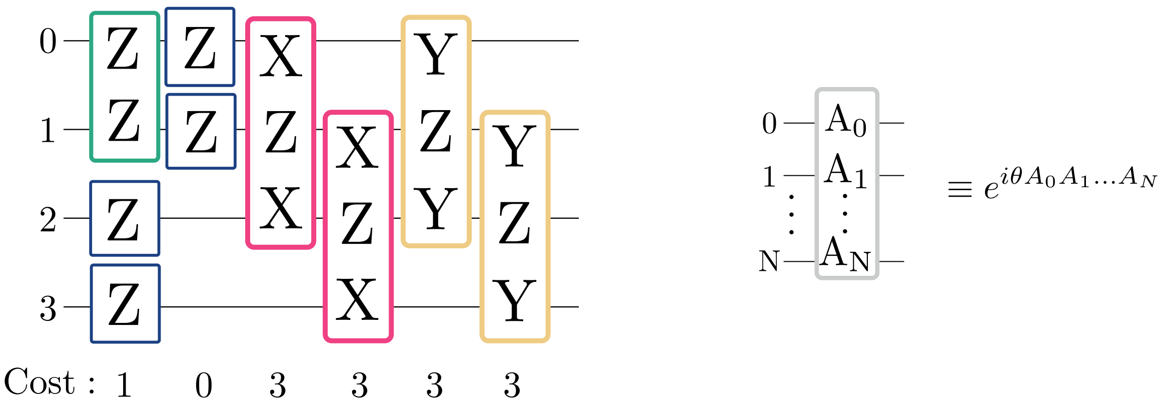

Supplementary Figure 23. (Left) Circuit decomposition to implement all the terms of the Hamiltonian in Eq. (115) once. The cost of each layer of gates is shown below.(Right) Circuit notation used. Each box in the left represents a unitary. The parameters θ used may vary depending on the particular algorithm.

2. *Fermionic swap networks*

Many terms in the circuit represented in Supplementary Figure 23 have cost 3, since their Pauli string crosses a third qubit, whereas monomials involving only adjacent qubits have depth 1. It may therefore be advantageous to rearrange the modes before implementing interactions: *fermionic swap* (fswap) operations can be used to move modes through the graph. Although they also incur a cost (as described in Supplementary Note 5 A), here we assume they have uniform cost $d = 1$. However, if they successfully enable low-weight operations for all other interactions, they can prove beneficial overall.

The initial mode configuration can allow implementation of the terms $\{Z_0Z_1, Z_i\}$, but as above, we must split these into sublayers whose constituents have disjoint support, i.e., we create two layers $\{Z_0Z_1, Z_2, Z_3\}; \{Z_0, Z_1\}$. Thereafter, we run two fswap layers. Denoting the fswap between modes a and b as FSWAP_{ab}, we apply the layers $\{\mathrm{FSWAP}_{01}, \mathrm{FSWAP}_{23}\}; \{\mathrm{FSWAP}_{12}\}$, which maps the original JW ordering $(0, 1, 2, 3)$ into the ordering $(1, 3, 0, 2)$. In this new ordering the operators $\gamma_0\gamma_5$, $\gamma_2\gamma_7$, $\gamma_1\gamma_4$, and $\gamma_3\gamma_6$ all represent interactions between Majoranas in nearest neighbor modes and as such, they do not have strings of Z operators attached. This allows (after splitting into disjoint-support sublayers) the interactions

$$\{Y_0Y_2, Y_1Y_3\}; \{X_0X_2, X_1X_3\}.$$

We also must reverse the fswaps in order to recover the starting mode configuration, so after the final interaction layer, there are two further fswap layers. This step is useful if one wishes to apply the same exact circuit many times, but can be relaxed if we may start from a scrambled configuration in the next step. However, care should be taken when applying the compiler to ensure that translational invariance is preserved, and if modes are scrambled at the beginning of a given layer then a new compilation must be performed for that layer. Therefore, generally the simplest strategy is to return modes to their original configuration at the end of a given Trotter or VQE layer. The swapped circuit and its associated costs are depicted in Supplementary Figure 24. We include the cost of the fswap layers since it contributes to the overall circuit depth. The total depth using this method is then $d = 7$, verifying that the generation of an fswap network is justified here, since it leads to a decrease in circuit depth compared with the prior method which gave $d = 13$ in Supplementary Figure 23.

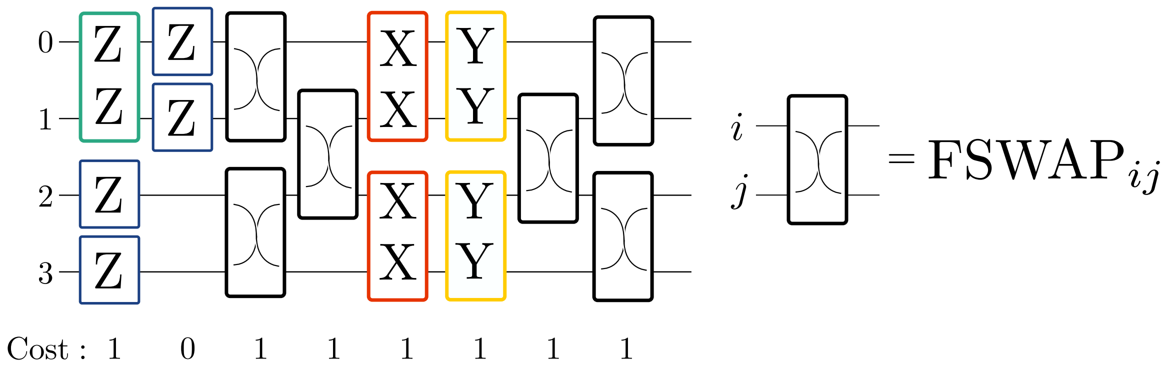

Supplementary Figure 24. (Left) Circuit implementing the unitaries generated by all the terms in the Hamiltonian. In this approach we use fermionic swap networks to decompose high weight operators into two qubit gates. If the cost of doing fermionic swap gates is small, this strategy can be advantageous. Further gains can be achieved by merging two-qubit gates – e.g., the gates generated by XX and YY at the centre of the circuit – which is preferred in a cost model where **any** 2-qubit gate has the same cost $d = 1$. (Right) Diagram for the fermionic- swap gate.

3. *Many unit cells*

We are now in a position to discuss the generalized model of impurity levels coupled to mobile electrons. Consider the Hamiltonian

$$H = \sum_{k \in \text{cells}} H_{\text{cell}}^{(k)} + t \sum_{\langle i,j \rangle} (c_{i,\sigma}^\dagger c_{j,\sigma} + c_{j,\sigma}^\dagger c_{i,\sigma}), \tag{118}$$

where $H_{\text{cell}}^{(k)}$ is the Hamiltonian of the kth cell, and the second term represents the intercell coupling, produced by the hopping of bath modes between nearest neighbor cells in 2D. Following the procedure outlined above has some drawbacks in this larger system. Although the fermions hop locally in the 2D system, after including a JW ordering that maps the system into a line, some local interactions become very non-local (see Supplementary Figure 25). This ultimately increases the circuit depth by a factor that depends on the whole size of the system.

To keep the operator weight of Pauli strings independent of the system size, we exploit the hybrid encoding introduced in Supplementary Note 3. In this encoding, we add extra ancilla qubits that allow to represent the fermion algebra with low-weight Pauli operators. The price to pay is that more qubits are needed. In materials the interactions (understood generically as electron-electron interactions or hopping) between the modes in the unit cell are expected to be more dense than the intercell interactions, meaning that inside the cell the fswap protocol could be very beneficial to bring modes together (as discussed in Supplementary Note 5 A). The hybrid encoding can minimise the use of the extra qubits by using them to implement the interactions between different cells, which are expected to be sparse.

B. Full-stack analysis: Strontium vanadate

In this section we present a full-stack analysis of the transition metal perovskite oxide $SrVO_3$. Its unit cell is shown in Supplementary Figure 26(a). Materials in the perovskite oxide family with chemical formula ABO_3 form basic components of the Earth's mantle, are central to many technological applications, and pioneer current research efforts to design bespoke materials with multifunctional properties. They can exhibit a wide range of physical properties, spanning insulating, semiconducting, and metallic characteristics as well as a superconducting, correlated, multiferroic, and ferroelectric phases, and highly controllable transitions between them. For example, synthetic perovskite oxides are used in batteries [74], qubits [75], high-temperature superconductors [76], solar cells [77], semiconductors [78], spin switches [79], multiferroics [80] and ferroelectrics [81]. In

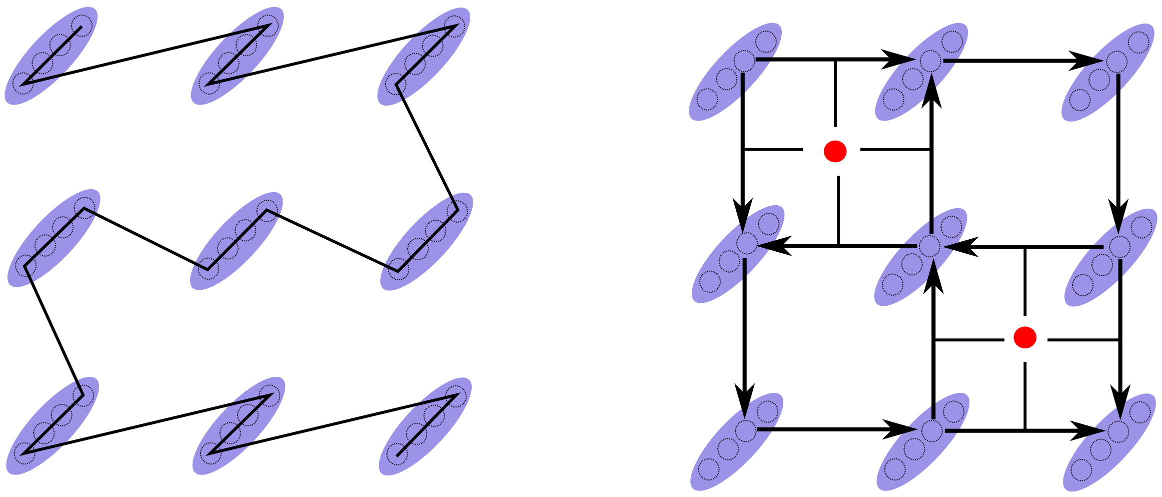

Supplementary Figure 25. (Left) JW ordering across a 2D lattice. Neighboring unit cells (ellipses) can end very far away in the JW chain, leading to a large operator weight for physical processes that happen locally. (Right) Using extra ancilla qubits (red circles), the hybrid encoding can generate low-weight Pauli operators for the intracell processes. For the intercell processes, JW ordering and fswap networks can implement all the required terms.

particular, $SrVO_3$ has been used as components in both the anode [82] and cathode [83] of Li-ion batteries. Their widespread usage is a result of the remarkable stability of the ABO_3 chemical structure [84], and allows for highly tunable functional properties as a result of being able to control and couple the many electronic degrees of freedom, such as orbital, charge, and lattice in a large phase space of chemical species. We start by presenting the DFT results and their subsequent mapping to MLWFs, followed by the computation of the single-body and two-body matrix elements that parametrise the complex fermionic Hamiltonian of a periodic system. We conclude with a discussion on the calculation of the quantum complexity for this Hamiltonian by calculating its circuit depth.

We first describe the results as obtained from DFT and the subsequent Wannierisation procedure. All DFT calculations were performed using the plane-wave code Quantum Espresso [29, 30], version 6.8, together with the GGA-PBE exchange correlation functional [85]. Atomic cores were treated using the ONCVPSP pseudopotential library [33] with valence configuration Sr($4s4p4d5s5s5p$), V($3s3p3d4s$), and O($2s2p$). The plane-wave basis representation is used for the wavefunctions, with a cutoff of $E_{\text{cut}} = 400$ eV (see Eq. (83)). We use a $4 \times 4 \times 4$ Γ-centered k-point mesh in the Brillouin zone for k-point sampling. Structural degrees of freedom are relaxed until all forces are smaller than 1 mRyd/a.u. (see Eq. (86)). Subsequently, the generation of MLWFs is performed with Wannier90 [31].

Supplementary Figure 26(b) presents the electronic bandstructure of $SrVO_3$ along the high symmetry Brillouin zone path. The Fermi level is indicated by the horizontal red line, which intersects a triply degenerate band with bandwidth ~ 2 eV, and is thus representative of a metallic system. We also note the set of 9 non-degenerate bands in the range $[-8, -2]$ eV (where E_f is zeroed).

To identify the orbital character of the electronic structure in Supplementary Figure 26 we perform a fatbands projection analysis according to Eq. (89) using d-orbital projections for the vanadium ions and p-orbital projections for the oxygen ions. These results are presented in Supplementary Figure 27, where we see that the states of the triply degenerate band at the Fermi level belong to the d-states of vanadium. Furthermore, we see from Supplementary Figure 27(a) and (b) that the full d-manifold of states splits into the e_g and t_{2g} crystal field subgroups of the overall full d (rotation) group, where the t_{2g} states are responsible for the conduction electrons. Moreover, the states below the Fermi level in the range $[-8, -2]$ eV are primarily O-p type. Knowing the orbital character of the electronic states is a crucial step in determining the necessary initial projectors used for generating MLWFs, which affects the numerical stability of the Wannierisation procedure. Additionally, it is an essential component in determining the physical interpretation of these electronic states, required

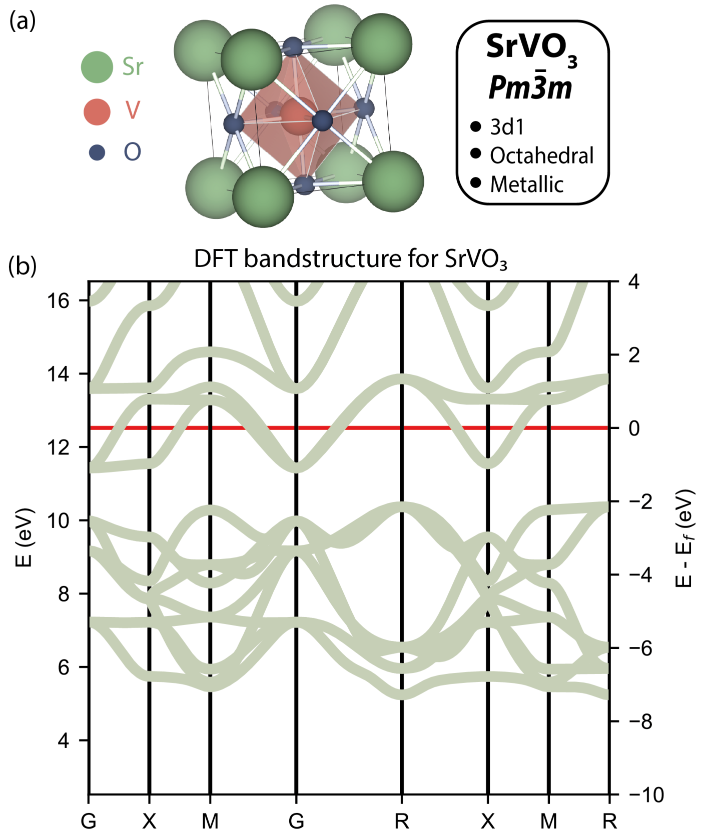

Supplementary Figure 26. (a) The structural unit cell of $SrVO_3$ with spacegroup $Pm\bar{3}m$, 3d1 nominal electronic valence configuration, and octahedral coordination environment. In this configuration, the compound is a metal. (b) The ground state electronic bandstructure as predicted using DFT along the high symmetry path in the Brillouin zone. The red line indicates the position of the Fermi level E_f.

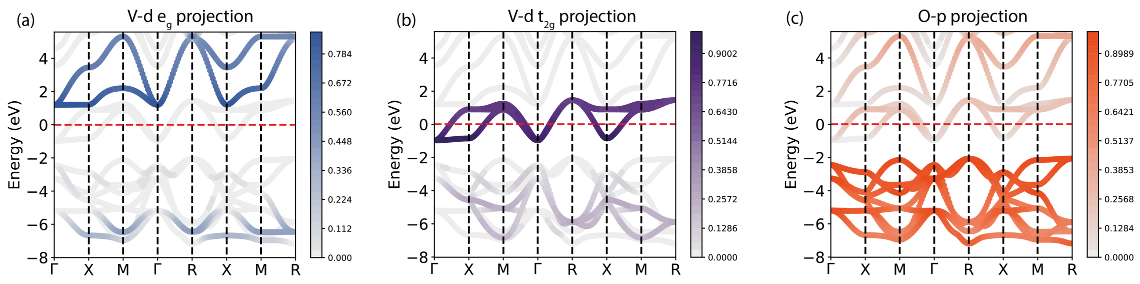

Supplementary Figure 27. Fatbands projections on the DFT electronic band structure for $SrVO_3$ using projectors corresponding to (a) V-d e_g orbitals, (b) V-d t_{2g} orbitals, and (c) O-p orbitals.

for comparing against experimental results.

Knowing the orbital character of the electronic bands near the Fermi level allows us to reliably choose regions of the bandstructure over which an active space can be chosen, and which can be used to parametrise fermionic Hamiltonians. The upper panel of Supplementary Figure 28 presents two possible choices of active space for $SrVO_3$. In Supplementary Figure 28(a) we illustrate the active space containing the 3 V-t_{2g} orbitals, while in Supplementary Figure 28(b) we show the active space that contains the 3 V-t_{2g} and 9 O-p orbitals. Accounting for spin degeneracy, the t_{2g} active space contains 6 modes, while the t_{2g}+p active space contains 24 modes. Choosing a larger window over

which the Wannier functions can be generated allows for their maximal localisation, but at the price of including additional modes, while wannierising over smaller active spaces results in a larger total spread of the resultant Wannier functions. In Supplementary Figure 28(c)-(d) we show the corresponding MLWFs, highlighting a larger total spread for the t_{2g} active space compared to the t_{2g}+p active space.

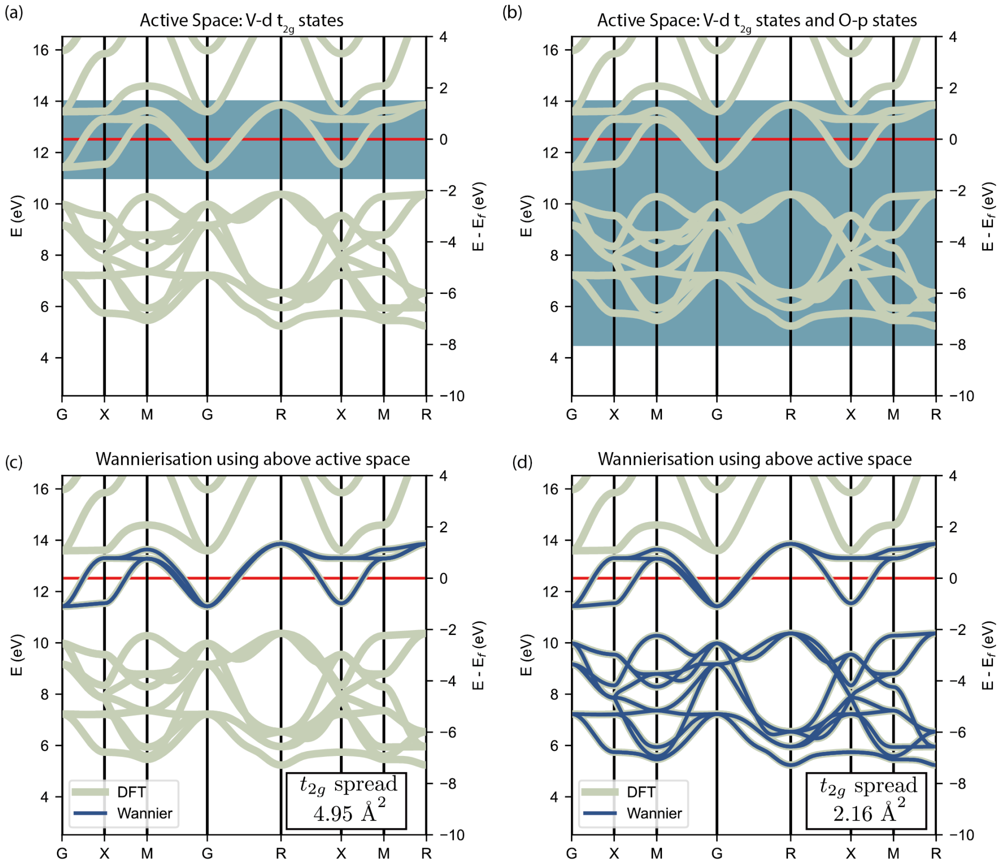

Supplementary Figure 28. Two choices of active space for the Wannierisation protocol consisting of (a) 3 V-d t_{2g} states and (b) 3 V-d t_{2g} states, as well as an additional 9 O-p states. The bands obtained via the Wannierization procedure for the two choices of active space are shown in panels (c) and (d). In the bottom-right corners we highlight the spread – as defined in [15] – of the MLWFs corresponding to the t_{2g} levels in the two cases, which is (c) 4.95 Åand (d) 2.16 Å, respectively.

Furthermore, Supplementary Figure 29(a)-(b) illustrates the individual spreads of the generated Wannier functions. By wannierising over the large energy window, we see that the smallest spreads belong to the V-t_{2g} orbitals, and increases for the O-p states. Supplementary Figure 29(c)-(d) presents the corresponding t_{2g} Wannier functions on a real space grid, highlighting the clear differences in their locality depending on the size of the active space considered. Using the t_{2g} active space only results in Wannier functions which extend into neighbour and nearest-neighbour cells, thus resulting in significant overlap with the Wannier functions in those cells. Whereas the larger active space results in localised Wannier functions that do not extend into neighbouring cells.

1. Computation of hopping matrix and Coulomb tensor coefficients

Once selected the active space and built the corresponding MLWFs, we can proceed with the calculation of the hopping matrix and Coulomb tensor coefficients as defined in Eq. (34). As explained

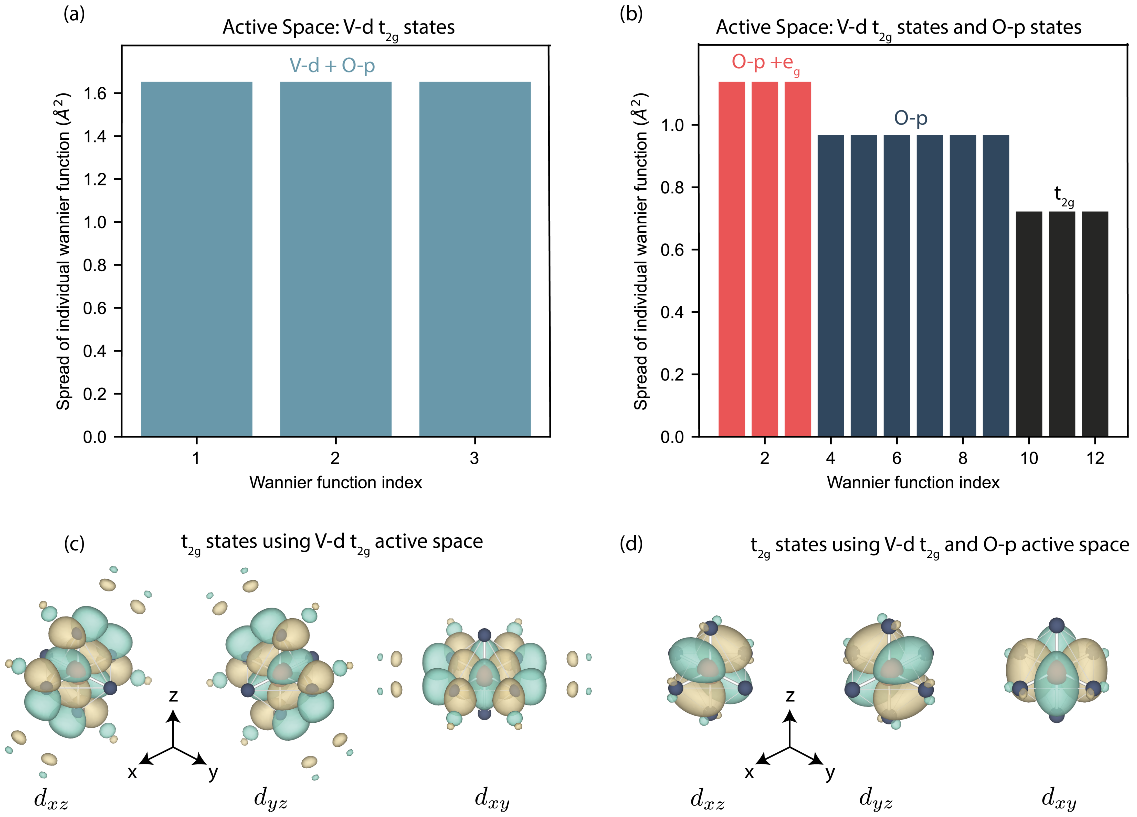

Supplementary Figure 29. The spread of each Wannier function for the subspaces consisting of (a) 3 V-d t_{2g} states and (b) 3 V-d t_{2g} states with an additional 9 O-p states, as illustrated in Supplementary Figure 28. The isosurface plot of the corresponding MLWFs for the (c) small and (d) large active spaces. Here, the isosurface value has been set to 0.325.

in Supplementary Note 2 F, in the usual DFT procedure the ionic potential $\tilde{U}(\mathbf{r})$ for the electrons in the active space is replaced by the effective Kohn-Sham potential $\tilde{U}_{\text{eff}}(\mathbf{r})$. The latter contains the entangled contributions arising from the original ionic potential, the screening effects of core electrons, and part of the electron-electron interaction. Therefore, it is convenient to rewrite the hopping matrix and Coulomb tensor for the electrons in the active space as

$$T(\mathbf{R})_{mn} = \int d\mathbf{r} \mathcal{W}^{\mathbf{R}}_{m,\sigma}(\mathbf{r}) \left[-\frac{\hbar^2 \nabla^2}{2m} + \tilde{U}_{\text{eff}}(\mathbf{r}) \right] \mathcal{W}^{\mathbf{0}}_{n,\sigma}(\mathbf{r}), \tag{119a}$$

$$\tilde{V}^{(\mathbf{0},\mathbf{R}_2,\mathbf{R}_3,\mathbf{R}_4)}_{slmn} = \frac{1}{2} \int d\mathbf{r} \int d\mathbf{r}' \mathcal{W}^{\mathbf{0}}_{s,\sigma}(\mathbf{r}) \mathcal{W}^{\mathbf{R}_2}_{l,\sigma'}(\mathbf{r}') W(\mathbf{r},\mathbf{r}') \mathcal{W}^{\mathbf{R}_3}_{m,\sigma'}(\mathbf{r}') \mathcal{W}^{\mathbf{R}_4}_{n,\sigma}(\mathbf{r}), \tag{119b}$$

where $W(\mathbf{r},\mathbf{r}')$ is the screened Coulomb potential between the electrons in the active space arising from the truncation procedure described in Supplementary Note 2 F 2 and which, in general, include contributions to the electron-electron interaction not captured by the DFT procedure. Many sophisticated approaches have been designed to mitigate the double-counting emerging from this decomposition and to obtain accurate values of the screened Coulomb potential [8, 36, 40, 86, 87] but they are beyond the scope of this work. Instead, one simpler approach is to consider a Thomas-Fermi screened interaction potential of the form $W(\mathbf{r},\mathbf{r}') = q_e/(4\pi\epsilon_0)e^{-\mu_{\text{TF}}|\mathbf{r}-\mathbf{r}'|}/|\mathbf{r}-\mathbf{r}'|$, with μ_{TF} a material dependent inverse screening length [88]. For the sake of simplicity, in what follows we will focus on the unscreened case only, corresponding to setting $\mu_{\text{TF}} = 0$ (i.e., we assume $W(\mathbf{r},\mathbf{r}') = V(|\mathbf{r}-\mathbf{r}'|)$). This will result in more, stronger, and longer-range interactions and, therefore, the results we will show below represent an upper bound for the quantum circuit complexities for the simulation of real materials. The effects of screening will be addressed in future work.

All the steps to calculate the hopping matrix and Coulomb tensor coefficients are described in detail in Appendix D. In what follows, we will give a brief description of the most important steps and analyse the outputs for the case of $SrVO_3$ with 3 V-t_{2g} states in its active space introduced in the previous section. Importantly, we will work within the following assumptions:

1. We will consider non-magnetic materials only, so that the two spin sectors are degenerate;

2. Hopping matrix and Coulomb tensor coefficients involving unit cells which are nearest neighbors of order larger than n_0 and n_{int}, respectively, are negligible;

3. Hopping matrix and Coulomb tensor coefficients whose absolute value is smaller than a pre-determined threshold are negligible.

The second assumptions takes advantage of the real-space localisation of the MLWFs and implies that the hopping matrix and the Coulomb tensor coefficients are calculated on motifs of order n_0 and n_{int}, respectively. Recalling the definitions of Supplementary Note 2 C 1, they are denoted by $\mathcal{N}_O^{n_0}$ and $\mathcal{N}_O^{n_{\text{int}}}$. In Supplementary Figure 30 we show the motifs of order 1,2, and 3 for $SrVO_3$. Each site of the motif corresponds to a unit cell of the material. We denote with $N_{\text{cells/motif}}$ the number of unit cells per motif and with and $N_{\text{orbitals/cell}}$ the number of orbitals per cell (i.e., the number of bands contained in the chosen active space). Note that $N_{\text{cells/motif}} = \max(\dim(\mathcal{N}_{\mathbf{0}}^{n_0}), \dim(\mathcal{N}_{\mathbf{0}}^{n_{\text{int}}}))$. The total number of complex fermion modes per motif is $M = 2N_{\text{cells/motif}}N_{\text{orbitals/cell}}$.

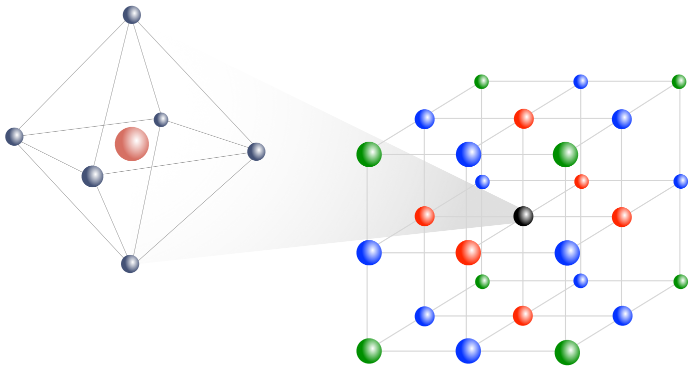

Supplementary Figure 30. Unit cell and motif for $SrVO_3$. In the unit cell, on the left, only the atoms whose atomic orbitals contribute significantly to the bands in the selected active space are shown, namely the central vanadium atom (big orange sphere) surrounded by six oxygen atoms (small blue spheres) (see Supplementary Figure 26). The full $SrVO_3$ lattice, a portion of which is shown on the right hand side, can be obtained by translating the unit cell by multiple integers of the lattice vectors. The motif of order $n = 1$ is formed by the central cell (black) and the 6 nearest neighbors (red), the motif of order $n = 2$ includes also the 12 next-nearest neighbors (blue), and the motif of order $n = 3$ is obtained by adding the 8 next-to-next-nearest neighbours (green).

a. Hopping matrix. The hopping matrix $T(\mathbf{R})$ can be directly obtained from the output of the Wannierisation procedure described in the previous section as performed by Wannier90. For instance, the hopping of electrons from the central unit cell at $\mathbf{R} = \mathbf{0}$ to the cells at $\mathbf{R}_A = \mathbf{R}_1$ and $\mathbf{R}_B = \mathbf{R}_1 + \mathbf{R}_2$ (with, in general, $\mathbf{R} = n_1\mathbf{R}_1 + n_2\mathbf{R}_2 + n_3\mathbf{R}_3$; see Eq. (11)) is described by the two matrices

$$T_{(100)}[\text{eV}] = \begin{pmatrix} -0.26 & 0 & 0 \\ 0 & -0.26 & 0 \\ 0 & 0 & -0.26 \end{pmatrix}, \quad T_{(110)}[\text{eV}] = \begin{pmatrix} 0.006 & 0.009 & 0 \\ 0.009 & 0.006 & 0 \\ 0 & 0 & -0.082 \end{pmatrix}, \tag{120}$$

respectively. Here, we introduced the notation $T_{(n_1n_2n_3)} := T(\mathbf{R})$. Noting that $|\mathbf{R}_A| < |\mathbf{R}_B|$, the equation above also shows that, in general, the value of the hopping matrix coefficients decrease as a function of the distance between the cells and therefore a nearest neighbor approximation is well justified.

n_0	All	Non-zero	$\lvert T(\mathbf{R})_{mn}\rvert > t_0$
1	63	21	15
2	171	81	33
5	513	296	93

Supplementary Table 4. Number of total (second column), non-zero (third column), and filtered hopping matrix coefficients (fourth column) for $SrVO_3$ for different values of the motif order, $n_0 = 1, 2, 5$, respectively.

In the next step we determine the order n_0 of the motif to be used in the truncation of the hopping matrix according to the second assumption discussed above. We do that by setting, for a given n_0, $T(\mathbf{R})_{mn} = 0, \forall \mathbf{R} \notin \mathcal{N}_{\mathbf{0}}^{n_0}$ and comparing the band structure obtained from the truncated hopping matrix with the original one. Here, we recall that $\mathcal{N}_{\mathbf{0}}^{n_0}$ denotes the set of lattice vectors corresponding to those unit cells which are nearest neighbors of order $\leq n_0$ with respect to the central one. The band structure obtained from truncated hopping matrix is given by the eigenvalues of the matrices

$$h(\mathbf{k})_{mn} = \sum_{\mathbf{R}\in\mathcal{N}_{\mathbf{0}}^{n_0}} e^{i\mathbf{k}\cdot\mathbf{R}} T(\mathbf{R})_{mn} \tag{121}$$

for values of $\mathbf{k}$ along the high symmetry path in the Brillouin zone. For $SrVO_3$, the bands obtained from this approximation are shown in blue in Supplementary Figure 31 for (a) $n_0 = 2$ and (b) $n_0 = 5$. To obtain a consistent approximation, we then introduce the following filtered hopping matrix

$$\overline{T}(\mathbf{R})_{mn} = \begin{cases} T(\mathbf{R})_{mn} & \text{if } \lvert T(\mathbf{R})_{mn}\rvert \geq t_0 \\ 0 & \text{otherwise} \end{cases}, \tag{122}$$

where we set to zero all the coefficients $T(\mathbf{R})_{mn}$ smaller than a threshold $t_0 = \tau_0 \times \max\lvert T(\mathbf{R})_{mn}\rvert$, $\forall \mathbf{R} \notin \mathcal{N}_{\mathbf{0}}^{n_0}$. The latter is obtained from the largest of the absolute values of the hopping coefficients involving sites with nearest neighbour order $> n_0$ with respect to the central cell. For $SrVO_3$ this further step results in the red bands in Supplementary Figure 31 which represent a good approximation of the truncated bands for both values of n_0. In this case, as shown in Supplementary Table 4, the two approximations combined allow us to reduce the number of non-zero hopping matrix coefficients $T(\mathbf{R})_{mn}$ from 81 to 33 for $n_0 = 2$ and from 296 to 93 for $n_0 = 5$.

In order to determine n_0 in systematic way, we look for the minimum value of n_0 such that the distance between the exact bands $\varepsilon_i(\mathbf{k})$ and the ones obtained from the filtered truncated hopping matrix $\overline{T}(\mathbf{R})_{mn}$, $\overline{\varepsilon}^{(n_0)}(\mathbf{k})$, is smaller than a pre-determined tolerance. In our case, we choose to measure the distance between the bands according to [89]

$$\mathcal{D}(n_0) = \max_{i,\mathbf{k}} \left\lvert \varepsilon_i(\mathbf{k}) - \overline{\varepsilon}_i^{(n_0)}(\mathbf{k}) \right\rvert, \tag{123}$$

where i is the band index and $\mathbf{k}$ is sampled from a regular grid on the Brillouin zone. In what follows, for each material we will use the smallest value of n_0 such that $\mathcal{D}(n_0) \leq 0.5$ eV.

b. Coulomb tensor. To obtain the Coulomb tensor coefficients $\tilde{V}_{slmn}^{(\mathbf{0},\mathbf{R}_2,\mathbf{R}_3,\mathbf{R}_4)}$ one has to compute numerically the 6−dimensional integral in Eq. (119b) using the Wannier functions generated by Wannier90 for each value of the orbital and site indices. For a standard material this would result in an extremely large number of integrals and, consequently, long classical computational time. However, thanks to the localisation of the MLWFs, only a few of these integrals are important in determining the properties of a material. Therefore, as stated in the second working assumption above, we only consider coefficients involving sites which are reciprocally nearest neighbors of order $\leq n_{\text{int}}$ and set to zero all the others. Using the notation introduced above, this means that only the coefficients $\tilde{V}_{slmn}^{(\mathbf{R}_1,\mathbf{R}_2,\mathbf{R}_3,\mathbf{R}_4)}$ with $\mathbf{R}_1, \mathbf{R}_2, \mathbf{R}_3, \mathbf{R}_4 \in \mathcal{N}_{\{\mathbf{0},\mathbf{R}_1,\mathbf{R}_2,\mathbf{R}_3,\mathbf{R}_4\}}^{n_{\text{int}}}$ are non-zero. As described in details in Appendix D 2 b, the symmetry properties of the Coulomb tensor given in Eq. (41) make it possible to identify a minimal set of unique site configurations from which all of the other can be reconstructed at a later stage. For instance, since $\tilde{V}_{slmn}^{(\mathbf{0},\mathbf{0},\mathbf{R},\mathbf{0})} = \tilde{V}_{lsnm}^{(\mathbf{0},\mathbf{0},\mathbf{0},\mathbf{R})}$ it is sufficient to compute

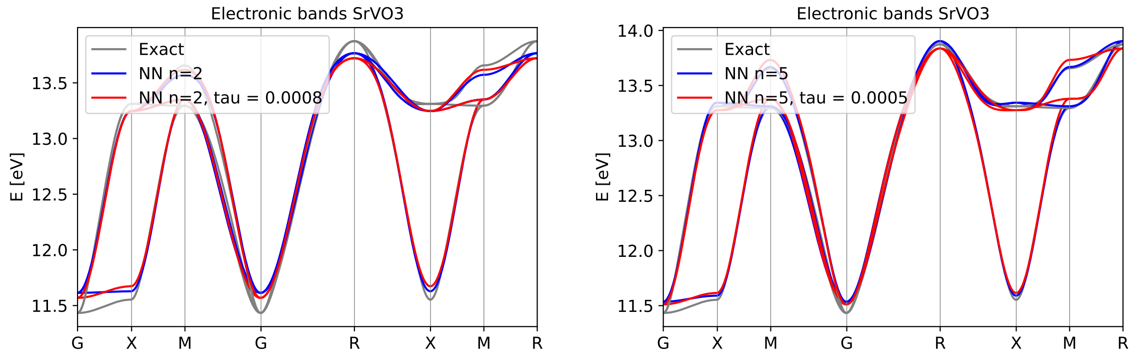

Supplementary Figure 31. Comparison between the exact electronic band structure of $SrVO_3$, $\varepsilon_i(\mathbf{k})$ (grey lines), the one obtained by truncating the hopping matrix $T(\mathbf{R})_{mn}$ to a motif of order n_0, $\varepsilon_i^{(n_0)}(\mathbf{k})$ (blue lines), and the one obtained from the filtered hopping matrix $\tilde{T}(\mathbf{R})_{mn}$, $\overline{\varepsilon}_i^{(n_0)}(\mathbf{k})$ (red lines), for (left panel) $n_0 = 2$ and (right panel) $n_0 = 5$. Here, τ_0 is determined in a such a way that t_0 coincides with the largest absolute value of $T(\mathbf{R})_{mn}, \forall \mathbf{R} \notin \mathcal{N}_{\mathbf{0}}^{n_0}$.

only coefficients with site structure $(\mathbf{0}, \mathbf{0}, \mathbf{0}, \mathbf{R})$. For each unique site configuration, exploiting again Eq. (41), we then determine all the non-equivalent orbital configurations required to obtain the full Coulomb tensor. As an example, for a coefficient with site structure $(\mathbf{0}, \mathbf{0}, \mathbf{R}, \mathbf{R})$ the orbital configurations (s, l, m, n) and (l, s, n, m) are equivalent and, therefore, $\tilde{V}_{slmn}^{(\mathbf{0},\mathbf{0},\mathbf{R},\mathbf{R})} = \tilde{V}_{lsnm}^{(\mathbf{0},\mathbf{0},\mathbf{R},\mathbf{R})}$. Hence, for a given site configuration, we need to compute the Coulomb tensor coefficients only for a restricted set of orbital configurations. This procedure, which is based on fundamental symmetry properties of the Coulomb tensor, allows us to significantly reduce the number of integrals to be computed. For $SrVO_3$, the latter goes from 5913 to 831 for $n_{\text{int}} = 1$ and from 101169 to 3180 for $n_{\text{int}} = 2$. See the "Unique" column in Supplementary Table 5.

As stated above, we also assume that the physical properties of the system are determined by those Coulomb tensor coefficients whose absolute value is larger than a given threshold. We fix the latter as $t_{\text{int}} = \tau_{\text{int}} \times \max|\tilde{V}_{slmn}^{(\mathbf{0},\mathbf{R}_2,\mathbf{R}_3,\mathbf{R}_4)}|$, with $\mathbf{R}_2, \mathbf{R}_3, \mathbf{R}_4 \in \mathcal{N}^{n_{\text{int}}}_{\{\mathbf{0},\mathbf{R}_2,\mathbf{R}_3,\mathbf{R}_4\}}$ and $s, l, m, n \in \{1, ..., M\}$ and define the filtered Coulomb tensor as

$$\overline{V}_{slmn}^{(\mathbf{0},\mathbf{R}_2,\mathbf{R}_3,\mathbf{R}_4)} = \begin{cases} \tilde{V}_{slmn}^{(\mathbf{0},\mathbf{R}_2,\mathbf{R}_3,\mathbf{R}_4)} & \text{if } |\tilde{V}_{slmn}^{(\mathbf{0},\mathbf{R}_2,\mathbf{R}_3,\mathbf{R}_4)}| \geq t_{\text{int}} \\ 0 & \text{otherwise} \end{cases}. \tag{124}$$

This approximation can be conveniently combined with the Cauchy-Schwarz inequality introduced in Supplementary Note 2 A 2 to further reduce the number of integrals which one needs to compute. Indeed, Eq. (8) implies that

$$|\overline{V}_{slmn}^{(\mathbf{R}_1,\mathbf{R}_2,\mathbf{R}_3,\mathbf{R}_4)}|^2 \leq \overline{V}_{ssnn}^{(\mathbf{R}_1,\mathbf{R}_1,\mathbf{R}_4,\mathbf{R}_4)} \overline{V}_{mmll}^{(\mathbf{R}_3,\mathbf{R}_3,\mathbf{R}_2,\mathbf{R}_2)}. \tag{125}$$

Hence, thanks to the discrete translational invariance, if all the coefficients with site structure $(\mathbf{0}, \mathbf{0}, \mathbf{R}, \mathbf{R})$ (with $\mathbf{R} \in \mathcal{N}_{\mathbf{0}}^{n_0}$) are known one can a priori exclude from the computation all those coefficients with site structure $(\mathbf{R}_1, \mathbf{R}_2, \mathbf{R}_3, \mathbf{R}_4)$ and orbital configuration (s, l, m, n) such that

$$\overline{V}_{ssnn}^{(\mathbf{R}_1,\mathbf{R}_1,\mathbf{R}_4,\mathbf{R}_4)} \overline{V}_{mmll}^{(\mathbf{R}_3,\mathbf{R}_3,\mathbf{R}_2,\mathbf{R}_2)} < t_{\text{int}},$$

since Eq. (125) guarantees that they are going to be smaller than the threshold. The advantages deriving by using this procedure are shown in Supplementary Figure 32 for $SrVO_3$ with $\tau_{\text{int}} = 0.01$, $n_{\text{int}} = 1$ and $n_{\text{int}} = 2$. In this case, the Cauchy-Schwarz inequality allows us to further reduce the number of integrals to be computed from a total of 831 to 444 (60 of which will turn to have absolute value greater than the threshold t_{int} after evaluation) for $n_{\text{int}} = 1$ and from 39387 to 3180 (168 of which have absolute value greater than the threshold t_{int}) for $n_{\text{int}} = 2$. See the "Unique+Cauchy-Schwarz" and the last columns in Supplementary Table 5.

$n_{\rm int}$	All	Unique	Unique+Cauchy-Schwarz	Unique $\lvert\tilde{V}^{(\mathbf{R}_1,\mathbf{R}_2,\mathbf{R}_3,\mathbf{R}_4)}_{slmn}\rvert > t_{\rm int}$
1	5913	831	444	60
2	101169	39387	3180	168

Supplementary Table 5. Number of the Coulomb tensor coefficients to be computed for $SrVO_3$ using the various procedures described in the main text for $n_{\rm int} = 1$ and $n_{\rm int} = 2$: total number of coefficients (second column), number of unique coefficients (third column), number of unique coefficients after employing the Cauchy-Schwarz inequality (fourth column), and number of unique coefficients above the threshold (fifth column).

Finally, to obtain a consistent approximation for the Coulomb tensor, we perform an additional filtering step: We focus on the coefficients of the tensor with $n_{\rm int} = 2$ not included in the tensor with $n_{\rm int} = 1$ (i.e., coefficients $\overline{V}^{(\mathbf{R}_1,\mathbf{R}_2,\mathbf{R}_3,\mathbf{R}_4)}_{slmn}$ such that at least one of $\mathbf{R}_1, \mathbf{R}_2, \mathbf{R}_3, \mathbf{R}_4$ is not in $\mathcal{N}^{n_{\rm int}}_{\{\mathbf{0},\mathbf{R}_1,\mathbf{R}_2,\mathbf{R}_3,\mathbf{R}_4\}}$) and we look for the one with the largest absolute value; then, we use the latter as a new threshold $t'_{\rm int}$ to filter once more the Coulomb tensor with $n_{\rm int} = 1$ as we did in Eq. (124).

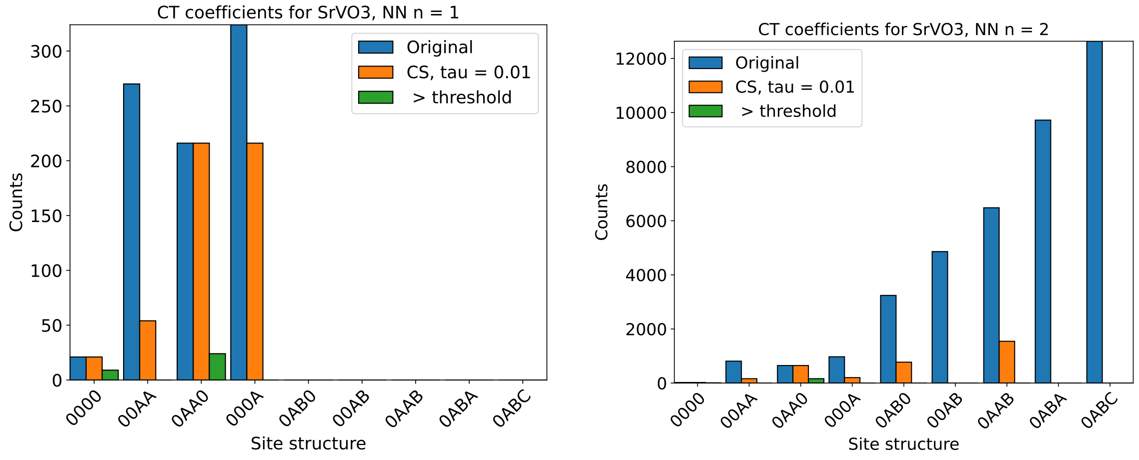

Supplementary Figure 32. Number of unique Coulomb tensor coefficients with site structure $ABCD$ (i.e., $\tilde{V}^{(\mathbf{R}_A,\mathbf{R}_B,\mathbf{R}_C,\mathbf{R}_D)}_{slmn}$ with s, l, m, n being unique orbital configurations of the site structure $(\mathbf{R}_A, \mathbf{R}_B, \mathbf{R}_C, \mathbf{R}_D)$) to be computed without (blue) and with (orange) the Cauchy-Schwarz inequality for (left panel) $n_{\rm int} = 1$ and (right panel) $n_{\rm int} = 2$. In both panels, the green bars denote the number of coefficients per site structure whose actual absolute value is greater than the threshold, with $\tau_{\rm int} = 0.01$.

c. Single-index Hamiltonian From the hopping matrix and the Coulomb tensor evaluated as described above it is straightforward to construct the motif Hamiltonian in the Wannier basis (see Eq. (29)). To proceed, it is convenient to make the (trivial) spin structure of the coefficients explicit and to build a completely anti-symmetric Hamiltonian. This can be done by introducing the following spinful Hopping matrix and Coulomb tensor coefficients,

$$\overline{T}_{(\mathbf{R}_1,m,\sigma_1),(\mathbf{R}_2,n,\sigma_2)} = \begin{cases} \overline{T}(\mathbf{R}_1 - \mathbf{R}_2)_{mn} & \text{if } \sigma_1 = \sigma_2 \\ 0 & \text{otherwise} \end{cases} \tag{126}$$

and

$$\overline{V}_{(\mathbf{R}_1,s,\sigma_1),(\mathbf{R}_2,l,\sigma_2),(\mathbf{R}_3,m,\sigma_3),(\mathbf{R}_4,n,\sigma_4)} = \begin{cases} \overline{V}^{s,(\mathbf{R}_1,\mathbf{R}_2,\mathbf{R}_3,\mathbf{R}_4)}_{slmn} & \text{if } \sigma_1 = \sigma_2 = \sigma_3 = \sigma_4 \\ \frac{1}{2}\overline{V}^{(\mathbf{R}_1,\mathbf{R}_2,\mathbf{R}_3,\mathbf{R}_4)}_{slmn} & \text{if } \sigma_1 = \sigma_3 \text{ and } \sigma_2 = \sigma_4 \ (\sigma_1 \neq \sigma_2) \\ -\frac{1}{2}\overline{V}^{(\mathbf{R}_2,\mathbf{R}_1,\mathbf{R}_3,\mathbf{R}_4)}_{lsmn} & \text{if } \sigma_1 = \sigma_4 \text{ and } \sigma_2 = \sigma_3 \ (\sigma_1 \neq \sigma_2) \\ 0 & \text{otherwise} \end{cases}, \tag{127}$$

respectively. Here, $\overline{V}^{s,(\mathbf{R}_1,\mathbf{R}_2,\mathbf{R}_3,\mathbf{R}_4)}_{slmn} = \big[\overline{V}^{(\mathbf{R}_1,\mathbf{R}_2,\mathbf{R}_3,\mathbf{R}_4)}_{slmn} - \overline{V}^{(\mathbf{R}_2,\mathbf{R}_1,\mathbf{R}_3,\mathbf{R}_4)}_{lsmn}\big]/2$. Each site-mode-spin triplet $(\mathbf{R}_i, i, \sigma_i)$ is then mapped to a single-index $\alpha_i \in \{1, ..., 2M\}$, with M the number of complex fermion modes per motif, so that the motif Hamiltonian can be written as

$$H = \sum_{\alpha,\beta} \overline{T}_{\alpha\beta} w^\dagger_\alpha w_\beta + \sum_{\alpha,\beta,\gamma,\delta} \overline{V}_{\alpha\beta\gamma\delta} w^\dagger_\alpha w^\dagger_\beta w_\gamma w_\delta. \tag{128}$$

Finally, as discussed in Supplementary Note 3 B, we introduce the Majorana basis operators as $w_\alpha = (\gamma_\alpha + i\overline{\gamma}_\alpha)/2$ and $w^\dagger_\alpha = (\gamma_\alpha - i\overline{\gamma}_\alpha)/2$, in terms of which the full motif Hamiltonian reads

$$H_M = \sum_{k\in\{0,1\}^{2M}} \alpha_k \prod_j \gamma_j^{k_{2j}} \overline{\gamma}_j^{k_{2j+1}} \ , \ |k| \in \{2, 4\}. \tag{129}$$

This can then be used as an input for the VQE and TDS algorithm described in Supplementary Note 4 and Supplementary Note 5. As discussed in Supplementary Note 5 B, Eq. (129) can be used to tile a system of any size, without increasing the depth of the layer of quantum gates that implements these interactions.

C. Circuit analysis

In this section we apply the circuit compilation techniques described throughout this manuscript to a series of materials, to assess the feasibility of simulating those materials on quantum hardware. We begin in Supplementary Note 6 C 1 by analysing strontium vanadate ($SrVO_3$) in detail, since we have described it completely in Supplementary Note 6 B, followed by four other materials in Supplementary Note 6 C 3. Finally, we analyse the number of measurement rounds that would be necessary to approximate the observables of interest for each material, under the set of measurement strategies described in Supplementary Note 4 D.

1. *Strontium vanadate*

Having the motif Hamiltonian of $SrVO_3$ in Majorana form as above, we map it to Pauli operators following the hybrid encoding introduced in Supplementary Note 3. To do this, we first recall that the hybrid encoding acts on the sites of a Cartesian grid. Thus, we have to consider the Cartesian motif introduced in Supplementary Note 2 C 1.

The latter contains N_C site, of which $N_D = N_C - N_{\text{cells/motif}}$ are not contained in the material real-space motif. This results in $M_D = N_D N_{\text{modes/cell}}$ additional complex fermion modes, with $N_{\text{modes/cell}} = 2N_{\text{orbitals/cell}}$ for a total of $M_C = M + M_D$ modes. The latter do not enter the motif Hamiltonian and, therefore, there are no interactions involving such modes. However, they must be kept in order to apply the tiling algorithm described in Supplementary Note 5 B, introducing a qubit overhead of $2M_D$. In particular, the mapping of the order $n = 1$ motif of $SrVO_3$ (consisting of the black and red sites in Supplementary Figure 30) to the corresponding $3 \times 3 \times 3$ Cartesian motif is shown in the bottom panel of Supplementary Figure 33.

Each site of the Cartesian motif is labeled by a single site-index $x \in \{\,0, ..., N_C - 1\,\}$, with $x = 0$ denoting the central cell, $x \in \{\,1, ..., 6\,\}$ labeling the 6 nearest neighbours and $x \in \{\,7, ..., 26\,\}$ corresponding to the $N_D = 27 - 7 = 20$ additional sites forming the Cartesian motif. Since $SrVO_3$ has a cubic lattice, the nearest-neighbour order of the sites is the same in the real-space and Cartesian motif, i.e., nearest neighbours of order n in real space are mapped onto nearest neighbours of order n in the Cartesian grid.

The $2M_C$ Majorana modes of the Cartesian motif are then encoded into qubits via the hybrid encoding discussed in Supplementary Note 3. The total number of qubits required to simulate the Cartesian motif is $N_Q = 2M_C + N_F$, with N_F being the number of auxiliary face qubits. After that the circuit is compiled with respect to the algorithm of interest, e.g., VQE. As outlined in Supplementary Note 5, a series of compilation routines are applied to the set of interactions to derive the final circuit, including the fswap network described in Supplementary Note 5 A. As explained

	Algorithm	Onsite/NN Interactions	Swaps	NNN+ Interactions	Swaps	Total
$SrVO_3$	TDS	72	336	700	0	1108
	TDS*	552	0	700	0	1252
	VQE	72	336	476	0	884
	VQE*	555	0	476	0	1031

Supplementary Table 6. Circuit depth analysis for $SrVO_3$, compiling VQE and TDS. Each algorithm is compiled with and without the use of fswap networks (asterisks denote the fswap network compilation is omitted). We report the breakdown of the circuit depth into the implementation of interactions and swaps. Interactions and depths are reported separately for (i) onsite and nearest-neighbour (NN) terms, which can be tiled generically across the encoding and therefore run in parallel, and (ii) next-nearest-neighbour and beyond (denoted NNN+) terms, which can not be tiled on the encoding and therefore must be costed explicitly. Properties of $SrVO_3$ are given in Supplementary Table 9, and the number of terms of each Hamiltonian type are listed in Supplementary Table 11.

there, it is important to separate the interactions based on the sites of the real-space motif upon which they act. This can be seen in the diagram at the bottom of Supplementary Figure 33, where we show the location on the Cartesian motif of the real-space interaction terms involving the central cell located at 0 and the nearest neighbour one at x (denoted by the pair $(0, x)$), for $x \in \{1, ..., 6\}$.

In Supplementary Figure 33**(a)** we show the circuit depth breakdown (for onsite and nearest-neighbour terms for simplicity) by layer without including fswaps, similar to the analysis in Supplementary Note 6 A. In Supplementary Figure 33**(b)** we can see how the circuit layers are distributed among terms acting on various sites. In this material, all interactions involve at most two sites: the central site and one neighbour. In contrast, including fswaps, we see in Supplementary Figure 33**(c)** that the depth of each layer reduces, but we have more layers. In this case, the majority of circuit depth is spent on implementing fswap operations, also listed in Supplementary Table 6. In general, it is difficult to know a priori whether the fswap network will be beneficial to the depth. In our case, we run the circuit compiler both with and without the fswap network to determine the shortest circuit which should simulate the same process.

In the same way we can employ the circuit compiler to evaluate different algorithms; in particular, we consider VQE and TDS, and compile both with and without the aid of an fswap network. This is reported in Supplementary Table 6. We see that the swap network yields lower circuit depths in each case, as elucidated in Supplementary Figure 33. Overall the shortest available circuit corresponds to VQE including an fswap network: for the remainder of this section we will consider VQE in particular, but it is straightforward to run the alternative compilations, which are listed in Appendix E 2.

2. Trotter error

In the case of TDS we may also compute an estimate of the Trotter error. This is done using an upper bound estimate given by Eq. (26) in [90],

$$\epsilon \leq C_1 \frac{T\delta^p}{(p+1)!} + C_2 \frac{T}{\delta} I(N) \tag{130}$$

with

$$C_1 := npB_p^2 \Lambda^{p-1} N[MH_p - B_p + B_p(N/\Lambda)]^{p-1} \tag{131}$$

$$C_2 := nB_p^2 (MH_p\Lambda)^p N[(S_pM)^2 - S_pM]. \tag{132}$$

Here, T is the target time; p is the Trotter order (currently the compiler is only designed to handle $p = 1$); N is the largest number of terms in a given mutually commuting grouping; M is the number of mutually commuting groupings; Λ is a bound on the largest norm of any mutually commuting

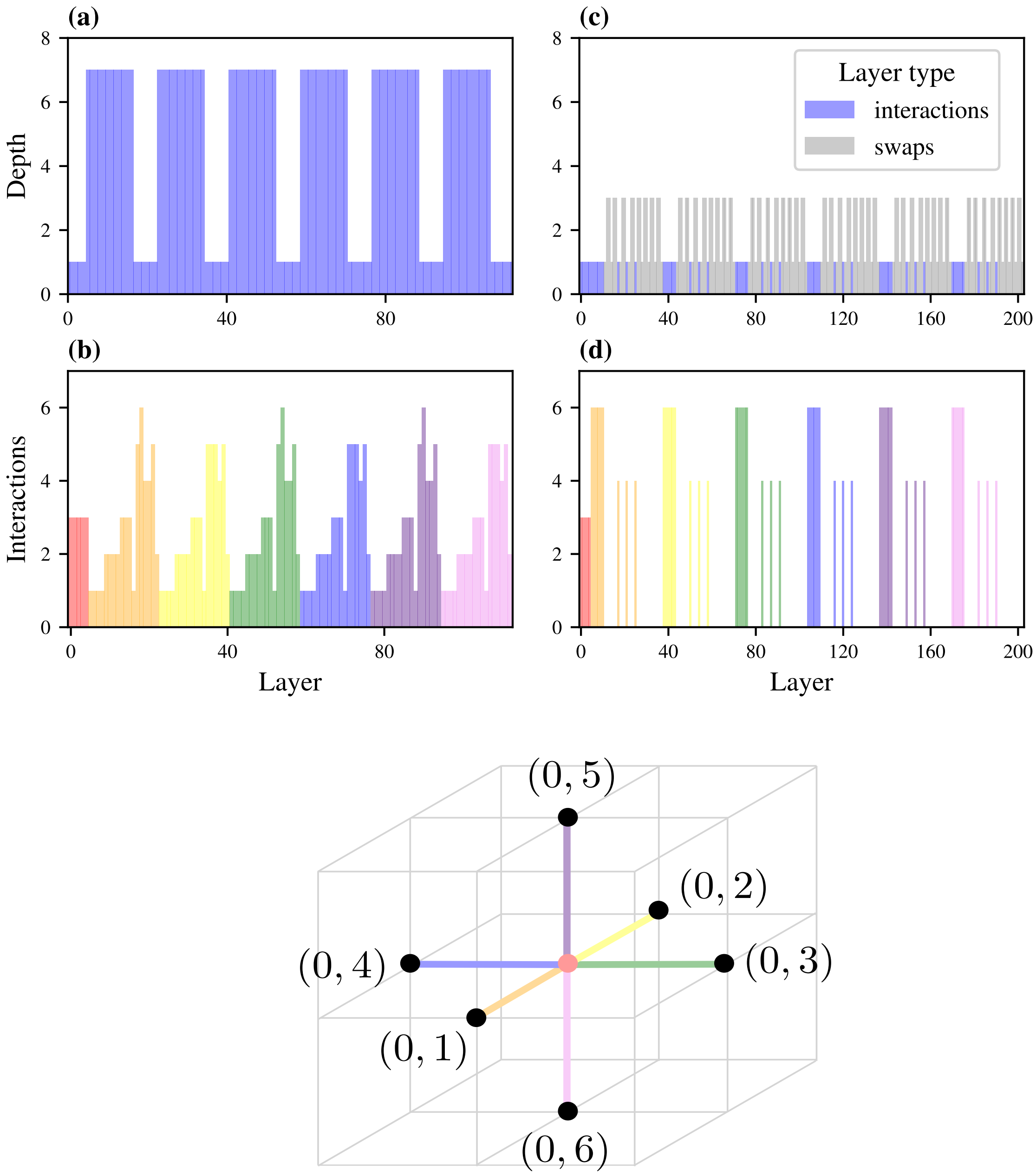

Supplementary Figure 33. Circuit breakdown for $SrVO_3$ for onsite and nearest neighbour terms. Top: **(a)** Circuit depth cost per layer in an approach without fswaps. **(b)** The number of interactions implemented in each layer. **(c)** Circuit depth cost per layer using fswaps. **(d)** Breakdown of the number of interactions per layer using fswaps. Here, layers with zero interactions implement fswaps. The colour code in **(b)** and **(d)** is the same and denotes the pair of sites $(0, x)$ involved in an interactions, as shown in the bottom diagram, with x increasing to the right. Bottom: Mapping of the $SrVO_3$ motif of order $n = 1$ (consisting of the cells highlighted by the pink and black dots) to the $3 \times 3 \times 3$ Cartesian motif (compare with Supplementary Figure 30). Each coloured line denote the real-space interaction $(0, x)$ between the central cell (labeled by 0 in the Cartesian motif) and the $x-$th neighbouring cell. The compiler groups terms by their sites in advance, so the final circuit layering will have sections dedicated to each site grouping.

grouping; δ is the Trotter step size; n is the maximum number of terms from a single grouping that do not commute with a given term in another grouping, and B_p, H_p, S_p, and I are defined in the original work. This equation may be inverted to compute the required circuit depth of a TDS simulation for a given target time T and Trotter error ϵ.

It is important to emphasize here that the bounds on the Trotter error will depend on the order in which the terms in the Hamiltonian are executed. However the order of execution will depend on the choices taken by the compiler. And the quality of the Trotter error plays a role in the multi-layer circuit depth of executing time dynamics for some fixed time. As such one may wish to prioritize minimizing Trotter error over minimizing the circuit depth of a single layer by modifying the execution of the compiler. One concrete way this can be done is by decomposing into layers of mutually commuting terms first before proceeding with executing any swap network protocols.

We include here a comparative analysis of two compiler strategies to examine their relative efficacy in reducing overall circuit depth of TDS. One strategy prioritizes the circuit depth of a single layer by decomposing into commuting layers after performing swap networks, and the other prioritizes Trotter error by decomposing into commuting layers before performing swap networks. In this case we fix the Trotter order $p = 1$. Given a single layer circuit produced by the compiler using a particular compiler strategy, and given a fixed Trotter error, we numerically invert equation (130) to compute the requisite Trotter step size δ. For order 1 Trotter formulae, the total circuit depth to evolve for time T is given by DT/δ where D is the depth of a single layer. Thus we can compare the quality of different compiler choices by considering the Trotter ratio D/δ. This is shown in Supplementary Figure 35. It is important to emphasize that these values do not indicate the actual circuit depth of a practical time dynamics simulation, since the choice of evolution time T will depend on the details of the algorithm as well as the spectral norm of the Hamiltonian, which in our analysis has not been normalized.

Additionally we include here a comparison of how different Trotter orders p influence total circuit depth for $SrVO_3$ – see Supplementary Figure 34. This is calculated by upper bounding the circuit depth using the first order Trotter layers computed by the compiler. It is important to emphasize that these plots are meant to illustrate the effect of different Trotter orders, and are not necessarily prescriptive of a likely range of useful target times. This is because the particular target time may depend on the application, and additionally the Hamiltonians are neither normalized, nor is an appropriate characteristic time scale for the material known ahead of time.

3. *Further materials*

By focusing on VQE and including an fswap network in each case, we can follow the same procedure outlined for $SrVO_3$ in Supplementary Note 6 C 1 to construct circuits for the simulation of a number of materials, summarised in Supplementary Table 7. We choose this as a representative sample of materials across a minimal but technologically relevant chemical space, spanning from light materials such as hydrogen and lithium to well known correlated ions like copper. Again we see that the circuit depth is dominated by the cost of implementing NNN+ terms, owing to the explicit inclusion of all terms in the lattice outlined in Supplementary Note 5 B 1, while onsite/NN terms are relatively cheap because they can be performed in parallel (Supplementary Note 5 B). In particular, the larger circuit depth observed for the superconductor H_3S relative to the other materials observed is due to the large bandwidth of states considered in the active space, which spans ≈ 40 eV, roughly four times as large as of the other materials under consideration. This requires many more NNN+ terms to reproduce the Kohn-Sham electronic bandstructure within the considered tolerance. These resource requirements are orders of magnitude lower than previous efforts, and can be improved further by (i) reducing the number of NNN+ terms through effective screening; (ii) invoking a translationally invariant fermionic encoding that integrates fully the connectivity structure of the Hamiltonian, beyond nearest neighbours such that the NNN+ terms are generically tilable in the same way as the onsite/NN terms.

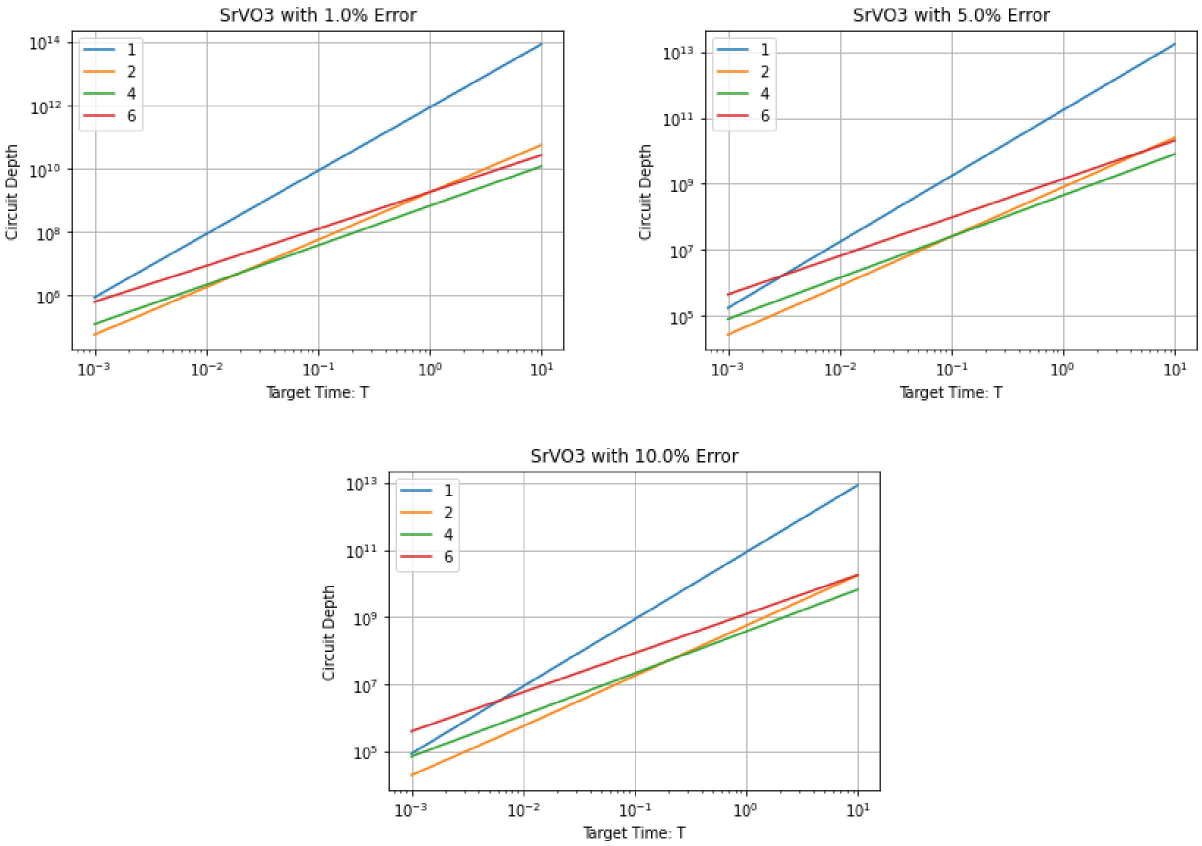

Supplementary Figure 34. A comparison of the effect of Trotter order on the total depth of a time dynamics simulation of $SrVO_3$ for a range of target simulation times and a range of target Trotter errors, using the Trotter layer circuit compiled by prioritizing Trotter error. These figures are meant to illustrate relative performance of different Trotter orders, and do not necessarily correspond to the timescales which may be desired in experiments.

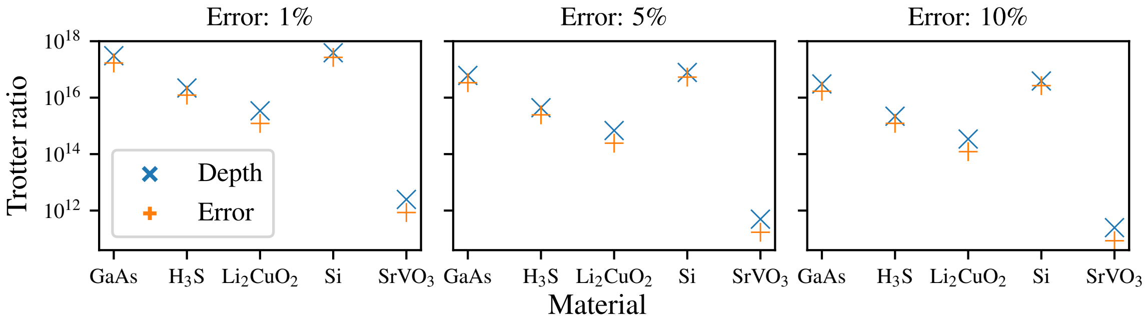

Supplementary Figure 35. Trotter ratio for each material, showing the outcomes of the circuit compiler when alternative decomposition strategies are employed, namely which prioritise the circuit depth or Trotter error of a single layer. Trotter ratio is defined in the text as D/δ, where D is the circuit depth computed by the compiler for a single layer and δ is the Trotter step size. These figures are meant to illustrate relative performance of different compiler routines, and not as indicators of expected circuit depth. Furthermore this analysis only holds for Trotter order $p = 1$, which does not yield optimal Trotter error rates. For alternative analyses which consider $p > 1$ see Supplementary Figure 34.

	Qubits	Onsite/NN Interactions	Swaps	NNN+ Interactions	Swaps	Total
GaAs	1120	98	564	7191	0	7853
H_3S	1870	126	1088	36126	0	37340
Li_2CuO_2	1024	123	1544	6710	0	8377
Si	1120	112	592	7857	0	8561
$SrVO_3$	180	72	336	476	0	884

Supplementary Table 7. Circuit resources required for a representative set of materials. We list the number of qubits and circuit depth required for a single layer of VQE. The number of qubits reported is a function of both the number of modes of the Cartesian motif and of the number auxiliary face qubits introduced by the hybrid encoding. We report the breakdown of the circuit depth into the depth spent implementing interactions/swaps by whether the terms are onsite/NN or NNN+. The circuit depths reported refer to a single layer of VQE, without accounting for state preparation. Properties of the listed materials are given in Supplementary Table 9, and the number of terms of each Hamiltonian type are listed in Supplementary Table 11.

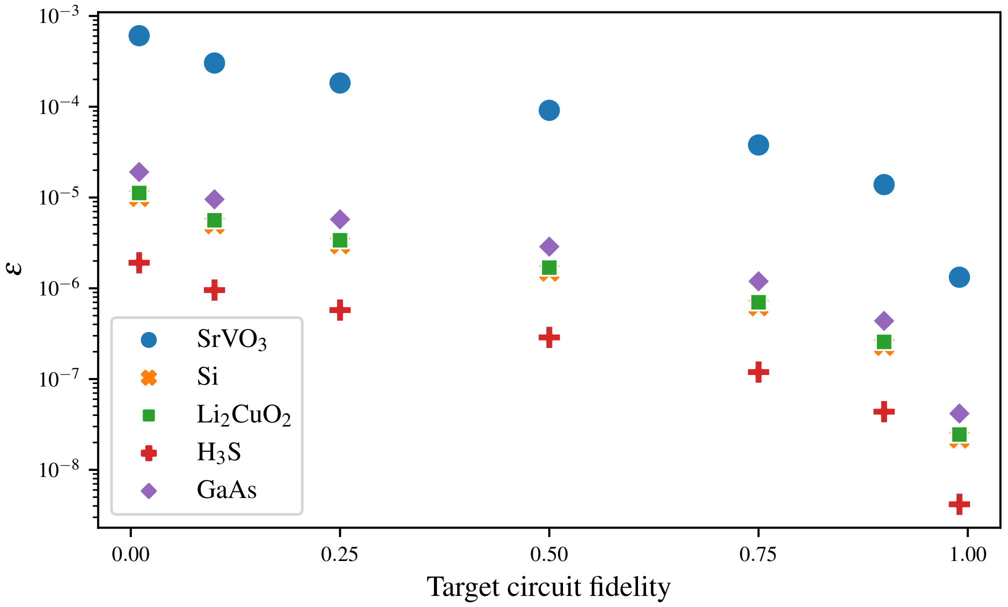

Supplementary Figure 36. Estimate of the maximum two qubit gate error ε allowed to implement a single layer of VQE within a target circuit fidelity. The number of two qubits for each materials is given in Table 1 of the main text.

4. Hardware requirements

To determine a naive estimate of the hardware requirements for the implementation of a single layer of VQE, we assume that the overall circuit fidelity is given by $\mathcal{F} = (1-\varepsilon)^{N_{2q}}$, with ε the typical two qubit gate error and N_{2q} the number of the required two qubit gates (see Table 1 of the main text). State-of-the-art error mitigation techniques allow the recovery of useful results even with circuit fidelities as low as 10% [91, 92]. In Supplementary Figure 36 we show how the requirements on the two qubit gate ε error change as a function of the target circuit fidelity.

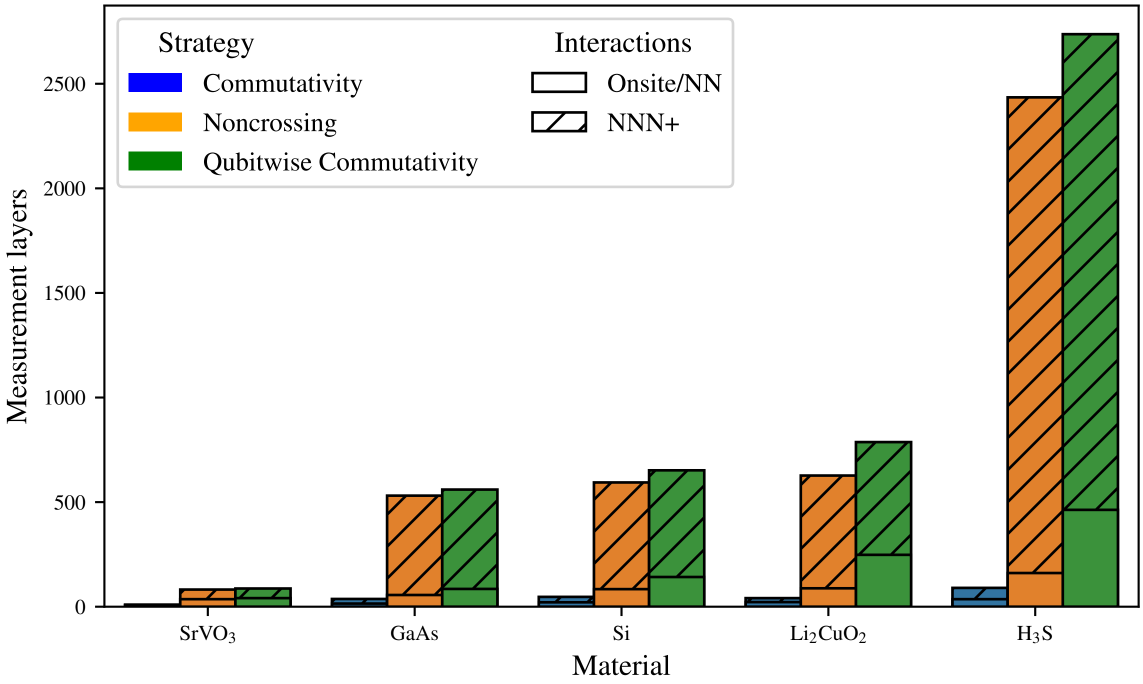

Supplementary Figure 37. Measurement layers: the number of different measurements required in order to measure the terms included in the Hamiltonian. Various measurement strategies are presented, split by the types of Hamiltonian terms involved in each measurement layer. The noncrossing case always measures NNN+ terms using a QWC strategy. Properties of the listed materials are given in Supplementary Table 9. These data are listed explicitly in Supplementary Table 12.

5. *Measurement rounds*

We analyse the number of measurement layers required for each of the studied materials using each of the measurement strategies described in Supplementary Note 4 D, again breaking into the subset required for onsite/NN terms and NNN+ terms in Supplementary Figure 37 (and listed in Supplementary Table 12). There are three key messages:

(i) The commuting terms measurement strategy is optimal, and far superior to alternative methods, as expected. However, it is difficult to prepare the appropriate bases in which to measure the sets of commuting terms in practice.

(ii) The noncrossing-terms strategy outperforms qubitwise commutativity, especially when considering only onsite/NN terms.

(iii) When a noncrossing or QWC strategy is used, similarly to the circuit depth, the NNN+ terms demand many more measurement layers than the onsite/NN counterpart. Currently, even if a noncrossing strategy is used for onsite/NN terms, we are forced to use QWC for NNN+ terms. The measurement cost could be substantially reduced by developing an appropriate generalisation of the noncrossing concept to these terms.

Supplementary Notes 7 – Outlook

Simulating materials is a promising application for quantum computers. The progress reported here combines a number of complementary approaches across the full quantum materials simulation

stack that, when combined together, dramatically reduce the quantum circuit depth requirements by orders of magnitude compared to naive baseline estimates. Crucially, the design process produces quantum circuit depths for Trotter and VQE layers which are independent of the material's size by taking advantage of the locality of material Hamiltonians.

We expect that our proposed framework for materials simulation on quantum computers can be enhanced further by continuing to incorporate physically motivated structure into the choices of fermionic encodings, basis representations, and swap network protocols. Examples of such considerations which may be particularly fruitful include

- a more sophisticated incorporation of point and spin symmetries into the choice of fermionic encoding;
- a more careful consideration of the choice of ordering of modes in the hybrid chains;
- a more aggressive analysis of how many terms in the Hamiltonian may be truncated – in particular, the systematic incorporation of electron screening effects in many materials truncates the long-range effects of the Coulomb interaction, which can be computed using classical approaches such as RPA [93] and constrained DFT [94]. It is our expectation that this will decrease the number of non-local quartic interactions needed to be considered in the quantum simulation;
- further localisation of the single-particle Wannier states, such as with the recently proposed selected columns of the density matrix maximal localisation technique [95];
- a tighter feedback loop between the choice of fermionic basis and the resulting form of the qubit Hamiltonian;
- properly accounting for the translational symmetries of NNN+ terms in the fermionic encoding;
- identifying early termination heuristics for the fswap networks.

One consequence of this work is the identification of certain materials, from the small set we have considered, which are particularly well suited to quantum simulation due to the details of their physics – such as $SrVO_3$. Beyond reducing circuit depths and improving error mitigation techniques, identifying the appropriate physical systems which are best suited for simulation on NISQ devices is essential. The development of the tools described in this work can be used to allow the application of data-driven and high-throughput techniques to understand the classes of materials most amenable to quantum simulation.

It is possible that the strategies employed in this work to tackle materials may also be applicable to other quantum systems such as chemicals – particularly those with some kind of extended spatial structure. We also mention that the computation of ground state properties for materials may be more efficiently realised on classes of model Hamiltonians, such as those with Slater-Kanamori interactions, within approximate embedding formalisms, which have enjoyed recent success using classical algorithms [36], but are ultimately constrained by the exponentially scaling Hilbert space with their system size.

Finally, this work has made some assumptions about the structure of the quantum hardware, the most significant of which is the assumption of all-to-all hardware connectivity. This assumption, while valid for certain architectures (such as some ion traps), does not hold universally; it was made largely to reduce the complexity of performing the analysis, so as to more rapidly demonstrate the benefits of the strategies we are proposing. We plan to continue to iterate on the design of the compiler to incorporate specific hardware layouts, and introduce additional strategies to improve circuit depths in these cases. However it is important to recognise that the horizon of quantum hardware design is extremely broad, and it is likely that the development of hardware may well be done in an anticipation of specific algorithmic requirements. Developing tools such as the one given here can help better identify what these requirements may look like.

Our results show that considering seriously the structure of the physical problem at hand and incorporating these considerations into the design of quantum algorithms can dramatically accelerate progress towards quantum advantage.

Appendix

A. Baseline for qubit requirements and gate depth of materials

Here, we briefly give a naive estimate of the resources (i.e., qubit number, circuit depths, and two-qubit gate count) required to simulate a material on a quantum computer using general existing methods and without taking advantage of the tailored approach we exploited in the main text.

We consider a material with periodic boundary conditions, discrete translational symmetry, and lattice volume V (i.e., with a total of V unit cells). In the spirit of the Born-Oppenheimer approximation [3], we assume a stationary atomic configuration and we are interested in simulating the electronic degrees of freedom of the system. For the sake of simplicity, we may choose to describe the material in the Bloch basis introduced in Supplementary Note 2. In analogy with the procedure described in Supplementary Note 2 F 1, we assume that we are able to identify a set of active bands where the relevant physics takes place. In this case we are concerned with the Hamiltonian restricted to the m modes indexed by those bands. These m bands are determined by identifying the last occupied atomic orbital for each atom in the material and then including all occupied orbitals for each atom. Accounting for spin, the total number of active fermionic modes is $m = 2Vb$, where b is the number of bands.

One may represent the fermionic system on the qubits of a quantum computer using the Jordan-Wigner (JW) transform in accordance with some pre-chosen linear ordering of the fermionic modes (see Supplementary Note 3). This requires one qubit per mode. We denote by H' the Hamiltonian expressed as a sum of Pauli operators on the qubit system corresponding to the JW transform of H.

We now wish to estimate the circuit depth of a potential quantum algorithm for such a representation. As a benchmark we consider the circuit depth of the following unitary circuit, as used in the main text:

$$U = \prod_j \exp(i\alpha_j H'_j), \tag{A1}$$

where we are free to choose the ordering of the product. Circuits of this type appear as subroutines in both VQE (under a Hamiltonian variational ansatz) and time dynamic simulation (TDS). In both cases, these subroutines are repeated multiple times, introducing an additional multiplicative overhead to the circuit depth, which we will not detail here.

A consequence of choosing the JW transform is that many of the terms in H' operate on a large number of qubits. In particular, fermionic operations corresponding to interactions between fermionic modes far from each other in the aforementioned linear ordering precipitate costly circuit decompositions of individual $\exp(i\alpha_j H'_j)$ terms.

In this Appendix we consider two previously known methods of implementing the desired interactions. Here, we will assume for simplicity that H' consists of quartic terms only, because in practice quadratic terms can likely be implemented at a lower-order cost during the course of the algorithm to implement the quartic terms. The first is simply to implement the terms in H' in sequence, via the logarithmic-depth circuit for computing parities described in Supplementary Note 4 A. Each term can be implemented in depth at most $2\lceil \log_2 m \rceil - 1$ (given that we are allowed all-to-all interactions) leading to an overall depth of at most $T(2\lceil \log_2 m \rceil - 1)$ for a Hamiltonian with T terms. Note that it may be possible to reduce the complexity somewhat by implementing some of these terms in parallel, and using the fact that many of the terms act nontrivially on fewer than m qubits; we do not explore these further here.

The second method was proposed in [62], and allows all quartic interactions to be implemented in quantum circuit depth approximately $O(m^3)$. More precisely, assuming that each quartic term requires 2-qubit depth 3 (as used in Supplementary Note 4 A), the overall 2-qubit gate depth is at least approximately

$$0.76m^{3.06} + \frac{12T}{m}, \tag{A2}$$

where we estimate the total cost by summing the cost of swap layers from Fig, 7E of [62] (taking the fit up to 400 qubits), and make the optimistic assumption that the T terms can be optimally

parallelised in between the swap layers, such that we implement $m/4$ of them at each layer, each with 2-qubit gate depth 3.

It remains to compute the total number of terms T.

In terms of the complex fermion creation and annihilation operators $f^\dagger_{\mathbf{k},b,\sigma}$ and $f_{\mathbf{k},b,\sigma}$, the quartic interactions in the Bloch basis are (see, e.g., second term in Eq. (26))

$$H_{\text{int}} = \sum_{\mathbf{k}_i, b_i} V^{\mathbf{k}_1\mathbf{k}_2\mathbf{k}_3\mathbf{k}_4}_{b_1b_2b_3b_4} \delta_{\mathbf{k}_1+\mathbf{k}_2,\mathbf{k}_3+\mathbf{k}_4} \sum_{\sigma,\sigma'} f^\dagger_{\mathbf{k}_1,b_1,\sigma} f^\dagger_{\mathbf{k}_2,b_2,\sigma'} f_{\mathbf{k}_3,b_3,\sigma'} f_{\mathbf{k}_4,b_4,\sigma}, \tag{A3}$$

where $\mathbf{k}_i$ are Bloch momenta, b_i are band indices and σ is the spin. Here $\delta_{\mathbf{k}_1+\mathbf{k}_2,\mathbf{k}_3+\mathbf{k}_4}$ enforces explicitly the Bloch momentum conservation (up to lattice vectors). To ease the count, it is useful to write H_{int} in terms of singlet and triplet scattering terms (valid in presence of time reversal symmetry)

$$H_{\text{int}} = \sum_{\alpha_i} \delta_{\mathbf{k}_1+\mathbf{k}_2,\mathbf{k}_3+\mathbf{k}_4} \left(V^+_{\alpha_1\alpha_2\alpha_3\alpha_4} \psi^\dagger_S(\alpha_1,\alpha_2)\psi_S(\alpha_3,\alpha_4) + V^-_{\alpha_1\alpha_2\alpha_3\alpha_4} \sum_{a=0,\uparrow,\downarrow} \psi^\dagger_a(\alpha_1,\alpha_2)\psi_a(\alpha_3,\alpha_4) \right),$$

where we have defined the super-index $\alpha_i = (\mathbf{k}_i, b_i)$ that can take Vb different values and $V^\pm_{\alpha_1\alpha_2\alpha_3\alpha_4} \equiv \frac{1}{2}(V^{\mathbf{k}_1\mathbf{k}_2\mathbf{k}_3\mathbf{k}_4}_{b_1b_2b_3b_4} \pm V^{\mathbf{k}_1\mathbf{k}_2\mathbf{k}_4\mathbf{k}_3}_{b_1b_2b_4b_3})$. The singlet and triplet operators are

$$\psi_S(\alpha_1,\alpha_2) = \frac{1}{\sqrt{2}}(f_{\mathbf{k}_1,b_1,\uparrow} f_{\mathbf{k}_2,b_2,\downarrow} - f_{\mathbf{k}_1,b_1,\downarrow} f_{\mathbf{k}_2,b_2,\uparrow}), \tag{A4}$$

$$\psi_0(\alpha_1,\alpha_2) = \frac{1}{\sqrt{2}}(f_{\mathbf{k}_1,b_1,\uparrow} f_{\mathbf{k}_2,b_2,\downarrow} + f_{\mathbf{k}_1,b_1,\downarrow} f_{\mathbf{k}_2,b_2,\uparrow}), \tag{A5}$$

$$\psi_\sigma(\alpha_1,\alpha_2) = f_{\mathbf{k}_1,b_1,\sigma} f_{\mathbf{k}_2,b_2,\sigma}. \tag{A6}$$

For the singlet operator above, we can choose $\binom{Vb}{2}$ values for the pair of indices α_1 and α_2 that give a different operator plus Vb choices when $\alpha_1 = \alpha_2$. On the other hand, for any of the triplet operators one can only choose $\binom{Vb}{2}$ values of the pair (α_1, α_2) that generate a different operator. Note that $\psi_a(\alpha,\alpha) = 0$.

Then, the overall bound is given by

$$T = \frac{1}{V}\left[\left[\binom{Vb}{2} + Vb\right]^2 + 3\binom{Vb}{2}^2\right] = V^3b^4 - V^2b^3 + Vb^2. \tag{A7}$$

Here, the reduction by a factor of V is due to lattice momentum conservation. Additional symmetries of the Hamiltonian may introduce more savings in the number of terms (with the size of the savings being proportional to the size of the symmetry), but few are likely to be as large as the lattice translation symmetry.

Using the fact that a Hamiltonian with T terms can be implemented with an overall depth of at most $T(2\lceil\log_2 m\rceil - 1)$ and Eq. (A2), we can derive two upper bounds on the quantum circuit depth. In terms of V and b, they take the forms

$$\text{UB}_1 = (V^3b^4 - V^2b^3 + Vb^2)(2\lceil\log_2(Vb)\rceil - 1) \tag{A8}$$

$$\text{UB}_2 = 6.34(Vb)^{3.06} + 6(V^2b^3 - Vb^2 + b), \tag{A9}$$

respectively. We can also put a crude lower bound on the quantum circuit complexity of any method based on the JW transform. If we have T quartic terms and m qubits, we can implement at most $m/4$ terms at each step. Assuming again that each quartic term requires 2-qubit depth 3, we require 2-qubit gate depth of at least $12T/m$, or in terms of V and b, at least

$$\text{LB} = 6(V^2b^3 - Vb^2 + b). \tag{A10}$$

In order to compare these naive estimates with the results reported in Supplementary Note 6, in Table 1 of the main text we considered a system consisting of $V = 3^3$ unit cells for $SrVO_3$ and

Element	electron config.	orbitals considered
Gallium	[Ar] $3d^{10}4s^24p^1$	3 (from p)
Arsenic	[Ar] $3d^{10}4s^24p^3$	3 (from p)
Hydrogen	$1s^1$	1 (from s)
Sulfur	[Ne] $3s^23p^4$	3 (from p)
Lithium	[He] $2s^1$	1 (from s)
Copper	[Ar] $3d^{10}4s^1$	6 (from d and s)
Oxygen	[He] $2s^22p^4$	3 (from p)
Silicon	[Ne] $3s^23p^2$	3 (from p)
Strontium	[Kr] $5s^2$	1 (from s)
Vanadium	[Ar] $3d^34s^2$	6 (from d and s)

Supplementary Table 8. Electron orbitals of different elements. To obtain the number of bands for the different materials presented in Table 1 of the main text, we use the orbitals in the valence shell for each atom and multiply it by the number of times an element appears in the the chemical formula of the material. We use GaAs (Ga+As); H_3S (3H+S); Li_2CuO_2 (2Li+Cu+2O); $SrVO_3$ (Sr+V+3O); Si (Si).

$V = 5^3$ for the rest of the materials (see Table 9 below) and reported the better of the two upper bounds in each case. These sizes have been chosen according to the procedure described in Sec. 6 B 1. Note that, thanks to the nearsightedness of electronic systems, one can expect that such sizes for the motif are sufficient to compute estimates of local electronic properties with an accuracy of a few percentage points [96, 97]. On the other hand, the number of fermionic modes implied by such motifs is well beyond the capabilities of current state-of-the-art numerical techniques. For instance, in Ref. [98], in which several numerical methods for the simulation of a standard 2D Hubbard model are compared, the largest instance that has been examined consisted of a few hundred fermionic modes.

The assumptions that we use for the number of bands are shown in Table 8. Although other estimates for the number of bands are possible, note that for a large piece of material $V \gg b$ and dominates in (A8).

Upper and lower bounds on the number of gates can be obtained following similar steps. In particular, each term in H' can be implemented in at most $2m-3$ two-qubit gates. See Supplementary Figure 16, where we also assumed that the single qubit rotation and two CNOT gates at its sides can be combined into a single two-qubit gate. Assuming that the terms in H' are implemented in sequence, this would lead to an overall gate count

$$\mathrm{GUB}_1 = (V^3b^4 - V^2b^3 + Vb^2)(2m-3) \tag{A11}$$

On the other hand, following the method of Ref. [62] and assuming that each quartic term can be implemented with 3 two-qubit gates, we obtain

$$\mathrm{GUB}_2 = 0.76m^{3.06}\lfloor m/2 \rfloor + 3(V^3b^4 - V^2b^3 + Vb^2), \tag{A12}$$

where we used that each swap layer consists of at most $\lfloor m/2 \rfloor$ gates of individual cost 1.

Finally, a lower bound on the gate count can be derived assuming that each of the T quartic term requires two-qubit gate depth 3. We obtain

$$\mathrm{GLB} = 3(V^3b^4 - V^2b^3 + Vb^2). \tag{A13}$$

B. Exponentially localized Wannier functions

Here we discuss the two conditions for the existence of maximally localized Wannier functions (MLWFs). Recalling the definition of the Wannier functions introduced in Supplementary Note 2 C, we have

$$\mathcal{W}^{\mathbf{0}}_{s,\sigma}(\mathbf{r}) = \sum_{\mathbf{k},n} u_{\mathbf{k},n,\sigma}(\mathbf{r})U_{ns}(\mathbf{k})e^{-i\mathbf{k}\cdot\mathbf{r}} \equiv \sum_{\mathbf{k}} v_{\mathbf{k},s,\sigma}(\mathbf{r})e^{-i\mathbf{k}\cdot\mathbf{r}}, \tag{B1}$$

where $v_{\mathbf{k},s,\sigma}(\mathbf{r})$ are quasi-Bloch functions. We can use the analyticity of the quasi-Bloch functions $v_{\mathbf{k},s,\sigma}(\mathbf{r})$ as a function of the crystal momentum $\mathbf{k}$ to show that the Wannier functions are localized, using the following result [16–18]

Theorem 1 (Cloizeaux): Let $f(\mathbf{k})$ be a function of the $n-$dimensional complex vector $\mathbf{k} = \mathbf{k}' + i\mathbf{k}''$ defined in the $n-$torus with periods $\mathbf{b}_j$ $(j = 1, \ldots, n)$, i.e., $f(\mathbf{k} + \mathbf{b}_j) = f(\mathbf{k})$. If $f(\mathbf{k})$ is an analytic function of $\mathbf{k}$ in a strip defined by $|\mathbf{k}''| < A$, with A a positive real number, then:

1. $f(\mathbf{k})$ can be expanded in a convergent Fourier series in this domain as

$$f(\mathbf{k}) = \sum_{\mathbf{R}} e^{i\mathbf{k}\cdot\mathbf{R}} g(\mathbf{R}), \tag{B2}$$

   where $\mathbf{R} = \sum_j n_j \mathbf{r}_j$ is a reciprocal lattice vector to $\mathbf{k}$, with $\mathbf{r}_j$ satisfying $\mathbf{b}_j \cdot \mathbf{r}_l = 2\pi\delta_{jl}$, and

2. the Fourier coefficients $g(\mathbf{R})$ satisfy $\lim_{|\mathbf{R}|\to\infty} e^{b|\mathbf{R}|} g(\mathbf{R}) = 0$ for any $0 < b < A$.

Conversely, if the Fourier coefficients have this asymptotic behavior, the series converges and is analytic in the region $|\mathbf{k}''| < A$.

Clearly, if we can show that a quasi-Bloch function $v_{\mathbf{k},s,\sigma}(\mathbf{r})$ is indeed an analytic function of the crystal momentum, then the corresponding Wannier function $\mathcal{W}_{s,\sigma}(\mathbf{r})$ will be exponentially localized as a function of $\mathbf{R}$, due to Theorem 1 above. The quasi-Bloch functions $v_{\mathbf{k},s,\sigma}(\mathbf{r})$ are associated with the single-particle energies, which are analytic except for points where the bands are degenerate. In that case, the energy surface in the complex plane has a branch-cut. We can define the projector onto the considered bands as

$$P(\mathbf{k}) = \frac{1}{2\pi i} \int_{\mathcal{C}(\mathbf{k})} \frac{dz}{z - H_0(\mathbf{k})}, \tag{B3}$$

where the contour $\mathcal{C}(\mathbf{k})$ encloses the bands in the active space (see Supplementary Figure 38). Here $H_0(\mathbf{k})$ is the non-interacting part of the Hamiltonian.

The gap A between the last band considered and the first band outside the active space determines the decay of the exponential localization of the Wannier functions. It has been shown [19] that if the sum of Chern numbers on the bands belonging to the active space is zero, then the quasi-Bloch functions exist. The Chern number of a group of bands is defined as

$$C_\ell = \frac{i}{2\pi} \sum_{i<j} \int_{BZ} B^{ij}(\mathbf{k}) dk_i \wedge dk_j, \tag{B4}$$

where the integration is performed in the Brillouin zone. The Berry connection $B^{ij}(\mathbf{k})$ characterizes the change of frame between different points in the Brillouin zone and is given by

$$B^{ij}(\mathbf{k}) = \mathrm{Tr}\left(P(\mathbf{k}) \left[\frac{\partial P(\mathbf{k})}{\partial k_i}, \frac{\partial P(\mathbf{k})}{\partial k_j} \right] \right). \tag{B5}$$

Therefore, the two following conditions are sufficient to ensure the existence of localized Wannier functions:

1. Vanishing Chern numbers. This condition is automatically satisfied in a system with time-reversal symmetry. Note that, however, systems without time-reversal symmetry can still have vanishing Chern number.

2. Existence of a large gap between the bands in the active space. Note that, in this case, the system does not necessarily represent an insulator, as the Fermi energy can lie within the active space, which is separated from the rest of the bands.

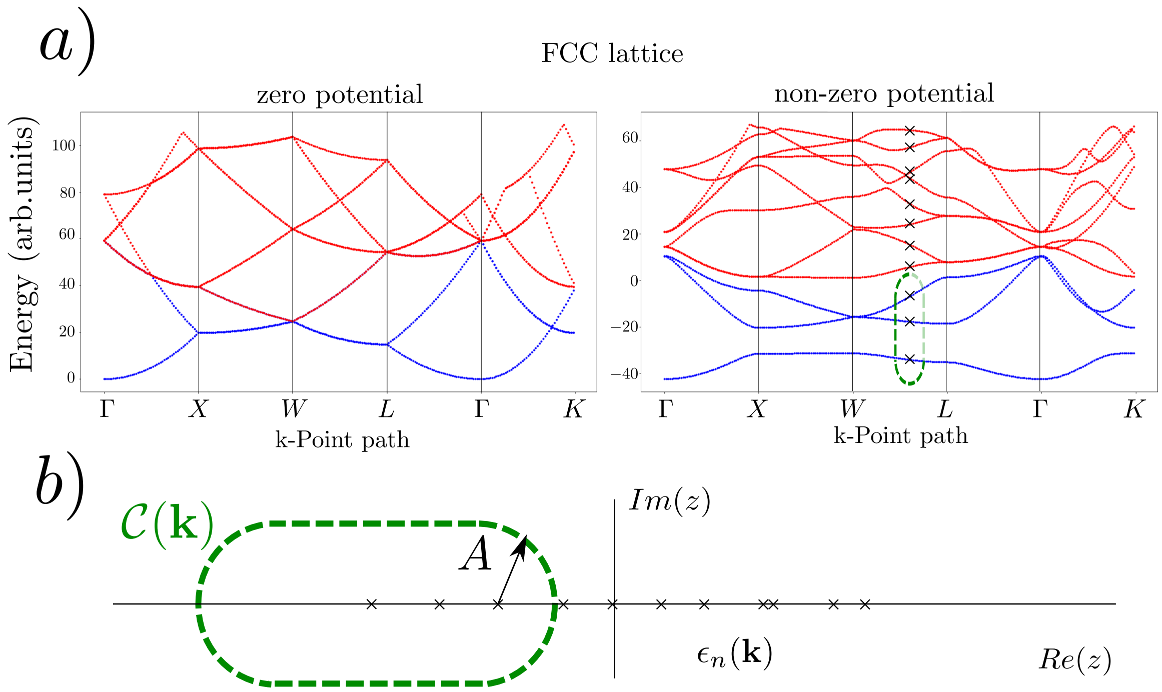

Supplementary Figure 38. **(a)** left. Band structure for face centered cubic (FCC) lattice, in the empty lattice approximation ($U_{\mathbf{G}} = 0$) across a high symmetry path. The three bottom bands are highly degenerate for many values of the crystal momentum. **(a)** right. Bands structure for FCC lattice, with $U_{\mathbf{G}} = 100 \sum_a \frac{e^{i\mathbf{r}_a \cdot \mathbf{G}}}{\mathbf{G}}$, where the sum runs over the atoms in the unit cell. We observe that the bands split at high symmetry points, and a gap (for each $\mathbf{k}$) develops between the three lower bands and the upper energy bands. Considering each band as the real cross-section of a complex eigenenergy parameterized by $\mathbf{k} = \mathbf{k}' + i\mathbf{k}''$, we can define a projector onto the lower three bands by using the Riesz projector defined in Eq. (B3) with $\mathcal{C}(\mathbf{k})$ given by the dashed green contour. **(b)** The integration contour $\mathcal{C}(\mathbf{k})$ for the Riesz projector. The width of the strip in the imaginary direction where the projector is analytic is determined by the gap A between the active bands and the rest.

C. Optimality of the Jordan-Wigner transform and fswap networks

Here we show that, perhaps surprisingly, if our goal is to implement time-evolution according to an arbitrary set of quadratic fermionic interactions (in an order of our choice), then an algorithm combining the Jordan-Wigner (JW) transform and fermionic swap (fswap) networks is close to optimal (in a particular sense). This algorithm, which was described in [11], was proven optimal among fswap network algorithms within the JW transform in [52]; here, by contrast, we prove it is close to optimal among all algorithms (of a somewhat restricted form) within any encoding.

To achieve this, we consider the following setup:

1. We are given a fermionic Hamiltonian on m modes of the form
$$H = \sum_{j,k} H_{jk} = \sum_{j,k} h_{jk}(c_j^\dagger c_k + c_k^\dagger c_j). \tag{C1}$$
2. Our goal is to implement the unitary transformation
$$U = \prod_{j,k} e^{i\theta_{jk} H_{jk}}, \tag{C2}$$
with θ_{ij} a set of parameters, where we can take the product in an arbitrary order of our choice. This is sufficient to implement the Hamiltonian variational ansatz in VQE, for example.

3. Our goal is to minimise the 2-qubit gate depth required to implement this transformation. We assume that an arbitrary 2-qubit gate has unit cost.

4. We assume that each mode is associated with one or more qubits, such that at least one of these is a "data" qubit; and that to implement each interaction on 2 modes requires at least one 2-qubit gate across all the associated data qubits.

To implement this using the JW transformation, we order the fermionic modes arbitrarily and associate each one with a qubit. We then use the fswap network proposed by Kivlichan et al. [11], which alternates between swapping pairs of adjacent modes starting at even and odd positions. We can combine time-evolution according to the corresponding terms in the Hamiltonian with these fswap operations at no additional cost, as we are assuming that all 2-qubit operations are equivalent. After m layers of fswap operations, all pairs of modes have interacted and the order is returned to the original order, albeit reversed (which can be dealt with by acting on the modes in reverse order in the next step of the algorithm, if desired). Therefore, the algorithm has 2-qubit gate depth m.

To obtain a corresponding lower bound, we observe that in the worst case, there are $\binom{m}{2}$ interactions to be implemented in total. At each step, at most $\lfloor m/2 \rfloor$ interactions can be implemented, because each interaction involves at least one 2-qubit gate across the data qubits. If there are more than m data qubits, this bound continues to hold, as we can select one qubit per mode to produce the set on which we need to implement the interactions. Therefore, in 2-qubit gate depth k we can implement at most $k\lfloor m/2 \rfloor$ interactions. Implementing all the interactions then requires $k \geq m-1$ (if m is even) or $k \geq m$ (if m is odd).

D. Hamiltonian coefficients pipeline

In this Appendix we describe in details the steps required to generate all the hopping matrix (HM) and Coulomb tensor (CT) coefficients for an arbitrary material in the Wannier basis.

1. Full Hamiltonian of an electronic system on a lattice

AWe consider a material consisting of N_{cells} sites arranged in a translationally invariant (Bravais) lattice $\mathcal{G}$. Working within the Born-Oppenheimer approximation [3], we assume that its electronic problem has been restricted to an active space spanned by $N_{\text{orbitals/cell}}$ Wannier functions (WFs), $\mathcal{W}^P_{i,\sigma}(\mathbf{r})$ per cell, with $P \in \mathcal{G}$, $i \in \{1, ..., N_{\text{orbitals/cell}}\}$ labeling the various orbitals, and $\sigma \in \{\uparrow, \downarrow\}$ the spin quantum number. By choosing a coordinate system with origin O, each point $P \in \mathcal{G}$ can be generated by a discrete translation by a vector $\mathbf{R}_P = n^P_1\mathbf{R}_1 + n^P_2\mathbf{R}_2 + n^P_3\mathbf{R}_3 \in \mathcal{B}$, with $\mathbf{R}_a$, $a = 1, 2, 3$, the primitive vector of $\mathcal{G}$ and $\mathcal{B} = \{\mathbf{R} = n_1\mathbf{R}_1 + n_2\mathbf{R}_2 + n_3\mathbf{R}_3 \mid n_a \in \mathbb{Z}, a = 1, 2, 3\}$ the set of all the possible discrete translations on $\mathcal{G}$. Hence, $\mathbf{R}_O = \mathbf{0}$ and $\mathcal{W}^P_{i,\sigma}(\mathbf{r}) = \mathcal{W}_{i,\sigma}(\mathbf{r} - \mathbf{R}_P)$.

The full Hamiltonian for the most general electronic system on the lattice $\mathcal{G}$ is

$$H = H_0 + H_{\text{int}}, \tag{D1}$$

with quadratic and quartic contributions given by

$$H_0 = \sum_{\sigma_1,\sigma_2}\sum_{A,B}\sum_{i,j} t_{(A,i,\sigma_1),(B,j,\sigma_2)} w^\dagger_{A,i,\sigma_1} w_{B,j,\sigma_2}, \tag{D2a}$$

$$H_{\text{int}} = \sum_{\sigma_1,\sigma_2,\sigma_3,\sigma_4}\sum_{A,B,C,D}\sum_{i,j,k,l} V_{(A,i,\sigma_1),(B,j,\sigma_2),(C,k,\sigma_3),(D,l,\sigma_4)} w^\dagger_{A,i,\sigma_1} w^\dagger_{B,j,\sigma_2} w_{C,k,\sigma_3} w_{D,l,\sigma_4}, \tag{D2b}$$

with $\sigma_1, \sigma_2, \sigma_3, \sigma_4 \in \{\downarrow, \uparrow\}$, $A, B, C, D \in \mathcal{G}$, and $i, j, k, l \in \{1, ..., N_{\text{orbital/cell}}\}$. Here, $w^{(\dagger)}_{A,i,\sigma}$ destroys (creates) an electron at site A with mode i and spin σ. Finally, t and V denote the spinful HM and the CT, respectively.

In what follows we will make the following assumptions:

- **Non-magnetic material (NMM) approximation**: we will consider materials without spin-orbit coupling and in the absence of magnetic fields. In this case, the spin up and spin down sectors are degenerate. From here on, we will therefore omit the spin index in the WFs, i.e., $\mathcal{W}^{P}_{i,\sigma}(\mathbf{r}) := \mathcal{W}^{P}_{i}(\mathbf{r})$. Note that this assumption can be relaxed by generalizing Eq. (D3) and Eq. (D6) below.
- **Real Wannier functions**: we will assume that the WFs $\mathcal{W}^{P}_{i,\sigma}(\mathbf{r})$ are real. This is always true if maximally localized Wannier functions can be built (see Appendix B) [19].
- **Nearest-neighbour (NN) approximation:** in calculating the HM and CT coefficients, we will consider the following NN approximation of order n. Thanks to the localization properties of the MLWFs, this is a reasonable assumption for all the materials we considered in the main text. By looking at a lattice site $P \in \mathcal{G}$ we group the other sites of the lattice in ascending order of Euclidean distances from P, namely $0 = d_0 < d_1 < d_2 < ... < d_n$. All the sites of the lattice having the same distance d_n from P represent the nearest neighbours of order n of P. Then, in the NN approximation of order n we compute only those HM (CT) coefficients involving lattice sites A and B (A, B, C, D) which are NN of order $\leq n$ with respect to each other, i.e., such that $|P_1 - P_2| \leq d_n$, $\forall P_1, P_2 \in \{A, B\}$ ($\forall P_1, P_2 \in \{A, B, C, D\}$) and set $t^{AB}_{ij} = 0$ ($V^{ABCD}_{ijkl} = 0$) otherwise. Here, $|...|$ denotes the Euclidean distance between two sites. In particular, we introduce the general notation $\mathcal{N}^{n}_{\mathcal{G}'} = \{\, P \in \mathcal{G} \mid |P - P'| \leq d_n,\ \forall P' \in \mathcal{G}' \,\}$, with $\mathcal{G}' \subseteq \mathcal{G}$, and $N^{n}_{\mathcal{G}'} = \dim(\mathcal{N}^{n}_{\mathcal{G}'})$, to denote the set of sites of $\mathcal{G}$ which are NN of order $\leq n$ with respect to all the sites of $\mathcal{G}'$ and their total number, respectively.

Within these approximations, we have that the coefficients of the spinful HM in Eq. (D2) are defined as

$$t_{(A,i,\sigma_1),(B,j,\sigma_2)} = \begin{cases} T^{AB}_{ij} & \text{if } \sigma_1 = \sigma_2 \\ 0 & \text{otherwise} \end{cases}, \tag{D3}$$

where the *bare*[8] HM coefficients can be computed as

$$T^{AB}_{ij} = \int d\mathbf{r} \mathcal{W}_i(\mathbf{r} - \mathbf{R}_A) h_{\text{sp}} \mathcal{W}_j(\mathbf{r} - \mathbf{R}_B). \tag{D4}$$

Here, $h_{\text{sp}} = -\hbar^2\nabla^2/(2m) + \tilde{U}_{\text{eff}}(\mathbf{r})$ is the single particle Hamiltonian, with $\tilde{U}_{\text{eff}}(\mathbf{r})$ the effective Kohn-Sham potential (implicitly) derived within the Density Functional Theory (DFT), as explained in Supplementary Note 2 F). Thanks to the discrete translational invariance of the lattice, all the HM coefficients can be obtained from the *core* ones, which are defined by setting $A = O$ in the equation above,

$$T^{OB}_{ij} = \int d\mathbf{r} \mathcal{W}_i(\mathbf{r}) h_{\text{sp}} \mathcal{W}_j(\mathbf{r} - \mathbf{R}_B). \tag{D5}$$

The coefficients of the *spinful* CT in Eq. (D2), $V_{(A,i,\sigma_1),(B,j,\sigma_2),(C,k,\sigma_3),(D,l,\sigma_4)}$, are defined in terms of the *bare* CT coefficients, V^{ABCD}_{ijkl}, as

$$V_{(A,i,\sigma_1),(B,j,\sigma_2),(C,k,\sigma_3),(D,l,\sigma_4)} = \begin{cases} V^{s,ABCD}_{ijkl} & \text{if } \sigma_1 = \sigma_2 = \sigma_3 = \sigma_4 \\ \frac{1}{2} V^{ABCD}_{ijkl} & \text{if } \sigma_1 = \sigma_3 \text{ and } \sigma_2 = \sigma_4 \ (\sigma_1 \neq \sigma_2) \\ -\frac{1}{2} V^{BACD}_{jikl} & \text{if } \sigma_1 = \sigma_4 \text{ and } \sigma_2 = \sigma_3 \ (\sigma_1 \neq \sigma_2) \\ 0 & \text{otherwise} \end{cases}, \tag{D6}$$

with $V^{s,ABCD}_{ijkl} = (V^{ABCD}_{ijkl} - V^{BACD}_{jikl})/2$. In turn, the CT coefficients can be obtained as

$$V^{ABCD}_{ijkl} = \frac{1}{2} \int d\mathbf{r} \int d\mathbf{r}' \mathcal{W}_i(\mathbf{r} - \mathbf{R}_A) \mathcal{W}_j(\mathbf{r}' - \mathbf{R}_B) V(|\mathbf{r} - \mathbf{r}'|) \mathcal{W}_k(\mathbf{r}' - \mathbf{R}_C) \mathcal{W}_l(\mathbf{r} - \mathbf{R}_D), \tag{D7}$$

---

[8] In what follows we will omit this specification and we will implicitly assume that all the HM and CT coefficients but the *spinful* ones are *bare* coefficients.

with $V(|\mathbf{r}-\mathbf{r}'|)$ the (screened) Coulomb potential. Note that, by exploiting the discrete translational invariance of the lattice, we can set $A = O$ and get the *core* CT coefficients

$$C_{ijkl}^{OBCD} \equiv V_{ijkl}^{OBCD} = \frac{1}{2}\int d\mathbf{r}\int d\mathbf{r}'\mathcal{W}_i(\mathbf{r})\mathcal{W}_j(\mathbf{r}'-\mathbf{R}_B)V(|\mathbf{r}-\mathbf{r}'|)\mathcal{W}_k(\mathbf{r}'-\mathbf{R}_C)\mathcal{W}_l(\mathbf{r}-\mathbf{R}_D). \quad \text{(D8)}$$

These coefficients can be computed via Monte Carlo (MC) integration and the full CT can then be retrieved from the core coefficients thanks to the lattice translational invariance (see below).

a. Cauchy-Schwarz inequality To reduce the number of CT coefficients to be computed, we will exploit a Cauchy-Schwarz (CS) inequality between the coefficients themselves. This is obtained by re-writing Eq. (D7) as an inner product,

$$V_{ijkl}^{ABCD} = \langle \rho_{il}^{AD}, \rho_{kj}^{CB}\rangle \equiv \frac{1}{2}\int d\mathbf{r}\int d\mathbf{r}' V(|\mathbf{r}-\mathbf{r}'|)\rho_{il}^{AD}(\mathbf{r})\rho_{kj}^{CB}(\mathbf{r}'), \quad \text{(D9)}$$

with $\rho_{ij}^{AB}(\mathbf{r}) \equiv \mathcal{W}_i(\mathbf{r}-\mathbf{R}_A)\mathcal{W}_j(\mathbf{r}-\mathbf{R}_B)$. Since $\tilde{V}(|\mathbf{r}-\mathbf{r}'|)$ is always a positive definite kernel, it can be shown that the inner product we have just introduced is well-defined and that the following CS inequality holds

$$|V_{ijkl}^{ABCD}|^2 \leq V_{iill}^{AADD} V_{kkjj}^{CCBB}. \quad \text{(D10)}$$

In what follows we will show that, assuming that all the coefficients smaller than a given threshold are negligible, this inequality can be exploited to determine a priori which coefficients of the CT need to be computed via MC integration.

b. Symmetry properties of the Coulomb tensor. For the sake of simplicity we now introduce the composite indices λ_i grouping together the site and orbital indices (e.g., $\lambda_1 = (A, i)$). From Eq. (D7), by exploiting the hermiticty of the CT, i.e., $V_{\lambda_1\lambda_2\lambda_3\lambda_4} = V_{\lambda_4\lambda_3\lambda_2\lambda_1}$, the swap symmetry $V_{\lambda_1\lambda_2\lambda_3\lambda_4} = V_{\lambda_2\lambda_1\lambda_4\lambda_3}$, and the reality of the Wannier functions, one can derive the following identities (see also Supplementary Note 2 D 1)

$$V_{\lambda_1\lambda_2\lambda_3\lambda_4} = V_{\lambda_4\lambda_3\lambda_2\lambda_1} = V_{\lambda_2\lambda_1\lambda_4\lambda_3} = V_{\lambda_4\lambda_2\lambda_3\lambda_1} = V_{\lambda_1\lambda_3\lambda_2\lambda_4} = V_{\lambda_3\lambda_4\lambda_1\lambda_2} = V_{\lambda_3\lambda_1\lambda_4\lambda_2} = V_{\lambda_2\lambda_4\lambda_1\lambda_3}, \quad \text{(D11)}$$

which can be exploited to further reduce the number of CT coefficients we have to compute.

2. Motif Hamiltonian

As discussed in Supplementary Note 6 B 1 of the main text, the real-space localisation of the WFs allows us to focus on the Hamiltonian of small portion of the material consisting of a central cell and its NNs up to a given order n, which we refer to as a *motif* of order n. The whole system (and its full Hamiltonian) can then be obtained via the tiling procedure described in Supplementary Note 5 B. Note that, thanks to the discrete translational invariance of the lattice, we need to include in the motif Hamiltonian only those HM and CT coefficients involving the central unit cell at least once. That is, onsite interactions, and inter- and intra-cell hoppings involving exclusively cells different from the central ones are excluded. The motif Hamiltonian is hence given by $H^m = H_0^m + H_{\text{int}}^m$, with

$$H_0^m = \sum_{\sigma_1,\sigma_2}\sum_{B}\sum_{i,j}\left[t^m_{(O,i,\sigma_1),(B,j,\sigma_2)} w^\dagger_{O,i,\sigma_1} w_{B,j,\sigma_2} + t^m_{(B,j,\sigma_2),(O,i,\sigma_1)} w^\dagger_{B,j,\sigma_2} w_{O,i,\sigma_1}\right], \quad \text{(D12a)}$$

$$\begin{aligned} H_{\text{int}}^m = \sum_{\sigma_1,\sigma_2,\sigma_3,\sigma_4}\sum_{B,C,D}\sum_{i,j,k,l}\Big[& V^m_{(O,i,\sigma_1),(B,j,\sigma_2),(C,k,\sigma_3),(D,l,\sigma_4)} w^\dagger_{O,i,\sigma_1} w^\dagger_{B,j,\sigma_2} w_{C,k,\sigma_3} w_{D,l,\sigma_4} \\ & + V^m_{(B,j,\sigma_2),(O,i,\sigma_1),(C,k,\sigma_3),(D,l,\sigma_4)} w^\dagger_{B,j,\sigma_2} w^\dagger_{O,i,\sigma_1} w_{C,k,\sigma_3} w_{D,l,\sigma_4} \\ & + V^m_{(B,j,\sigma_2),(C,k,\sigma_3),(O,i,\sigma_1),(D,l,\sigma_4)} w^\dagger_{B,j,\sigma_2} w^\dagger_{C,k,\sigma_3} w_{O,i,\sigma_1} w_{D,l,\sigma_4} \\ & + V^m_{(B,j,\sigma_2),(C,k,\sigma_3),(D,l,\sigma_4),(O,i,\sigma_1)} w^\dagger_{B,j,\sigma_2} w^\dagger_{C,k,\sigma_3} w_{D,l,\sigma_4} w_{O,i,\sigma_1}\Big], \end{aligned} \quad \text{(D12b)}$$

with $B, C, D \in \mathcal{N}^{n}_{\{O\}}$. Here, $t^{m}_{(A,i,\sigma_1),(B,j,\sigma_2)}$ and $V^{m}_{(A,i,\sigma_1),(B,j,\sigma_2),(C,k,\sigma_3),(D,l,\sigma_4)}$ are defined as in Eq. (D3) and Eq. (D6), respectively, with $A, B, C, D \in \mathcal{N}^{n}_{\{O,A,B,C,D\}}$.

In what follows, we will illustrate the various stages of the calculation of the HM and CT of the motif Hamiltonian within the real Wannier functions, NMM, and order n NN approximations described in Appendix D 1.

a. *Hopping matrix coefficients*

a. Stage H1: core HM coefficients. In the first stage, the *core* HM coefficients T^{OB}_{ij}, with $B \in \mathcal{G}'$, introduced in Eq. (D5) are read from the output of DFT/Wannier90 calculations. Here, $\mathcal{G}'$ is determined by size of the lattice considered in the DFT simulation.

b. Stage H2: filtered core HM coefficients. In the second stage, the core HM coefficients T^{OB}_{ij} are restricted to NNs of order n_0, i.e., for lattice sites $B \in \mathcal{N}^{n_0}_{\{O\}}$. To determine n_0, we compare the exact bands $\varepsilon_i(\mathbf{k})$ with the ones obtained from the *filtered* order n NN HM, $\overline{\varepsilon}^{(n_0)}_i(\mathbf{k})$. The latter are given by the eigenvalues of the matrices

$$h(\mathbf{k})_{ij} = \sum_{\mathbf{B}\in\mathcal{N}^{n_0}_{\mathbf{0}}} e^{i\mathbf{k}\cdot\mathbf{R}} \overline{T}^{OB}_{ij}, \tag{D13}$$

with filtered HM coefficients

$$\overline{T}^{OB}_{ij} = \begin{cases} T^{OB}_{ij} & \text{if } |T^{OB}_{ij}| \geq t_0 \\ 0 & \text{otherwise,} \end{cases} \tag{D14}$$

and threshold $t_0 = \max|T^{OB}_{mn}|$, $\forall m, n$ and $\forall B \notin \mathcal{N}^{n_0}_{\{O\}}$ (i.e., t_0 is given by the largest absolute value of the HM coefficients which are NN of order $> n_0$ with respect to O). In what follows, an overlying bar will always be used to denote filtered quantities. To make the comparison quantitative, we measure the distance between bands as [89]

$$\mathcal{D}(n_0) = \max_{i,\mathbf{k}} \left|\varepsilon_i(\mathbf{k}) - \overline{\varepsilon}^{(n_0)}_i(\mathbf{k})\right| \tag{D15}$$

and we determine n_0 as the minimum integer such that $\mathcal{D}(n_0) \leq \eta_\varepsilon$, with η_ε a pre-determined threshold (for instance, in the main text we set $\eta_\varepsilon = 0.5$ eV), for values of $\mathbf{k}$ sampled from a regular grid in the Brillouin zone.

c. Stage H3: motif HM coefficients. In the third stage, the filtered *motif* HM coefficients $\overline{T}^{OB}_{ij}$ and $\overline{T}^{BO}_{ij}$ with $B \in \mathcal{N}^{n_0}_{\{O\}}$ [as defined in Eq. (D12a)] are retrieved via

$$\overline{T}^{BO}_{ij} = \overline{T}^{OB}_{ji}. \tag{D16}$$

d. Stage H4: spinful motif HM coefficients. In the fourth stage, the *spinful* motif HM coefficients $\tilde{t}_{(A,i,\sigma_1),(B,j,\sigma_2)}$ with $A, B \in \mathcal{N}^{n_0}_{\{O\}}$are obtained via Eq. (D3),

$$t_{(A,i,\sigma_1),(B,j,\sigma_2)} = \begin{cases} \overline{T}^{AB}_{ij} & \text{if } \sigma_1 = \sigma_2 \\ 0 & \text{otherwise} \end{cases}. \tag{D17}$$

e. (optional) Stage H4b: spinful lattice HM coefficients. In this optional stage, all the filtered spinful HM coefficients over the lattice $\mathcal{G}$ are obtained from Eq. (D17) by exploiting the discrete translational invariance:

$$t_{(A',i,\sigma_1),(B',j,\sigma_2)} = t_{(O,i,\sigma_1),(B,j,\sigma_2)} \quad \text{with } P' = P + \mathbf{R},\ \forall \mathbf{R} \in \mathcal{B}. \tag{D18}$$

b. Coulomb tensor coefficients

Here we describe the steps to compute the Coulomb integrals defined in Eq. (D7) via MC integration for a motif of order n_{int}. Since these are 6−dimensional integrals, their evaluation is costly. It is therefore essential to evaluate as little integrals as possible. To do so, we will take advantage of both the CS inequality introduced in Eq. (D10) and the symmetry properties of the CT of Eq. (D11).

a. Stage CT1: fundamental CT coefficients. In the first stage, we compute via MC integration the *fundamental* terms required to apply the CS inequality of Eq. (D10), denoted by F_{iill}^{OODD}. These are defined as

$$F_{iill}^{OODD} = \begin{cases} V_{iill}^{OOOO} & \forall i, l \geq i \\ V_{iill}^{OODD} & \forall i, l \text{ and } \forall C \in \mathcal{N}_{\{O\}}^{n_{\mathrm{int}}} \setminus \{O\} \end{cases} . \tag{D19}$$

To reduce the number of Coulomb integrals to be computed it is reasonable to assume that the CT coefficients whose absolute value is smaller than a pre-determined threshold do not contribute to the properties of the material. Importantly, due to Eq. (D10), the CT coefficient with the largest absolute value will be one of the fundamental coefficients above. Hence, we can fix a threshold as $t_{\mathrm{int}} = \tau_{\mathrm{int}} \times \max(|F_{iill}^{OODD}|)$ with, e.g., $\tau_{\mathrm{int}} \sim 10^{-2}$, and define the *filtered* fundamental CT coefficients as

$$\overline{F}_{i}^{OODD} = \begin{cases} F_{iill}^{OODD} & \text{if } |F_{ill}^{OODD}| \geq t_{\mathrm{int}} \\ 0 & \text{otherwise} \end{cases} . \tag{D20}$$

b. Stage CT2: filtered unique CT coefficients. In the second stage, we exploit Eq. (D10) to compute via MC integration only those *unique* CT coefficients, defined as $U_{ijkl}^{OBCD} = V_{ijkl}^{OBCD}$, $\forall (O, B, C, D) \in \mathcal{C}^{OBCD}$ and $\forall (i, j, k, l) \in \mathcal{S}^{OBCD}$, such that $\overline{F}_{iill}^{OODD} \overline{F}_{kkjj}^{OO(B-C)(B-C)} \geq t_{\mathrm{int}}$. Here, $\mathcal{C}^{OBCD}$ and $\mathcal{S}^{OBCD}$ denote the minimal set of lattice sites and the unique orbital configurations required in the evaluation of terms with site structure (O, B, C, D), respectively. In particular, the latter is defined as the quotient set of all the possible orbital configurations $\mathcal{M}$ induced by the equivalence relation R^{OBCD}, i.e., $\mathcal{S}^{OBCD} = \mathcal{M}/R^{OBCD} = \{ [(i, j, k, l)]^{OBCD} \mid (i, j, k, l) \in \mathcal{M} \}$, with $[(i, j, k, l)]^{OBCD}$ the equivalence class associated with the orbital configuration (i, j, k, l) with respect to R^{OBCD}. In turn, for each lattice site configuration (O, B, C, D), R^{OBCD} is determined by the symmetry properties of the CT outlined in Eq. (D11). For example, looking at the site structure (O, O, O, O), the configurations

$$(i, j, k, l), (l, k, j, i), (j, i, l, k), (l, j, k, i), (i, k, j, l), (k, l, i, j), (k, i, l, j), (j, l, i, k),$$

$\forall i, j, k, l \in \{1, ..., N_{\mathrm{orbitals/cell}}\}$, are equivalent according to R^{OOOO} since $V_{ijkl}^{OOOO} = V_{lkji}^{OOOO} = ... = V_{jlik}^{OOOO}$. Hence,

$$[(i, j, l, k)]^{OOOO} = \{ (i, j, k, l), (l, k, j, i), (j, i, l, k), (l, j, k, i), (i, k, j, l), (k, l, i, j), (k, i, l, j), (j, l, i, k) \}$$

represents the equivalence class of a given orbital configuration $(i, j, k, l) \in \mathcal{M}$. On the other hand, if we consider the site structure (O, B, B, O), only the configurations

$$(i, j, k, l), (l, j, k, i), (i, k, j, l), (l, k, j, i),$$

$\forall i, j, k, l \in \{1, ..., N_{\mathrm{orbitals/cell}}\}$, are equivalent according to R^{OBBO}. Indeed, for instance, $V_{ijkl}^{OBBO} \neq V_{jilk}^{BOOB}$ and, therefore, in contrast with the previous case, (i, j, k, l) and (j, i, l, k) are not equivalent. For the site structure (O, B, B, O), the equivalence class of a given orbital configuration $(i, j, k, l) \in \mathcal{M}$ is therefore

$$[(i, j, k, l)]^{OOBB} = \{ (i, j, k, l), (l, j, k, i), (i, k, j, l), (l, k, j, i) \} .$$

Note that, in this case, in order to retrieve all the possible CT coefficients (i.e., with $B \in \mathcal{N}_{\{O\}}^{n_{\mathrm{int}}}$ and $\forall (i, j, k, l) \in \mathcal{M}$) we need to compute the CT coefficients for the following minimal set of lattice site configurations

$$\mathcal{C}^{OBBO} = \{ (O, B, B, O) \mid B \in \mathcal{N}_{\{O\}}^{n}, B \neq O \} .$$

Following similar steps, it is possible to determine the orbital equivalence classes $[(i,j,k,l)]^{OBCD}$ and the minimal set of required lattice site configurations $\mathcal{C}^{OBCD}$ for an arbitrary lattice site structure (O,B,C,D). In particular, we have

- $\mathcal{C}^{OOOO} = \{\,(O,O,O,O)\,\}$ and

$$[(i,j,k,l)]^{OOOO} = \{\,(i,j,k,l),(l,k,j,i),(j,i,l,k),(l,j,k,i),(i,k,j,l),(k,l,i,j),(k,i,l,j),(j,l,i,k)\,\}\,.$$

- $\mathcal{C}^{OBBO} = \{\,(O,B,B,O) \mid B \in \mathcal{N}^n_{\{O\}}, B \neq O\,\}$ and

$$[(i,j,k,l)]^{OBBO} = \{\,(i,j,k,l),(l,j,k,i),(i,k,j,l),(l,k,j,i)\,\}\,.$$

- $\mathcal{C}^{OOBB} = \{\,(O,O,B,B) \mid B \in \mathcal{N}^n_{\{O\}}, B \neq O\,\}$ and

$$[(i,j,k,l)]^{OOBB} = \{\,(i,j,k,l),(j,i,l,k)\,\}\,.$$

- $\mathcal{C}^{OOOB} = \{\,(O,O,O,B) \mid B \in \mathcal{N}^n_{\{O\}}, B \neq O\,\}$ and

$$[(i,j,k,l)]^{OOOB} = \{\,(i,j,k,l),(i,k,j,l)\,\}\,.$$

- $\mathcal{C}^{OBCO} = \{\,(O,B,C,O) \mid B,C \in \mathcal{N}^n_{\{O,B,C\}}, B \neq O, C > B\,\}$ and

$$[(i,j,k,l)]^{OBCO} = \{\,(i,j,k,l),(l,j,k,i)\,\}\,.$$

  Note that coefficients with $C < B$ can be retrieved via $V^{OBCO}_{ijkl} = V^{OCBO}_{ikjl}$.

- $\mathcal{C}^{OOBC} = \{\,(O,O,B,C) \mid B,C \in \mathcal{N}^n_{\{O,B,C\}}, B \neq O, C > B\,\}$ and

$$[(i,j,k,l)]^{OOBC} = \{\,(i,j,k,l)\,\}\,, \quad \text{i.e., } \mathcal{S}^{OOBC} = \mathcal{M}.$$

  Note that coefficients with $C < B$ can be retrieved via $V^{OOBC}_{ijkl} = V^{OOCB}_{jilk}$.

- $\mathcal{C}^{OBBC} = \{\,(O,B,B,C) \mid B,C \in \mathcal{N}^n_{\{O,B,C\}}, B \neq O, C \notin \{\,O,B\,\}\,\}$ and

$$[(i,j,k,l)]^{OBBC} = \{\,(i,j,k,l),(i,k,j,l)\,\}\,.$$

- $\mathcal{C}^{OBCB} = \{\,(O,B,C,B) \mid B,C \in \mathcal{N}^n_{\{O,B,C\}}, B \neq O, C \notin \{\,O,B\,\}\,\}$ and

$$[(i,j,k,l)]^{OBCB} = \{\,(i,j,k,l)\,\}\,, \quad \text{i.e., } \mathcal{S}^{OBCB} = \mathcal{M}.$$

- $\mathcal{C}^{OBCD} = \{\,(O,B,C,D) \mid B,C,D \in \mathcal{N}^n_{\{O,B,C,D\}}, B \neq O, C > B, D \notin \{\,O,B,C\,\}\,\}$ and

$$[(i,j,k,l)]^{OBCD} = \{\,(i,j,k,l)\,\}\,, \quad \text{i.e., } \mathcal{S}^{OBCD} = \mathcal{M}.$$

  Note that coefficients with $C < B$ can be retrieved via $V^{OBCD}_{ijkl} = V^{OCBD}_{ikjl}$.

It can be shown that the choices above allow us to minimize the number of CT coefficients to be computed. Finally, the *filtered* unique coefficients are introduced as

$$\tilde{U}^{OBCD}_i = \begin{cases} \overline{U}^{OBCD}_{ill} & \text{if } |U^{OBCD}_{ill}| \geq t_{\text{int}} \\ 0 & \text{otherwise} \end{cases}\,. \tag{D21}$$

c. Stage CT3: filtered motif CT coefficients. In the third stage, the filtered *motif* CT coefficients entering Eq. (D12), $V_{ijkl}^{m,ABCD}$, $\forall A,B,C,D \in \mathcal{N}^n_{\{O,A,B,C,D\}}$ and $\forall (i,j,k,l) \in \mathcal{M}$, are obtained from the filtered unique ones. First, by exploiting the equivalence classes introduced in the previous stage, we can obtain the coefficients $V_{ijkl}^{m,OBCD}$, $\forall (O,B,C,D) \in \mathcal{C}^{OBCD}$ and $\forall (i,j,k,l) \in \mathcal{M}$. The latter can then be extended to any $A,B,C,D \in \mathcal{N}^n_{\{O,A,B,C,D\}}$ (and at least one among A,B,C,D equals to O) via the following identities

- $V_{ijkl}^{OBBO} = V_{jilk}^{BOOB}$,
- $V_{ijkl}^{OOBB} = V_{ikjl}^{OBOB} = V_{ljki}^{BOBO} = V_{lkji}^{BBOO}$,
- $V_{ijkl}^{OOOB} = V_{jilk}^{OOBO} = V_{jlik}^{OBOO} = V_{ljki}^{BOOO}$,
- $V_{ijkl}^{OBCO} = V_{jilk}^{BOOC}$,
- $V_{ijkl}^{OOBC} = V_{ikjl}^{OBOC} = V_{lkji}^{CBOO} = V_{ljkl}^{COBO}$,
- $V_{ijkl}^{OBBC} = V_{ljki}^{CBBO} = V_{jilk}^{BOCB} = V_{jlik}^{BCOB}$,
- $V_{ijkl}^{OBCB} = V_{ljki}^{BBCO} = V_{ikjl}^{OCBB} = V_{lkji}^{BCBO} = V_{jilk}^{BOBC} = V_{jlik}^{BBOC} = V_{kilj}^{COBB} = V_{klij}^{CBOB}$,
- $V_{ijkl}^{OBCD} = V_{jlik}^{BDOC} = V_{jilk}^{BODC} = V_{ljki}^{DBCO}$.

d. Stage CT4: spinful motif CT coefficients. The filtered *spinful* motif CT coefficients of Eq. (D12b), $V^m_{(A,i,\sigma_1),(B,j,\sigma_2),(C,k,\sigma_3),(D,l,\sigma_4)}$ with $A,B,C,D \in \mathcal{N}^n_{\{O,A,B,C,D\}}$ and $(i,j,k,l) \in \mathcal{M}$, can be obtained via Eq. (D6) as

$$V^m_{(A,i,\sigma_1),(B,j,\sigma_2),(C,k,\sigma_3),(D,l,\sigma_4)} = \begin{cases} V_{ijkl}^{m,s,ABCD} & \text{if } \sigma_1=\sigma_2=\sigma_3=\sigma_4 \\ \frac{1}{2}V_{ijkl}^{m,ABCD} & \text{if } \sigma_1=\sigma_3 \text{ and } \sigma_2=\sigma_4 \ (\sigma_1\neq\sigma_2) \\ -\frac{1}{2}V_{jikl}^{m,BACD} & \text{if } \sigma_1=\sigma_4 \text{ and } \sigma_2=\sigma_3 \ (\sigma_1\neq\sigma_2) \\ 0 & \text{otherwise} \end{cases}, \tag{D22}$$

with $V_{ijkl}^{m,s,ABCD} = (V_{ijkl}^{m,ABCD} - V_{jikl}^{m,BACD})/2$.

e. (optional) Stage CT4b: spinful lattice CT coefficients. In this optional stage, all the filtered spinful coefficients over the lattice $\mathcal{G}$ are obtained from motif coefficients by exploiting the discrete translational invariance of the lattice:

$$V_{(A',i,\sigma_1),(B',j,\sigma_2),(C',k,\sigma_3),(D',l,\sigma_4)} = V^m_{(A,i,\sigma_1),(B,j,\sigma_2),(C,k,\sigma_3),(D,l,\sigma_4)}, \tag{D23}$$

with $P' = P + \mathbf{R}$, $\forall P \in \mathcal{N}^n_{\{O,A,B,C,D\}}$ and $\forall \mathbf{R} \in \mathcal{B}$.

f. Stage CT5: consistently filtered CT coefficients. In general, the NN approximation of order n_{int} we performed in the previous stages is not guaranteed to be consistent, i.e., there may be CT coefficients corresponding to a higher order NN approximation whose absolute value is actually larger than the threshold t_{int}. To avoid such a situation and obtain a consistent approximation of the CT, in this fifth stage we can

- repeat the procedure above for $n_{\text{int}}+1$ using the same value of t_{int};
- set a new threshold t'_{int} as the largest of the absolute values of the coefficients of the CT obtained within a NN approximation of order $n_{\text{int}}+1$ which are not contained in the CT resulting from the NN approximation of order n_{int}.
- filter the CT obtained within the NN approximation of order n_{int} with the new threshold t'_{int}.

Note that, in principle, to be sure that the approximation of the CT is consistent, one should repeat the procedure above for all NN orders $> n_{\text{int}}$. However, taking into account the localization of the MLWFs and due the growing computational cost, in this work we limit our analysis to the case with $n_{\text{int}}+1$ only.

Material	Spacegroup	Bands	Dimension	Size	Modes	Qubits
GaAs	F$\bar{4}$3m	4	5x5x5	125	1000	1120
H_3S	Im$\bar{3}$m	7	5x5x5	125	1750	1870
Li_2CuO_2	P1	11	5x3x3	45	990	1024
Si	Fd3m	4	5x5x5	125	1000	1120
$SrVO_3$	Pm$\bar{3}$m	3	3x3x3	27	162	180

Supplementary Table 9. Material properties.

c. *Single-index HM and CT coefficients and Majorana Hamiltonian*

To obtained the Majorana form of the motif Hamiltonian, we first map each composite site-mode-spin indices triplet (A_i, i, σ_i) to a single index $\alpha_i \in \{1, ..., M\}$, with $M = 2N_{\text{orbitals/cell}}N_{\text{cells/motif}}$ the total number of complex fermion modes. Here, $N_{\text{cells/motif}}$ is determined as $N_{\text{cells/motif}} = \max(N_O^{n_0}, N_O^{n_{\text{int}}})$ with $N_O^{n_0}$ and $N_O^{n_{\text{int}}}$ the number of cells forming the motives used in the calculation of the HM and CT coefficients, respectively. If $N_O^{n_0} > N_O^{n_{\text{int}}}$ ($N_O^{n_0} < N_O^{n_{\text{int}}}$) all the CT coefficients with single indices corresponding to sites of $\mathcal{N}_O^{n_0}$ ($\mathcal{N}_O^{n_{\text{int}}}$) not included in $\mathcal{N}_O^{n_{\text{int}}}$ ($\mathcal{N}_O^{n_0}$) are set to zero. In terms of the single indices, the quadratic and quartic part of the motif Hamiltonian become

$$H_0 = \sum_{\alpha,\beta} t_{\alpha\beta} w_\alpha^\dagger w_\beta, \tag{D24a}$$

$$H_{\text{int}} = \sum_{\alpha,\beta,\gamma,\delta} V_{\alpha\beta\gamma\delta} w_\alpha^\dagger w_\beta^\dagger w_\gamma w_\delta, \tag{D24b}$$

respectively.

As discussed in Supplementary Note 3 B, the Majorana basis operators are defined by $w_\alpha = (\gamma_\alpha + i\bar{\gamma}_\alpha)/2$ and $w_\alpha^\dagger = (\gamma_\alpha - i\bar{\gamma}_\alpha)/2$. In terms of the latter, the motif Hamiltonian reads

$$H_M = \sum_{k\in\{0,1\}^{2M}} \alpha_k \prod_j \gamma_j^{k_{2j}} \bar{\gamma}_j^{k_{2j+1}} \ , \ |k| \in \{2, 4\}. \tag{D25}$$

E. Materials analysis

1. Material properties

In Supplementary Table 9 we list the properties of the materials studied throughout Supplementary Note 6.

2. Circuit depth

Here we present the complete data relating to the summary presented in Supplementary Table 7. In particular, while that overview shows the circuit depths for each material by compiling the circuit including an fswap network for VQE, here we compile with and without fswap networks for both VQE and TDS. In Supplementary Table 10, we report the circuit depth split between the onsite and nearest neighbour (Onsite/NN) terms, separately from the depth for the implementation of terms involving next nearest neighbours and beyond (NNN+). In Supplementary Table 11 we list the number of terms within the corresponding Hamiltonians which are described as either onsite/NN or NNN+.

Here we treat the onsite/NN terms differently from the NNN+ terms, on the basis that the former are *translatable* while the latter are *nonstranslatable*. Translatable terms may be tiled according to

the strategy outlined in Supplementary Note 5 B: e.g. we first evaluate the depth of a group of terms which involve only the central cell; then we assume that equivalent terms from neighbouring cells can be implemented simultaneously, i.e. in parallel to the those terms from the central cell. As such, the depth of the onsite/NN terms is deemed to represent the depth of implementing all the translatable terms. In principle, translatable terms may be tiled indefinitely to achieve any desired lattice size, i.e. in order to simulate a given material in bulk, we need only increase the number of qubits, but the circuit depth will remain constant. This allows us to evaluate the circuit depth corresponding to onsite/NN terms using only a representative set of *fundamental* terms: any term which is a translation of a fundamental term is omitted for the purpose of costing (but must be restored in parallel when actual circuits are built, such that the Hamiltonian is faithfully executed). This is not true in the case of nontranslatable terms, so we must fix lattice sizes in order to retrieve meaningful circuit costs – these lattice sizes are listed in Supplementary Table 9. To effectively cost the NNN+ terms, we must explicitly include all interactions extending beyond next-nearest-neighbour cells, e.g. for a sample of $SrVO_3$ with lattice dimensions $3 \times 3 \times 3$, where each lattice point corresponds to a cell with six modes, there are 288 NNN+ terms in the Hamiltonian, while there are 3159 onsite/NN terms. We see that in most cases the depth is dominated by the long range NNN+ terms, owing to the inclusion of many terms when the lattice of the fermionic encoding is tiled.

The compiler terminates by producing instructions for a quantum circuit: the instructions consist of circuit layers, where each layer is either an interaction layer or fswap layer. Interaction layers are a set of Hamiltonian terms which the compiler has determined may be implemented in parallel, according to the decomposition rules of the given algorithm. The interaction layers implement all quadratic or quartic Hamiltonian terms in the circuit. Fswap layers are then a set of mode-pairs, indicating which modes should be interchanged in order to facilitate subsequent interaction layers, implemented via Eq. (100).

The compiler does *not* account for hardware constraints, such as available gates or qubit connectivity. The final stage is then to iterate over the circuit layers and ensure that, as well as simultaneous with respect to the compiler's strategy, they are simultaneous on the target hardware. In cases where terms within a layer clash, they are placed on separate sublayers – this is achieved using graph coloring, where terms are encoded as nodes and incompatibilities are encoded as edges. This mechanism is versatile and allows for arbitrary compatability rules to represent various hardware, e.g. devices where ISWAP gates are available facilitate cheaper fswaps than those without ISWAP. The default rule is simply that terms acting on any overlapping qubits must be in separate sublayers.

3. Measurement layers

In Supplementary Table 12 we provide the data used in the analysis in Supplementary Note 4 D. In particular, the measurement layers presented in Supplementary Figure 37 are repeated in Supplementary Table 12 for ease of interpretation.

Material	Dimension	Modes	Qubits	Algorithm	Onsite/NN	NNN+	Total
GaAs	$5 \times 5 \times 5$	1000	1120	TDS	664	9305	9969
				TDS*	1090	9305	10395
				VQE	662	7191	7853
				VQE*	1072	7191	8263
H_3S	$5 \times 5 \times 5$	1750	1870	TDS	1217	38228	39445
				TDS*	7024	38228	45252
				VQE	1214	36126	37340
				VQE*	7000	36126	43126
Li_2CuO_2	$5 \times 3 \times 3$	990	1024	TDS	1675	6885	8560
				TDS*	3385	6885	10270
				VQE	1667	6710	8377
				VQE*	3341	6710	10051
Si	$5 \times 5 \times 5$	1000	1120	TDS	706	8904	9610
				TDS*	1746	8904	10650
				VQE	704	7857	8561
				VQE*	1733	7857	9590
$SrVO_3$	$3 \times 3 \times 3$	162	180	TDS	408	700	1108
				TDS*	552	700	1252
				VQE	408	476	884
				VQE*	555	476	1031

Supplementary Table 10. Resource requirements for each of the examined materials for a number of compiled algorithms. Algorithms denoted without an asterisk are compiled with the use of an fswap network, while those with an asterisk omit the fswap network. Properties of the listed materials are given in Supplementary Table 9, and the number of terms of each Hamiltonian type are listed in Supplementary Table 11.

Material	Onsite/NN	NNN+	Total
GaAs	27500	22080	49580
H_3S	52925	46376	99301
Li_2CuO_2	13953	3344	17297
Si	30300	22880	53180
$SrVO_3$	3159	288	3447

Supplementary Table 11. Number of terms in the materials' Hamiltonians, listed by whether they involve at most one nearest neighbour (onsite/NN) or extend to next nearest neighbour and beyond (NNN+).

	Strategy	Z	Onsite/NN	NNN+	Total
GaAs	Commutatitvity	1	15	22	38
	Noncrossing	1	56	475	532
	Qubitwise Commutativity	1	85	475	561
H_3S	Commutatitvity	1	36	54	91
	Noncrossing	1	161	2274	2436
	Qubitwise Commutativity	1	463	2274	2738
Li_2CuO_2	Commutatitvity	1	22	19	42
	Noncrossing	1	88	539	628
	Qubitwise Commutativity	1	248	539	788
Si	Commutatitvity	1	21	26	48
	Noncrossing	1	84	510	595
	Qubitwise Commutativity	1	142	510	653
$SrVO_3$	Commutatitvity	1	6	4	11
	Noncrossing	1	36	46	83
	Qubitwise Commutativity	1	41	46	88

Supplementary Table 12. Measurement layers required, corresponding to those represented in Supplementary Figure 37. Number of measurement layers are reported by the type of terms involved in the layer. Terms involving only single qubit Z measurements are extracted to a first measurement layer in each case; then terms are grouped by whether they are onsite/NN or NNN+, and the given strategies applied.

Supplementary References

[1] F. Jensen, *Introduction to computational chemistry* (John Wiley & Sons, Chichester, 2017).

[2] D. Wecker, M. B. Hastings, and M. Troyer, "Progress towards practical quantum variational algorithms", Phys. Rev. A **92**, 042303 (2015).

[3] L. S. Cederbaum, "Born-Oppenheimer approximation and beyond", in *Conical intersections. electronic structure, dynamics and spectroscopy*, edited by W. Domcke, D. R. Yarkony, and H. Köppel (World Scientific, Singapore, 2004).

[4] J. Sólyom, *Fundamentals of the physics of solids*, Vol. 2 (Springer-Verlag, Berlin, 2009).

[5] N. W. Ashcroft and N. D. Mermin, *Solid state physics* (Saunders College Publishing, Philadelphia, 1976).

[6] A. Georges, "Strongly correlated electron materials: dynamical mean-field theory and electronic structure", AIP Conf. Proc **715**, 3–74 (2004).

[7] K. Haule, "Exact double counting in combining the dynamical mean field theory and the density functional theory", Phys. Rev. Lett. **115**, 196403 (2015).

[8] M. Imada and T. Miyake, "Electronic structure calculation by first principles for strongly correlated electron systems", J. Phys. Soc. Japan **79**, 112001 (2010).

[9] S. Kanno, S. Endo, T. Utsumi, and T. Tada, "Resource estimations for the Hamiltonian simulation in correlated electron materials", Phys. Rev. A **106**, 012612 (2022).

[10] C. Derby, J. Klassen, J. Bausch, and T. Cubitt, "Compact fermion to qubit mappings", Phys. Rev. B **104**, 035118 (2021).

[11] I. D. Kivlichan, J. McClean, N. Wiebe, C. Gidney, A. Aspuru-Guzik, G. K.-L. Chan, and R. Babbush, "Quantum simulation of electronic structure with linear depth and connectivity", Phys. Rev. Lett. **120**, 110501 (2018).

[12] L. Boeri et al., "The 2021 room-temperature superconductivity roadmap", Journal of Physics: Condensed Matter (2021).

[13] M. Park, X. Zhang, M. Chung, G. B. Less, and A. M. Sastry, "A review of conduction phenomena in Li-ion batteries", Journal of Power Sources **195**, 7904–7929 (2010).

[14] J. Sólyom, *Fundamentals of the physics of solids*, Vol. 1 (Springer-Verlag, Berlin, 2007).

[15] N. Marzari, A. A. Mostofi, J. R. Yates, I. Souza, and D. Vanderbilt, "Maximally localized Wannier functions: theory and applications", Rev. Mod. Phys. **84**, 1419–1475 (2012).

[16] J. D. Cloizeaux, "Energy bands and projection operators in a crystal: analytic and asymptotic properties", Phys. Rev. **135**, A685–A697 (1964).

[17] J. D. Cloizeaux, "Analytical properties of n-dimensional energy bands and Wannier functions", Phys. Rev. **135**, A698–A707 (1964).

[18] G. Nenciu, "Existence of the exponentially localised Wannier functions", Commun. Math. Phys **91**, 10.1007/BF01206052 (1983).

[19] C. Brouder, G. Panati, M. Calandra, C. Mourougane, and N. Marzari, "Exponential localization of Wannier functions in insulators", Phys. Rev. Lett. **98**, 046402 (2007).

[20] C. Derby and J. Klassen, *A compact fermion to qubit mapping part 2: alternative lattice geometries*, 2021, arXiv:2101.10735 [quant-ph].

[21] M. Hamermesh, *Group theory and its application to physical problems* (Dover Publication, Inc., Mineola, N.Y., 2003).

[22] E. Clementi and D. L. Raimondi, "Atomic screening constants from SCF functions", J. Chem. Phys. **38**, 2686–2689 (1963).

[23] R. O. Jones and O. Gunnarsson, "The density functional formalism, its applications and prospects", Rev. Mod. Phys. **61**, 689–746 (1989).

[24] R. M. Martin, *Electronic structure. basic theory and practical methods* (Cambridge University Press, Cambridge, 2020).

[25] J. Hafner, C. Wolverton, and G. Ceder, "Toward computational materials design: the impact of density functional theory on materials research", MRS Bulletin **31**, 659–668 (2006).

[26] N. Marzari, A. Ferretti, and C. Wolverton, "Electronic-structure methods for materials design", Nat. Mater. **20**, 736–749 (2021).

[27] P. Hohenberg and W. Kohn, "Inhomogeneous electron gas", Phys. Rev. **136**, B864–B871 (1964).

[28] W. Kohn and L. J. Sham, "Self-consistent equations including exchange and correlation effects", Phys. Rev. **140**, A1133–A1138 (1965).

[29] P. Giannozzi et al., "QUANTUM ESPRESSO: a modular and open-source software project for quantum simulations of materials", J. Phys. Condens. Matter **21**, 395502 (2009).

[30] P Giannozzi et al., "Advanced capabilities for materials modelling with quantum ESPRESSO", J. Phys. Condens. Matter **29**, 465901 (2017).

[31] G. Pizzi et al., "Wannier90 as a community code: new features and applications", J. Phys. Condens. Matter **32**, 165902 (2020).

[32] K. Lejaeghere et al., "Reproducibility in density functional theory calculations of solids", Science **351**, aad3000 (2016).

[33] D. R. Hamann, "Optimized norm-conserving Vanderbilt pseudopotentials", Phys. Rev. B **88**, 085117 (2013).

[34] BIOVIA Materials Studio, *Plane wave basis set*, `https://www.tcm.phy.cam.ac.uk/castep/documentation/WebHelp/content/modules/castep/thcastepplanebasis.htm` (2020).

[35] E. Pavarini, "Solving the strong-correlation problem in materials", Riv. Nuovo Cimento **44**, 597–640 (2021).

[36] G. Kotliar, S. Y. Savrasov, K. Haule, V. S. Oudovenko, O. Parcollet, and C. A. Marianetti, "Electronic structure calculations with dynamical mean-field theory", Rev. Mod. Phys. **78**, 865–951 (2006).

[37] G. Knizia and G. K.-L. Chan, "Density matrix embedding: a simple alternative to dynamical mean-field theory", Phys. Rev. Lett. **109**, 186404 (2012).

[38] M. Nusspickel and G. H. Booth, *Systematic improvability in quantum embedding for real materials*, 2022, arXiv:`2107.04916 [cond-mat.str-el]`.

[39] A. Georges, G. Kotliar, W. Krauth, and M. J. Rozenberg, "Dynamical mean-field theory of strongly correlated fermion systems and the limit of infinite dimensions", Rev. Mod. Phys. **68**, 13–125 (1996).

[40] F. Aryasetiawan, M. Imada, A. Georges, G. Kotliar, S. Biermann, and A. I. Lichtenstein, "Frequency-dependent local interactions and low-energy effective models from electronic structure calculations", Phys. Rev. B **70**, 195104 (2004).

[41] M. Casula, P. Werner, L. Vaugier, F. Aryasetiawan, T. Miyake, A. J. Millis, and S. Biermann, "Low-energy models for correlated materials: bandwidth renormalization from coulombic screening", Phys. Rev. Lett. **109**, 126408 (2012).

[42] L. Vaugier, H. Jiang, and S. Biermann, "Hubbard U and hund exchange J in transition metal oxides: screening versus localization trends from constrained random phase approximation", Phys. Rev. B **86**, 165105 (2012).

[43] W. Metzner and D. Vollhardt, "Correlated lattice fermions in $d = \infty$ dimensions", Phys. Rev. Lett. **62**, 324–327 (1989).

[44] S. B. Bravyi and A. Y. Kitaev, "Fermionic quantum computation", Ann. Phys. (N. Y.) **298**, 210–226 (2002).

[45] R. C. Ball, "Fermions without fermion fields", Phys. Rev. Lett. **95**, 176407 (2005).

[46] F. Verstraete and J. I. Cirac, "Mapping local hamiltonians of fermions to local Hamiltonians of spins", J. Stat. Mech.: Theory Exp. **2005**, P09012–P09012 (2005).

[47] M. Steudtner and S. Wehner, "Fermion-to-qubit mappings with varying resource requirements for quantum simulation", New J. Phys. **20**, 063010 (2018).

[48] Y.-A. Chen, A. Kapustin, and D. Radicevic, "Exact bosonization in two spatial dimensions and a new class of lattice gauge theories", Ann. Phys. (N. Y.) **393**, 234–253 (2018).

[49] K. Setia, S. Bravyi, A. Mezzacapo, and J. D. Whitfield, "Superfast encodings for fermionic quantum simulation", Phys. Rev. Research **1**, 033033 (2019).

[50] Z. Jiang, J. McClean, R. Babbush, and H. Neven, "Majorana loop stabilizer codes for error mitigation in fermionic quantum simulations", Phys. Rev. Applied **12**, 064041 (2019).

[51] C. Cade, L. Mineh, A. Montanaro, and S. Stanisic, "Strategies for solving the Fermi-Hubbard model on near-term quantum computers", Phys. Rev. B **102**, 235122 (2020).

[52] T. Hagge, *Optimal fermionic swap networks for Hubbard models*, 2021, arXiv:2001.08324 [quant-ph].

[53] M. A. Nielsen and I. L. Chuang, *Quantum computation and quantum information: 10th anniversary edition* (Cambridge University Press, Cambridge, 2010).

[54] K. Bharti et al., *Noisy intermediate-scale quantum (NISQ) algorithms*, 2021, arXiv:2101.08448 [quant-ph].

[55] A. Peruzzo, J. McClean, P. Shadbolt, M.-H. Yung, X.-Q. Zhou, P. J. Love, A. Aspuru-Guzik, and J. L. O'Brien, "A variational eigenvalue solver on a photonic quantum processor", Nat. Commun. **5**, 4213 (2014).

[56] L. Mineh and A. Montanaro, "Solving the hubbard model using density matrix embedding theory and the variational quantum eigensolver", Phys. Rev. B **105**, 125117 (2022).

[57] J. Watrous, "Quantum computational complexity", in *Encyclopedia of complexity and systems science*, edited by R. A. Meyers (Springer, New York, 2009), pp. 7174–7201.

[58] Z. Jiang, K. J. Sung, K. Kechedzhi, V. N. Smelyanskiy, and S. Boixo, "Quantum algorithms to simulate many-body physics of correlated fermions", Phys. Rev. Applied **9**, 044036 (2018).

[59] J. P. Bonilla Ataides, D. K. Tuckett, S. D. Bartlett, S. T. Flammia, and B. J. Brown, "The XZZX surface code", Nat. Commun. **12**, 2172 (2021).

[60] E. Dennis, A. Kitaev, A. Landahl, and J. Preskill, "Topological quantum memory", J. Math. Phys. **43**, 4452–4505 (2002).

[61] O. Higgott, M. Wilson, J. Hefford, J. Dborin, F. Hanif, S. Burton, and D. E. Browne, "Optimal local unitary encoding circuits for the surface code", Quantum **5**, 517 (2021).

[62] G. H. Low, N. Wiebe, N. M. Klco, and Y. Su, "Swap networks for quantum computation", pat. 16 / 438,409 (2019).

[63] A. A. Hagberg, D. A. Schult, and P. J. Swart, "Exploring network structure, dynamics, and function using NetworkX", in Proceedings of the 7th python in science conference, edited by G. Varoquaux, T. Vaught, and J. Millman (2008), pp. 11 –15.

[64] L. Lao and D. E. Browne, *2QAN: a quantum compiler for 2-local qubit Hamiltonian simulation algorithms*, 2021, arXiv:2108.02099 [quant-ph].

[65] O. Crawford, B. v. Straaten, D. Wang, T. Parks, E. Campbell, and S. Brierley, "Efficient quantum measurement of Pauli operators in the presence of finite sampling error", Quantum **5**, 385 (2021).

[66] A. Kandala, A. Mezzacapo, K. Temme, M. Takita, M. Brink, J. M. Chow, and J. M. Gambetta, "Hardware-efficient variational quantum eigensolver for small molecules and quantum magnets", Nature **549**, 242–246 (2017).

[67] V. Verteletskyi, T.-C. Yen, and A. F. Izmaylov, "Measurement optimization in the variational quantum eigensolver using a minimum clique cover", J. Chem. Phys. **152**, 124114 (2020).

[68] A. Jena, S. Genin, and M. Mosca, *Pauli partitioning with respect to gate sets*, 2019, arXiv:1907.07859 [quant-ph].

[69] T.-C. Yen, V. Verteletskyi, and A. F. Izmaylov, *Measuring all compatible operators in one series of a single-qubit measurements using unitary transformations*, 2020, arXiv:1907.09386 [quant-ph].

[70] P. Gokhale, O. Angiuli, Y. Ding, K. Gui, T. Tomesh, M. Suchara, M. Martonosi, and F. T. Chong, *Minimizing state preparations in variational quantum eigensolver by partitioning into commuting families*, 2019, arXiv:1907.13623 [quant-ph].

[71] X. Bonet-Monroig, R. Babbush, and T. E. O'Brien, "Nearly optimal measurement scheduling for partial tomography of quantum states", Phys. Rev. X **10**, 031064 (2020).

[72] I. Hamamura and T. Imamichi, "Efficient evaluation of quantum observables using entangled measurements", npj Quantum Inf. **6**, 56 (2020).

[73] Z. Baranyai, "On the factorization of the complete uniform hypergraphs", in *Infinite and finite sets (proc. intern. coll. keszthely, 1973)*, edited by A. Hajnal, R. Rado, and V. T. Sós (North-Holland, Amsterdam, 1975).

[74] J. Suntivich, H. A. Gasteiger, N. Yabuuchi, H. Nakanishi, J. B. Goodenough, and Y. Shao-Horn, "Design principles for oxygen-reduction activity on perovskite oxide catalysts for fuel cells and metal–air batteries", Nat. Chem. **3**, 546–550 (2011).

[75] J. Liu et al., "Coherent electric field manipulation of Fe3+ spins in PbTiO3", Sci. Adv. **7**, eabf8103 (2021).

[76] J. G. Bednorz and K. A. Müller, "Possible hightc superconductivity in the Ba-La-Cu-O system", Z. Phys. B **64**, 189–193 (1986).

[77] N.-G. Park, "Perovskite solar cells: an emerging photovoltaic technology", Mater. Today **18**, 65–72 (2015).

[78] C. C. Stoumpos et al., "Crystal growth of the perovskite semiconductor CsPbBr3: a new material for high-energy radiation detection", Cryst. Growth Des. **13**, 2722–2727 (2013).

[79] C. Lupo, E. Sheridan, E. Fertitta, D. Dubbink, C. J. Pickard, and C. Weber, "From Slater to Mott physics by epitaxially engineering electronic correlations in oxide interfaces", npj Comput. Mater. **7**, 94 (2021).

[80] N. A. Spaldin and R. Ramesh, "Advances in magnetoelectric multiferroics", Nat. Mater. **18**, 203–212 (2019).

[81] H.-Y. Ye et al., "Metal-free three-dimensional perovskite ferroelectrics", Science **361**, 151–155 (2018).

[82] X. Li, Z. Lin, N. Jin, X. Yang, Y. Du, L. Lei, P. Rozier, P. Simon, and Y. Liu, "Perovskite-type SrVO3 as high-performance anode materials for lithium-ion batteries", Adv. Mater. **n/a**, 2107262 (2021).

[83] Yue Jin Shan, L. Chen, Y. Inaguma, M. Shikano, M. Itoh, and T. Nakamura, "Lithium insertion into ceramic SrVO3-δ", Solid State Ion. **70-71**, 429–433 (1994).

[84] M. R. Filip and F. Giustino, "The geometric blueprint of perovskites", Proc. Natl. Acad. Sci. U.S.A. **115**, 5397–5402 (2018).

[85] J. P. Perdew, K. Burke, and M. Ernzerhof, "Generalized gradient approximation made simple", Phys. Rev. Lett. **77**, 3865–3868 (1996).

[86] V. I. Anisimov, F Aryasetiawan, and A. I. Lichtenstein, "First-principles calculations of the electronic structure and spectra of strongly correlated systems: the LDA+U method", J. Phys. Condens. Matter **9**, 767–808 (1997).

[87] F. Aryasetiawan and O. Gunnarsson, "The GW method", Reports on Progress in Physics **61**, 237–312 (1998).

[88] T. Ribic, E. Assmann, A. Tóth, and K. Held, "Cubic interaction parameters for t_{2g} Wannier orbitals", Phys. Rev. B **90**, 165105 (2014).

[89] K. F. Garrity and K. Choudhary, "Database of Wannier tight-binding Hamiltonians using high-throughput density functional theory", Sci. Data **8**, 106 (2021).

[90] L. Clinton, J. Bausch, and T. Cubitt, "Hamiltonian simulation algorithms for near-term quantum hardware", Nat. Commun. **12**, 4989 (2021).

[91] T. E. O'Brien et al., *Purification-based quantum error mitigation of pair-correlated electron simulations*, 2022, arXiv:2210.10799 [quant-ph].

[92] S. Stanisic, J. L. Bosse, F. M. Gambetta, R. A. Santos, W. Mruczkiewicz, T. E. O'Brien, E. Ostby, and A. Montanaro, "Observing ground-state properties of the Fermi-Hubbard model using a scalable algorithm on a quantum computer", Nat. Commun. **13**, 5743 (2022).

[93] X. Ren, P. Rinke, C. Joas, and M. Scheffler, "Random-phase approximation and its applications in computational chemistry and materials science", J. Mater. Sci. **47**, 7447–7471 (2012).

[94] I. V. Solovyev, "Combining DFT and many-body methods to understand correlated materials", J. Phys. Condens. Matter **20**, 293201 (2008).

[95] A. Damle, L. Lin, and L. Ying, "SCDM-k: localized orbitals for solids via selected columns of the density matrix", J. Comput. Phys. **334**, 1–15 (2017).

[96] W. Kohn, "Density functional and density matrix method scaling linearly with the number of atoms", Phys. Rev. Lett. **76**, 3168–3171 (1996).

[97] E. Prodan and W. Kohn, "Nearsightedness of electronic matter", Proc. Natl. Acad. Sci. U.S.A. **102**, 11635–11638 (2005).

[98] J. P. F. LeBlanc et al., "Solutions of the two-dimensional Hubbard model: benchmarks and results from a wide range of numerical algorithms", Phys. Rev. X **5**, 041041 (2015).
