# Supplementary material for: Towards near-term quantum simulation of materials
Source: Nat Commun. 2024 Jan 24;15:211. doi: 10.1038/s41467-023-43479-6 (PMC10808561; doi:10.1038/s41467-023-43479-6)
Supplement: Supplementary file 2 — Peer Review File [file 41467_2023_43479_MOESM2_ESM.pdf]

nature portfolio

Peer Review File

Towards near-term quantum simulation of materials

Open Access This file is licensed under a Creative Commons Attribution 4.0 International License, which permits use, sharing, adaptation, distribution and reproduction in any medium or format, as long as you give appropriate credit to the original author(s) and the source, provide a link to the Creative Commons license, and indicate if changes were made. In the cases where the authors are anonymous, such as is the case for the reports of anonymous peer reviewers, author attribution should be to 'Anonymous Referee' followed by a clear attribution to the source work. The images or other third party material in this file are included in the article's Creative Commons license, unless indicated otherwise in a credit line to the material. If material is not included in the article's Creative Commons license and your intended use is not permitted by statutory regulation or exceeds the permitted use, you will need to obtain permission directly from the copyright holder. To view a copy of this license, visit http://creativecommons.org/licenses/by/4.0/.

REVIEWER COMMENTS

Reviewer #1 (Remarks to the Author):

In this manuscript, the authors carry out a comprehensive study of a strategy for simulating (qualitative approximations to) real materials on a near-term quantum computer. Starting from the electronic structure Hamiltonian, they begin by generating effective Hamiltonians by using efficient classical calculations to select an active space and find a localized basis. They map the fermionic operators of these effective Hamiltonians to qubit operators using a combination of the techniques of [11] with the standard Jordan-Wigner mapping, with the aim of minimizing the circuit depth required to implement a Trotter step. They optimize the circuits for implementing a Trotter step and the strategies for efficiently measuring the expectation value of the energy. This composite approach is applied to a variety of systems, ultimately producing detailed cost estimates (in terms of the number of qubits and the circuit depth) for several material systems.

Much of the work of this paper involves thoughtfully combining existing approaches and carefully working out the details in their implementation. The strategies they use to calculate the effective Hamiltonians are well-known classically (and also well-known to require care during their application). Similarly, their fermion-to-qubit mappings build substantially on prior work, although it appears that hybridizing previous approaches is a new combination. Overall, the main technical achievement here is not in the individual components, but in their end-to-end analysis and comparison with more naive approaches.

As a referee, I have nothing but praise for the quality of the technical work and the clarity of the presentation. This paper will clearly be a useful guide to future researchers extending this line of work. I do, however, take some issue with the conclusions touted by the paper. The last sentence of the abstract reads "our results show that realistic materials simulation may be feasible on quantum computers without necessarily requiring fully scalable, fault-tolerant quantum computers, providing …"

First of all, I don't think that it's fair to call these simulations "realistic materials simulations." It is perfectly reasonable to hope that interesting physical phenomena will be

qualitatively captured by the small active space Hamiltonians considered in this work, but it's hard to imagine (and the paper does not make the case) that these simulations will provided quantitative accuracy. This is especially true when one considers studying dynamics, where even a reasonable accurate approximation to the low-energy states of the system might not be sufficient. This point may be minor, but I think that care should be taken so as not to oversell the potential of their approach. Besides changing the particular phrase in the abstract, I think the main text would benefit from some short discussion of the potential downsides of treating real materials using these model Hamiltonian approaches (and perhaps a bit about the path towards systematically improving them).

More seriously, I don't see how the authors have supported the claim that these resource estimates are practical for a NISQ machine. At a glance, even their smallest example would require two-qubit gate error rates on the order of 1E-5 to implement a single Trotter step with O(1) errors. Any real application would probably require (at least) several Trotter steps, and at that point the precision requirements imply a device that is far below the error-correction threshold. I would be more comfortable recommending publication if the authors used their resource estimates to outline the requirements that a NISQ device would need to satisfy in order to implement these algorithms under a range of plausible assumptions (about e.g., the cost of error mitigation and other factors) and refrained from making the positive claim about feasibility unless they can substantiate it more thoroughly.

Overall, I think it is highly likely that the proposed approach will never actually be implemented on a NISQ machine for any reasonably large system. However, I'm optimistic that the same kinds of techniques will prove useful in early fault-tolerance and I would like to see the paper published somewhere appropriate so that the scientific community can build on their work in this direction.

Reviewer #2 (Remarks to the Author):

The manuscript entitled "Towards near-term quantum simulation of materials" by Laura Clinton and co-workers introduces a framework to perform calculations on strongly correlated periodic solids using

future fault-tolerant quantum computers.

The main text puts forth a complex multi-step program how to translate the problem of electronic state of solids to a form that is amenable for future quantum computers. Along this way authors must choose between several alternatives guided by a requirement to reduce qubit and gate resources of quantum computers. Their milestones are:

1. Use a limited selection of bands, called as "active space" to reduce complexity of the corresponding Hamiltonians and associated correlated calculations

2. The use of maximally localized Wannier functions to increase the locality of electron-electron interaction in microscopic hamiltonians

3. Using tailored fermion-to-qubit transformations (encodings) to reduce weights of Pauli operators (lengths of the corresponding Pauli strings) in qubit Hamiltonians

4. Employ the variation quantum eigensolver (VQE) to solve for the ground state energy (equilibrium properties) and time dynamics simulation (TDS) for spectral properties.

While this program is definitely significantly sounding and solid, there are many literature omissions as well as straight mistakes in its discussion. First of all, the manuscript ignores almost completely (except noting in passing in lines 310-313) the main competitor in the realm of accurately solving the electronic structure problems for solids: density matrix embedding/density embedding theories, DMET and DET. DET has been successfully applied to solids as early as in 2014 [J. Chem. Phys. 141, 054113 (2014)]. In recent years, DMET is evolved

into rather mature theory for correlation in solids including strongly correlated systems, see for example, [J. Chem. Theory Comput. 16, 119 (2020)], Even more, the active space concept and Wannier functions to determine the impurity Hamiltonian are already exploited in J. Chem. Theory Comput. 16, 130 (2020). In the manuscript, on the other hand, it appears that steps 1-2 are completely new and have never been assessed in literature.

There are few incorrect statements and formulae in general description of VQE. Namely, if a qubit Hamiltonian is written as

$$\sum_k h_k, (1)$$

then it is implied that h_k are Pauli strings (multi-qubit Pauli products, Pauli "words") multiplied by corresponding numerical coefficients deduced from one- and two-electron integrals. Then VQE Ansatz *cannot* be written as

$$\prod_k \exp(i t_k h_k) (2)$$

since amplitudes can be only attached to "bare" Pauli strings, so Hamiltonian must be probably written as

$$\sum_k C_k h_k (3)$$

Second, this description is *incorrect* as generators of entanglement in Eq.~(2) in VQE are *never* the same Pauli terms as in the Hamiltonian itself. The convergence of VQE energy estimates, as well as the depth and overall complexity of various VQE flavours depend *crucially* on the choice of generators; one of the most sophisticated choices are made by the iterative qubit cluster method (iQCC), see for example, J. Chem. Theory Comput. 16, 1055 (2022). Lastly, the

statements about poor suitability of the Jordan-Wigner transformation for VQE is incorrect: within iQCC the initial set of generators is *always* comprised of at most 4-qubit Pauli strings.

That is, almost every statement made by authors in justifying their program can be challenged by existing studies. Worse, the entire work looks like as an executive summary (main body of merely 7 pages) to a research *proposal* (87-page supplementary document) with very few concrete results. In fact, the main result of the paper is Table 1 with just resource estimates. Given the plethora of already published works on resource estimates in different setups, it is insufficiently important and impactful contribution to the field. Steps in the program require careful examination, which probably is worth of several separate publications.

Overall, my recommendation is to reject publication in Nature Communications as the manuscript is not solution to a well-known important problem, nor some groundbreaking experiment, and is not suitable for a broad/general audience. Authors did not provide strong and convincing arguments in favour of their problem compared to other state-of-the-art approaches. There are other journals such as PRX that may accept large and complex studies, which I can recommend.

Reviewer #3 (Remarks to the Author):

Materials science belongs to the most promising applications of quantum computers. Despite enormous ongoing progress in quantum technologies, the fault-tolerant regime still seems to be quite far and current focus is thus on hybrid quantum-classical algorithms with shallow circuits, such as the variational quantum eigensolver (VQE).

In this work, Clinton et al. present novel algorithms/improvements, which dramatically reduce the cost of VQE (and also time dynamics simulations) of real materials, and could be

realizable on the noisy near-term devices. As is shown in the Results section, the combination of several improvements (employing the locality of Hamiltonians in the Wannier basis, restricting quantum simulation to the relevant subspace of the Hilbert space, or ingenuine fermion-to-qubit mapping) can lead to six orders of magnitude shorter quantum circuits, which is remarkable.

Overall, the paper is very well written. I believe that the text has been polished many times (based on the history of its arXiv version). The supplementary information is exhaustive. In my opinion the presented developments are very important and fits the demands of the journal. I therefore recommend a publication in Nature Communications.

I have only few suggestions for improvement:

1) The idea of reducing the cost of quantum simulations by employing the (complete) active space was previously suggested by Reiher et al. for molecules (though not periodic systems). I think that this should be mentioned in the text and the paper of Reiher et al. (https://doi.org/10.1073/pnas.1619152114) cited.

2) The authors use Kohn-Sham orbital energies for selection of the active space. In quantum chemistry, selection of the active space is a very important issue and several highly sophisticated methods, e.g. based on the quantum information measures (https://doi.org/10.1103/PhysRevB.68.195116, https://doi.org/10.1016/j.chemphys.2005.10.018) have been developed. These methods were also automated (https://doi.org/10.1021/acs.jctc.6b00156) or even employed the machine learning (https://doi.org/10.1021/acs.jctc.1c00235). I believe that the aforementioned approaches can be used also for periodic materials, which could be mentioned in the text. This way, the active space could be possibly further shrunk in some cases.

3) Recently, there appeared the paper using Bloch and Wannier representations of periodic systems in the context of qubitized quantum phase estimation (10.1103/PhysRevResearch.5.013200). I think it should be also cited.

4) Despite the fact that the results (circuit depths) are very promising, they are still beyond current quantum hardware as mentioned by the authors. I would very much appreciate if the authors could briefly review capabilities of current hardware so that the reader can immediately see, how far we are from practical quantum simulations of real materials.

Response to reviewer #1:

In this manuscript, the authors carry out a comprehensive study of a strategy for simulating (qualitative approximations to) real materials on a near-term quantum computer. Starting from the electronic structure Hamiltonian, they begin by generating effective Hamiltonians by using efficient classical calculations to select an active space and find a localized basis. They map the fermionic operators of these effective Hamiltonians to qubit operators using a combination of the techniques of [11] with the standard Jordan-Wigner mapping, with the aim of minimizing the circuit depth required to implement a Trotter step. They optimize the circuits for implementing a Trotter step and the strategies for efficiently measuring the expectation value of the energy. This composite approach is applied to a variety of systems, ultimately producing detailed cost estimates (in terms of the number of qubits and the circuit depth) for several material systems.

Much of the work of this paper involves thoughtfully combining existing approaches and carefully working out the details in their implementation. The strategies they use to calculate the effective Hamiltonians are well-known classically (and also well-known to require care during their application). Similarly, their fermion-to-qubit mappings build substantially on prior work, although it appears that hybridizing previous approaches is a new combination. Overall, the main technical achievement here is not in the individual components, but in their end-to-end analysis and comparison with more naive approaches.

As a referee, I have nothing but praise for the quality of the technical work and the clarity of the presentation. This paper will clearly be a useful guide to future researchers extending this line of work. I do, however, take some issue with the conclusions touted by the paper. The last sentence of the abstract reads “our results show that realistic materials simulation may be feasible on quantum computers without necessarily requiring fully scalable, fault-tolerant quantum computers, providing …”

First of all, I don’t think that it’s fair to call these simulations “realistic materials simulations.” It is perfectly reasonable to hope that interesting physical phenomena will be qualitatively captured by the small active space Hamiltonians considered in this work, but it’s hard to imagine (and the paper does not make the case) that these simulations will provided quantitative accuracy. This is especially true when one considers studying dynamics, where even a reasonable accurate approximation to the low-energy states of the system might not be sufficient. This point may be minor, but I think that care should be taken so as not to oversell the potential of their approach. Besides changing the particular phrase in the abstract, I think the main text would benefit from some short discussion of the potential downsides of treating real materials using these model Hamiltonian approaches (and perhaps a bit about the path towards systematically improving them).

We thank the referee for these suggestions. Firstly, we have modified the particular phrase in the abstract to indicate “specific properties” and also included in the main text a more fleshed out discussion of model hamiltonian methods based on active space approaches, indicating

some strategies that can potentially improve them. We hope these additions clarify how our approach relates to materials simulations.

More seriously, I don't see how the authors have supported the claim that these resource estimates are practical for a NISQ machine. At a glance, even their smallest example would require two-qubit gate error rates on the order of 1E-5 to implement a single Trotter step with O(1) errors. Any real application would probably require (at least) several Trotter steps, and at that point the precision requirements imply a device that is far below the error-correction threshold. I would be more comfortable recommending publication if the authors used their resource estimates to outline the requirements that a NISQ device would need to satisfy in order to implement these algorithms under a range of plausible assumptions (about e.g., the cost of error mitigation and other factors) and refrained from making the positive claim about feasibility unless they can substantiate it more thoroughly.

Overall, I think it is highly likely that the proposed approach will never actually be implemented on a NISQ machine for any reasonably large system. However, I'm optimistic that the same kinds of techniques will prove useful in early fault-tolerance and I would like to see the paper published somewhere appropriate so that the scientific community can build on their work in this direction.

Although we think it is entirely reasonable to be pessimistic about the prospect of running these kinds of algorithms on NISQ devices, we believe the gate counts for some of these materials also leave significant room for optimism. The two qubit gate count for a single layer of the protocol we consider for $SrVO_3$ is approximately 7000 – whereas successful quantum simulations of model systems have been performed on existing hardware using two qubit gate counts of approximately 2500. We have included the gate counts for the compiled circuits in the main results table (Table 1), and a brief discussion of the prospect of running a practical simulation. We have also added a figure to the Supplementary Information (Fig. S39) comparing the required two qubit gate error as a function of the target circuit fidelity to implement a single layer for the various materials we consider. Ultimately the concrete conclusions of this paper are not that practical algorithms of this type can definitively be run on near term devices, but rather that material simulation on near term devices is a promising direction to pursue in earnest, and that a simulation of this type may be feasible in the near future, given further improvements to the hardware and/or the algorithms -- whereas a naive estimate would suggest that such simulations are well within the fault tolerant regime.

We completely agree that even in the case where these simulations are not possible on NISQ devices, the techniques we consider remain useful in the fault tolerant setting.

Response to reviewer #2:

The manuscript entitled "Towards near-term quantum simulation of materials" by Laura Clinton and co-workers introduces a framework to perform calculations on strongly correlated periodic solids using future fault-tolerant quantum computers.

The main text puts forth a complex multi-step program how to translate the problem of electronic state of solids to a form that is amenable for future quantum computers. Along this way authors must choose between several alternatives guided by a requirement to reduce qubit and gate resources of quantum computers. Their milestones are:

1. Use a limited selection of bands, called as "active space" to reduce complexity of the corresponding Hamiltonians and associated correlated calculations

2. The use of maximally localized Wannier functions to increase the locality of electron-electron interaction in microscopic hamiltonians

3. Using tailored fermion-to-qubit transformations (encodings) to reduce weights of Pauli operators (lengths of the corresponding Pauli strings) in qubit Hamiltonians

4. Employ the variation quantum eigensolver (VQE) to solve for the ground state energy (equilibrium properties) and time dynamics simulation (TDS) for spectral properties.

While this program is definitely significantly sounding and solid, there are many literature omissions as well as straight mistakes in its discussion. First of all, the manuscript ignores almost completely (except noting in passing in lines 310-313) the main competitor in the realm of accurately solving the electronic structure problems for solids: density matrix embedding/density embedding theories, DMET and DET. DET has been successfully applied to solids as early as in 2014 [J. Chem. Phys. 141, 054113 (2014)]. In recent years, DMET is evolved into rather mature theory for correlation in solids including strongly correlated systems, see for example, [J. Chem. Theory Comput. 16, 119 (2020)], Even more, the active space concept and Wannier functions to determine the impurity Hamiltonian are already exploited in J. Chem. Theory Comput. 16, 130 (2020). In the manuscript, on the other hand, it appears that steps 1-2 are completely new and have never been assessed in literature.

We thank the referee for these suggestions regarding embedding theories. We have included an additional discussion in the “Identifying the active space” subsection of the “Results” section and incorporated the above references.

There are few incorrect statements and formulae in general description of VQE. Namely, if a qubit Hamiltonian is written as

\sum_k h_k, (1)

then it is implied that h_k are Pauli strings (multi-qubit Pauli products, Pauli "words") multiplied by corresponding numerical coefficients deduced from one- and two-electron integrals. Then VQE Ansatz *cannot* be written as

\prod_k \exp(i t_k h_k) (2)

since amplitudes can be only attached to "bare" Pauli strings, so Hamiltonian must be probably written as

\sum_k C_k h_k (3)

In our description at the start of subsection A of the “Results” section (“Simulation algorithms”) (and, in more detail, in subsection D of “Methods” section and in Section IV of the Supplementary Information), we are not inherently assuming that the h_k terms are Pauli strings. Here, we are giving a high-level description of the Hamiltonian variational ansatz (Wecker et al., Phys. Rev. A **92**, 042303 (2015)), which is defined in terms of an arbitrary decomposition of an overall Hamiltonian as a sum of terms, where time-evolution according to each of the terms can be implemented efficiently. These terms may or may not be Pauli strings. In addition, if the terms h_k are weighted Pauli strings, it is still possible to define an ansatz in terms of weighted combinations of these terms. This may be useful, for example, if the same variational parameter is used for multiple terms (see, e.g., Cade et al., Phys. Rev. B **102**, 235122 (2020)).

Second, this description is *incorrect* as generators of entanglement in Eq.~(2) in VQE are *never* the same Pauli terms as in the Hamiltonian itself.

In the case of the Hamiltonian variational ansatz (HVA) (Wecker et al, Phys. Rev. A **92**, 042303 (2015)), which has been extensively used in the literature, and is our focus in this work, the terms in the VQE ansatz are often the Pauli terms in the Hamiltonian itself. This ansatz has been demonstrated to have a number of positive traits, such as preserving symmetries of the system, outperforming unitary coupled cluster methods (UCC) for larger molecules (Wecker et al, Phys. Rev. A **92**, 042303 (2015)), and avoiding the problem of barren plateaus (Wiersema et al., PRX Quantum **1**, 020319 (2020)). There are also good theoretical reasons why it is a good choice, related to adiabatic quantum computation. One could consider alternative variational ansatze, but we expect these to lead to higher circuit complexities. The review article Tilly et al., Physics Report **986**, 1 (2022) (https://doi.org/10.1016/j.physrep.2022.08.003) has more details on these points.

The convergence of VQE energy estimates, as well as the depth and overall complexity of various VQE flavours depend *crucially* on the choice of generators; one of the most sophisticated choices are made by the iterative qubit cluster method (iQCC), see for example, J. Chem. Theory Comput. 16, 1055 (2022). Lastly, the statements about poor suitability of the Jordan-Wigner transformation for VQE is incorrect: within iQCC the initial set of generators is *always* comprised of at most 4-qubit Pauli strings.

We thank the reviewer for pointing out these works. Although the iQCC is certainly an interesting variant of VQE, it is simply not what we consider in this work. Furthermore, we know of no evidence indicating that iQCC is always the best variational ansatz for VQE -- the cited paper demonstrates the efficacy but not the universal superiority of iQCC. A notable property of iQCC is that the choice of circuit elements of the variational ansatz are determined by an adaptive procedure in which the quantum computer plays an integral role, similar to ADAPT-VQE. Our aim with this work is to estimate costs for large material simulation in near term, potentially as yet non-existent, devices. It seems to us impossible to perform such an offline cost estimate for the iQCC method, or any other adaptive VQE methods, making them unsuitable for the aims of this work. Finally, even in the hypothetical scenario where it were the case that iQCC always outperformed the HVA in terms of both cost and accuracy, our results would then give an upper bound on the potential cost of performing VQE, which is precisely what we are arguing. We have no doubt these results can be improved upon, and it may well be that these improvements come from adaptive methods such as iQCC.

If one does use the HVA, or indeed many other variational ansatze, then it is the case that the circuit cost will depend on the fermionic representation -- and that Jordan-Wigner can be disadvantageous in this setting, as we show.

That is, almost every statement made by authors in justifying their program can be challenged by existing studies. Worse, the entire work looks like as an executive summary (main body of merely 7 pages) to a research *proposal* (87-page supplementary document) with very few concrete results. In fact, the main result of the paper is Table 1 with just resource estimates. Given the plethora of already published works on resource estimates in different setups, it is insufficiently important and impactful contribution to the field. Steps in the program require careful examination, which probably is worth of several separate publications.

We would like to emphasize that the significance of this work is that the resource estimates we supply are dramatically lower than one might expect, and that this is achieved using a novel approach, which synthesizes a number of existing and new ideas in a genuinely new way, rather than merely estimating the resources used by standard approaches. If it were the case that our methods yielded estimates that were comparable to existing estimates then we would agree – but our estimates yield many orders of magnitude reduction over estimates using standard methods. We believe this is significant because it changes the landscape of what may be possible.

Overall, my recommendation is to reject publication in Nature Communications as the manuscript is not solution to a well-known important problem, nor some groundbreaking experiment, and is not suitable for a broad/general audience. Authors did not provide strong and convincing arguments in favour of their problem compared to other state-of-the-art approaches. There are other journals such as PRX that may accept large and complex studies, which I can recommend.

We respectfully disagree: our view is that - as highlighted in Table 1 - our results significantly change the landscape of what may be possible with relatively near-term quantum computers, and address key problems in materials simulation which are of interest to a broad community including physicists, chemists and computer scientists.

Response to reviewer #3:

Materials science belongs to the most promising applications of quantum computers. Despite enormous ongoing progress in quantum technologies, the fault-tolerant regime still seems to be quite far and current focus is thus on hybrid quantum-classical algorithms with shallow circuits, such as the variational quantum eigensolver (VQE).

In this work, Clinton et al. present novel algorithms/improvements, which dramatically reduce the cost of VQE (and also time dynamics simulations) of real materials, and could be realizable on the noisy near-term devices. As is shown in the Results section, the combination of several improvements (employing the locality of Hamiltonians in the Wannier basis, restricting quantum simulation to the relevant subspace of the Hilbert space, or ingenuine fermion-to-qubit mapping) can lead to six orders of magnitude shorter quantum circuits, which is remarkable.

Overall, the paper is very well written. I believe that the text has been polished many times (based on the history of its arXiv version). The supplementary information is exhaustive. In my opinion the presented developments are very important and fits the demands of the journal. I therefore recommend a publication in Nature Communications.

I have only few suggestions for improvement:

1) The idea of reducing the cost of quantum simulations by employing the (complete) active space was previously suggested by Reiher et al. for molecules (though not periodic systems). I think that this should be mentioned in the text and the paper of Reiher et al. (https://doi.org/10.1073/pnas.1619152114) cited.

We thank the referee for pointing this out. We have included a short paragraph (including also some comments related to point (2) below) mentioning this work for molecular systems in the main text in the "Previous work" subsection of the “Results” section.

2) The authors use Kohn-Sham orbital energies for selection of the active space. In quantum chemistry, selection of the active space is a very important issue and several highly sophisticated methods, e.g. based on the quantum information measures (https://doi.org/10.1103/PhysRevB.68.195116, https://doi.org/10.1016/j.chemphys.2005.10.018) have been developed. These methods were also automated (https://doi.org/10.1021/acs.jctc.6b00156) or even employed the machine learning (https://doi.org/10.1021/acs.jctc.1c00235). I believe that the aforementioned approaches can be used also for periodic materials, which could be mentioned in the text. This way, the active space could be possibly further shrunk in some cases.

We thank the referee for pointing out these helpful references. We have included these in the "Previous work" subsection of the “Results” section.

3) Recently, there appeared the paper using Bloch and Wannier representations of periodic systems in the context of qubitized quantum phase estimation (10.1103/PhysRevResearch.5.013200). I think it should be also cited

We thank the referee for pointing this reference out to us and we agree it should be included. We have cited it in the "Previous work" subsection of the “Results” section.

4) Despite the fact that the results (circuit depths) are very promising, they are still beyond current quantum hardware as mentioned by the authors. I would very much appreciate if the authors could briefly review capabilities of current hardware so that the reader can immediately see, how far we are from practical quantum simulations of real materials.

As remarked in our response to Referee 1, we have added a discussion of this important point to the “Discussion” section of the main text, as well as a figure showing the two qubit gate error required to implement a layer of VQE as a function of the circuit target fidelity for the various materials we consider in Section IX.C.4 of the Supplementary Information (see Fig. S39).

REVIEWERS' COMMENTS

Reviewer #1 (Remarks to the Author):

In this revised version of the manuscript, the authors have fully addressed my concerns about the tone of the paper, which struck me as overly optimistic when I reviewed an earlier version of the manuscript.

Furthermore, I am confused by some of the technical comments made by the second referee and I urge the editors to disregard them. They appear to misunderstand that the authors were focused on providing cost estimates for the Hamiltonian variational anstaz, which, as the authors' response points out, has some attractive theoretical properties. Within the context of that ansatz, I believe that the authors statements are correct and the referee's are not. I also agree with the authors that studying adaptive methods like ADAPT-VQE, qubit-ADAPT-VQE, or iQCC would compound the difficulty in providing meaningful resource estimates at this scale and I think they made a reasonable choice in not considering them.

I appreciate the care the authors have taken in addressing my comments and I now fully recommend publication.

Reviewer #2 (Remarks to the Author):

In the revised version of the manuscript "Towards near-term quantum simulation of materials" author carefully addressed the issues that were raised by reviewers. However, the conceptual disagreement between authors and myself are still present. In my opinion, authors did not provide convincing arguments in favour of their approach to "change the landscape of what may be possible". Despite they claim orders-of-magnitude improvements in the number of gates, reduced qubit count and circuit depths, it is completely *unclear* what are the trade-offs one will be paying implementing their composite method. There are multiple places where computational savings may results in diminished accuracy, systematic deviations or even completely nonsensical results, but the manuscript contains not a single number reporting such evidence. I did not find in the main text how the approximations

made could affect, say, lattice parameters or fundamental gaps. Authors completed only preliminary calculations (relegated to supplementary, so they could be considered as "unimportant") at the DFT level for bands and Coulomb matrix elements in the Wannier basis. While these steps are indispensable in implementation of their program, the most interesting part, the execution of the quantum algorithm on (at least) a simulator, are missing. Authors could have done that for computationally feasible model systems, but this verification is missing. With all said above, I strongly believe that the proposed program remains mostly speculative and it is premature to publish it in Nature Communications as a work that "change the landscape of what may be possible".

Reviewer #1 (Remarks to the Author):

In this revised version of the manuscript, the authors have fully addressed my concerns about the tone of the paper, which struck me as overly optimistic when I reviewed an earlier version of the manuscript.

Furthermore, I am confused by some of the technical comments made by the second referee and I urge the editors to disregard them. They appear to misunderstand that the authors were focused on providing cost estimates for the Hamiltonian variational anstaz, which, as the authors' response points out, has some attractive theoretical properties. Within the context of that ansatz, I believe that the authors statements are correct and the referee's are not. I also agree with the authors that studying adaptive methods like ADAPT-VQE, qubit-ADAPT-VQE, or iQCC would compound the difficulty in providing meaningful resource estimates at this scale and I think they made a reasonable choice in not considering them.

I appreciate the care the authors have taken in addressing my comments and I now fully recommend publication.

Response

We thank the referee for their assessment of our work and appreciate their recommendation.

Reviewer #2 (Remarks to the Author):

In the revised version of the manuscript "Towards near-term quantum simulation of materials" author carefully addressed the issues that were raised by reviewers. However, the conceptual disagreement between authors and myself are still present. In my opinion, authors did not provide convincing arguments in favour of their approach to "change the landscape of what may be possible". Despite they claim orders-of-magnitude improvements in the number of gates, reduced qubit count and circuit depths, it is completely *unclear* what are the trade-offs one will be paying implementing their composite method. There are multiple places where computational savings may results in diminished accuracy, systematic deviations or even completely nonsensical results, but the manuscript contains not a single number reporting such evidence. I did not find in the main text how the approximations made could affect, say, lattice parameters or fundamental gaps. Authors completed only preliminary calculations (relegated to supplementary, so they could be considered as "unimportant") at the DFT level for bands and Coulomb matrix elements in the Wannier basis. While these steps are indispensable in implementation of their program, the most interesting part, the execution of the quantum algorithm on (at least) a simulator, are missing. Authors could have done that for computationally feasible model systems, but this verification is missing. With all said above, I strongly believe that the proposed program remains mostly speculative and it is premature to publish it in Nature Communications as a work that "change the landscape of what may be possible".

Response

Although we agree with the reviewer that the effects of the approximations introduced within our approach (mainly, the selection of an active space) are hard to quantify, we would like to point out that this issue is common to all (classical or quantum) embedding and active-space-based methods. The latter have proven capable of capturing some quantitative and qualitative properties of materials. However, a rigorous treatment of such approximations is an open problem and it is beyond the scope of our work.

To make this point clearer, we have added the following sentence in the Discussion section of the updated manuscript:

> “First, although ab-initio embedding and active space based methods have shown the potential to improve the description of certain properties of materials, a rigorous quantification of the trade-offs introduced by these approximations in the simulation of large materials' properties and dynamics is an open problem and will require additional investigation".
